# Supplementary material for: Hydrogen-helium immiscibility boundary in gas-giant planetary interiors from machine-learning molecular dynamics
Source: Nat Commun. 2026 Sep 1;17:10382. doi: 10.1038/s41467-026-77117-8 (PMC13627725; doi:10.1038/s41467-026-77117-8)
Supplement: Supplementary file 1 [file 41467_2026_77117_MOESM1_ESM.pdf]

Supplementary Information
Hydrogen–helium immiscibility boundary in gas-giant planetary interiors from machine-learning molecular dynamics

Xiaoyu Wang,[1] Sebastien Hamel,[2] and Bingqing Cheng[1,3,4,5,6,*]

[1] *Department of Chemistry, UC Berkeley, California 94720, United States*

[2] *Lawrence Livermore National Laboratory, Livermore, CA, USA*

[3] *The Institute of Science and Technology Austria, Am Campus 1, 3400 Klosterneuburg, Austria*

[4] *Materials Sciences Division, Lawrence Berkeley National Laboratory, Berkeley, CA, USA*

[5] *Chemical Sciences Division, Lawrence Berkeley National Laboratory, Berkeley, CA, USA*

[6] *Bakar Institute of Digital Materials for the Planet, UC Berkeley, California 94720, United States*

(Dated: July 29, 2026)

* bingqingcheng@berkeley.edu

CONTENTS

I. Details on the DFT calculations 3

A. DFT calculations with respect to the plane-wave cutoff energy 4

B. Finite-size effects on DFT calculations 4

II. Details on constructing the MLPs 6

A. Training set 6

B. MLP training 7

C. Validation 9

III. Benchmarks of the MLPs 9

A. NVT simulations based on vdW-DF 10

1. Pure H 10

2. Pure He 10

3. H/He mixture 10

IV. Simulations using the MLP 17

A. Sensitivity analysis regarding system size 17

B. MLP MD simulations 21

C. Promoting MLP-derived ΔG^{mix} to the DFT level 22

D. Computational performance of MLPs 24

E. Chemical potentials of the H/He System 24

V. Atomic–molecular phase transition of hydrogen 31

VI. Thermodynamic models for H/He mixtures 33

A. Third-order Redlich–Kister model 33

B. Prediction of the immiscibility boundaries 39

C. Non-ideal mixing behavior 40

VII. Path integral molecular dynamics simulations 42

VIII. Supplementary References 43

I. DETAILS ON THE DFT CALCULATIONS

We performed production density functional theory (DFT) calculations using the plane-wave code VASP [1–3], which treats ionic motion classically and computes forces quantum mechanically through finite-temperature DFT. To assess the impact of the chosen functional, three exchange-correlation functionals were employed: Perdew–Burke–Ernzerhof (PBE) [4], van der Waals density functional (vdW-DF) [5], and Heyd–Scuseria–Ernzerhof (HSE) hybrid functional [6, 7]. We sampled the Brillouin zone using a k-point grid resolution of at least 0.02 Å^{-1} for the PBE and vdW-DF calculations with a plane-wave energy cutoff of 1200 eV, while the HSE calculations were performed using a resolution of 0.03 Å^{-1}. For the Δ-learning model from vdW-DF to HSE, the baseline vdW-DF energies and forces were recalculated at 0.03 Å^{-1} to ensure consistency with the HSE results. We used the hard version of the hydrogen PBE projector augmented wave (PAW) potential (`PAW_PBE H_h 06Feb2004`), while for helium we used the only available version (`PAW_PBE He 05Jan2001`) from the VASP library.

We also re-analyzed previous *ab initio* molecular dynamics (AIMD) trajectories from a previous study based on vdW-DF [8], which performed more than 7500 NVT AIMD simulations of H/He mixtures with a Nosé–Hoover thermostat in VASP, covering 29 helium fractions (x_{He}) across 15 temperatures (T = 1000–15000 K) and multiple pressures (P = 20–3000 GPa). In particular, their calculations used the Baldereschi mean-value k-point [9] and a timestep of 0.2 fs for 64-electron systems, achieving pressure convergence within 1%.

To validate the accuracy of our DFT calculations, we performed two additional tests based on vdW-DF. First, we examined the convergence of the DFT results with respect to the plane-wave cutoff energy to ensure that the chosen computational setup yields well-converged energies, forces, and pressures. Second, we assessed finite-size effects by comparing DFT energies obtained for the same thermodynamic conditions at two different system sizes, $N = 64$ and $N = 1024$. Together, these tests confirm that our DFT calculations are sufficiently converged with respect to both plane-wave cutoff and cell size.

A. DFT calculations with respect to the plane-wave cutoff energy

We performed a cutoff-convergence test over the range of 700–1300 eV and checked the convergence of energy, forces, and pressure, as shown in Fig. S1. The results show that the energy converges at a cutoff of 1200 eV, since it changes by only 0.235 meV/atom (0.015%) from 1100 to 1200 eV and by only 0.099 meV/atom (0.007%) from 1200 to 1300 eV. Correspondingly, the atomic forces change marginally over the tested range: from 1100 to 1200 eV, the relative changes in the three force components are all below 0.13%, while from 1200 to 1300 eV they are at most 0.06%. Although the pressure still shows a small monotonic increase with increasing cutoffs, its change is already reduced to 0.147 GPa (0.019%) between 1200 and 1300 eV, which does not affect the conclusions of this work. Therefore, ENCUT = 1200 eV was adopted in all DFT calculations to ensure reliable convergence.

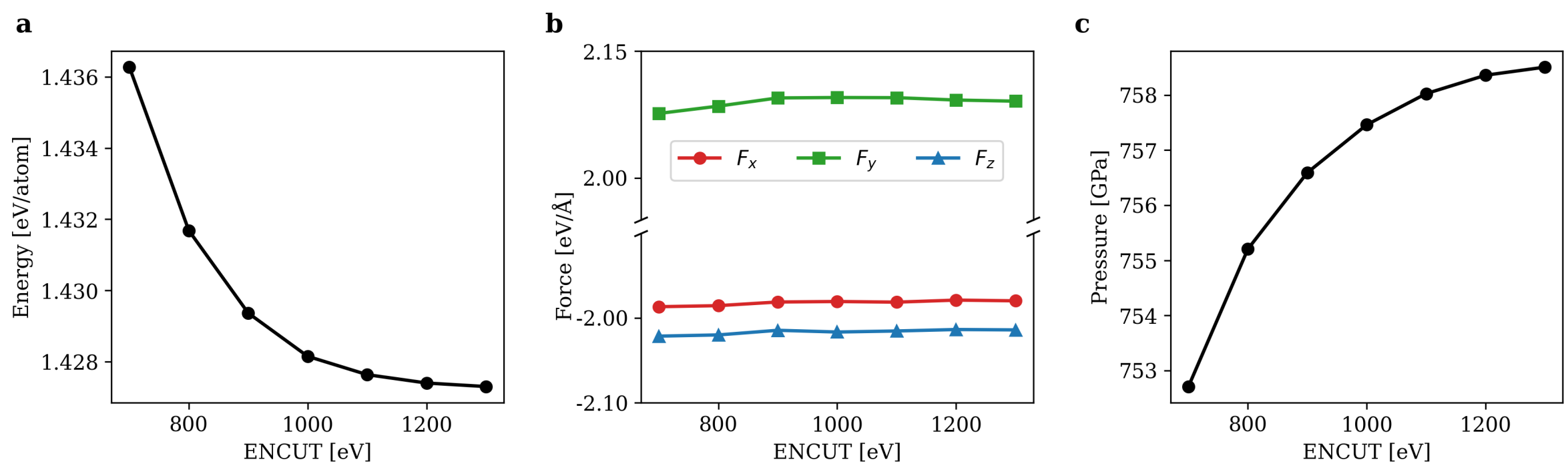

FIG. S1. Convergence of DFT-calculated energy (a), force (b), and pressure (c) with respect to the plane-wave cutoff energy for the H/He system at 800 GPa and 7000 K.

B. Finite-size effects on DFT calculations

To directly assess the finite-size effects in our DFT reference data, we evaluated both vdW-DF DFT and N2P2 MLP energies for a dataset of 7000 snapshots at two system sizes, $N = 64$ and $N = 1024$. For each system size, the dataset includes 12 helium compositions ($x_{\mathrm{He}} = 0.016$, 0.047, 0.11, 0.20, 0.30, 0.39, 0.55, 0.61, 0.73, 0.80, 0.92, and 0.98) at 150 GPa and 2000 K, as well as at 150 GPa and 5000 K, and 11 compositions (x_{He} = 0.016–0.92) at 800 GPa and 7000 K. As shown in Fig. S2, the DFT energies obtained for $N = 64$ and $N = 1024$ are broadly consistent within uncertainty across all investigated thermodynamic

conditions.

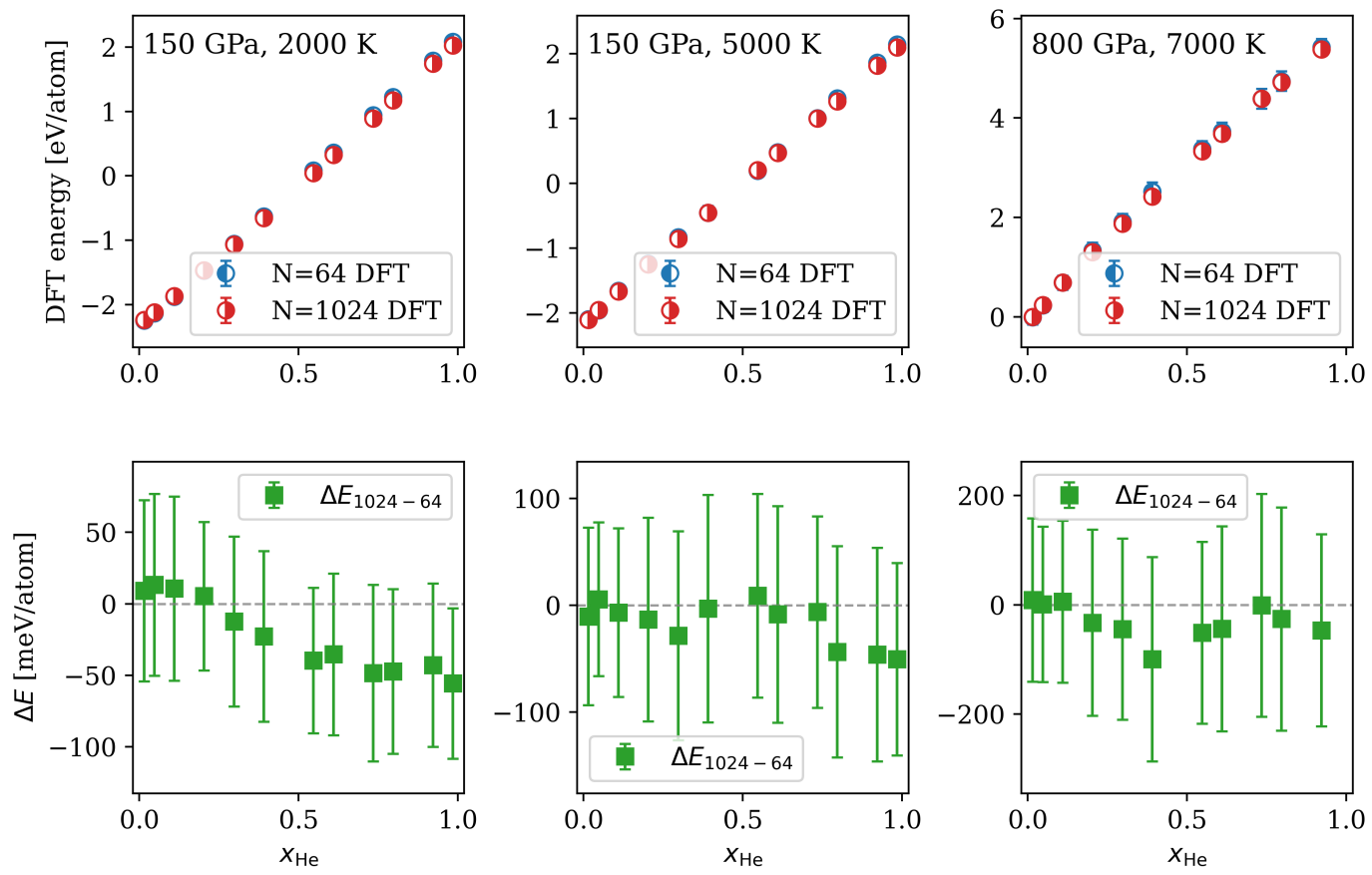

FIG. S2. Per-atom energies derived from vdW-DF DFT for system-size $N = 64$ and $N = 1024$ as a function of helium fraction, x_{He}, for H/He mixtures at three thermodynamic conditions: 150 GPa and 2000 K, 150 GPa and 5000 K, and 800 GPa and 7000 K. For each system size, DFT energies are shown in blue and MLP energies in red. Error bars indicate the standard error of the mean over the 100 sampled configurations.

II. DETAILS ON CONSTRUCTING THE MLPS

To achieve first-principles accuracy, we developed flexible machine learning potentials (MLPs) for the high-pressure H/He system. To assess the influence of model architectures and select one with an optimal efficiency/accuracy balance, we tested three MLP architectures: the parallelizable and CPU-efficient N2P2 [10], the more recent and accurate MACE [11] and CACE [12] models. In total, we developed 8 MLPs using three types of architectures trained on data from three XC functionals (PBE, vdW-DF, HSE). Six MLPs were obtained by direct fitting: one for each of the three architectures for both PBE and vdW-DF. The other two MLPs were developed using a Δ-learning strategy in the N2P2 package: a vdW-DF MLP was trained on the Δ component Δ(PBE$\rightarrow$vdW-DF) relative to the PBE MLP baseline, and an HSE MLP was trained on Δ(vdW-DF$\rightarrow$HSE) relative to the vdW-DF MLP baseline. We evaluated the performance of all MLPs for H/He mixtures by comparing energetic and thermodynamic properties against AIMD reference data [8] and one another, as discussed below.

A. Training set

The vdW-DF training set comprised 13,389 configurations (1,553,967 atoms) with varying x_{He} across a broad range of P–T conditions (1–1200 GPa and 500–16000 K), which was generated via a combination of strategies: AIMD simulations, random structure searches, active learning, and adapting from previous AIMD calculations for high-pressure H/He mixtures [8]. We then obtained the training set for the PBE MLP by recomputing the PBE energies and forces for all these configurations, as well as supplementing with a previous pure hydrogen training set [13]. To make the DFT computational cost of generating the training set for the HSE MLP tractable, we prepared a subset of 1968 selected configurations that were recomputed with HSE for Δ-learning [10]. As a validation of this Δ-learning strategy, we additionally built a vdW-DF subset of 2699 configurations to fit the Δ-learning MLP Δ(PBE$\rightarrow$vdW-DF).

B. MLP training

a. N2P2 We applied the N2P2 code [10] to train the Behler–Parrinello artificial neural networks [14] on datasets for the three functionals (PBE, vdW-DF, HSE) with a 10% test split. In this framework, each atomic environment is defined by the relative coordinates of neighboring atoms within a cutoff radius of 8 Bohr radius ($r_c = 4.23$ Å). To enforce rotational and permutational invariance, we employed Behler–Parrinello symmetry functions: 119 for hydrogen and 113 for helium, including radial and angular terms with angular prefactors ($\lambda = -1, +1$) and exponents ($\zeta = 1.0, 4.0, 16.0$).

b. CACE We trained CACE models on the vdW-DF and PBE datasets, with a 95%/5% training/testing split. For the CACE representation, we used a cutoff radius of $r_c = 3.0$ Å, 6 Bessel radial functions with a radial embedding dimension of $c = 12$, a maximum angular momentum of $l_{\text{max}} = 3$, a maximum body order of $\nu_{\text{max}} = 3$, 2 embedding layers ($N_{\text{embedding}} = 2$), and one message-passing layer.

c. MACE To fit the MACE MLPs for vdW-DF and PBE, we divided each dataset into training (11,812 configurations), validation (241 configurations), and test (1336 configurations) sets. Specifically, the equivariant MACE model used a cutoff of $r_c = 3.0$ Å, an angular-momentum cutoff of $L_{\text{max}} = 1$ (equivariant), 64 channels, and two interaction layers (i.e., one message-passing layer).

To further validate our training strategy, we examined several alternative fitting protocols, including models trained with stress data and models trained on restricted subsets of the dataset. To assess whether including stress data in the training improves the model, we trained both MACE and CACE models with stress tensors included in the fit and compared them with the corresponding models trained using only energies and forces. The resulting testing errors are summarized in Table S2, which shows that adding stress or virial information to the training has only a negligible effect on the overall model performance.

We also tested whether restricting the training set to a narrower thermodynamic region could improve the model performance within the same region. For this purpose, we trained an additional MACE model using only configurations between 400 and 600 GPa and between 3000 and 6000 K, and then evaluated it on test configurations from the same P–T window. We compared its performance with that of the original MACE model trained on the full dataset. As shown in Table S3, the model trained on the full dataset performs slightly

TABLE S1. Training and testing errors (RMSE) for different MLP architectures based on the vdW-DF, PBE, and HSE functionals.

Functional	ML package	Training Error (RMSE)		Testing Error (RMSE)	
		Energy [meV/atom]	Force [meV/Å]	Energy [meV/atom]	Force [meV/Å]
vdW-DF	N2P2	32.49	445.12	33.38	454.58
	MACE	13.10	187.30	12.50	192.00
	CACE	8.30	273.88	7.25	272.75
	Δ(PBE→vdW-DF)	1.16	39.77	1.18	40.54
PBE	N2P2	15.55	432.81	15.38	444.74
	MACE	11.50	174.70	11.40	179.90
	CACE	8.09	263.27	7.12	261.34
HSE	Δ(vdW-DF→HSE)	10.89	246.91	13.22	248.26

TABLE S2. Comparison of testing errors for MLPs trained with and without stress tensors.

Model	RMSE E [meV/atom]	RMSE F [meV/Å]	RMSE Stress [meV/Å^3]
MACE (E+F)	12.5	192.0	27.3
MACE (E+F+S)	12.9	195.6	24.7
CACE (E+F)	7.25	272.8	32.0
CACE (E+F+S)	7.97	305.3	32.7

better even within this restricted thermodynamic domain, yielding lower errors in energy, forces, and stress. This result suggests that the broader training set improves the overall robustness and transferability of the model without sacrificing local accuracy.

TABLE S3. RMSEs evaluated on configurations from 400–600 GPa and 3000–6000 K.

Model	E [meV/atom]	F [meV/Å]	Stress [meV/Å^3]
MACE (whole set)	7.8	214.4	17.1
MACE (400–600 GPa and 3000–6000 K)	8.0	233.0	22.7

All the training scripts and MLPs constructed here are provided in the GitHub repository (https://github.com/ChengUCB/HighPressure_HHe).

C. Validation

Table S1 summarizes the root mean square errors (RMSE) in energies and forces for the three MLP architectures for three functionals (vdW-DF, PBE, and HSE). These results reveal a clear performance difference between MLP architectures: CACE is superior for energy predictions, with training/testing errors of 8.30/7.25 meV/atom for vdW-DF and of 8.09/7.12 meV/atom for PBE, whereas MACE excels in force prediction, achieving the lowest force RMSEs of 187.30/192.00 meV/Å for vdW-DF and of 174.70/179.90 meV/Å for PBE. In contrast, N2P2 exhibits higher errors in both energy and force predictions.

The Δ-learning vdW-DF MLP (Δ(PBE$\rightarrow$vdW-DF)) yielded training/testing errors of 1.16/1.18 meV/atom for energies and 39.77/40.54 meV/Å for forces. Moreover, the Δ-learning HSE MLP (Δ(vdW-DF$\rightarrow$HSE)) gave training/testing errors of 10.89/13.22 meV/atom for energies and 246.91/248.26 meV/Å for forces.

III. BENCHMARKS OF THE MLPS

We extensively benchmarked the fitted MLPs by comparing with AIMD results [8], including EOSs and radial distribution functions (RDFs). The benchmarks covered pure liquid hydrogen, pure liquid helium, and seven liquid H/He mixtures (x_{He} = 0.125, 0.25, 0.375, 0.5, 0.625, 0.75, and 0.875) across P–T conditions of 100–1000 GPa and 2000–10000 K. To ensure a direct comparison, the MLP MD simulations were set to match the DFT MD protocols exactly, employing the same initial configurations, thermodynamic conditions, time steps, and simulation durations. All MD runs for N2P2-based MLPs were performed in LAMMPS [15] on CPUs utilizing the Nosé–Hoover thermostat. For MLPs constructed with MACE and CACE, MD simulations were conducted in the ASE package [16] with the same thermostat. This section presents the benchmark results for the vdW-DF MLPs with the complete set of benchmarks provided in the GitHub repository (https://github.com/ChengUCB/HighPressure_HHe).

A. NVT simulations based on vdW-DF

1. *Pure H*

Fig. S3 compares the hydrogen–hydrogen RDFs (gHH) computed using four vdW-DF MLPs trained with different approaches against benchmark AIMD results. The reference AIMD data [8] was obtained using a 64-atom liquid hydrogen system at temperatures of 2000–10000 K and densities (ρ) of 0.60–2.14 g/cm^3, which corresponds to approximately 100–1100 GPa.

One can see that the first peak of gHH decreases with increasing pressure and temperature. This feature signals the atomic–molecular transition of hydrogen at elevated P–T, as discussed later in Section V.

2. *Pure He*

Fig. S4 shows the helium–helium RDFs (gHeHe) in pure helium using four vdW-DF MLPs. The liquid helium dataset was created from AIMD simulations of a 32-atom helium system at T of 2000–10000 K and ρ of 1.42–4.25 g/cm^3, corresponding to P of 100–1000 GPa.

3. *H/He mixture*

We performed NVT MD simulations for H/He mixtures across 7 compositions (x_{He} = 0.125, 0.25, 0.375, 0.5, 0.625, 0.75, and 0.875) using four vdW-DF MLPs. The system consists of at least 64 electrons (32 to 64 atoms) and the pressure ranged from 100 GPa to 1000 GPa, in line with previous AIMD studies [8]. As a representative example, Figs. S5, S6, and S7 show good agreement between AIMD and MLP MD results of the gHH, gHeHe, and H–He RDFs (gHHe) for a 48-atom system at $x_{\mathrm{He}} = 0.5$, respectively. Furthermore, Fig. S8 demonstrates that the MLP-derived EOSs (P–ρ relationships) align closely with AIMD data across all compositions at five temperatures (T = 2000, 3000, 4000, 6000, and 10000 K). The consistent accuracy for both RDFs and EOSs confirms that all four MLPs are suitable for MD simulations.

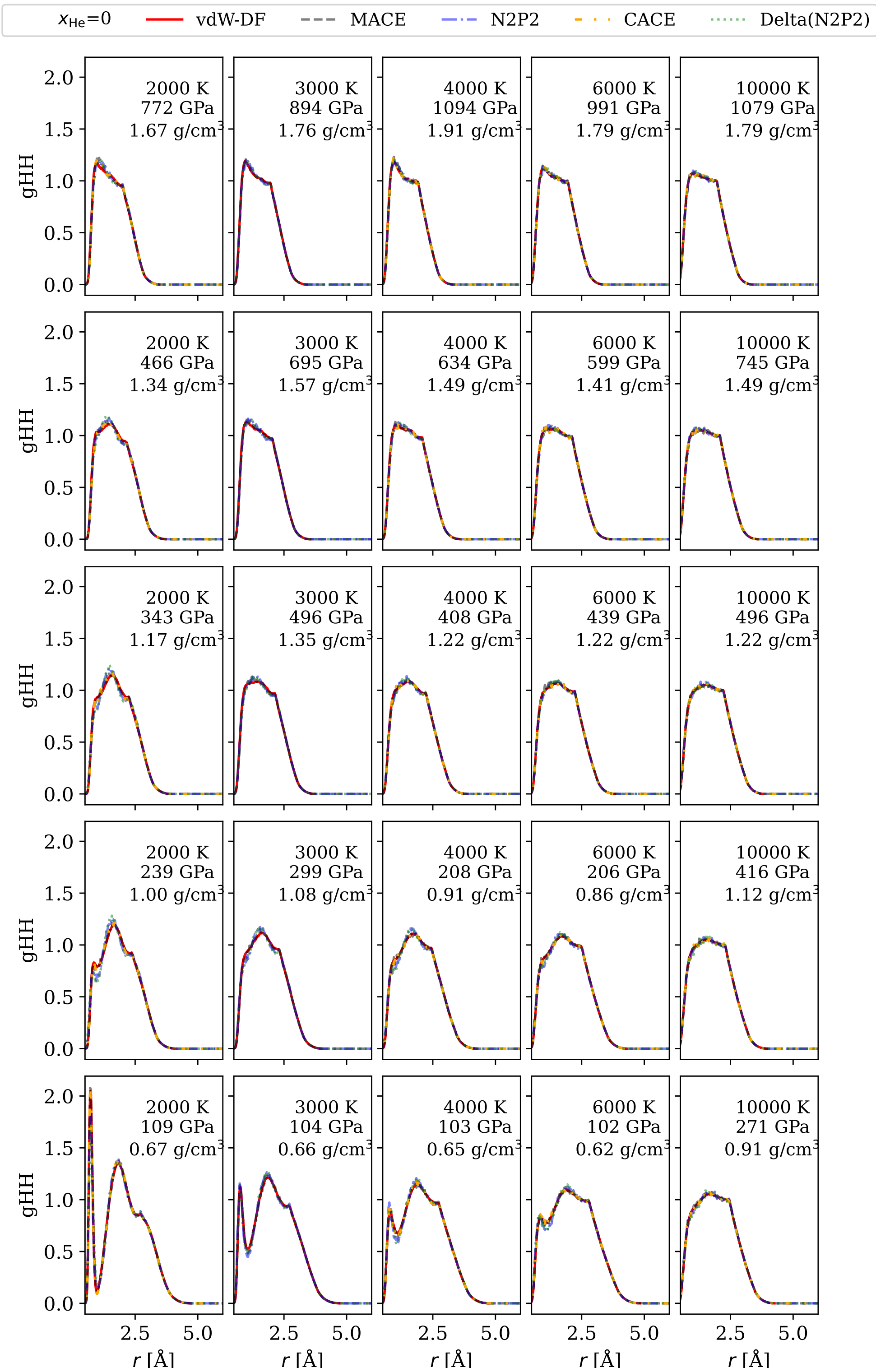

FIG. S3. hydrogen–hydrogen radial distribution functions (gHH) of pure hydrogen computed from NVT MD simulations with a system of 64 hydrogen atoms using four vdW-DF MLPs. Solid red lines denote reference vdW-DF AIMD results [8].

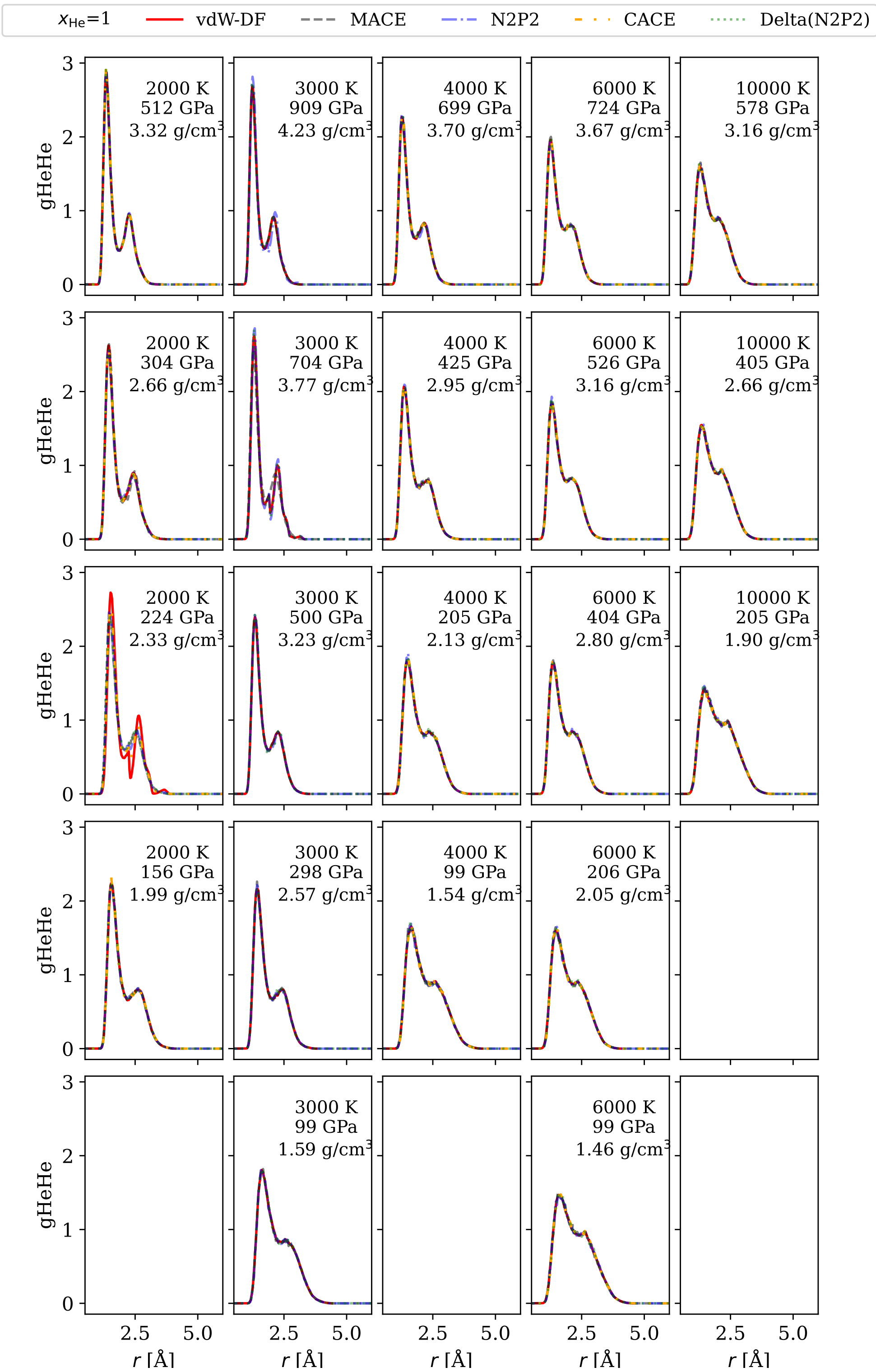

FIG. S4. helium–helium radial distribution functions (gHeHe) of pure helium computed from NVT MD simulations with a system of 32 helium atoms using four vdW-DF MLPs. Solid red lines denote reference vdW-DF AIMD results [8].

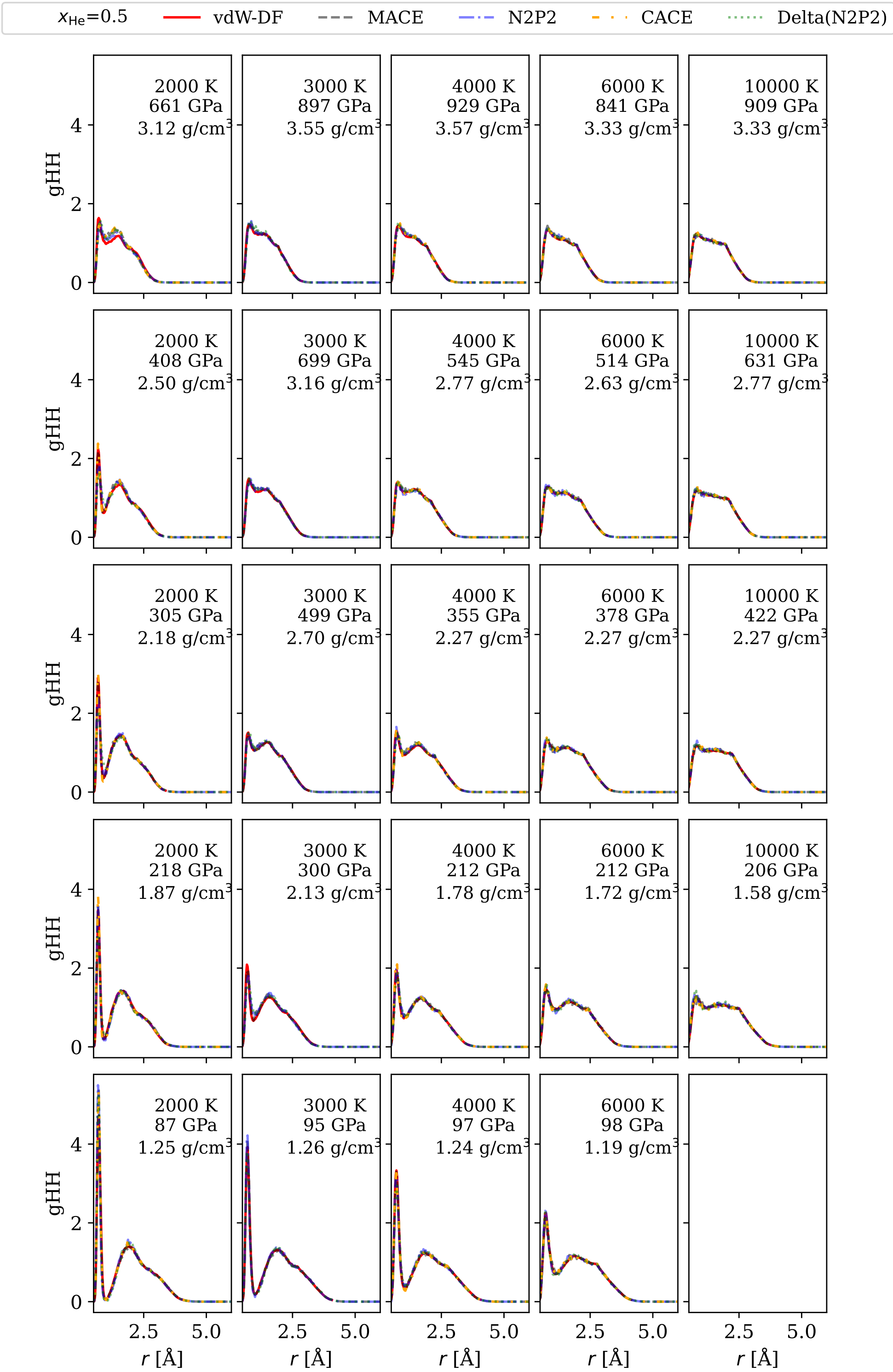

FIG. S5. hydrogen–hydrogen radial distribution functions (gHH) for a H/He mixture of $x_{\mathrm{He}} = 0.5$ computed from NVT MD simulations with a system of 24 hydrogen and 24 helium atoms using four vdW-DF MLPs. Solid red lines denote reference vdW-DF AIMD results [8].

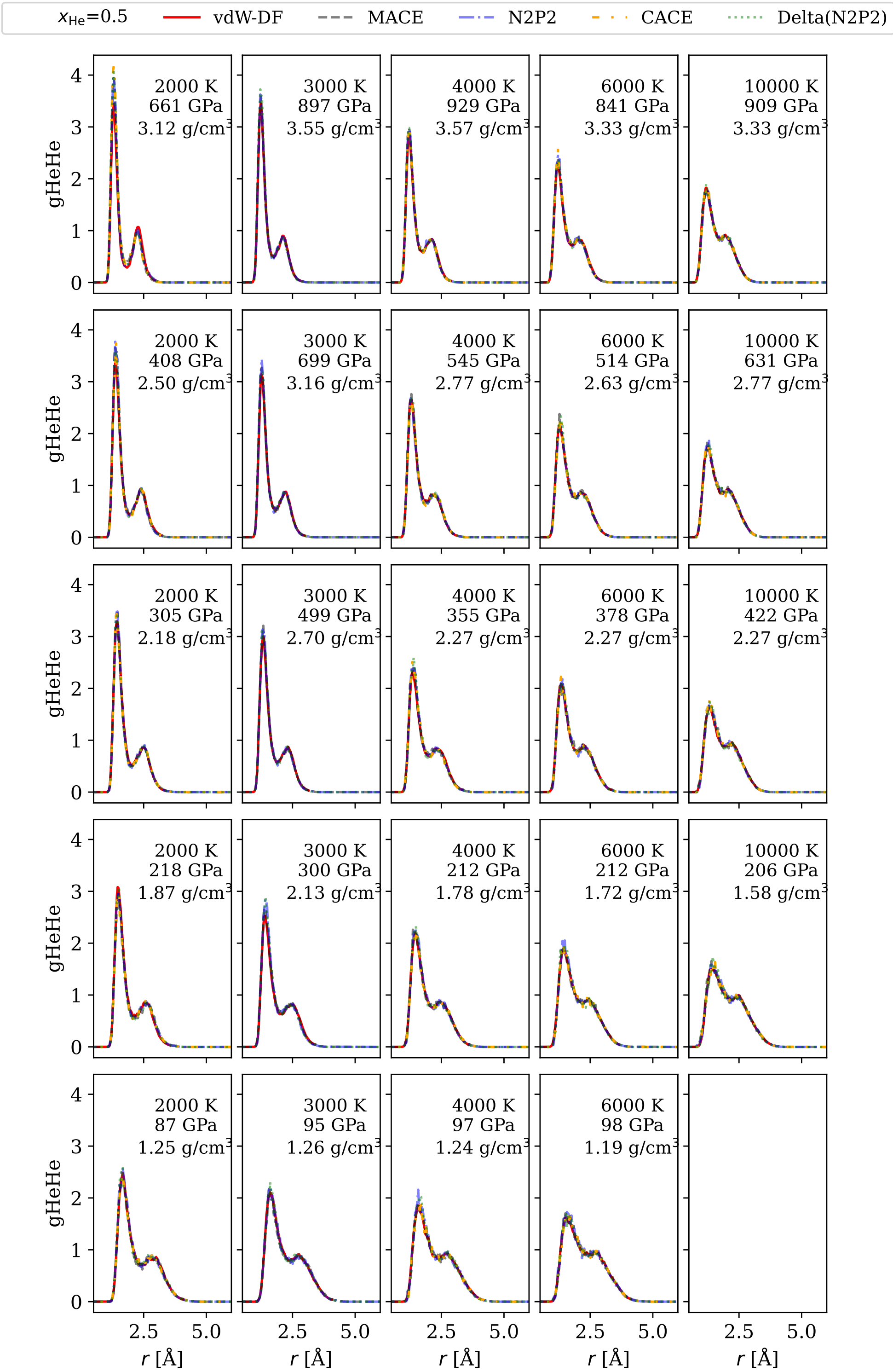

FIG. S6. helium–helium radial distribution functions (gHeHe) for a H/He mixture of $x_{\rm He} = 0.5$ computed from NVT MD simulations with a system of 24 hydrogen and 24 helium atoms using four vdW-DF MLPs. Solid red lines denote reference vdW-DF AIMD results [8].

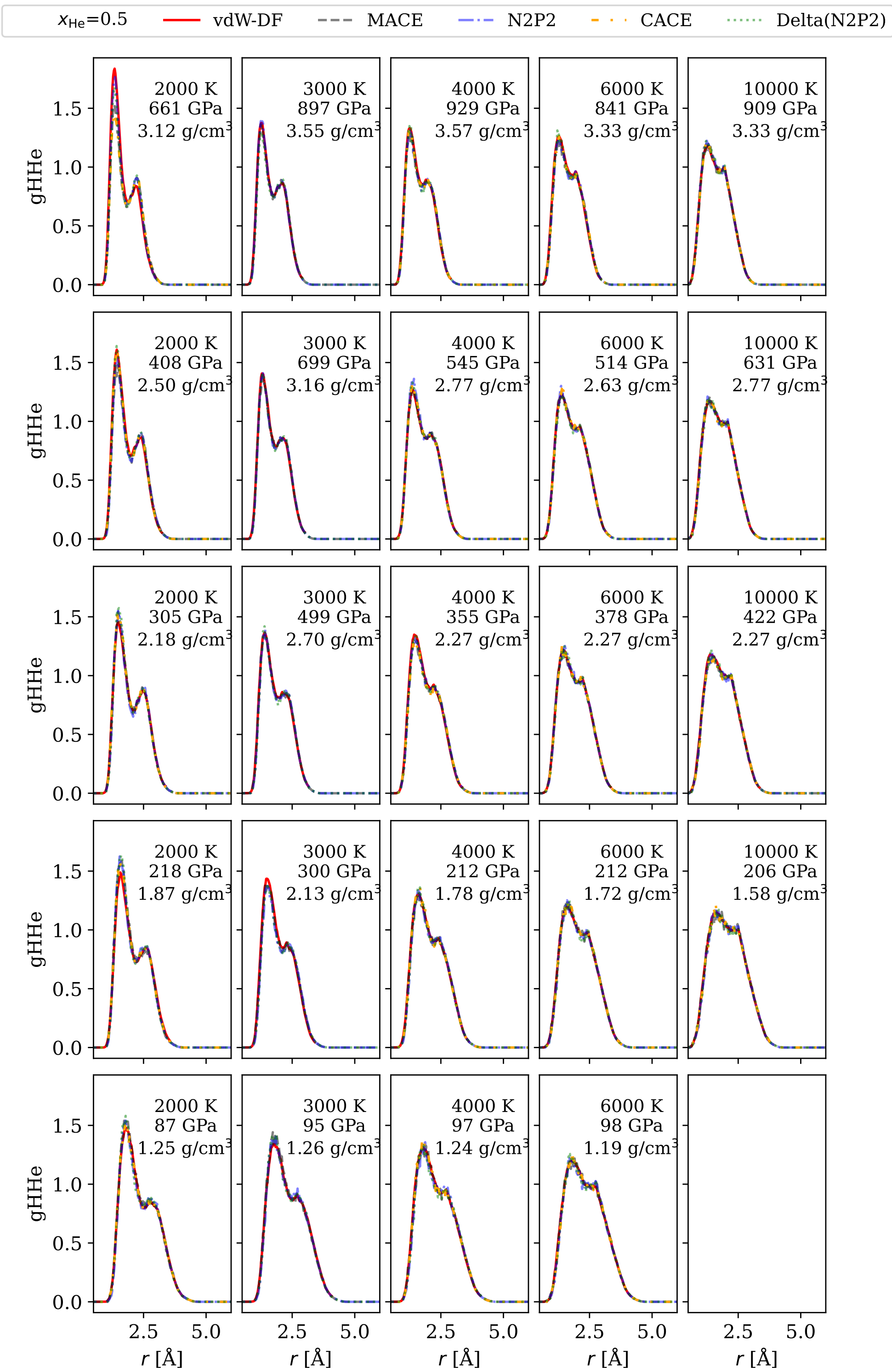

FIG. S7. Hydrogen–helium radial distribution functions (gHHe) for a H/He mixture of $x_{\mathrm{He}} = 0.5$ computed from NVT MD simulations with a system of 24 hydrogen and 24 helium atoms using four vdW-DF MLPs. Solid red lines denote reference vdW-DF AIMD results [8].

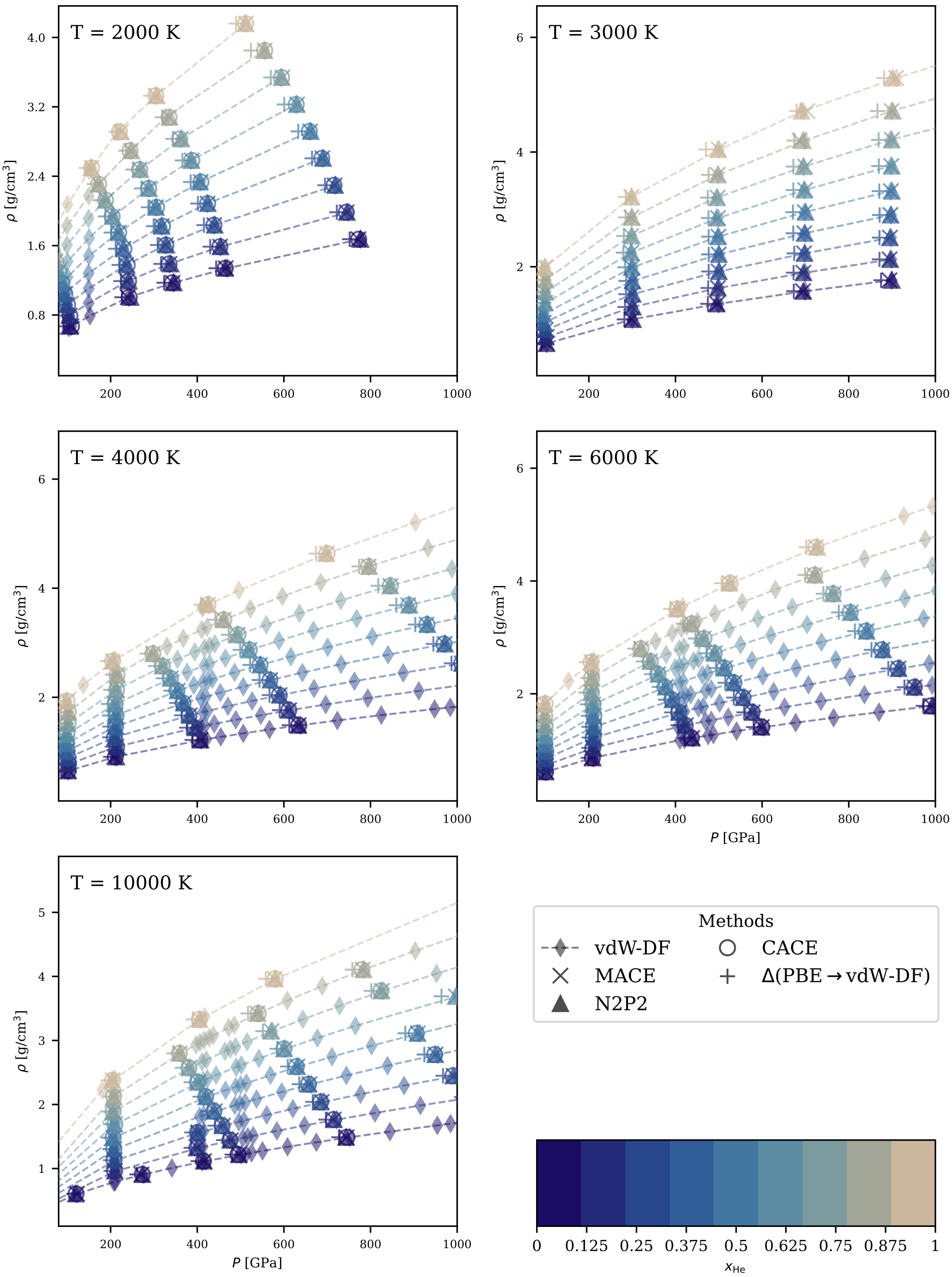

FIG. S8. Density (ρ) as a function of pressure (P) for seven H/He mixtures (x_{He} = 0.125, 0.25, 0.375, 0.5, 0.625, 0.75, and 0.875) at five temperatures (T= 2000, 3000, 4000, 6000, and 10000 K), predicted by vdW-DF DFT (dashed lines) and four vdW-DF MLPs (markers). The results are from NVT MD simulations of a system with 64 electrons (32–64 atoms).

IV. SIMULATIONS USING THE MLP

A. Sensitivity analysis regarding system size

To further demonstrate the finite-size effect for H/He demixing, we have performed a systematic size-scaling analysis using the vdW-DF N2P2 MLP. More specifically, we calculated RDFs (Fig. S9), structure factors ($S(k)$) (Fig. S10), chemical potentials (μ) and mixing Gibbs free energy (ΔG^{mix}) (Fig. S12) for system sizes ranging from the number of atoms $N = 64$ to $N = 3456$. The analysis was carried out under four thermodynamic conditions relevant to gas giants: 150 GPa and 2000 K (molecular hydrogen), 150 GPa and 3000 K (atomic hydrogen), 150 GPa and 5000 K (atomic hydrogen), and 800 GPa and 7000 K (atomic hydrogen).

From a structural perspective, Fig. S9 shows that the RDFs obtained for $N = 64$ and $N = 192$ are not fully consistent, indicating that the assumption of a completely mixed and homogeneous system is not strictly valid in small-scale simulations. For instance, in a 40 ps MD run for $x_{\mathrm{He}} = 0.30$ at 150 GPa and 2000 K with a system size of 64 atoms, we observed phase separation even in a small simulation cell of $N = 64$, as shown in Fig. S11. Moreover, once phase separation sets in, larger system sizes are needed to properly capture long-wavelength concentration fluctuations. For example, in an H/He mixture with $x_{\mathrm{He}} = 0.1$ at 150 GPa and 2000 K (the first column in Fig. S9), H/He demixing is reflected in $g_{\mathrm{HHe}} < 1$ and $g_{\mathrm{HeHe}} > 1$ at larger distances.

Fig. S10 shows the structure factor $S(k)$ and also the extrapolation to the $S(0)$ values using the Ornstein–Zernike (OZ) fits. The fitted $S(0)$ values show finite size effects: they are not converged at small system sizes (≤ 1024 atoms), and only become converged when the system size reaches 1728 atoms and above.

Moreover, using the S0 method based on the $S(0)$ values to determine the free energies of mixing, we find that the calculated ΔG^{mix} curves are also prone to finite size effects: they require larger system sizes ($N \geq 1728$) to achieve convergence; see Fig. S12. Notably, simulations with $N \leq 512$ (dark blue lines) may fail to capture phase separation as the computed ΔG^{mix} remains artificially convex due to suppressed composition fluctuations.

These results highlight why MLPs are essential in the present work: they enable the large-scale MD simulations needed to investigate H/He demixing under well-converged conditions.

Moreover, we confirm that the mixing free energies and the resulting immiscibility boundary reported in the main text, which are computed based on $N = 3456$ simulations, are fully converged with respect to system size.

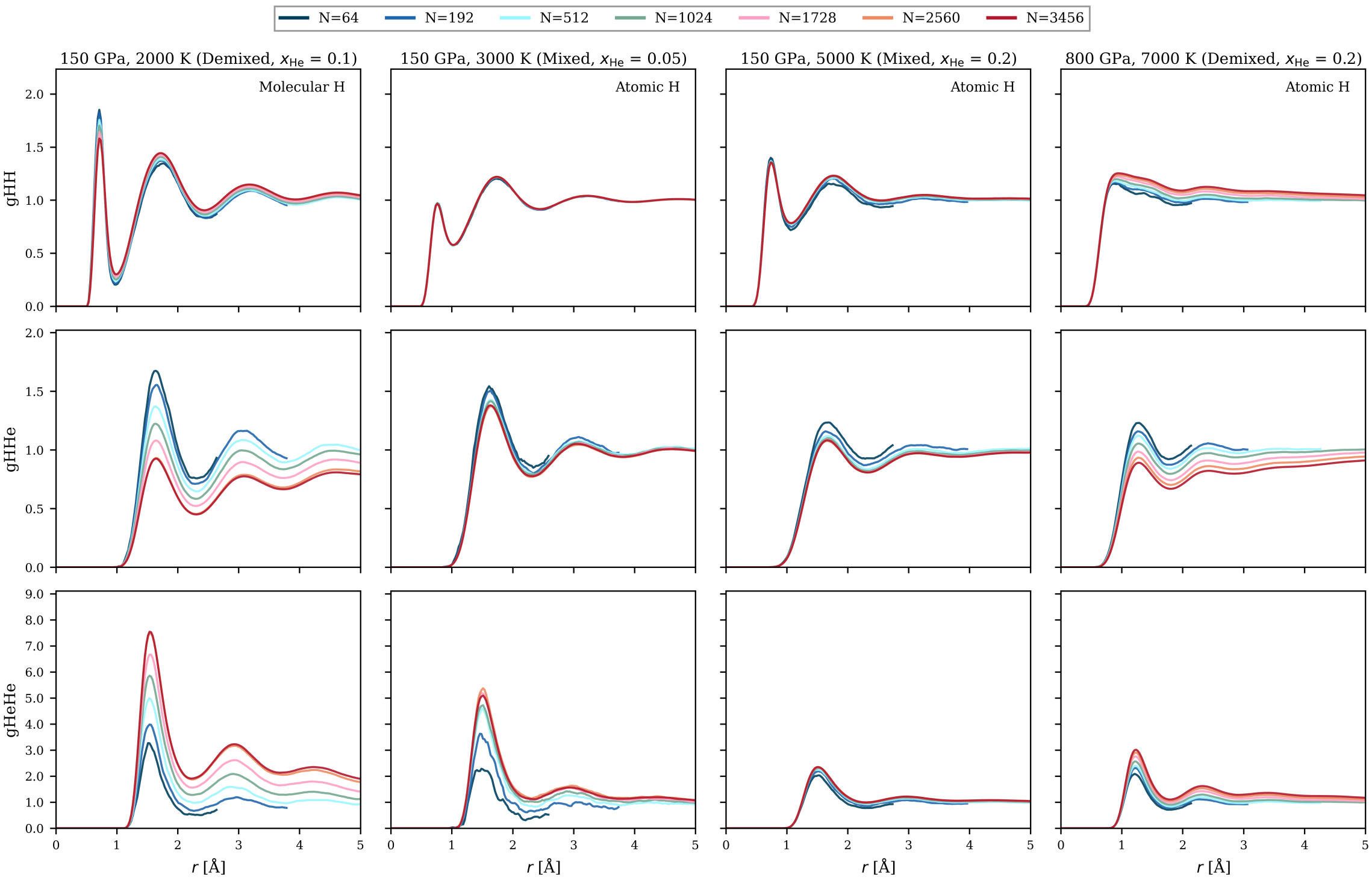

FIG. S9. System-size dependence of the partial radial distribution functions (H–H, H–He, and He–He) from $N = 64$ to $N = 3456$. Each column corresponds to a distinct P–T–x_{He} condition.

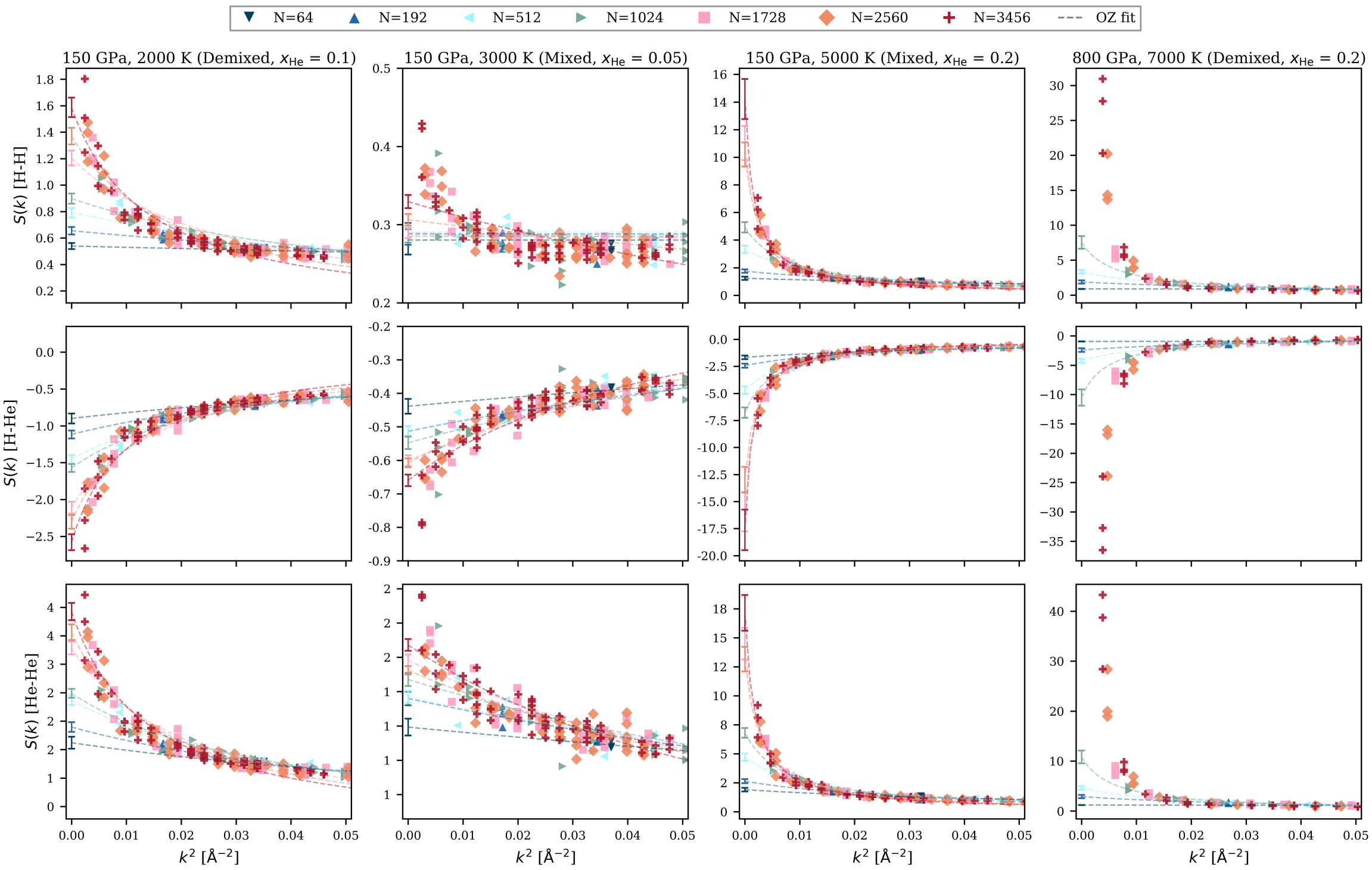

FIG. S10. System-size dependence of the structure factors $S(k)$ for H–H, H–He, and He–He pairs from $N = 64$ to $N = 3456$. Each column corresponds to a distinct P–T–x_{He} condition. Dashed lines indicate Ornstein–Zernike (OZ) fits, while error bars at $k^2 = 0$ denote the extrapolated S^0 limits.

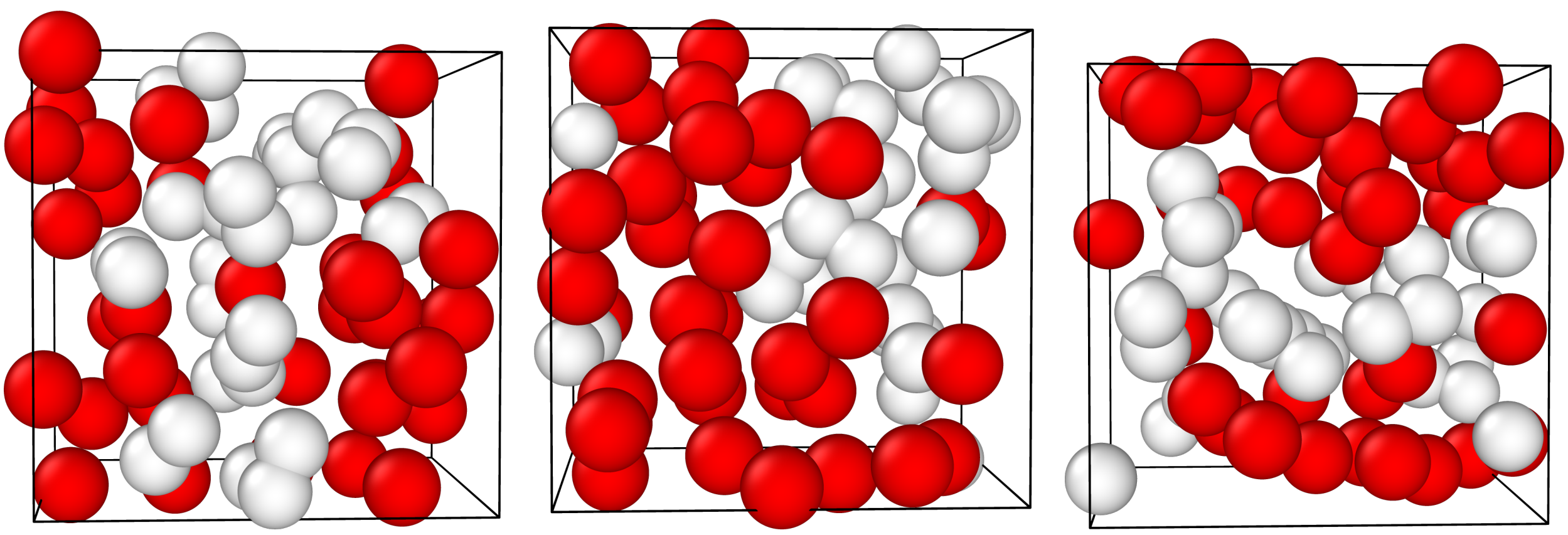

FIG. S11. Snapshots from MD simulations at 150 GPa and 2000 K for $x_{\mathrm{He}} = 0.30$ with a system size of 64 atoms. Hydrogen and helium atoms are shown as white and red particles, respectively.

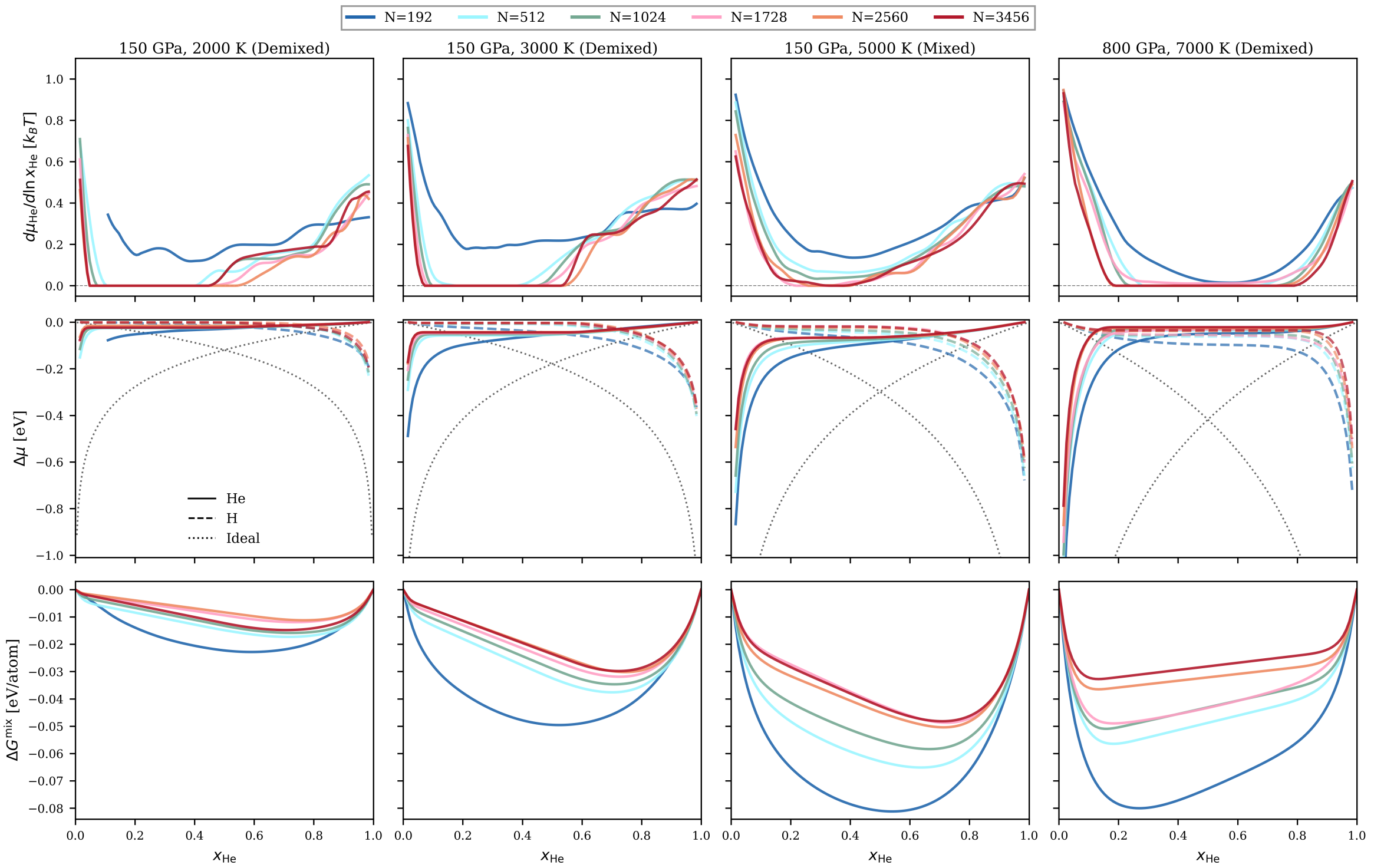

FIG. S12. System-size dependence of three thermodynamic properties from $N = 192$ to 3456: the derivative of the helium chemical potential $(\partial\mu_{\rm He}/\partial\ln x_{\rm He})_{T,P}$, chemical potentials $(\Delta\mu)$ of He (solid lines) and H (dashed lines), and per-atom mixing free energy $(\Delta G^{\rm mix})$. Each column corresponds to a distinct P–T condition. Dotted gray lines indicate ideal solution behaviors.

B. MLP MD simulations

All MD simulations for MLPs trained based on N2P2 [10] were performed in LAMMPS [15] on CPUs. After a 10 ps equilibration for a 3456-atom system, each production run was performed for 60 ps with a timestep of 0.2 fs in the isothermal–isobaric (NPT) ensemble using the Nosé–Hoover thermostat and barostat. For MLPs constructed with MACE and CACE, MD simulations were conducted with the ASE package [16] utilizing the Nosé–Hoover thermostat and Berendsen barostat.

To validate these MD settings, we conducted a sensitivity analysis on high-pressure H/He mixtures at 200 GPa and four temperatures (4000, 6000, 8000, and 10000 K), using the vdW-DF N2P2 MLP. In particular, we assessed the impact of simulation timestep (0.1–0.2 fs) and duration (40–80 ps) on computed chemical potentials of hydrogen (μ_{H}) and helium (μ_{He}). As shown in Fig. S13, the differences in the derived chemical potentials are quantitatively negligible across three tested setups: 1) a 40-ps run with a timestep of 0.1 fs (circles), 2) a 40-ps run with a timestep of 0.2 fs (crosses), and 3) an 80-ps run with a timestep of 0.2 fs (triangles). Therefore, the MD simulation settings used in the main text (a 60 ps simulation duration with a timestep of 0.2 fs) have been validated.

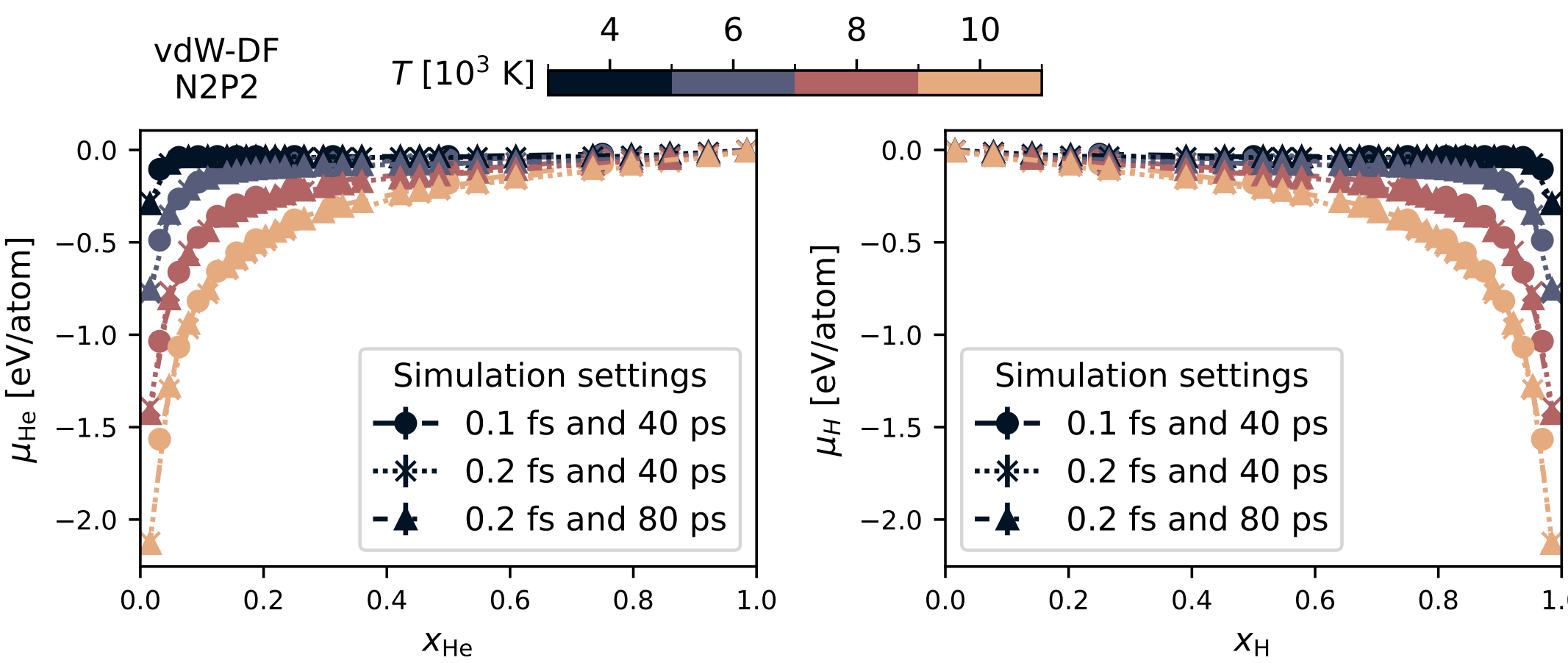

FIG. S13. Chemical potentials of hydrogen (μ_{H}, left) and helium (μ_{He}, right) in H/He mixtures computed with three different simulation settings at four temperatures (T= 4000, 6000, 8000, and 10000 K). All simulations were conducted using a vdW-DF MLP trained with N2P2.

C. Promoting MLP-derived ΔG^{mix} to the DFT level

To quantify the effect of MLP errors, we promoted the MLP-derived ΔG^{mix} to the DFT level using free-energy perturbation (FEP) for the large system with $N = 1024$ based on vdW-DF DFT calculations at three P–T conditions (150 GPa, 2000 K; 150 GPa, 5000 K; 800 GPa, 7000 K).

First, we evaluated both vdW-DF DFT and N2P2 MLP energies for a dataset of 7000 snapshots. For each system size ($N = 64$ and $N = 1024$), these configurations cover 12 helium compositions (x_{He}=0.016, 0.047, 0.11, 0.20, 0.30, 0.39, 0.55, 0.61, 0.73, 0.80, 0.92, and 0.98) at 150 GPa and 2000 K, as well as at 150 GPa and 5000 K, and 11 compositions (x_{He}=0.016–0.92) at 800 GPa and 7000 K. As shown in Fig. S14, the DFT and MLP energies are in close agreement for both system sizes across the investigated P–T–x_{He} space, with $R^2 = 0.9999$. Importantly, the large system size (1024 atoms) does not deteriorate the accuracy of the MLP. Such consistency provides the basis for applying an FEP correction from the MLP to the DFT level.

For each P–T–x_{He} condition, we sampled 100 uncorrelated configurations of system size $N = 1024$ from MD simulations using the vdW-DF N2P2 MLP. We recomputed the vdW-DF DFT energies and forces for these configurations, which form the content for the $N = 1024$ vdW-DF DFT dataset described in Section I B. We then computed the energy difference, $\Delta U = U_{\mathrm{DFT}} - U_{\mathrm{MLP}}$, where U_{DFT} is the DFT energy of a given configuration, and U_{MLP} is the corresponding MLP energy evaluated for the same configuration. The free-energy difference per atom for the system at the DFT and the MLP level, Δg, can then be estimated as:

$$N\Delta g = -k_{\mathrm{B}}T \ln \left\langle \exp\left(-\frac{\Delta U}{k_{\mathrm{B}}T}\right)\right\rangle_{\mathrm{MLP}}. \tag{1}$$

Using such free energy correction terms Δg, we promoted the MLP-derived ΔG^{mix} to the DFT level for systems at fixed P–T–x_{He}.

As shown in Fig. S15, the MLP mixing free energies are in good agreement with the DFT values at all three thermodynamic conditions considered, namely 150 GPa and 2000 K, 150 GPa and 5000 K, and 800 GPa and 7000 K, which together span molecular and atomic hydrogen as well as mixing and demixing regimes. The largest absolute correction to ΔG^{mix} occurs at the lowest-temperature condition, 150 GPa and 2000 K, where the maximum deviation, observed at $x_{\mathrm{He}} = 0.55$, remains below 10 meV/atom. At the two higher temper-

atures, which are more relevant to the immiscibility boundary, the MLP and DFT values of ΔG^{mix} are essentially indistinguishable.

Even though at temperatures relevant to the immiscibility boundary the MLP is not estimated to bring significant error to the H/He immiscibility boundary, here we provide the worst-case scenario estimate for the upper bound of the error. If we assume that the residual error in ΔG^{mix} is on the order of 10 meV/atom, the corresponding effect on the curvature, $(\partial^2 \Delta G^{\mathrm{mix}}/\partial x_{\mathrm{He}}^2)_{T,P}$, would lead to a shift in the helium compositions of the demixed phases of less than 0.05. Even assuming a maximum shift of 0.05, based on the phase diagrams reported in Fig. 2(d–f) of the main text, the DFT-level correction would translate into an adjustment of less than 1000 K in the derived phase boundaries. As such, even with this most conservative estimate, corrections of this magnitude are therefore not expected to significantly alter the predicted demixing boundary.

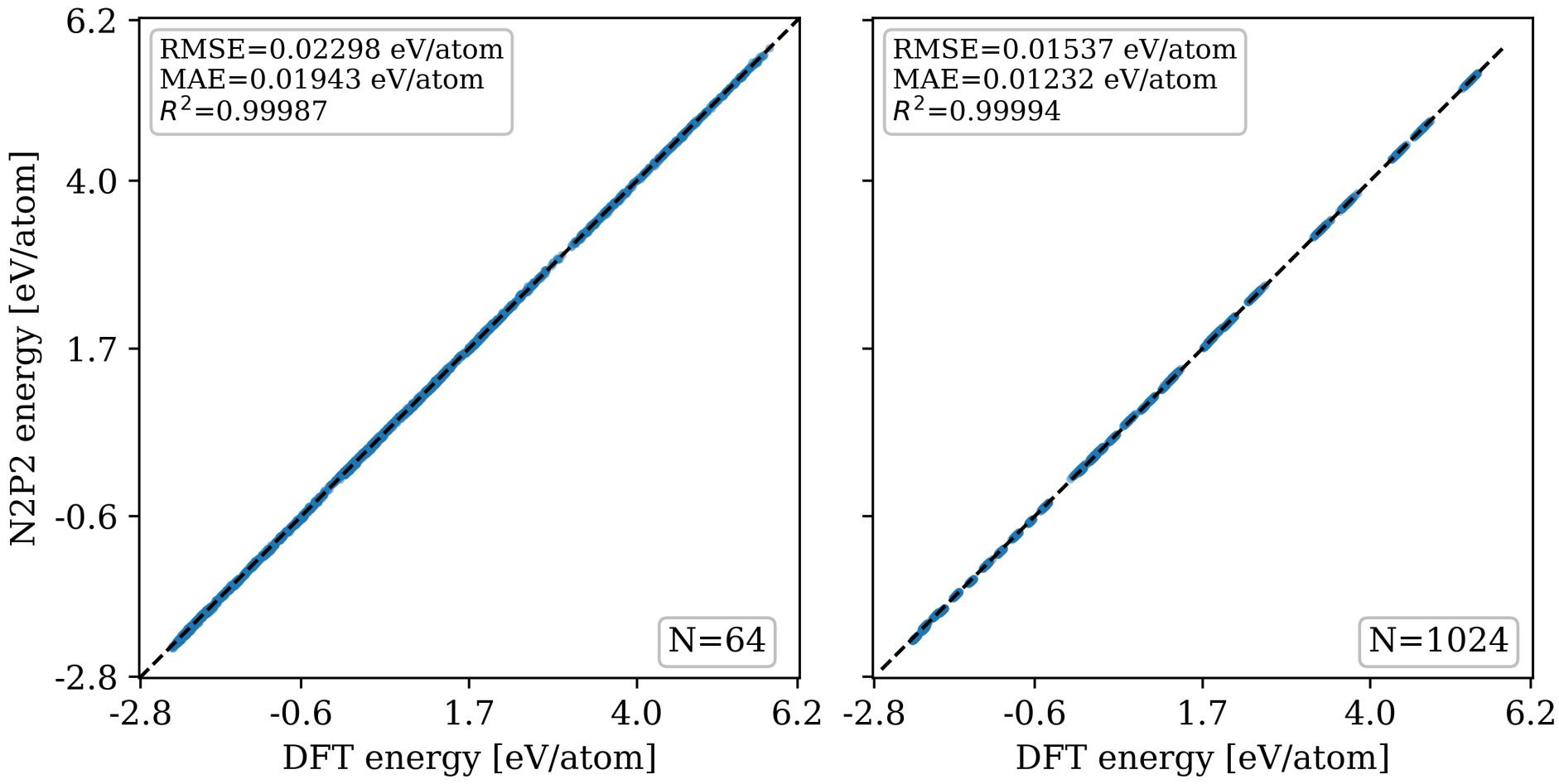

FIG. S14. Parity comparison of per-atom energies predicted by the N2P2 MLP against vdW-DF DFT reference energies for H/He mixtures at 150 GPa and 2000 K, 150 GPa and 5000 K, and 800 GPa and 7000 K. The left and right panels show the aggregated results for $N = 64$ and $N = 1024$, respectively.

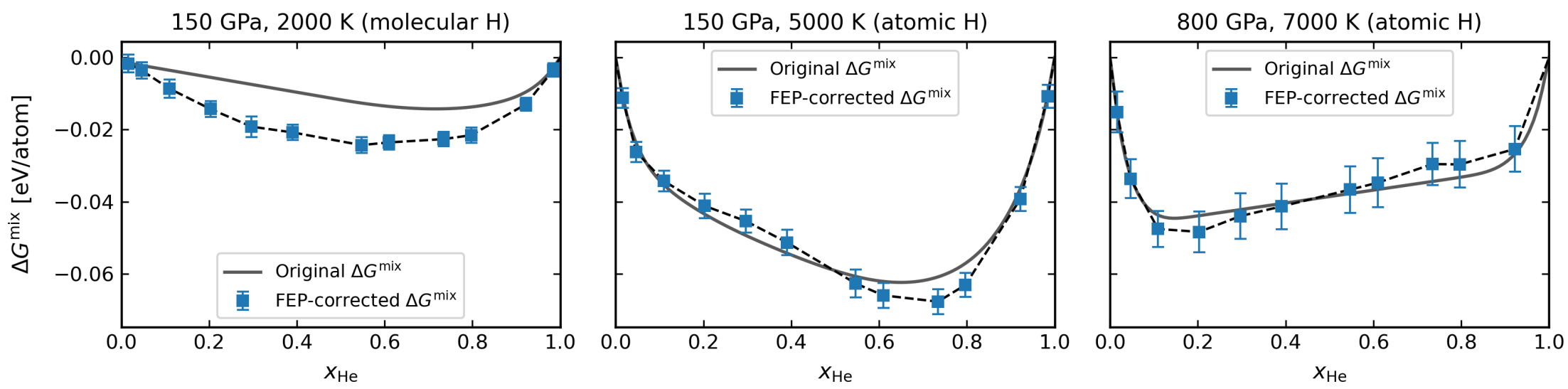

FIG. S15. Per-atom Gibbs free energy of mixing, $\Delta G^{\rm mix}$, as a function of $x_{\rm He}$ for H/He mixtures for three thermodynamic conditions based on vdW-DF: 150 GPa and 2000 K, 150 GPa and 5000 K, and 800 GPa and 7000 K. Gray solid lines show the original MLP results for the $N = 3456$ system, while blue squares denote the FEP-corrected $\Delta G^{\rm mix}$.

D. Computational performance of MLPs

While all three vdW-DF MLPs (N2P2, CACE, and MACE) provide consistent predictions for H/He phase separation as discussed in the main text (see Fig. 1), their computational performance varies substantially. For a 3456-atom H/He system on a single NVIDIA L40 GPU using ASE, the CACE model achieves 3.1 MD steps/s, twice the speed of the MACE model of 1.5 MD steps/s. In contrast, the N2P2 model reaches a faster speed on CPUs (56-core Intel Xeon) using LAMMPS, about 5.8 MD steps/s (0.1 ns/day). Similar performance trends were observed for MLPs trained on PBE and HSE. As such, we selected the more computationally efficient N2P2 MLPs for high-throughput simulations to efficiently sample the large P–T–$x_{\rm He}$ parameter space relevant to gas giants, despite their higher energy and force errors.

E. Chemical potentials of the H/He System

With the S0 method, we calculated the composition-dependent chemical potentials in H/He mixtures to determine their phase behaviors, using N2P2 MLPs trained on vdW-DF, PBE, and HSE. As discussed in the main text, we sampled a dense grid of P–T conditions, with 7 pressures set at 100, 150, 200, 400, 600, 800, 1000 GPa, 12 temperatures from 1000 K to 12000 K in 1000 K increments, and 27 compositions with $x_{\rm He}$ from 0.015 to 0.98. Demixing boundaries were then located by identifying P–T–$x_{\rm He}$ conditions where the Gibbs

free energy of mixing ΔG^{mix} satisfies $(\partial^2 \Delta G^{\mathrm{mix}}/\partial x_{\mathrm{He}}^2)_{T,P} = 0$.

The resulting chemical potentials and derived miscibility gaps for each functional are presented in this section. Figs. S16, S17, and S18 present the chemical potentials for hydrogen (red circles) and helium (blue crosses) for the vdW-DF, PBE, and HSE MLPs, respectively. The vertical dashed lines denote the solubility limit of helium in hydrogen at each P–T condition. The corresponding phase compositions ($x_{\mathrm{He-poor}}$ and $x_{\mathrm{He-rich}}$) that define the miscibility gap are listed in Tables S4, S5, and S6.

TABLE S4. H/He miscibility gap computed based on the vdW-DF N2P2 MLP.

P [GPa]	T [K]	$x_{\mathrm{He-poor}}$	$x_{\mathrm{He-rich}}$	P [GPa]	T [K]	$x_{\mathrm{He-poor}}$	$x_{\mathrm{He-rich}}$
150	1000	0.060	0.546	600	6000	0.133	0.800
150	2000	0.067	0.469	600	7000	0.189	0.746
150	3000	0.075	0.504	600	8000	0.308	0.635
150	4000	0.121	0.516	800	2000	0.035	0.803
200	2000	0.063	0.562	800	3000	0.062	0.886
200	3000	0.069	0.567	800	4000	0.072	0.864
200	4000	0.104	0.581	800	5000	0.102	0.861
200	5000	0.139	0.556	800	6000	0.137	0.807
400	2000	0.034	0.805	800	7000	0.190	0.770
400	3000	0.072	0.804	800	8000	0.256	0.742
400	4000	0.071	0.758	1000	2000	0.040	0.735
400	5000	0.107	0.754	1000	3000	0.072	0.641
400	6000	0.153	0.698	1000	4000	0.076	0.738
400	7000	0.256	0.602	1000	5000	0.106	0.808
600	2000	0.034	0.824	1000	6000	0.135	0.811
600	3000	0.067	0.865	1000	7000	0.153	0.805
600	4000	0.095	0.808	1000	8000	0.218	0.762
600	5000	0.106	0.806	1000	9000	0.375	0.623

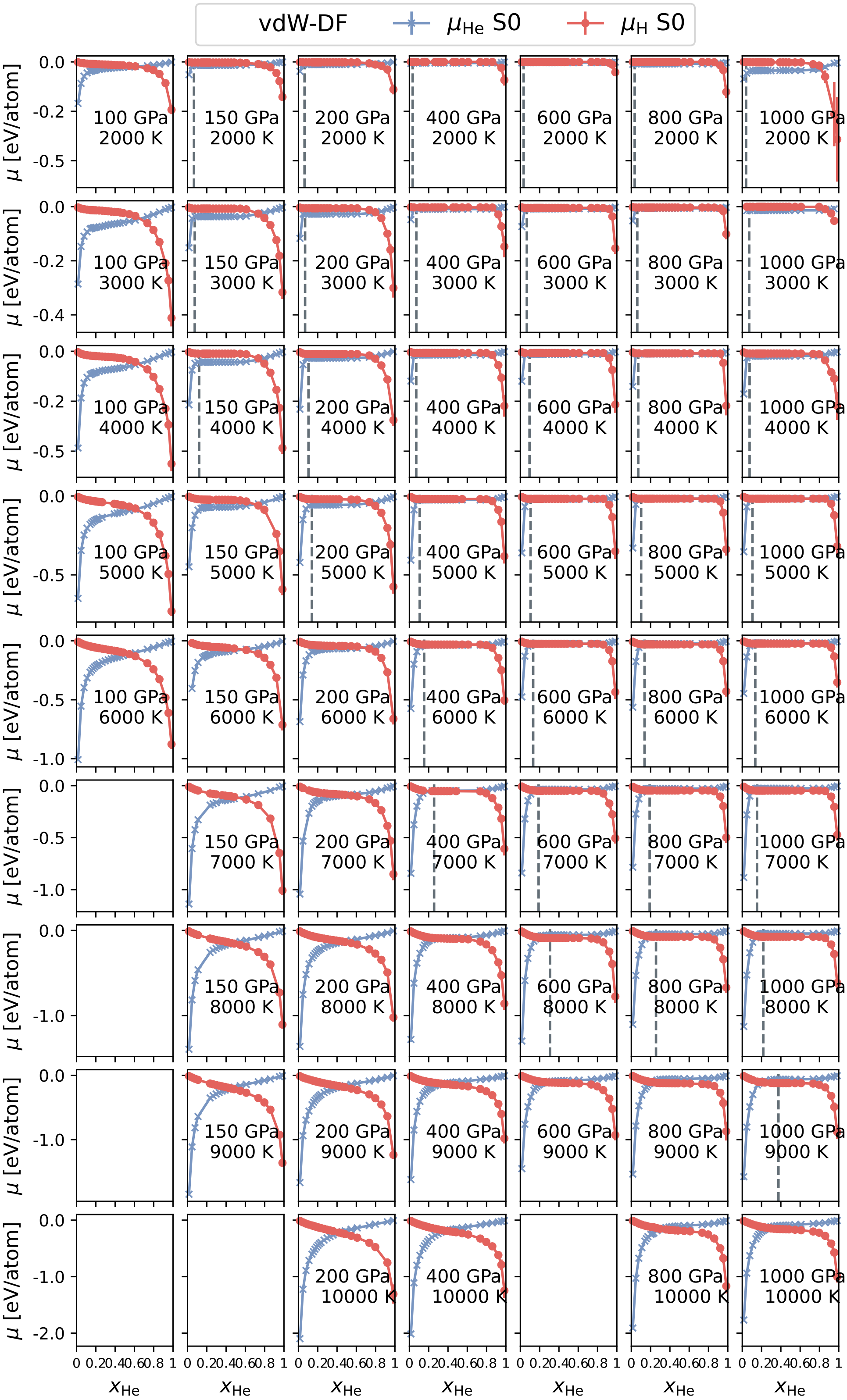

FIG. S16. The chemical potentials of hydrogen (μ_{H}, red circles) and helium (μ_{He}, blue crosses) in H/He mixtures at different temperature and pressure conditions based on the vdW-DF N2P2 MLP. Vertical dashed lines indicate the solubility limit of helium in hydrogen at each P–T condition.

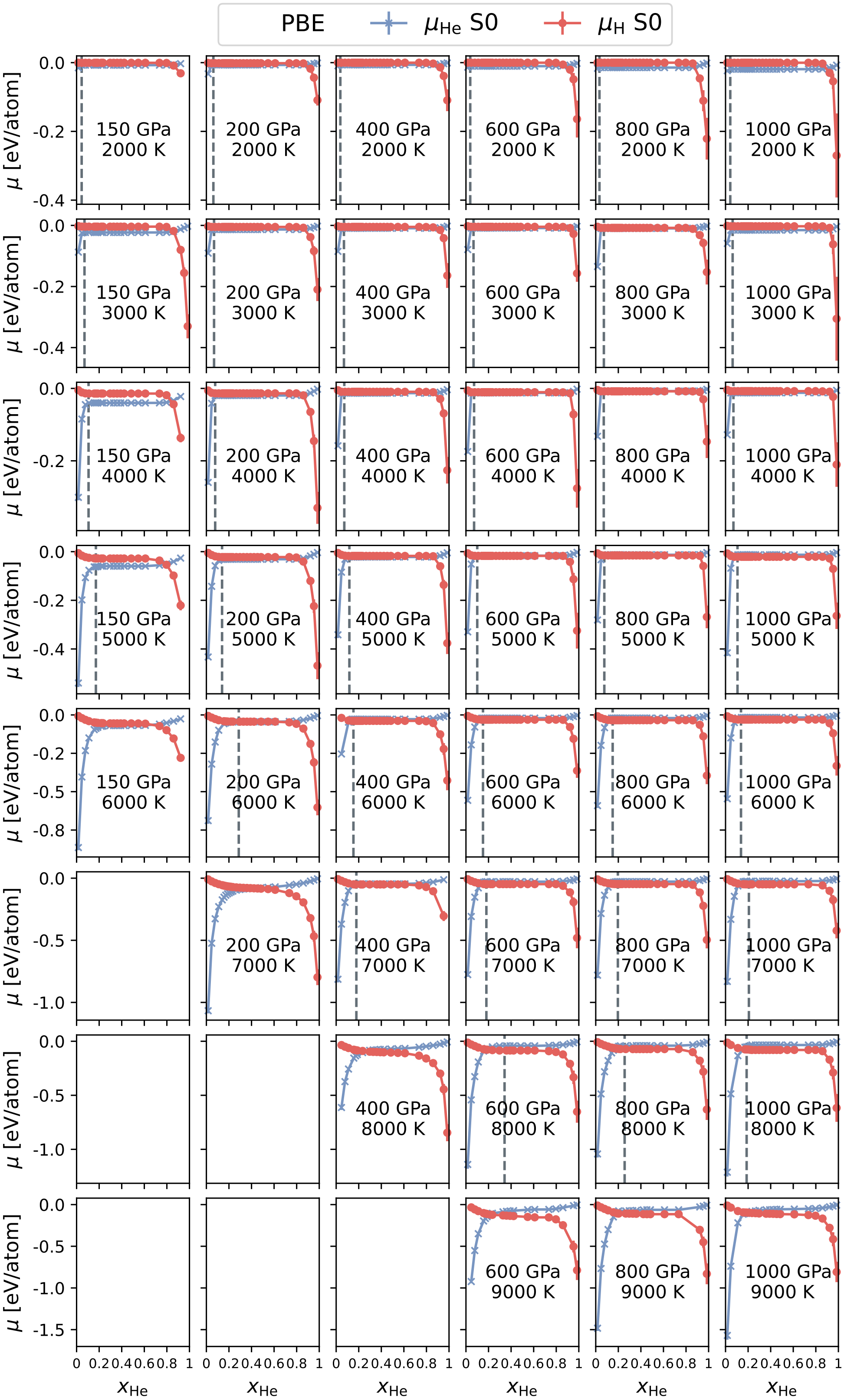

FIG. S17. The chemical potentials of hydrogen (μ_{H}, red circles) and helium (μ_{He}, blue crosses) in H/He mixtures at different temperature and pressure conditions based on the PBE N2P2 MLP. Vertical dashed lines indicate the solubility limit of helium in hydrogen at each P–T condition.

TABLE S5. H/He miscibility gap computed based on the PBE N2P2 MLP.

P [GPa]	T [K]	$x_{\mathrm{He-poor}}$	$x_{\mathrm{He-rich}}$	P [GPa]	T [K]	$x_{\mathrm{He-poor}}$	$x_{\mathrm{He-rich}}$
150	2000	0.045	0.744	600	5000	0.100	0.820
150	3000	0.070	0.746	600	6000	0.151	0.804
150	4000	0.106	0.736	600	7000	0.181	0.757
150	5000	0.171	0.583	600	8000	0.342	0.596
200	2000	0.062	0.805	800	2000	0.033	0.799
200	3000	0.068	0.802	800	3000	0.072	0.800
200	4000	0.078	0.802	800	4000	0.071	0.873
200	5000	0.139	0.750	800	5000	0.076	0.866
200	6000	0.287	0.621	800	6000	0.150	0.816
400	2000	0.037	0.789	800	7000	0.196	0.804
400	3000	0.069	0.830	800	8000	0.256	0.668
400	4000	0.072	0.812	1000	2000	0.042	0.800
400	5000	0.117	0.805	1000	3000	0.063	0.862
400	6000	0.153	0.768	1000	4000	0.070	0.924
400	7000	0.179	0.588	1000	5000	0.106	0.866
600	2000	0.038	0.748	1000	6000	0.137	0.822
600	3000	0.067	0.871	1000	7000	0.207	0.753
600	4000	0.072	0.863	1000	8000	0.187	0.800

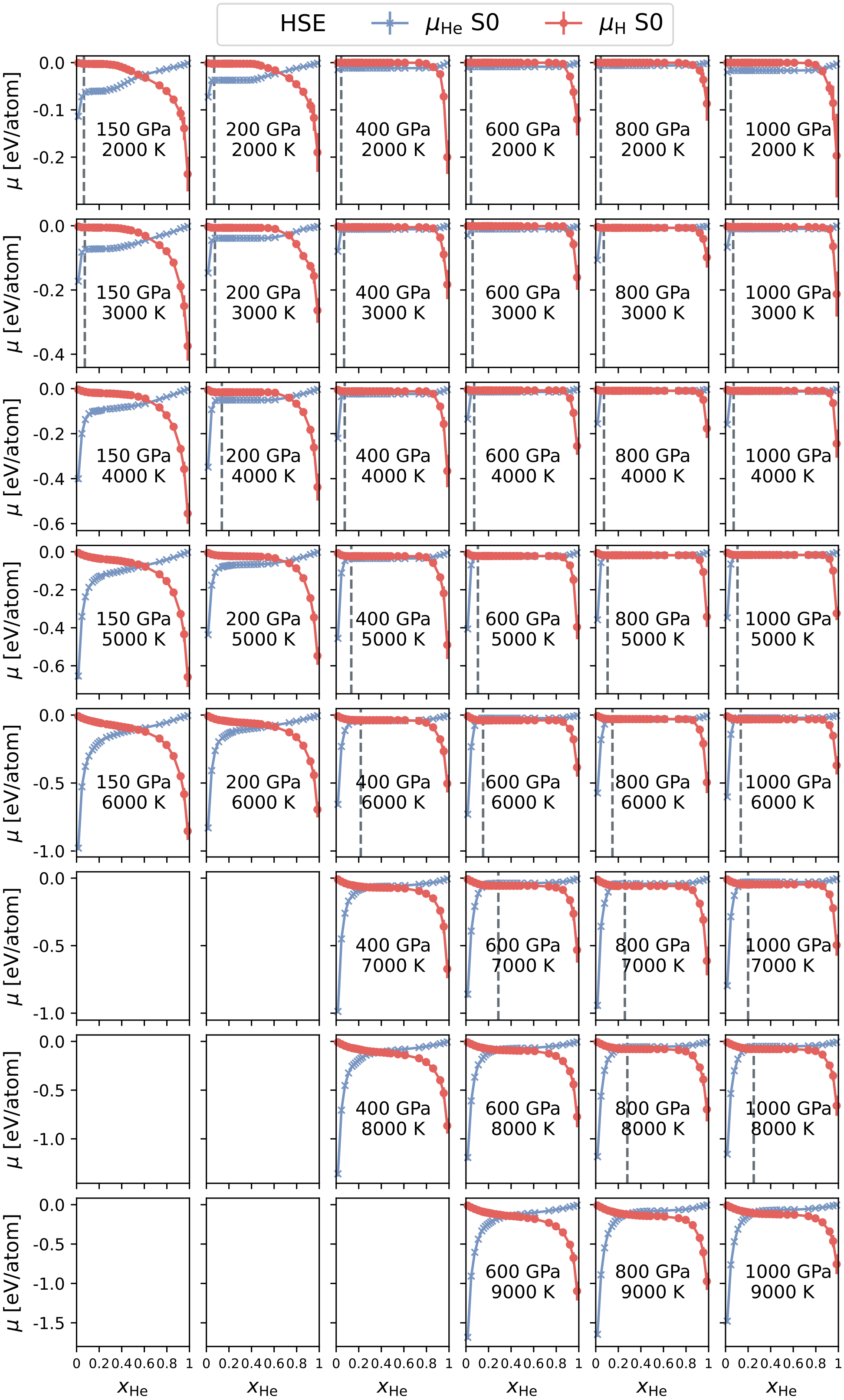

FIG. S18. The chemical potentials of hydrogen (μ_H, red circles) and helium (μ_{He}, blue crosses) in H/He mixtures at different temperature and pressure conditions based on the HSE N2P2 MLP. Vertical dashed lines indicate the solubility limit of helium in hydrogen at each P–T condition.

TABLE S6. H/He miscibility gap computed based on the HSE N2P2 MLP.

P [GPa]	T [K]	$x_{\mathrm{He-poor}}$	$x_{\mathrm{He-rich}}$	P [GPa]	T [K]	$x_{\mathrm{He-poor}}$	$x_{\mathrm{He-rich}}$
150	2000	0.109	0.224	600	7000	0.270	0.596
150	3000	0.076	0.244	600	8000	0.476	0.521
200	2000	0.064	0.376	800	2000	0.037	0.669
200	3000	0.075	0.494	800	3000	0.071	0.807
200	4000	0.105	0.551	800	4000	0.072	0.832
200	5000	0.232	0.395	800	5000	0.101	0.815
400	2000	0.039	0.684	800	6000	0.139	0.801
400	3000	0.068	0.803	800	7000	0.207	0.737
400	4000	0.076	0.756	800	8000	0.263	0.583
400	5000	0.118	0.736	1000	2000	0.041	0.645
400	6000	0.187	0.623	1000	3000	0.065	0.862
400	7000	0.379	0.459	1000	4000	0.071	0.863
600	2000	0.038	0.800	1000	5000	0.106	0.826
600	3000	0.047	0.804	1000	6000	0.128	0.807
600	4000	0.071	0.804	1000	7000	0.168	0.785
600	5000	0.105	0.798	1000	8000	0.249	0.575

V. ATOMIC–MOLECULAR PHASE TRANSITION OF HYDROGEN

To trace the atomic–molecular phase transition of hydrogen, we employed an order parameter $s(r)$ to quantify the degree of hydrogen pairing from gHH. Conceptually, $s(r)$ denotes the fraction of hydrogen atoms with one hydrogen neighbor, which is computed from gHH with a smooth cutoff function that is 1 within 0.8 Å and decays to 0 at 1.1 Å:

$$s(r) = \begin{cases} 1 & \text{if } r \leq d_0 \\ 0.5\left[\cos\left(\frac{r-d_0}{r_0}\pi\right)+1\right] & \text{if } d_0 < r \leq d_0 + r_0 \\ 0 & \text{if } r > d_0 + r_0 \end{cases} \tag{2}$$

where r is the interatomic distance, with cutoff parameters $d_0 = 0.8$ Å and $r_0 = 0.3$ Å. Accordingly, $s(r) = 1.0$ represents fully molecular hydrogen, whereas $s(r) = 0.6$ signifies a fully dissociated, atomic state of hydrogen. Physically, the decrease in $s(r)$ with increasing temperature and pressure is linked to the attenuation of the first peak of gHH (Fig. S19(a)), which signals hydrogen atomization.

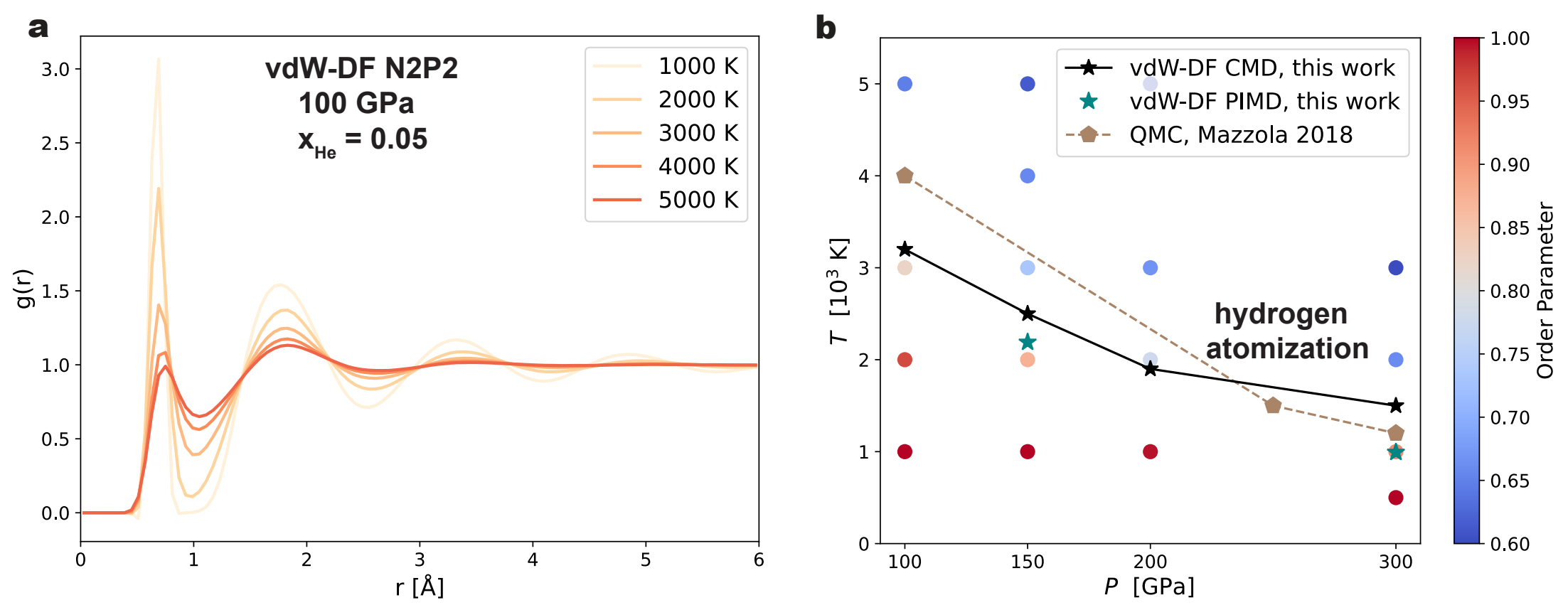

FIG. S19. The atomic–molecular transition of hydrogen in a H/He mixture of $x_{\mathrm{He}} = 0.05$. (a) Temperature dependence of the H–H radial distribution functions (gHH) at 100 GPa based on the vdW-DF N2P2 MLP. The first gHH peak diminishes with increasing temperature. (b) Order parameter $s(r)$ as a function of temperature and pressure. The black curve denotes the phase boundary of hydrogen atomization in a H/He mixture of $x_{\mathrm{He}} = 0.05$, compared with previous quantum Monte Carlo results [17] (brown dashed line) for $x_{\mathrm{He}} = 0.085$.

We determined the atomic–molecular phase transition for a H/He mixture at $x_{\mathrm{He}} = 0.05$

(Fig. S19(b)), a composition selected to preclude demixing in simulations. Consistent with previous work [13], the transition was defined as the P–T point of 50% hydrogen dissociation (i.e., $s(r) = 0.8$). At each pressure, the transition temperature was obtained by linearly interpolating the calculated $s(r)$ over temperature. The resulting transition boundary for hydrogen atomization based on vdW-DF is shown as the black curve in Fig. S19(b). This derived boundary aligns well with previous quantum Monte Carlo (QMC) MD simulations at $x_{\mathrm{He}} = 0.085$ (brown dashed line) [17].

To examine the impact of helium concentration on the molecular–atomic transition, we applied the same methodology to map the transition boundaries for pure hydrogen (green triangles in Fig. S20) and for an H/He mixture with $x_{\mathrm{He}} = 0.016$ (blue squares) and $x_{\mathrm{He}} = 0.05$ (red circles), based on three different functionals (PBE, vdW-DF, and HSE). Across all the functionals, increasing helium fractions raises the transition temperature, indicating that helium stabilizes molecular hydrogen at high pressures and thus delays hydrogen atomization, in agreement with previous QMC MD [17] and PBE AIMD [18] simulations of the nonmetal-to-metal transition in H/He mixtures [17, 19].

We further investigated the nuclear quantum effects (NQEs) on the molecular–atomic transition of hydrogen in an H/He mixture with $x_{\mathrm{He}} = 0.05$ via the ring polymer path integral molecular dynamics (PIMD) simulations, as detailed in Section VII. Based on the vdW-DF N2P2 MLP, inclusion of NQEs lowers the transition temperature of hydrogen by up to 450 K, see Table S7 and Fig. S20. As discussed in the main text, such a decrease in the atomic–molecular transition temperatures of hydrogen mostly originates from the zero-point energy (ZPE) in molecular hydrogen, which weakens the covalent bond and promotes molecular dissociation. This finding is consistent with previous DFT-based [20–24] and MLP-based studies [25] of dense hydrogen that explicitly account for NQEs.

TABLE S7. The atomic–molecular transition temperature of hydrogen T_{AM} in a H/He mixture of $x_{\mathrm{He}} = 0.05$ from MD and PIMD at 150 and 300 GPa, using the vdW-DF N2P2 MLP.

Method	T_{AM} at 150 GPa	T_{AM} at 300 GPa
MD	2520 K	1449 K
PIMD	2190 K	993 K

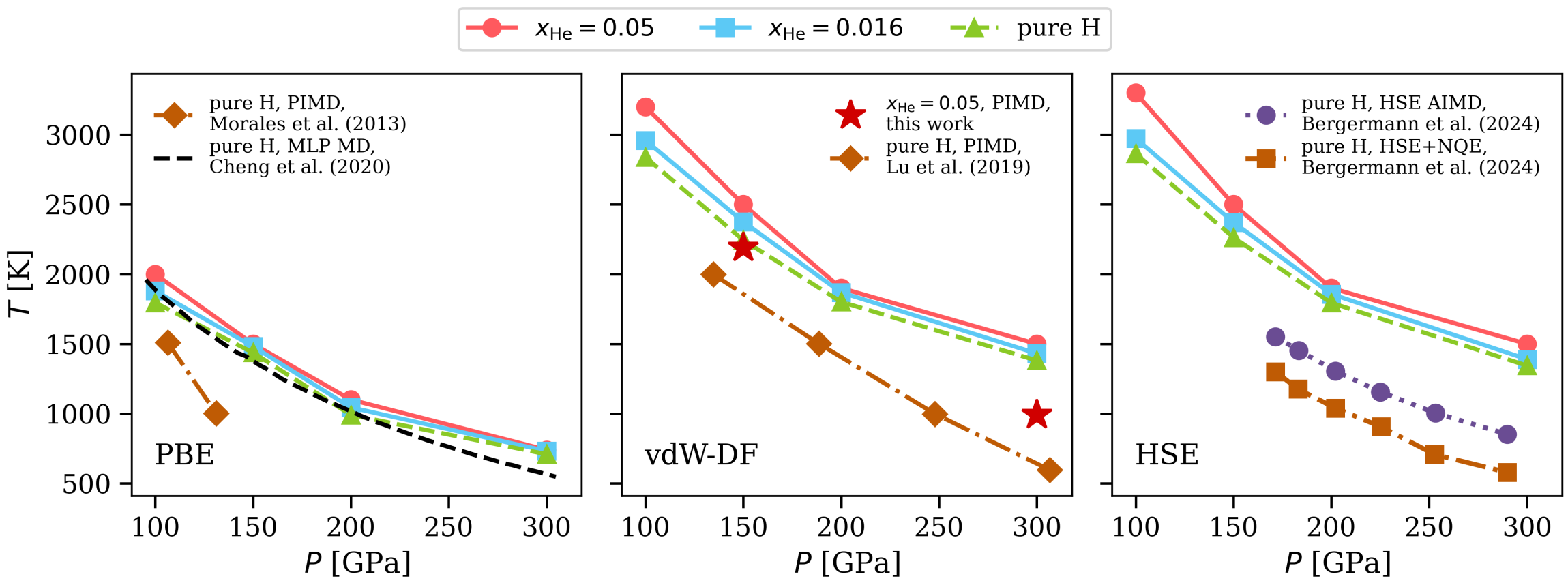

FIG. S20. Comparison of the atomic–molecular transition temperatures of hydrogen for H/He mixtures with $x_{\mathrm{He}} = 0.05$ and $x_{\mathrm{He}} = 0.016$, and for pure hydrogen, based on PBE (left), vdW-DF (middle), and HSE (right). Red stars represent the transition temperatures with $x_{\mathrm{He}} = 0.05$ from our PIMD simulations based on the vdW-DF N2P2 MLP at 150 and 300 GPa. The dashed black line denotes the transition boundary for pure hydrogen reported in the previous PBE MLP study [13]. The purple dotted line indicates the transition boundary for pure hydrogen in an earlier AIMD study [24]. Brown dot-dashed lines correspond to phase boundaries of pure hydrogen with NQEs reported by previous DFT-based studies [20, 21, 24].

VI. THERMODYNAMIC MODELS FOR H/HE MIXTURES

A. Third-order Redlich–Kister model

To rationalize the thermodynamics of H/He phase separation and understand the discrepancies with previous results, we fitted ΔG^{mix} to a third-order Redlich–Kister (R–K) solution model [26]:

$$\Delta G^{\mathrm{mix}} = k_B T(x_{\mathrm{He}} \ln x_{\mathrm{He}} + x_{\mathrm{H}} \ln x_{\mathrm{H}}) + x_{\mathrm{He}} x_{\mathrm{H}} \sum_{i=1}^{3} \omega_i \left(1 - 2x_{\mathrm{He}}\right)^{i-1}, \tag{3}$$

where the parameters ω_i account for the non-ideal interactions that drive phase separation. Consistent with the analyses in the main text, $\omega_i(T, P)$ parameters were fitted using data exclusively from single-phase regions, and the model fits are shown as solid lines in Fig. S21 for PBE and Fig. S22 for HSE. For the two-phase region, where the real free energy curve reduces to a straight line, we used dashed lines to denote the modeled ΔG^{mix} values ex-

trapolated from the individual R–K fits. At lower temperatures and higher pressures, we performed a single global fit of ΔG^{mix} to parameterize the P–T dependence of ω_i and extract their coefficients to model ΔG^{mix}, which is shown as dotted lines in Fig. S21(a) and Fig. S22(a). While $\omega_3(T, P)$ was treated as a constant at each pressure, the pressure and temperature dependence of the $\omega_1(T, P)$ and $\omega_2(T, P)$ parameters were described with the polynomial expression $\omega_i = c + a_1T + a_2T^2 + b_1P + b_2P^2$. During the fitting procedure, temperature was expressed in units of 10^3 K and pressure in units of 10^2 GPa.

We further validated the sufficiency of the third-order R–K model by fitting a fifth-order model to the vdW-DF data, as shown in Fig. S23. The model parameters ω_i were fitted to polynomial expressions of P and T using the following forms: $\omega_1 = a_0 + b_1T + b_2T^2 + c_1P + c_2P^2$, $\omega_2 = a_0 + b_1T + b_2T^2 + c_1P$, $\omega_4 = a_0 + b_1/T + c_1P$, while ω_3 and ω_5 share the linear form ($\omega = a_0 + c_1P$). The fitted $\omega_5(T, P)$ exhibits scattered, non-systematic behavior across the pressure range, whereas $\omega_4(T, P)$ is negatively correlated with temperature at $P \leq 200$ GPa but levels off to nearly constant values at higher pressures. Nevertheless, including ω_4 and ω_5 does not meaningfully improve the fit to ΔG^{mix} as shown in Fig. S23(a). Moreover, the immiscibility boundary inferred from the fifth-order R–K fits (dotted blue line in Fig. S24) shows no better agreement with direct MD results (solid black line) than the third-order model (dashed red line), indicating no benefit in predictive power from higher-order parameters. In addition, we find that the immiscibility boundaries obtained from the common-tangent construction (dashed black line in Fig. S24), as adopted in previous AIMD studies, are in good agreement with those determined from the criterion $(\partial^2 \Delta G^{\mathrm{mix}} / \partial x_{\mathrm{He}}^2)_{T,P} = 0$, with the difference remaining within 500 K. These findings demonstrate that varying the order of the R–K model or the post-analysis procedures changes the derived demixing temperature by no more than 1000 K.

To highlight the difference in the computed ΔG^{mix} between our MLP MD simulations and earlier AIMD studies [8] based on vdW-DF, Fig. S25 presents the ΔG^{mix} data and the corresponding third-order R–K fits at 400 GPa and 1000 GPa over a range of temperatures. In detail, the AIMD calculations for ΔG^{mix} at 400 GPa (left panel in Fig. S25) were derived by assuming ideal entropy of mixing, whereas ΔG^{mix} at 1000 GPa (right panel in Fig. S25) was analyzed with NQEs and non-ideal entropy. Our computed ΔG^{mix} differs from the AIMD results [8], irrespective of whether non-ideal effects in entropy and NQEs are included in the analysis of AIMD results. Such a difference eventually leads to the ∼2000 K discrepancy in

the inferred immiscibility boundary.

As a result, we found that the discrepancy in the predicted immiscibility boundaries originates from the large difference in the derived ΔG^{mix}. While it is difficult to ascertain the cause of the discrepancy without fully replicating the previous DFT work, we hypothesize that finite-size effects are the primary cause. Previous studies [8, 27] assumed that 64-atom H/He mixtures remain fully mixed and derived ΔG^{mix} on that basis. Our analysis, however, shows that simulations with $N = 64$ do not necessarily maintain a homogeneous mixed state and can therefore introduce errors into the computed ΔG^{mix}, as discussed in Section IV A.

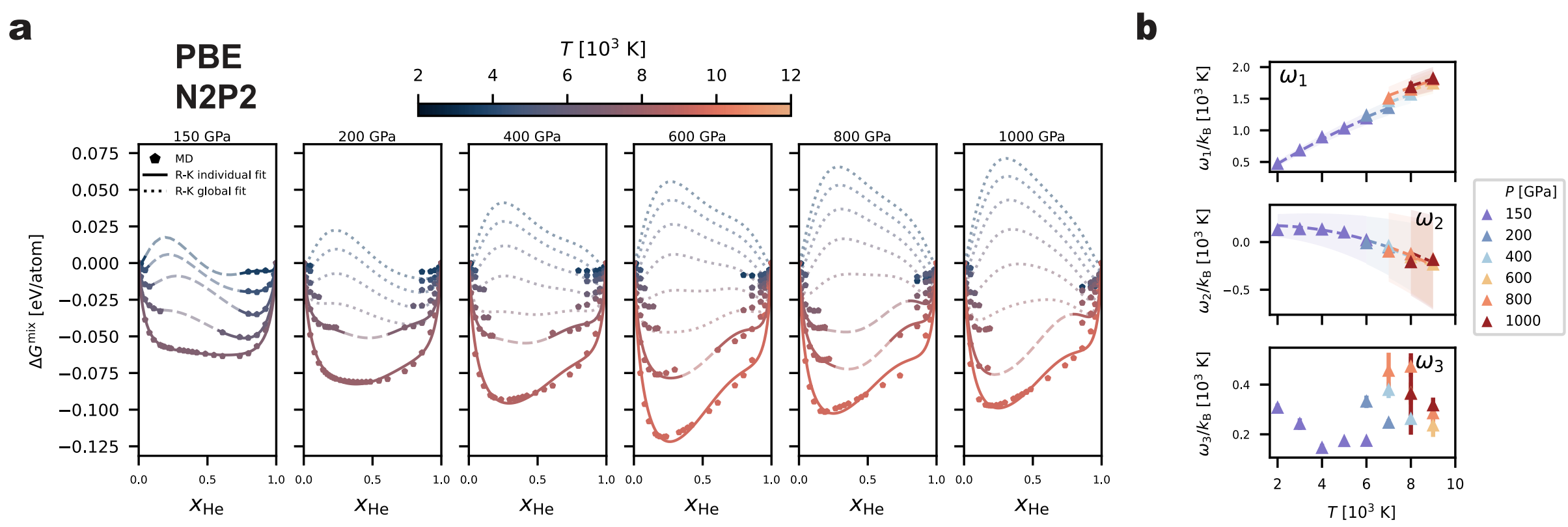

FIG. S21. The third-order Redlich–Kister (R–K) model fits of the mixing free energy (ΔG^{mix}) for H/He mixtures based on the PBE N2P2 MLP. (a) ΔG^{mix} computed from 150 to 1000 GPa and 2000 to 12000 K. The solid lines denote the fitted third-order R–K model at each P–T. The dashed lines represent the modeled ΔG^{mix} data from individual R–K fits for assumed homogeneous H/He mixtures within the demixing region. The dotted lines show ΔG^{mix} derived from a global R–K fit. (b) Fitted ω_i parameters derived from simulated ΔG^{mix}. The dashed lines, with shaded uncertainty bands, represent the polynomial fits for the pressure and temperature dependence of the $\omega_1(T, P)$ and $\omega_2(T, P)$ parameters.

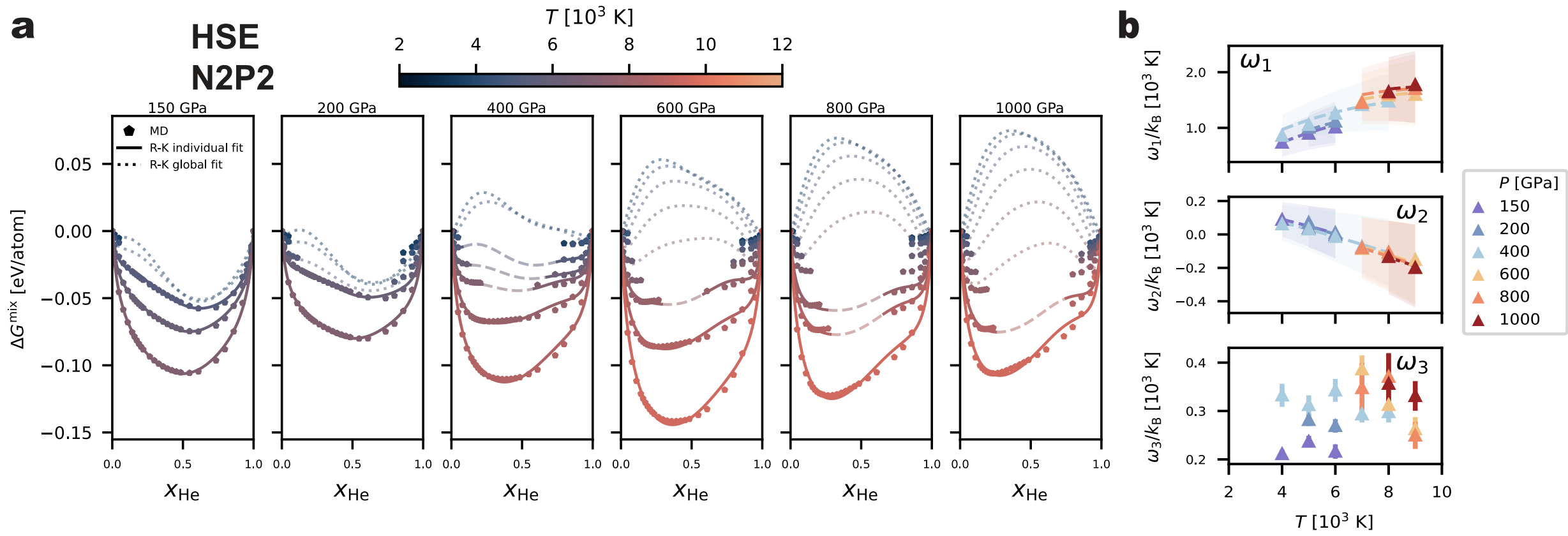

FIG. S22. The third-order Redlich–Kister (R–K) model fits of the mixing free energy (ΔG^{mix}) for H/He mixtures based on the HSE N2P2 MLP. (a) ΔG^{mix} computed from 150 to 1000 GPa and 2000 to 12000 K. The solid lines denote the fitted third-order R–K model at each P–T. The dashed lines represent the modeled ΔG^{mix} data from individual R–K fits for assumed homogeneous H/He mixtures within the demixing region. The dotted lines show ΔG^{mix} derived from a global R–K fit. (b) Fitted ω_i values derived from simulated ΔG^{mix}. The dashed lines, with shaded uncertainty bands, represent the polynomial fits for the pressure and temperature dependence of the $\omega_1(T, P)$ and $\omega_2(T, P)$ parameters.

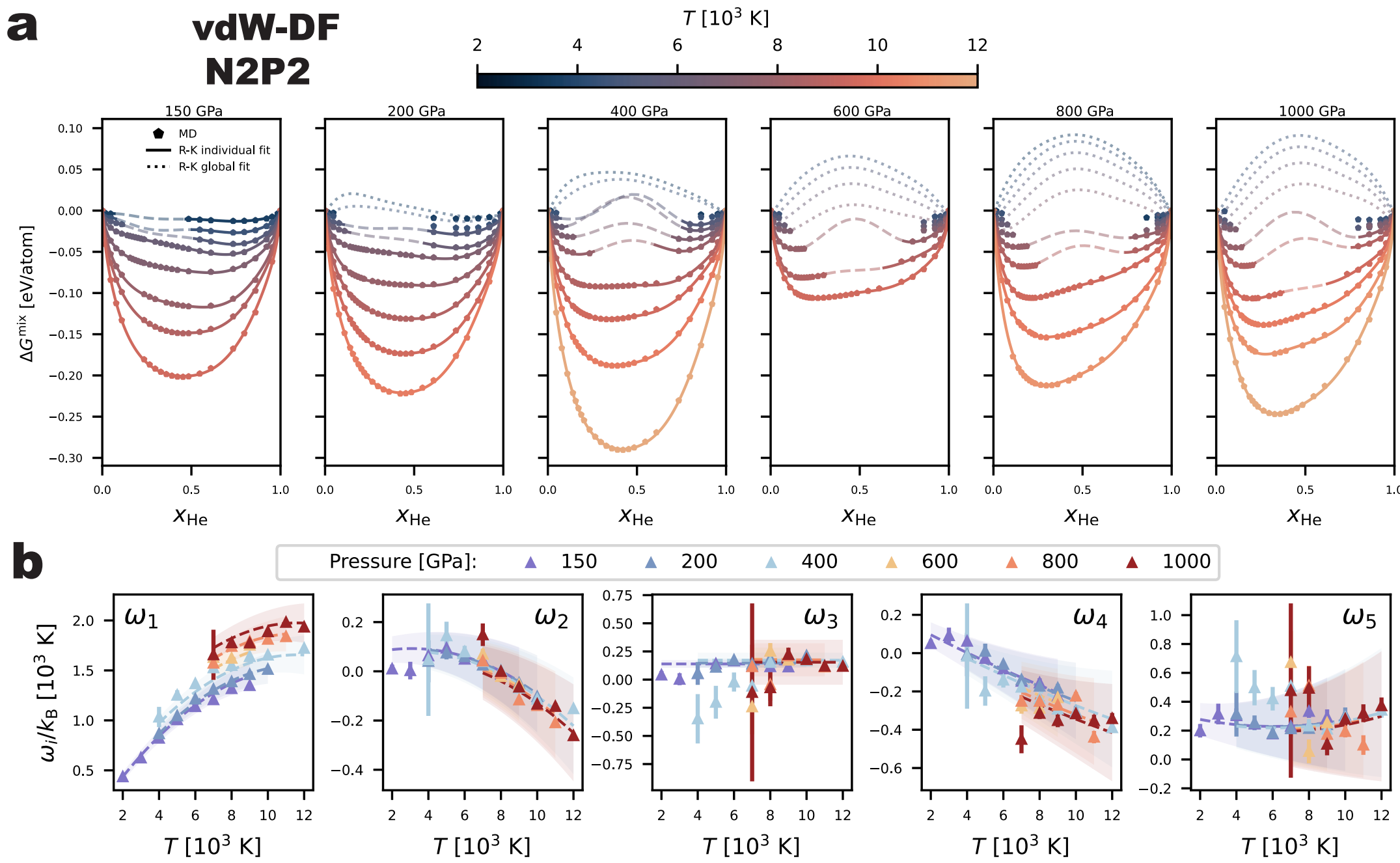

FIG. S23. The fifth-order Redlich–Kister (R–K) model fits of the mixing free energy (ΔG^{mix}) for H/He mixtures based on the vdW-DF N2P2 MLP. (a) ΔG^{mix} computed from 150 to 1000 GPa and 2000 to 12000 K, using the vdW-DF N2P2 MLP. The solid lines denote the fitted fifth-order R–K model at each P–T. The dashed gray lines indicate the modeled ΔG^{mix} values for assumed homogeneous H/He mixtures in the phase separation regime. (b) Fitted ω_i values derived from simulated ΔG^{mix}.

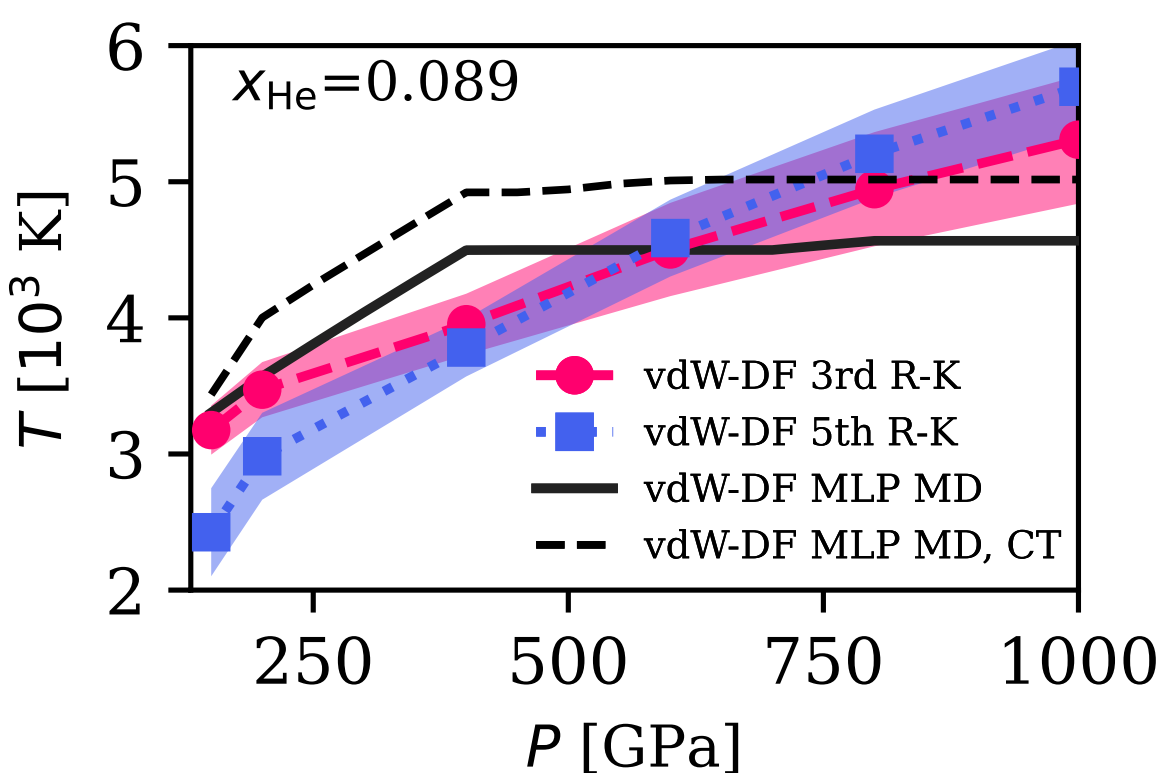

FIG. S24. Comparison of H/He immiscibility boundaries inferred from the third-order (dashed red) and fifth-order (dotted blue) Redlich–Kister fits to vdW-DF MLP MD data. The solid black line denotes the results from direct MLP MD simulations based on the criterion $\left(\partial^2\Delta G^{\rm mix}/\partial x_{\rm He}^2\right)_{T,P} \leq 0$, while the black dashed line denotes the results based on the common-tangent construction.

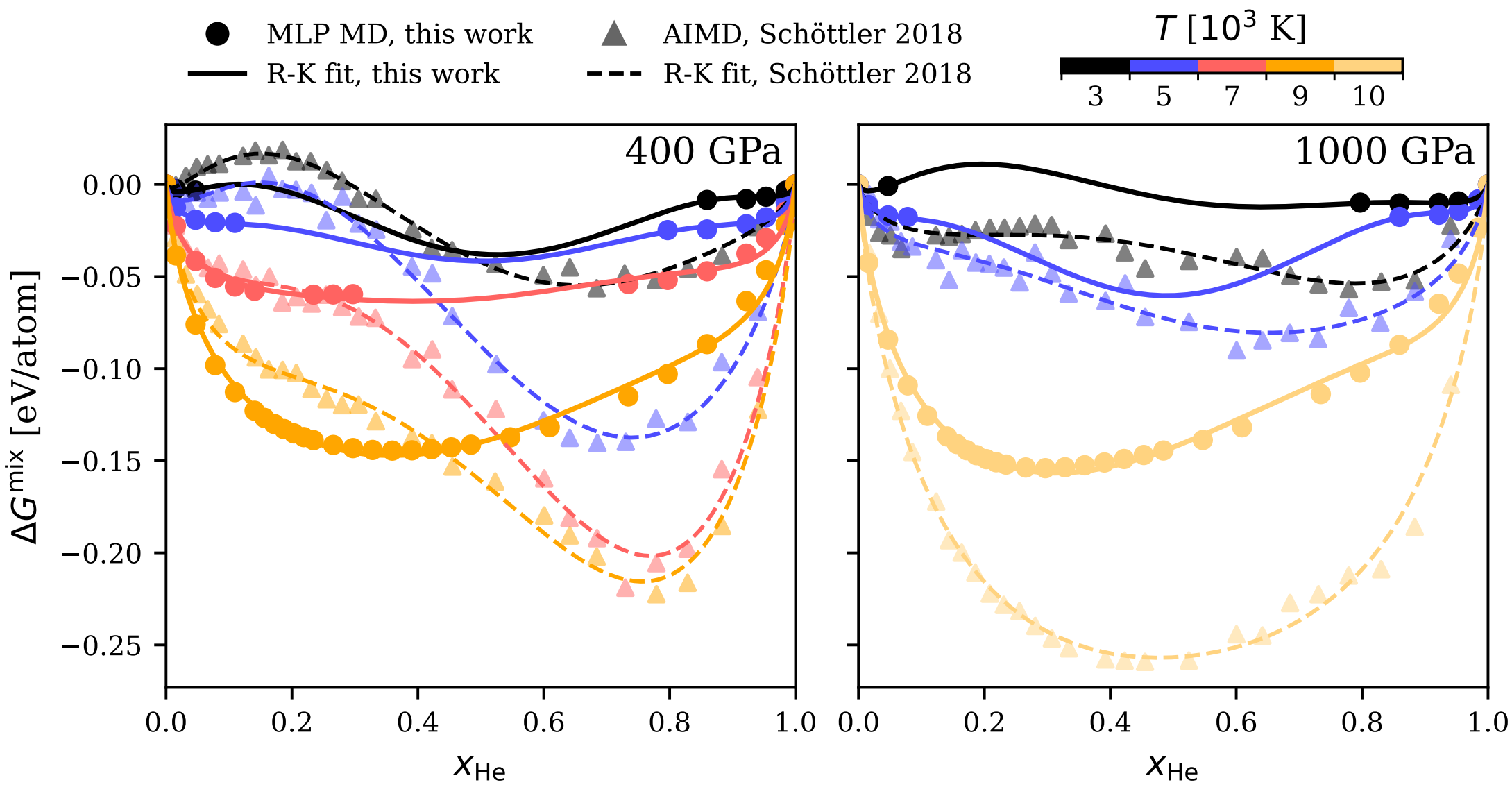

FIG. S25. Comparison of the Gibbs free energy of mixing ($\Delta G^{\rm mix}$) between the MLP MD and AIMD results [8] based on vdW-DF at 400 GPa (left) and 1000 GPa (right) over a range of temperatures. MLP MD results for $\Delta G^{\rm mix}$ are reported only for single-phase regions. Note that the AIMD data of $\Delta G^{\rm mix}$ at 400 GPa was derived by assuming the ideal entropy of mixing, whereas the data at 1000 GPa was analyzed by including nuclear quantum effects and non-ideal entropy contributions.

B. Prediction of the immiscibility boundaries

We employed the fitted R–K models to analytically determine the H/He immiscibility boundaries. At each P–T condition, phase separation is identified by the criterion $\left(\partial^2 \Delta G^{\text{mix}}/\partial x_{\text{He}}^2\right)_{T,P} \leq 0$, as expressed by:

$$(\frac{\partial^2 \Delta G^{\text{mix}}}{\partial x_{\text{He}}^2})_{T,P} = k_B T(\frac{1}{x_{\text{He}}} + \frac{1}{1-x_{\text{He}}}) - 2\omega_1 + 6\omega_2(2x_{\text{He}} - 1) + \omega_3(-48x_{\text{He}}^2 + 48x_{\text{He}} - 10) \leq 0. \tag{4}$$

We applied this criterion to predict the phase boundary for the protosolar helium fraction of $x_{\text{He}} = 0.089$, since Jupiter and Saturn are often assumed to have formed from the same material as the solar nebula [19, 28]. The resulting immiscibility boundaries for different functionals are presented in the main text in Fig. 3(c).

To validate our global-fit approach for the R–K parameters, we recalculated the miscibility gap based on our MLP MD results at 150 GPa and 200 GPa using the same individual P–T fitting protocol from previous AIMD work [8, 27]. As discussed in the main text, this alternative protocol from AIMD studies fits the R–K model at individual P–T conditions, without fitting polynomial expressions for the pressure and temperature dependence of ω_i. The resulting He-poor phase compositions from the vdW-DF, PBE, and HSE MLP subsets are summarized in Table S8. The derived H/He immiscibility boundaries are insensitive to the choice of density functional and are consistent with our global-fit approach reported in the main text, confirming the validity of both methodologies.

TABLE S8. Helium fraction (x_{He}) in the He-poor phase at the H/He phase immiscibility boundary. Predictions are obtained by fitting a third-order Redlich–Kister model independently at each P–T condition to MLP MD data subsets at 150 GPa and 200 GPa for three different functionals.

P [GPa]	T [10^3 K]	x_{He} (vdW-DF)	x_{He} (PBE)	x_{He} (HSE)
150	2.0	0.08	0.05	0.10
150	3.0	0.09	0.07	0.11
150	4.0	0.12	0.13	0.18
150	5.0	0.18	0.19	Fully mixed
200	2.0	0.06	0.05	0.05
200	3.0	0.08	0.06	0.05
200	4.0	0.11	0.10	0.10
200	5.0	0.14	0.13	0.17

C. Non-ideal mixing behavior

We further quantify the non-ideal mixing behavior of high-pressure H/He mixtures with the fitted R–K models. From Eq. 3, the mixing entropy can be taken from the temperature derivative as $\Delta S^{\mathrm{mix}} = -\left(\frac{\partial \Delta G^{\mathrm{mix}}}{\partial T}\right)_P = \Delta S^{\mathrm{id}} + \Delta S^{\mathrm{ex}}$. The excess entropy of mixing $\Delta S^{\mathrm{ex}}_{\mathrm{RK}}$ in the R–K model is then given by

$$\Delta S^{\mathrm{ex}}_{\mathrm{RK}} = -x_{\mathrm{He}} x_{\mathrm{H}} \sum_i \left(\frac{\partial \omega_i}{\partial T}\right) (1 - 2x_{\mathrm{He}})^{i-1} . \quad (5)$$

As a result, Fig. S26 presents the derived $\Delta S^{\mathrm{ex}}_{\mathrm{RK}}$ as a function of temperature for three DFT functionals, with ideal entropy of mixing ΔS^{id} (black dashed line) included for comparison. The $\Delta S^{\mathrm{ex}}_{\mathrm{RK}}$ values indicate a strong non-ideal mixing behavior in H/He mixtures, particularly at lower temperatures.

Likewise, the excess volume of mixing V^{ex} is obtained from the pressure derivative of ΔG^{mix} as $V^{\mathrm{ex}} = \left(\frac{\partial \Delta G^{\mathrm{mix}}}{\partial P}\right)_T$, which takes the form:

$$V^{\mathrm{ex}}_{\mathrm{RK}} = x_{\mathrm{He}} x_{\mathrm{H}} \sum_i \left(\frac{\partial \omega_i}{\partial P}\right) (1 - 2x_{\mathrm{He}})^{i-1} . \quad (6)$$

Consequently, the total volume of the mixture is expressed as the sum of the ideal components and the excess volume: $V^{\mathrm{mix}}_{\mathrm{RK}} = x_{\mathrm{H}} V^{\mathrm{pure}}_{\mathrm{H}} + x_{\mathrm{He}} V^{\mathrm{pure}}_{\mathrm{He}} + V^{\mathrm{ex}}_{\mathrm{RK}}$, where $V^{\mathrm{pure}}_{\mathrm{H}}$ and $V^{\mathrm{pure}}_{\mathrm{He}}$ were

obtained from MLP-MD simulations of the pure phases. The resulting density as a function of pressure for $x_{\mathrm{He}} = 0.09$ is shown in Fig. S27 for three DFT functionals.

For comparison, we also include the widely used tabulated EOS values for H/He mixtures from the previous study [29] with H/He interactions taken into account. Relative to these tabulated EOS data (black dotted line), the differences in our derived densities are at most 3.2% for vdW-DF, 7.2% for PBE, and 4.3% for HSE.

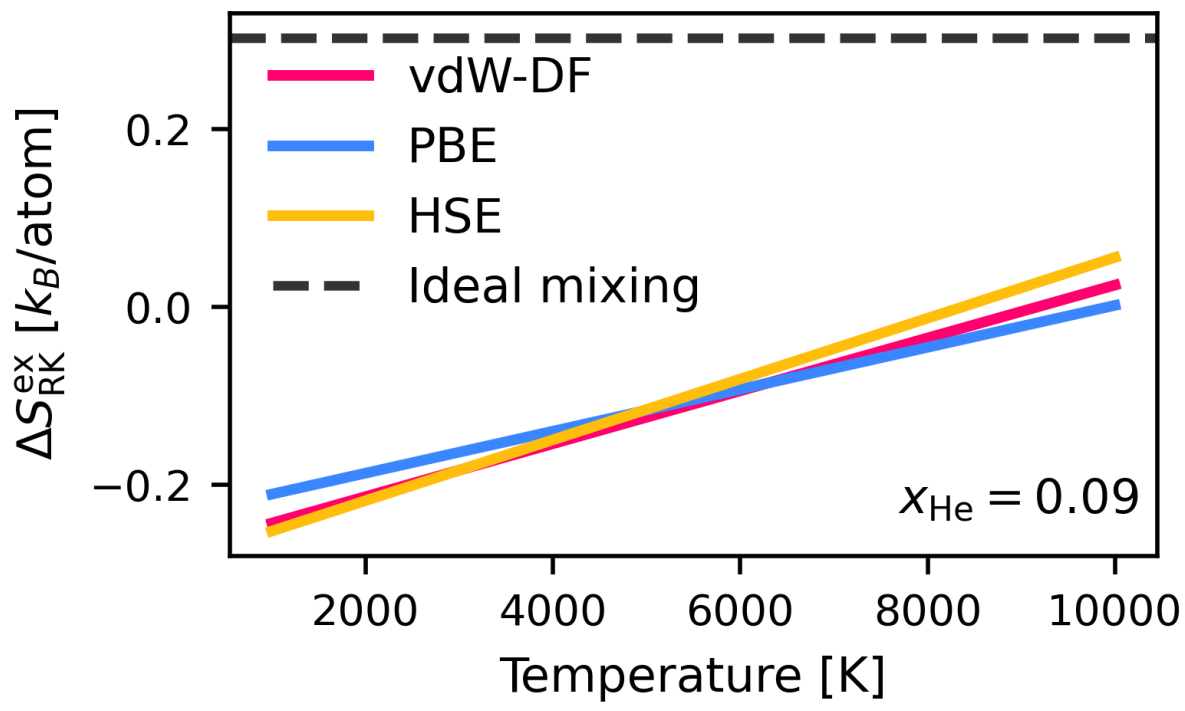

FIG. S26. Excess entropy $\Delta S^{\mathrm{ex}}_{\mathrm{RK}}$ computed from the fitted third-order Redlich–Kister models based on different DFT functionals.

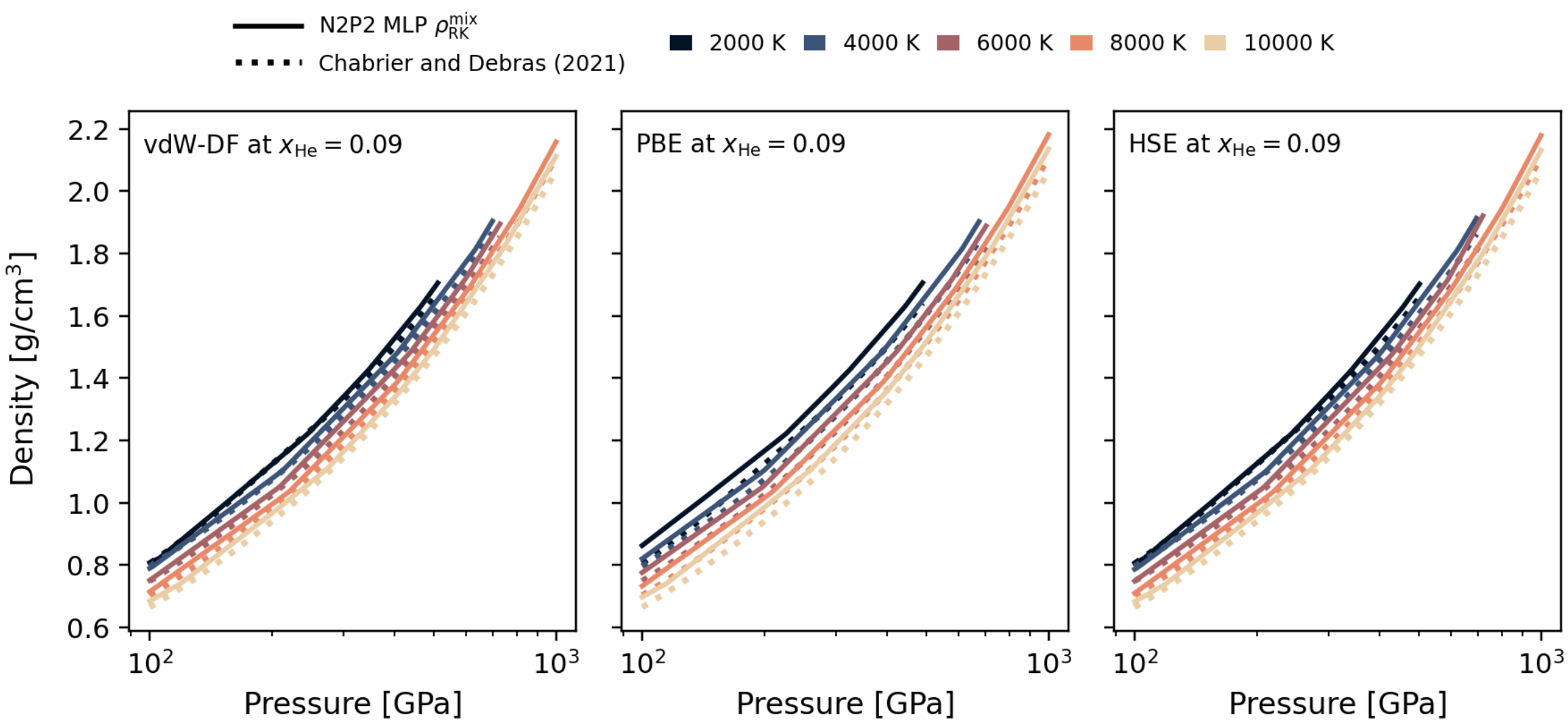

FIG. S27. Density as a function of pressure for $x_{\mathrm{He}} = 0.09$ at temperatures ranging from 2000 to 10000 K derived based on the R–K fits of vdW-DF (left), PBE (middle), and HSE (right) results, compared with the previous study [29].

VII. PATH INTEGRAL MOLECULAR DYNAMICS SIMULATIONS

To incorporate NQEs in H/He mixtures, we performed PIMD simulations based on the ring-polymer formalism [30] via the i-PI interface (Version 3.0) [31, 32] coupled with LAMMPS, using the vdW-DF N2P2 MLP. For comparison, we conducted both classical MD and PIMD simulations under three thermodynamic conditions relevant to giant-planet interiors: 1) 150 GPa and 2000 K, corresponding to molecular hydrogen; 2) 150 GPa and 3000 K, corresponding to atomic hydrogen; and 3) 400 GPa and 2000 K, corresponding to atomic hydrogen. At each P–T condition, we varied x_{He} from 0.016 to 0.98 across at least 24 compositions. A system size of $N = 2560$ and a timestep of 0.2 fs were selected to ensure numerical convergence of MD simulations and reduce the computational cost; see Section IV A.

In particular, we employed eight beads over the investigated temperature range (1000–3000 K) in PIMD simulations, based on convergence benchmarks in prior work [22, 23]. Consistent with the previous PIMD protocol for high-pressure hydrogen [23], each PIMD simulation began with a 10 ps equilibration in the NPT ensemble, followed by an 80 ps production run in the NVT ensemble. During the NPT equilibration stage, pressure was controlled using the isotropic stochastic barostat, with a relaxation time of 500 fs. Temperature was controlled using the stochastic path integral Langevin equation thermostat (`PILE_L`) in i-PI [33], with a relaxation time of 50 fs.

As shown in Fig. S28, the inclusion of NQEs (blue dashed lines) systematically increases ΔG^{mix} and therefore enhances H/He immiscibility. This trend is consistent with previous DFT results showing that the NQE contribution remains positive at lower pressures [34]. The maximum NQE estimated here, about 6 meV/atom at 150 GPa and 3000 K, is also consistent with the previously reported upper bound of 10 meV/atom at 100 GPa and 3000 K [34]. Overall, the inclusion of NQEs produces only a modest enhancement of H/He immiscibility.

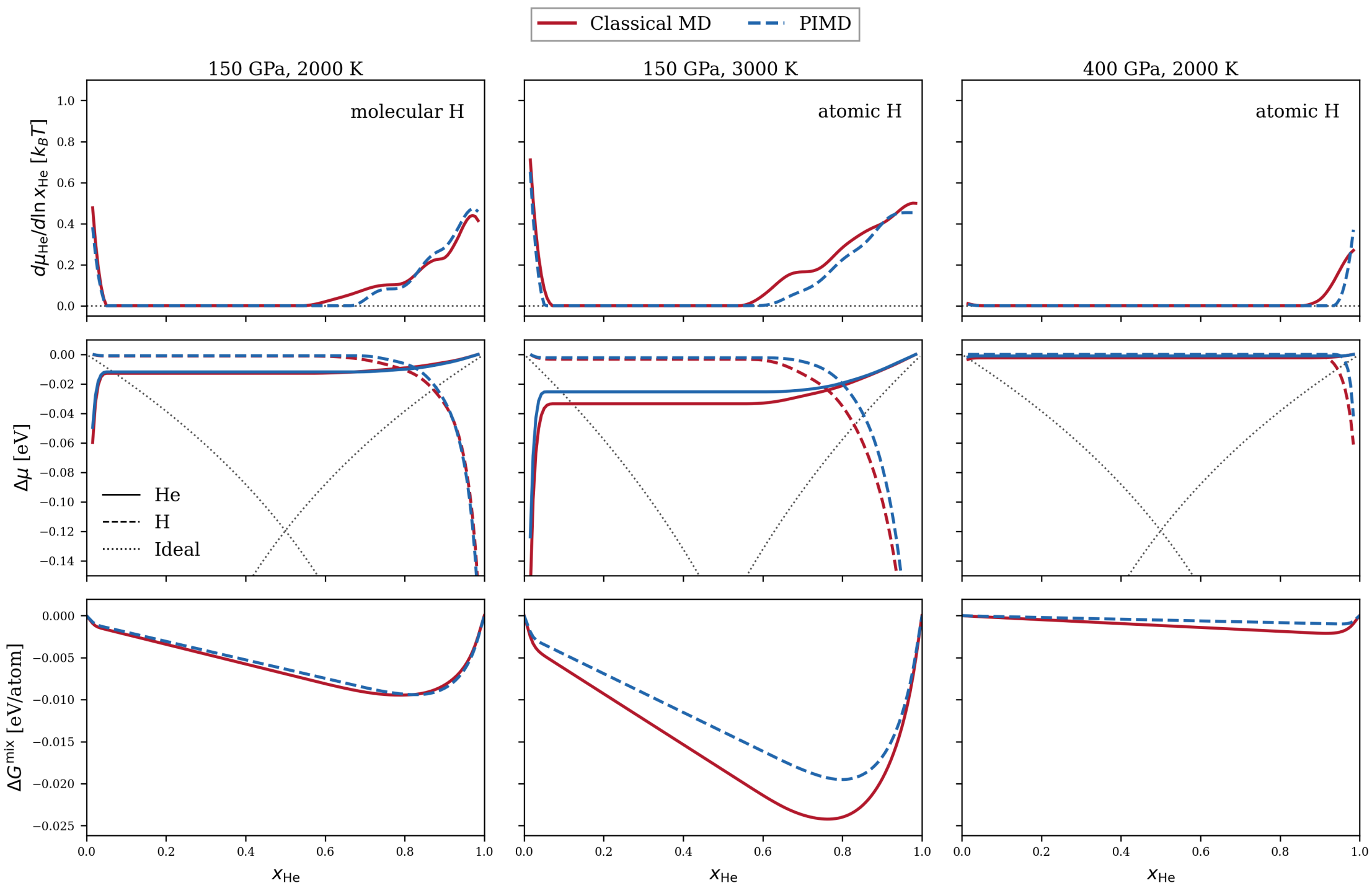

FIG. S28. Thermodynamic behaviors of H/He mixtures predicted by the vdW-DF N2P2 MLP with classical MD (red) and path integral MD (PIMD) simulations (blue). Each column represents a distinct thermodynamic condition: 1) 150 GPa and 2000 K (molecular H, phase-separated), 2) 150 GPa and 3000 K (atomic H, phase-separated), and 3) 400 GPa and 2000 K (atomic H, phase-separated).

VIII. SUPPLEMENTARY REFERENCES

[1] G. Kresse and J. Hafner, "*Ab initio* molecular dynamics for liquid metals," Physical Review B **47**, 558 (1993).

[2] G. Kresse and J. Hafner, "*Ab initio* molecular-dynamics simulation of the liquid-metal–amorphous-semiconductor transition in germanium," Physical Review B **49**, 14251 (1994).

[3] G. Kresse and J. Furthmüller, "Efficient iterative schemes for *ab initio* total-energy calculations using a plane-wave basis set," Physical Review B **54**, 11169 (1996).

[4] J. P. Perdew, K. Burke, and M. Ernzerhof, "Generalized gradient approximation made simple," Physical Review Letters **77**, 3865 (1996).

[5] K. Lee, É. D. Murray, L. Kong, B. I. Lundqvist, and D. C. Langreth, "Higher-accuracy van der Waals density functional," Physical Review B **82**, 081101 (2010).

[6] J. Heyd, G. E. Scuseria, and M. Ernzerhof, "Hybrid functionals based on a screened Coulomb potential," The Journal of Chemical Physics **118**, 8207 (2003).

[7] J. Heyd, G. E. Scuseria, and M. Ernzerhof, "Erratum: "Hybrid functionals based on a screened Coulomb potential" ," The Journal of Chemical Physics **124**, 219906 (2006).

[8] M. Schöttler and R. Redmer, "*Ab initio* calculation of the miscibility diagram for hydrogen-helium mixtures," Physical Review Letters **120**, 115703 (2018).

[9] A. Baldereschi, "Mean-value point in the brillouin zone," Physical Review B **7**, 5212 (1973).

[10] A. Singraber, T. Morawietz, J. Behler, and C. Dellago, "Parallel multistream training of high-dimensional neural network potentials," Journal of Chemical Theory and Computation **15**, 3075 (2019).

[11] I. Batatia, D. P. Kovacs, G. Simm, C. Ortner, and G. Csányi, "MACE: Higher order equivariant message passing neural networks for fast and accurate force fields," Advances in Neural Information Processing Systems **35**, 11423 (2022).

[12] B. Cheng, "Cartesian atomic cluster expansion for machine learning interatomic potentials," npj Computational Materials **10**, 157 (2024).

[13] B. Cheng, G. Mazzola, C. J. Pickard, and M. Ceriotti, "Evidence for supercritical behaviour of high-pressure liquid hydrogen," Nature **585**, 217 (2020).

[14] J. Behler and M. Parrinello, "Generalized neural-network representation of high-dimensional potential-energy surfaces," Physical Review Letters **98**, 146401 (2007).

[15] A. P. Thompson, H. M. Aktulga, R. Berger, D. S. Bolintineanu, W. M. Brown, P. S. Crozier, P. J. in 't Veld, A. Kohlmeyer, S. G. Moore, T. D. Nguyen, R. Shan, M. J. Stevens, J. Tranchida, C. Trott, and S. J. Plimpton, "LAMMPS - a flexible simulation tool for particle-based materials modeling at the atomic, meso, and continuum scales," Computer Physics Communications **271**, 108171 (2022).

[16] A. H. Larsen, J. J. Mortensen, J. Blomqvist, I. E. Castelli, R. Christensen, M. Dułak, J. Friis, M. N. Groves, B. Hammer, C. Hargus, E. D. Hermes, P. C. Jennings, P. B. Jensen, J. Kermode, J. R. Kitchin, E. L. Kolsbjerg, J. Kubal, K. Kaasbjerg, S. Lysgaard, J. B. Maronsson, T. Maxson, T. Olsen, L. Pastewka, A. Peterson, C. Rostgaard, J. Schiøtz, O. Schütt, M. Strange, K. S. Thygesen, T. Vegge, L. Vilhelmsen, M. Walter, Z. Zeng, and K. W. Jacobsen, "The atomic simulation environment—a Python library for working with atoms," Journal of Physics: Condensed Matter **29**, 273002 (2017).

[17] G. Mazzola, R. Helled, and S. Sorella, "Phase diagram of hydrogen and a hydrogen-helium mixture at planetary conditions by quantum Monte Carlo simulations," Physical Review Letters **120**, 025701 (2018).

[18] J. Vorberger, I. Tamblyn, B. Militzer, and S. A. Bonev, "Hydrogen-helium mixtures in the interiors of giant planets," Physical Review B **75**, 024206 (2007).

[19] R. Helled, G. Mazzola, and R. Redmer, "Understanding dense hydrogen at planetary conditions," Nature Reviews Physics **2**, 562 (2020).

[20] M. A. Morales, J. M. McMahon, C. Pierleoni, and D. M. Ceperley, "Nuclear quantum effects and nonlocal exchange-correlation functionals applied to liquid hydrogen at high pressure," Physical Review Letters **110**, 065702 (2013).

[21] B. Lu, D. Kang, D. Wang, T. Gao, and J. Dai, "Towards the same line of liquid–liquid phase transition of dense hydrogen from various theoretical predictions," Chinese Physics Letters **36**, 103102 (2019).

[22] J. Hinz, V. V. Karasiev, S. X. Hu, M. Zaghoo, D. Mejía-Rodríguez, S. B. Trickey, and L. Calderín, "Fully consistent density functional theory determination of the insulator-metal transition boundary in warm dense hydrogen," Physical Review Research **2**, 032065 (2020).

[23] S. van de Bund, H. Wiebe, and G. J. Ackland, "Isotope quantum effects in the metallization transition in liquid hydrogen," Physical Review Letters **126**, 225701 (2021).

[24] A. Bergermann, L. Kleindienst, and R. Redmer, "Nonmetal-to-metal transition in liquid hydrogen using density functional theory and the Heyd–Scuseria–Ernzerhof exchange-correlation functional," The Journal of Chemical Physics **161**, 234303 (2024).

[25] G. Tenti, B. Jäckl, K. Nakano, M. Rupp, and M. Casula, "Hydrogen liquid-liquid transition from first principles and machine learning," Physical Review B **112**, 104208 (2025).

[26] O. Redlich and A. Kister, "Thermodynamics of nonelectrolyte solutions - x-y-t relations in a binary system," Industrial & Engineering Chemistry **40**, 341 (1948).

[27] M. A. Morales, S. Hamel, K. Caspersen, and E. Schwegler, "Hydrogen-helium demixing from first principles: From diamond anvil cells to planetary interiors," Physical Review B **87**, 174105 (2013).

[28] J. N. Bahcall, M. Pinsonneault, and G. Wasserburg, "Solar models with helium and heavy-element diffusion," Reviews of Modern Physics **67**, 781 (1995).

[29] G. Chabrier and F. Debras, "A new equation of state for dense hydrogen–helium mixtures. ii.

taking into account hydrogen–helium interactions," The Astrophysical Journal **917**, 4 (2021).

[30] M. E. Tuckerman, *Statistical Mechanics: Theory and Molecular Simulation*, 2nd ed. (Oxford University Press, 2023).

[31] M. Ceriotti, J. More, and D. E. Manolopoulos, "i-PI: A Python interface for ab initio path integral molecular dynamics simulations," Computer Physics Communications **185**, 1019 (2014).

[32] Y. Litman, V. Kapil, Y. M. Y. Feldman, D. Tisi, T. Begušić, K. Fidanyan, G. Fraux, J. Higer, M. Kellner, T. E. Li, E. S. Pós, E. Stocco, G. Trenins, B. Hirshberg, M. Rossi, and M. Ceriotti, "i-PI 3.0: A flexible and efficient framework for advanced atomistic simulations," The Journal of Chemical Physics **161**, 062504 (2024).

[33] M. Ceriotti, M. Parrinello, T. E. Markland, and D. E. Manolopoulos, "Efficient stochastic thermostatting of path integral molecular dynamics," The Journal of Chemical Physics **133**, 124104 (2010).

[34] M. Schöttler, *Miscibility gap of hydrogen-helium mixtures at high pressures and temperatures*, Ph.D. thesis, Universität Rostock, Rostock, Germany (2018).
