# Supplementary material for: Hydrogen-helium immiscibility boundary in gas-giant planetary interiors from machine-learning molecular dynamics
Source: Nat Commun. 2026 Sep 1;17:10382. doi: 10.1038/s41467-026-77117-8 (PMC13627725; doi:10.1038/s41467-026-77117-8)
Supplement: Supplementary file 3 [file 41467_2026_77117_MOESM3_ESM.pdf]

nature portfolio

Peer Review File

Hydrogen–helium immiscibility boundary in gas-giant planetary interiors from machine-learning molecular dynamics

Corresponding Author: Professor Bingqing Cheng

This file contains all reviewer reports in order by version, followed by all author rebuttals in order by version.

Attachments originally included by the reviewers as part of their assessment can be found at the end of this file.

A version of this paper was originally rejected for publication by Nature Communications, however that decision was reconsidered after appeal by the authors.

Version 0:

Reviewer comments:

Reviewer #1

(Remarks to the Author)
This manuscript describes the calculation of immiscibility boundaries in hydrogen-helium mixtures at high pressure. The potential immiscibility of helium in high pressure hydrogen has wide implication in the understanding of planetary structure in Jovian planets. This topic has been the subject of many studies throughout the last 40 years. Due to the complexity in describing the thermodynamic properties of hydrogen-helium mixtures at high pressure, first-principles methods are typically used to make predictions when experimental measurements are absent, a typical case at the high pressures encountered in planetary interiors. Despite the great success in applicability and use of first-principles methods in the study of material properties, the calculation of immiscibility has been challenging due to the high sensitivity to finite-size effects, the complications associated to calculations of Gibbs free energies and the sensitivity of immiscibility boundaries to small energetic effects (e.g. poor convergence in systematic effects, etc). Because of these issues, the direct use of DFT in the evaluation of immiscibility boundaries in H-He mixtures has been difficult, although significant progress was made over the last decade.

This work presented in this article represents a significant step forward in this story. The authors use accurate DFT-derived effective potentials using modern machine-learning techniques. This allows them to reach system sizes and energetic resolution beyond what is allowed with direct use of DFT simulations, eliminating issues associated with finite system sizes. A second essential component is the use of the recently developed method that allows the calculation of the composition dependence of the chemical potential in complex mixtures using structure factor calculations, as opposed to traditional approaches involving particle insertion algorithms and/or thermodynamic integration.
The manuscript makes significant revisions to the understanding of Helium miscibility gathered over the last decade with direct simulations with DFT. I have the following recommendations to the authors.

1. The main conclusions of the article depend significantly on the ability of the ML potential to accurately reproduce DFT energetics over the entire range of compositions in the mixture. This is highly non-trivial, and unfortunately any systematic bias in the accuracy of the potential with Helium composition would lead to large changes in miscibility temperatures. It is difficult to assess the effect of using ML potentials in the resulting immiscibility temperatures. The authors provide extensive supplementary materials, but it is difficult to gauge this from all the data.

2. The authors should try to explain the reason for the discrepancy in their predicted immiscibility temperatures, compared to previous work. For example, if finite size effects are a leading cause, the authors can calculate transition temperatures using system sizes comparable to those used in DFT studies and demonstrate this explicitly. If the reason is the inaccuracy of the estimation of mixing Gibbs free energy, the authors can compare with previous calculations using thermodynamic integration. As it currently stands, it is difficult to gauge the accuracy of the new predictions.

3. ML potentials appear to have been fitted to DFT calculations with small systems, while calculations of the chemical potentials with the ML potentials used large systems. While the latter addresses the issues of size effects in the calculation of the chemical potential, electronic size effects are typically very large in these conditions and can bias results significantly. This is particularly important in the H-He mixture, since the increase in He induces a metal-to-insulator transition as the He composition increases, which is susceptible to electronic size effects. This in my opinion is a serious flaw in the current work. The authors should address this directly.

In summary, the predictions made by this article have wide implications in the understanding of the interior structure of hydrogen-rich planets and their approach uses state-of-the-art ML potentials and chemical potential calculations, but without direct evidence demonstrating the cause of the discrepancy with previous work it is difficult to assess the accuracy of the new predictions.

(Remarks on code availability)

Reviewer #2

(Remarks to the Author)
In this paper the authors calculate the miscibility of helium in hydrogen at very high pressures and temperatures. Before I read the paper, I believed that molecular helium is insoluble in metallic hydrogen, but otherwise they are soluble.

I dont understand helium rain (even though I've read the references). For a general reader, or even high pressure physics specialist, it would be good to have a clear explanation of what this is. Rain on earth is part of the water cycle, which requires precipitation (heavy droplets falling) and evaporation (water rising). If you really mean rain, please (find out) and explain the mechanism by which the helium rises. If you are simply talking about precipitation, use that correct term and explain that it isn't rain. Nobody talks about rain in vinaigrette...

There are a number of theoretical approximations involved which I will review first.

The Gibbs Free energy diffrence is taken from the S0 method laid out in Prof Cheng's paper
"Computing chemical potentials of solutions from structure factors". This claims to extract the gradient of the chemical potential directly from the partial Structure Factors. Although it is not widely used, and it seems extraordinary to me that it works, it does seem to be robust. Moreover, precise accuracy is not essential for the conclusions of the paper. Given the central role of S(q) in this, it would be good to see the S(q) plotted as well as the g(r) so the reader can see the convergence. Otherwise, I'm happy with S0, although this is a "gut-feeling" and I will be interested in what other reviewers say.

Second, a number of DFT calculations were carried out. Admirably, the author used three XC functionals, and Fig S12 shows this is a huge source of uncertainty, moving the atomisation temperature by 1000K between models. I think its safe to assign this to the XC functional rather than the MLIP fit - PBE is fairly well known to stabilise weakly bound molecular hydrogen, often with bonds >1A. We're not shown the hydrogen PBE g(r), and I wonder if this atomisation temperature is very sensitive to the choice of cutoff in Eq S1. Notably, this method and choice would claim that a fluid with 1A covalently bonded hydrogens was atomic. In fig 2, again we see at lower P the PBE calculations give much less solubility (which I assume links to metallization).

Third, the DFT data is approximated by machine-learned potentials. Again, the authors use several independent methods trained on exactly the relevant conditions for this work. The MLIPs are therefore interpolating and they should work well. That they give consistent results is encouraging.

So I'm happy that the methods are robust form extending Born-oppenheimer MD through MLIPs to calculate mixing free energy. This is the basis of an excellent paper in a specialist journal. However, Nature Communications is aimed a a general audience and, specifically, requires correct and novel physics as well as accurate methodology

Now we see for Fig 2g that the planetary-relevant (and presumably all) immiscibility boundaries are much lower in temperature than previous thought, either in experiment or straight DFT calculations. This is a strong claim, and a central result certainly novel enough for general audience. But, as a referee, I want to be confident it is correct.

This is especially relevant because the senior author has had this type of problem before, where MLP was used to increase the simulation size beyond what DFT could do, but once the DFT capabilities caught up, there was no sign of the finite size effects that the MLP claimed. https://www.nature.com/articles/s41586-021-04078-x

Another highly problematic is the lack of any quantum treatment of the protons. The underlying physics of the H-He incompatibility is the metallization of hydrogen which. An extensive body of experimental and theoretical work with diamond anvils and with path integral MD shows there are strong isotope effects in the metallization transition in liquid hydrogen (surprisingly, this work is not referenced).

I believe that if all the calculations here were repeated with deuterium instead of hydrogen, the phase boundaries would be identical (if not, where in the theory does a difference arise?). So the metal/insulator phase boundary is calculated for classical protons which is of theoretical interest only. Again, the authors have experience of full PIMD calculation enabled by

MLPs (e.g. https://www.pnas.org/doi/10.1073/pnas.1815117116) where they "reveal the crucial role of nuclear quantum fluctuations in modulating the thermodynamic stabilities of different phases of water"

So, while I accept this is careful and extensive technical work, I'm not convinced by the headline conclusion and I dont recommend publication in a general interest journal like Nature Comms.

(Remarks on code availability)
No code was given in the submission.
I am familiar with, and regularly use, the community codes used, VASP, LAMMPS ASE

Reviewer #3

(Remarks to the Author)
The manuscript by Wang, Hamel and Cheng is a very thorough calculation of the immiscibility boundary in a hydrogen-helium mixture. They use DFT calculations, machine learning models and extensive molecular dynamics to make the best estimate to date of the phase diagram. They then discuss the implications for the gas giant planets, Jupiter and Saturn. As their extensive references document, this scientific question has a history extending more than 50 years. However, modern computational technique are reaching the point where definitive answers are within reach. I think the present manuscript is likely to be the best study though I have several reservations that I discuss below. What makes it stand out is that they have employed three different DFT functionals and three different machine learning models. This enables one to get some idea of how sensitive the results are to the underlying assumptions. Their phase diagram (fig. 2) predicts a lower phase separation temperature than experiment and earlier works, but still of crucial importance in modeling Jupiter and Saturn.

I think this work shows definite progress in predicting this phase diagram. There are several major uncertainties however, that suggest to me that the story is not quite finished.

-are the DFT functionals and calculations accurate enough for hydrogen and helium in this regime? I am particularly concerned with the dispersion interaction, the finite size effects thermal electron energies in the hydrogen DFT calculation and differences caused by the different energy scales of hydrogen and helium. say from the use of pseudopotentials at high pressure.

-are the machine learning models adequate and indeed even necessary? In the introduction they say "DFT studies are constrained to system sizes of several hundred atoms...finite size effects can be significant for phase transition.." Although such a statement is the accepted wisdom for some phase transitions, I question whether size effects are important here. It would seem to me that if one does careful free energy calculations of homogenous helium and hydrogen phases and then carefully studies the changes when a few helium atoms are introduced into the hydrogen and vice versa, one should be able to get an accurate idea of at least the low and high concentration sides of the phase separation. using hundreds of atoms, thus avoiding the necessity of the machine learning approximation. Such a check would be more convincing. The pair correlation functions shown in the SI are informative but what is needed are the actual energetic and pressure comparison between the ML models and the DFT calculations.

I find the discussion of how the machine learning potential was trained and tested (see supplementary material) inadequate. How do we relate the RMS errors of the energy (is that per atom?) and force, to the errors in the phase diagram? I understand that these measures are standard in the field, however what should be important is whether a given model is biased toward pure hydrogen, helium, or a mixture and whether there is any pressure dependance in the bias.

I am also concerned that the "configurations spanned a broad range of PT conditions from 1-1200GPa.." Is this a good idea? Would't it be more accurate to have the training set be in the region of density relevant to the planetary adiabat, say 400GPa-600GPa and 3000K to 6000K. Was the composition of the training set controlled to represent the 3 phases (pure H, pure He and mixed) equally to avoid biasing the results? The training sets should be made available, otherwise the models and the results are not reproducible.

(Remarks on code availability)
The training set needs to be made available.

Reviewer #4

(Remarks to the Author)
The paper predicts the conditions of H-He immiscibility as they apply to our understanding of the evolution of giant planet interiors. The authors find that immiscibility and Helium rainout are likely to be occurring in Saturn today, whereas He rainout may not be occurring in Jupiter. These are important results that will serve as a baseline in future efforts to understand the thermal evolution of these bodies, their recently discovered compositional inhomogeneity, and the generation of their magnetic fields.

There have been many previous predictions of H-He immiscibility based on ab initio molecular dynamics simulations and

the present results place the critical temperature at the low temperature end of these previous efforts. It should be emphasized that predicting immiscibility in this system is computationally challenging and previous efforts may have been biased by small system sizes or limitations in the methodology, as the present authors discuss in some detail. As compared with previous work, I appreciated in this paper the use of relatively large systems, the application of the S0 method, which allows for very dense sampling of P-T-x space, the careful contstruction of MLP and comparison of multiple XC functionals.

I recommend publication after the following comments are addressed.

1) I would appreciate further discussion of the MLP construction, particularly addressing the following points: a) Errors in energy and force are probably acceptable, but not the smallest that have been published. For example, Deng et al. (PRB, 2023) in a three-component system over a comparable PT range produce energy and force RMSE at the low end of what is reported here while also fitting the viral very well. b) Do the authors attempt to fit the viral tensor? If not why? If so, what are the results? c) fitting to energy/force/(viral) involves meta-parameters that provide relative weights to these misfits. How did the authors choose these weights?

2) S0 method. I would appreciate confirmation of the convergence of this method in k-space for this system with a plot similar to that of Fig. 2 of Chen (JCP, 2022) .

3) S0 method. The authors recognize the issue of two-phase separation as it occurs during simulations more completely than do many previous studies. The authors argue that in their method, immiscible states are manifested by d \mu / dn = 0. I would appreciate a demonstration or argument confirming this result starting from their Eq. 1. It is not immediately obvious to me from the right hand side of this equation that a phase separated system should yield zero.

4) N-dependence. I may have missed this but the authors should discuss the dependence of their results on the system size.

5) ENCUT. The authors use ENCUT=1200 eV, which is very large, but their PAW potential is also extremely hard. They must demonstrate convergence of their DFT results with respect to ENCUT.

6) Fig. 2. Their critical curves for PBE and vdW-DF display prominent jogs near 600 GPa. The authors should comment on the possible origin of these features.

7) Fig. 2. The authors should say something more about the serious disagreement between their results and the experimental results of Brygoo et al.

8) RK. The authors do a sensible job of fitting their results to RK. These fits provide an additional opportunity to compare with previous results and I would appreciate the authors exploring this. T- and P- derivatives of Eq. 3 yield the entropy and volume of mixing (and the excess quantities). The former could be compared to the ideal mixing result, while the latter could be compared to widely used tabulated eos of H, He and H-He mixtures (Chabrier et al.), where volume mixing across the H-He join is known to be significantly non-ideal.

(Remarks on code availability)

Reviewer #5

(Remarks to the Author)
I co-reviewed this manuscript with one of the reviewers who provided the listed reports. This is part of the Nature Communications initiative to facilitate training in peer review and to provide appropriate recognition for Early Career Researchers who co-review manuscripts.

(Remarks on code availability)

Version 1:

Reviewer comments:

Reviewer #1

(Remarks to the Author)
Regarding the replies from the authors to the reviewer comments, I believe the authors did a good job attempting to address the concerns. The authors clearly went to great lengths to provide numerical evidence and tests for most of the reviewers concerns. I think the manuscript is clear, provides a comprehensive study of H/He miscibility using ML potentials and provides an accurate answer to the miscibility question in terms of the generated potentials. For these reasons, I think the paper should be published.

I must say, on the other hand, that the question of whether the ML potentials faithfully represent the associated DFT-based phase diagram for the mixture is still an open question. The free energy integration presented in the latest version of the paper does suggest that this is indeed the case, but in my opinion it is still something that we must assume/accept. Finally,

the paper does not address the limitations of DFT and the associated Born-Oppenheimer approximations inherent in the calculations, so questions still remain unfortunately.

(Remarks on code availability)

Reviewer #2

(Remarks to the Author)
I would like to thank the authors for their thorough response and testing.

The paper now makes a number of conclusions which I find strange. That's fine of course, the whole point of research is to reveal new physics, but they are somewhat hidden in the manuscript which risks being misleading.

Firstly, although the methodology is impressive, there are very large sources of error. It is quite hard to quantify these for a general reader, because they depend on P,T and concentration. But it seems to me that the uncertainty is roughly

500K (10%) cutoff dependence in g(r).
1000K from choice of XC functional
400K in atomisation from NQE
100K (10meV) from error in MLP training
+ some unquantified error from the S(0) method.

So essentially the uncertainty in the calculation is close to the reported effects. Uncertainty in conclusions needs to be stated up front in the abstract, not buried in SM.

A new conclusion is that the solubility lines is unaffected by NQE, even though NQE moves the metallization/atomization temperature by ~ 500K. Scientifically, this is far more interesting than a calculated phase boundary with large errors. Is the enthalpy of mixing somehow unrelated to metallization? It seems like new chemistry - at least to me. Please explain?

In Fig S9 it is shown that in the "mixed" atomic region 3000K/150GPa there is a big peak and the H2 distance, and at 5000K it is even bigger (for higher He%). Does it mean that the extra helium is somehow significantly driving H2 formation? What is the chemistry behind that? I presume it can be seen in the DFT calculation.

I have now lost track of the big picture here with regard to enthalpy of solution: is it the case that enthalpy of mixing is positive in atomic and
metallic hydrogen, so the solubility in the deep planet is driven by temperature rather than pressure. As I understand Fig 2d-f, the solubility boundary is where the red line crosses the black one: i.e. there are two solubility boundaries. Firstly, the enthalpy of mixing He in metallic H drives phase separation, then the high temperature overcomes this deeper in the planet. This would mean they calculated two physical miscibility boundaries in the planets from just one in the PT phase diagram. Is that correct? It seems like something that could be made clearer.

The story about helium rain remains confusing. In the introduction the authors state:

"Precipitate as helium-rich droplets that sink toward the planetary core under gravity. This phase separation and subsequent gravitational settling of helium rich components are commonly referred to as “helium rain”

Which is how I understood it from detailed reading of Helled etc., but not how almost every general reader understands it. As I said before, its deeply misleading to call this one-off process "rain", but that's perhaps not the author's fault. I suspect if they state clearly that their model predicts at most a one-off precipitation rather than a rain cycle they would increase their paper impact significantly. (Even a one-off event depends rather sensitively on the details of the planet formation.

But now in Fig 1b they show the "rain" between the molecular and metallic regions. In general I think the planetary "core" means the heavy elements below the metallic hydrogen layer, not the metallic hydrogen layer itself. Certainly this is what Helled etc. mean when they talk about "fuzzy core" of Jupiter. So it seems Fig 1b is at variance with the introduction about where the rain occurs (this may just be terminology).

So my basic summary of the paper is that...

With the best available calculation methods it is still very difficult to get sufficient accuracy to say anything definitive about solubility in Jupiter and Saturn. However within the uncertainty of teh mode is it possible that there are zero, one or two miscibility boundaries in Jupiter, and either one of two in Saturn. The model has some very strange chemistry and unexplained wherein He in solution disrupts metallisation, forming more hydrogen molecules.

(Remarks on code availability)

Reviewer #4

(Remarks to the Author)
The authors have addressed my comments very thoroughly. My remaining comments are relatively minor. I will continue to use the same numbers for the various points:

1. MLP training. I am still unclear on the MLP construction, particularly the use of the viral or lack thereof. I appreciate the authors' having done the extra work to train an MLP including the virial. The problem I still have though is that the RMS error in the viral is quite large: 4 GPa, as compared with 0.4 GPa in Deng et al. (2023) PRB.

3. Equation 1. Thanks for the derivation, which makes sense. My question however was a different one. My question was about the right-hand side of the equation. Is there a simple argument one can make that would help me understand why the RHS must become very small in an inhomogeneous system? i.e. something having to do with the values of S_ab(k->0) in an inhomogeneous as opposed to a homogeneous system. I am not questioning the basic result, and indeed I can confirm myself using numerical values of S_ab(k->0) that you have now provided that the RHS is indeed very small for the demixed cases. I am looking for a simple qualitative argument. Hope this makes sense.

6. Jog. I accept that the jog is an artifact of interpolation. Is it not possible to perform the interpolation in a slightly different way that makes the jog disappear?

8. RK. I appreciate the density vs. pressure plots now provided. I think it would be even more useful to compare the EXCESS volume of the RK against Chabrier and Debras.

(Remarks on code availability)

Reviewer #5

(Remarks to the Author)
I co-reviewed this manuscript with one of the reviewers who provided the listed reports. This is part of the Nature Communications initiative to facilitate training in peer review and to provide appropriate recognition for Early Career Researchers who co-review manuscripts.

(Remarks on code availability)

Version 2:

Reviewer comments:

Reviewer #2

(Remarks to the Author)
I thank the authors for the careful reply.

Most of the review stage concentrated on the technical aspects of the paper, I would like to move now to the bigger picture:

Like referee 1, I take on trust "that the question of whether the ML potentials faithfully represent the DFT". The authors have worked hard to address it with respect to the phase boundary and the FEP and say essentially that it is not proved, but what more could they do? I sympathise, and note a simple resolution:

The big difference between DFT and MLIP-predicted demixing temperature is now highlighted in the abstract - this seems like the main new finding of the paper. The difference is attributable to finite size effects, this being the only place that MLIPs have an advantage over DFT. In the SM, they run the MLIP on these too-small system and show that they can accurately reproduce the finite size errors. I dont fully understand why the S(0) in fig S10 is clearly unconverged, but the dG in fig S12 are converged. Can they say more about this?

I asked before:

"This would mean they calculated two physical miscibility boundaries in the planets from just one in the PT phase diagram."

I think they misunderstood the question. They state that there is only one immiscibility surface in the three-dimensional P-T-xHe space (I agree). But what I meant was that there are miscibility boundaries in the planets: i.e. as one descends into the

planet, there are two crossings of this surface (miscible -> immiscible -> miscible). This is strongly implied by Fig 2b. But if I understand the figure correctly, it should mean that the brown and red lines in figure 2d-f cross the black line twice? So the two-boundary picture in 2b applies only to PBE data and a couple of th e thermal profiles. So 2b shows an unusual scenario for their calculations, applying only to the least reliable of the functionals - is that correct? Also, in figs 2d-f it seems that the planetary thermal profiles don't cross into the molecular region for the chosen P range. That's not wrong, but creates a little confusion comparing to 2b

I'm fairly sure this is more a matter of clarification than criticism.

Also in figure 2, they show the black line crossing the atomic-molecular boundary. This implies that helium is immiscible in fluid molecular hydrogen. It that really what the model shows?

(Remarks on code availability)
I have not tried to run the code. Given the number of review requests I get and the timescales for reviewing 60 pages of MS and SM, this is not practical without funding.

Reviewer #4

(Remarks to the Author)
I appreciate the authors thoughtful responses to my concerns. I am happy to now recommend publication.

(Remarks on code availability)

Reviewer #5

(Remarks to the Author)
I co-reviewed this manuscript with one of the reviewers who provided the listed reports. This is part of the Nature Communications initiative to facilitate training in peer review and to provide appropriate recognition for Early Career Researchers who co-review manuscripts.

(Remarks on code availability)

Version 3:

Reviewer comments:

Reviewer #2

(Remarks to the Author)
I downloaded and ran the PBE/MACE potentail at low T where the structures are well known. The I4amd structure seems to be stable, and at 200GPa the potential created H2 molecules as it should - the exact structure is still controversial and not especially relevant. Obviously this is not very thorough but it was reassuring.
Im happy to recommend the paper for Nature Communications.

(Remarks on code availability)
I got chatGPT to download one version of the from the given github site and run it in the ASE native MACE interface. This all worked smoothly and automatically and the results are sensible.

Open Access This Peer Review File is licensed under a Creative Commons Attribution 4.0 International License, which permits use, sharing, adaptation, distribution and reproduction in any medium or format, as long as you give appropriate credit to the original author(s) and the source, provide a link to the Creative Commons license, and indicate if changes were made.
In cases where reviewers are anonymous, credit should be given to 'Anonymous Referee' and the source.
The images or other third party material in this Peer Review File are included in the article's Creative Commons license, unless indicated otherwise in a credit line to the material. If material is not included in the article's Creative Commons license and your intended use is not permitted by statutory regulation or exceeds the permitted use, you will need to obtain permission directly from the copyright holder.
To view a copy of this license, visit https://creativecommons.org/licenses/by/4.0/

We thank the referees for their comprehensive comments on our manuscript. The referees' comments helped to further clarify the method and strengthened the conclusions.

We performed an extensive revision of the paper. A list of major changes/additions:

- **Additional benchmarks and validations of the MLPs** based on **7000 new DFT calculations.** In particular, we quantified the effect of the MLP error on the predicted immiscibility boundary by promoting the MLP-derived mixing free energy to the DFT level, using free energy perturbation. We found that the MLP reproduces DFT thermodynamics well across the investigated P-T-x_{He} space, with the resulting uncertainty in the immiscibility boundary limited to less than 1000 K.
- Systematic characterization of **the finite size effects in DFT and MD calculations**. In particular, for MD simulations, we analyzed the system-size effect on radial distribution functions, structure factors, mixing free energies, and the inferred demixing boundary. These tests show that large-scale simulations are required for converged free energy calculations.
- Comparison of new **MLPs trained using alternative fitting protocols**, including models trained with stress/virial data and models trained on restricted subsets of the dataset. These comparisons further validate the training strategies and model architectures of our MLPs.
- Quantification of **nuclear quantum effects** using path-integral MD simulations based on the ring-polymer formalism. We found that nuclear quantum effects lower the hydrogen molecular-to-atomic transition temperature by up to 450 K and have only a minor effect on enhancing H/He immiscibility.
- Expanded discussion of the **reliability of the underlying exchange-correlation functionals** used in the DFT calculations, with reference to available QMC benchmarks.
- Clarification of the **physical interpretation and planetary implications of helium rain**. We cross-checked the latest planetary models for Jupiter and Saturn and further developed our discussion.

In what follows, we address each of the points raised by the referees. The revision of the manuscript in response to the referees' comments is highlighted in blue in the main text and supplementary information.

Reviewers' comments:

Reviewer #1 (Remarks to the Author):

This manuscript describes the calculation of immiscibility boundaries in hydrogen-helium mixtures at high pressure. The potential immiscibility of helium in high pressure hydrogen has wide implication in the understanding of planetary structure in Jovian planets. This topic has been the subject of many studies throughout the last 40 years. Due to the complexity in describing the thermodynamic properties of hydrogen-helium mixtures at high pressure, first-principles methods are typically used to make predictions when experimental measurements are absent, a typical case at the high pressures encountered in planetary interiors. Despite the great success in applicability and use of first-principles methods in the study of material properties, the calculation of immiscibility has been challenging due to the high sensitivity to finite-size effects, the complications associated to calculations of Gibbs free energies and the sensitivity of immiscibility boundaries to small energetic effects (e.g. poor convergence in systematic effects, etc). Because of these issues, the direct use of DFT in the evaluation of immiscibility boundaries in H-He mixtures has been difficult, although significant progress was made over the last decade.

This work presented in this article represents a significant step forward in this story. The authors use accurate DFT-derived effective potentials using modern machine-learning techniques. This allows them to reach system sizes and energetic resolution beyond what is allowed with direct use of DFT simulations, eliminating issues associated with finite system sizes. A second essential component is the use of the recently developed method that allows the calculation of the composition dependence of the chemical potential in complex mixtures using structure factor calculations, as opposed to traditional approaches involving particle insertion algorithms and/or thermodynamic integration.
The manuscript makes significant revisions to the understanding of Helium miscibility gathered over the last decade with direct simulations with DFT. I have the following recommendations to the authors.

1. The main conclusions of the article depend significantly on the ability of the ML potential to accurately reproduce DFT energetics over the entire range of compositions in the mixture. This is highly non-trivial, and unfortunately any systematic bias in the accuracy of the potential with Helium composition would lead to large changes in miscibility temperatures. It is difficult to assess the effect of using ML potentials in the resulting immiscibility temperatures. The authors provide extensive supplementary materials, but it is difficult to gauge this from all the data.

Authors:
The referee correctly pointed out that the accuracy of the MLP would affect the computed immiscibility boundary. To respond to this, we have done two sets of analyses:

1) We performed additional **benchmarks of the MLP against DFT** using a large system size of **N=1024** atoms, which covers the whole range of helium compositions at three P-T conditions;
2) We quantified the **effect of the MLP error** on the predicted miscibility boundary by computing the mixing free energy ΔG^{mix} at the DFT level, using the free energy perturbation (FEP) approach to promote the MLP free energies to the DFT level.

As a result, we confirmed that the MLP accurately reproduces DFT energies across the investigated P-T-x_{He} space, and the MLP-derived phase boundaries are consistent with the DFT reference within an uncertainty of 1000 K. The details for the tests are discussed in the following:

1) To demonstrate that the ML potential can accurately reproduce DFT energies, we evaluated both vdW-DF DFT and N2P2 vdW-DF MLP energies on a dataset of 7000 snapshots. For each system size (N=64 and N=1024), these configurations cover 12 helium compositions (x_{He} = 0.016, 0.047, 0.11, 0.20, 0.30, 0.39, 0.55, 0.61, 0.73, 0.80, 0.92, and 0.98) for 2000 K and 5000 K at 150 GPa, and 11 compositions (x_{He} = 0.016-0.92) at 800 GPa and 7000 K.
   As shown in Fig. R1, **the DFT and MLP energies are in close agreement for both system sizes** across the investigated P-T-x_{He} space, with R^2=0.9999. **Most importantly, the large system size (1024 atoms) does not deteriorate the accuracy of the MLP**.

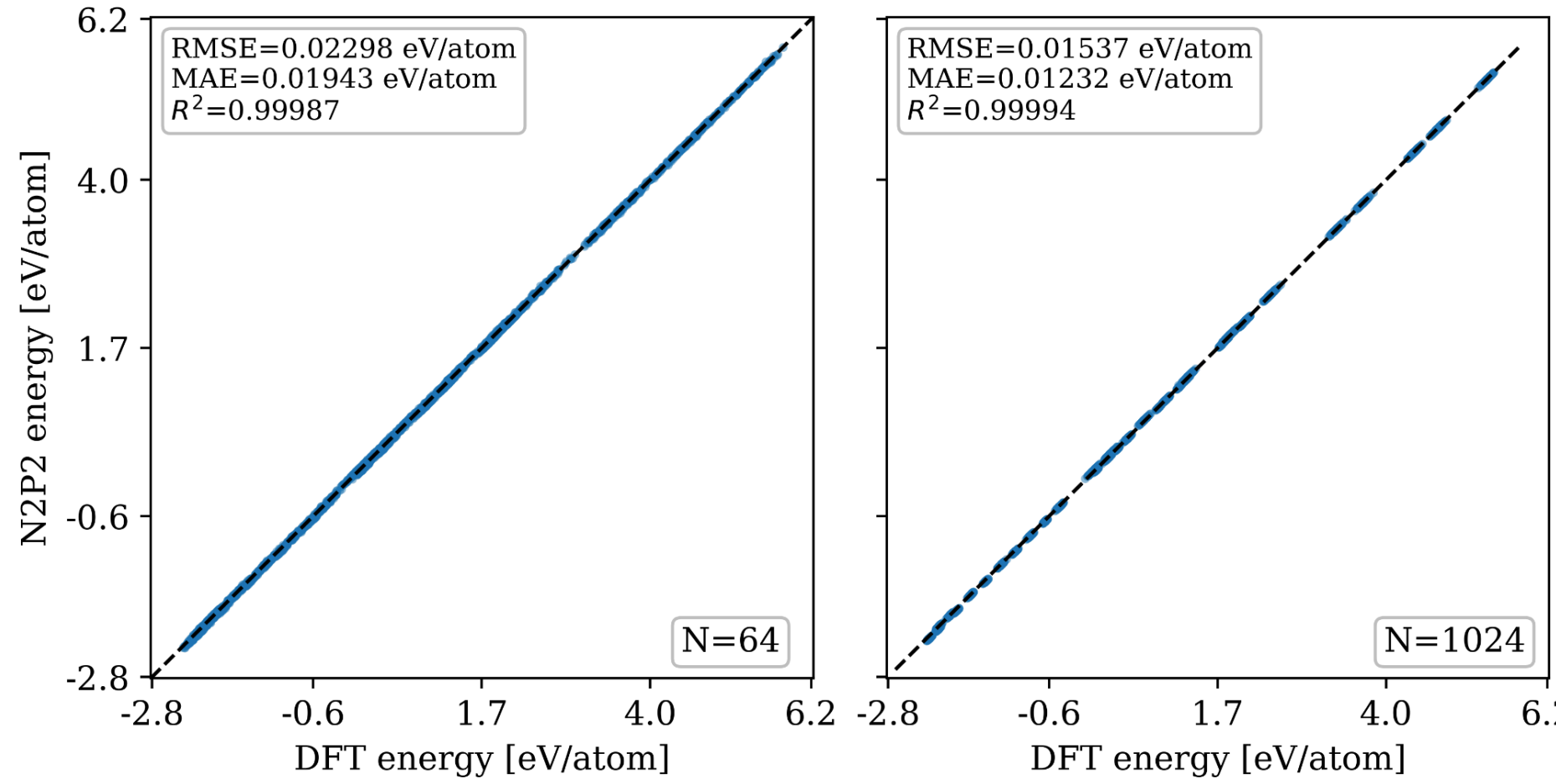

Fig. R1. Parity comparison of per-atom energies predicted by the N2P2 MLP against vdW-DF DFT reference energies for H/He mixtures at 150 GPa and 2000 K, 150 GPa and 5000 K, and 800 GPa and 7000 K. The left and right panels show the aggregated results for N=64 and N=1024, respectively.

We further verified that there is **no systematic bias in the accuracy of the MLP related to helium composition** by analyzing the composition-dependent energy differences between the DFT and N2P2 MLP calculations, as shown in Fig. R2:

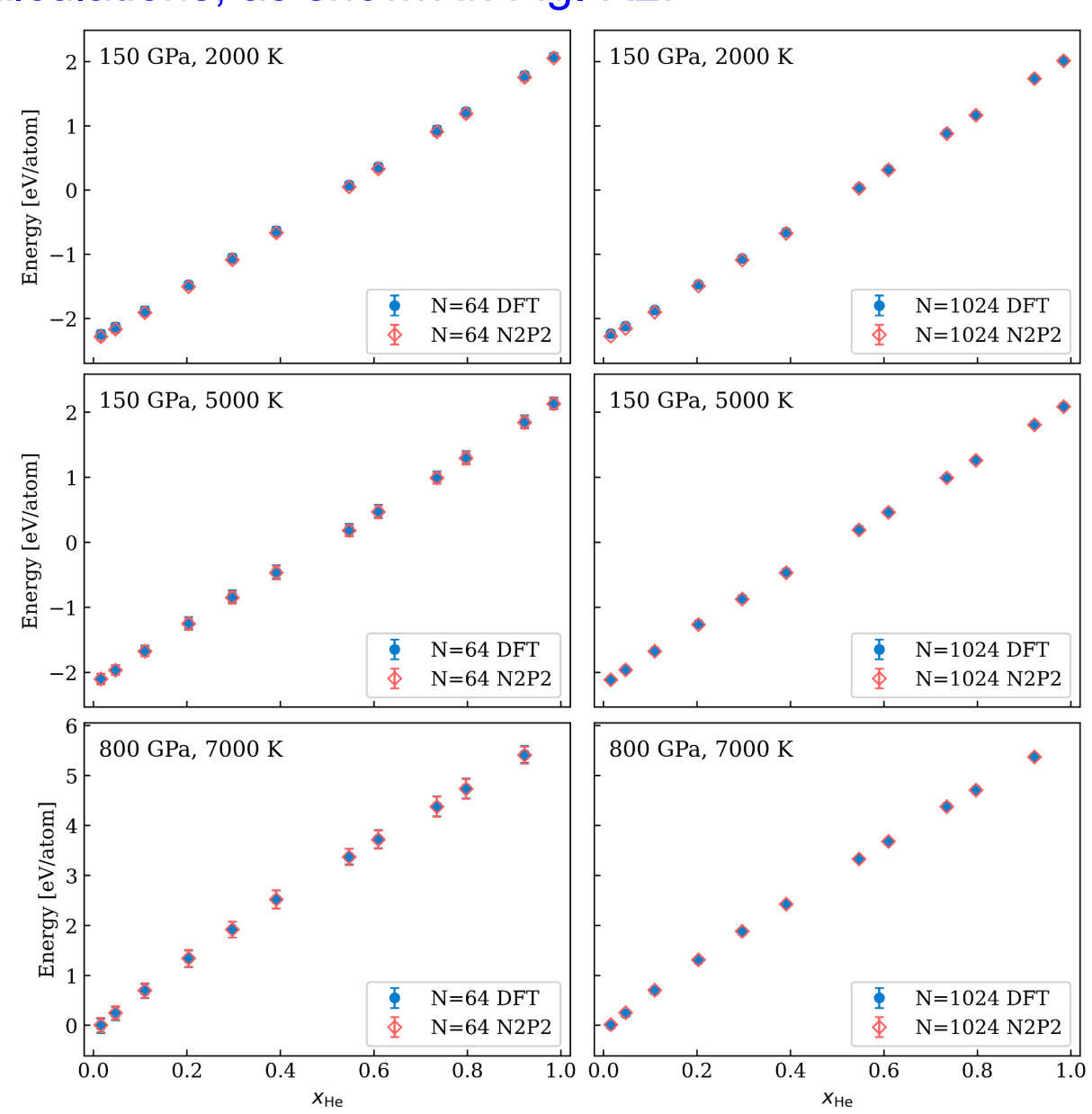

Fig. R2. Per-atom energies derived from vdW-DF DFT and N2P2 MLP as a function of helium fraction, x_{He}, for H/He mixtures at three thermodynamic conditions: 150 GPa and 2000 K, 150 GPa and 5000 K, and 800 GPa and 7000 K. The left and right columns show results for N=64 and N=1024, respectively. For each system size, DFT energies are shown in blue and MLP energies in red. Error bars indicate the standard error of the mean over the 100 sampled configurations.

2) To quantify the **effect of the MLP error on the computed immiscibility temperatures**, we further corrected the MLP-derived Gibbs free energy of mixing ΔG^{mix} to the DFT level using free-energy perturbation (FEP) for the large system with N=1024 based on vdW-DF.

In detail, for each P-T-x_{He} condition with N=1024, we used 100 configurations and computed the energy difference $\Delta U = U_{DFT} - U_{MLP}$. More specifically, U_{DFT} is the DFT energy for a configuration, and U_{MLP} is the corresponding MLP energy evaluated on the same configuration. The free-energy difference for the system at the DFT and the MLP level Δg can then be estimated as:

$$N\Delta g = -k_{\mathrm{B}}T \ln \left\langle \exp\left(-\frac{\Delta U}{k_{\mathrm{B}}T}\right)\right\rangle_{\mathrm{MLP}}$$

Using such free energy correction terms, we can compute the mixing Gibbs free energies ($\Delta G^{mix}(x_{He}) = x_{He}\,\mu_{He} + (1-x_{He})\,\mu_H + \Delta g(x_{He})$) for systems at fixed P, T. As shown in the following plot (Fig. R3), the MLP mixing free energies show overall good agreement with the DFT values at all three conditions (150 GPa, 2000 K; 150 GPa, 5000 K; 800 GPa, 7000 K) that cover atomic and molecular H, mixing and demixing conditions. The largest absolute correction to ΔG^{mix} is observed at the low temperature condition (150 GPa, 2000 K), and the biggest error (at x_{He}=0.54) is still within 10 meV/atom. At the two higher temperatures that are more relevant for the immiscibility boundary, the MLP mixing free energies are essentially on top of DFT values. Even if we take the most pessimistic view and assume that the ΔG^{mix} is on the order of 10 meV/atom, this discrepancy would propagate to the second-order derivative of ΔG^{mix} with respect to x_{He}, $(\partial^2 G/\partial x^2)_{T,P}$, and can potentially shift the helium composition of the demixed phases by less than 0.05. Such a shift may be even smaller when statistical uncertainties are taken into account (see error bars in Fig. R3). Consequently, the DFT-level correction translates to less than 1000 K adjustment in the derived phase boundaries, based on our phase diagrams (Fig. 2(d-f)) reported in the main text. Corrections of this magnitude are not expected to significantly shift the predicted demixing boundary.

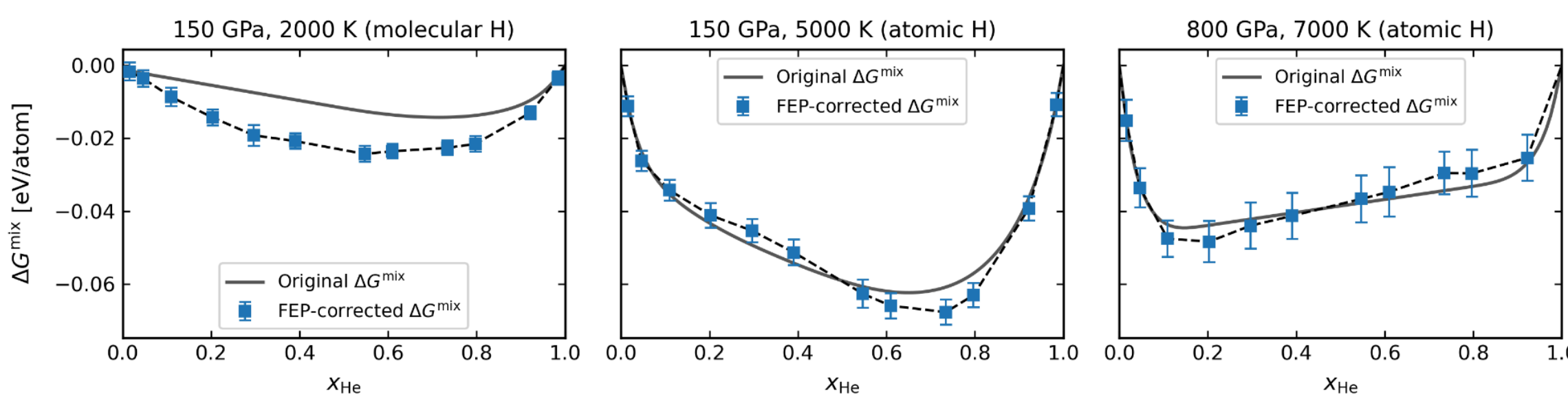

Fig. R3. Per-atom Gibbs free energy of mixing, ΔG^{mix}, as a function of x_{He} for H/He mixtures for three thermodynamic conditions based on vdW-DF: 150 GPa and 2000 K, 150 GPa and 5000 K, and 800 GPa and 7000 K. Gray solid lines show the original MLP results for the N=3456 system, while blue squares denote the FEP-corrected ΔG^{mix}.

2. The authors should try to explain the reason for the discrepancy in their predicted immiscibility temperatures, compared to previous work. For example, if finite size effects are a leading cause, the authors can calculate transition temperatures using system sizes comparable to those used in DFT studies and demonstrate this explicitly. If the reason is the inaccuracy of the estimation of mixing Gibbs free energy, the authors can compare with previous calculations using thermodynamic integration. As it currently stands, it is difficult to gauge the accuracy of the new predictions.

Authors:

The referee suggested that we further explain the discrepancy with previous (computational) work. We assume that the referee was referring to the previous DFT studies (Morales et al., 2010, Lorenzen et al., 2011, Morales et al., 2013, and Schottler et al., 2018) based on AIMD simulations. Such earlier AIMD studies used a relatively small system (N≤64 atoms) and assumed **complete mixing** at all conditions. The **single-phase** assumption enables **the evaluation of ΔG^{mix} for a homogeneous H/He mixture** using the thermodynamic integration method. They performed individual 5th-order RK fits to ΔG^{mix} at each P-T condition, and used common-tangent analyses of the modeled ΔG^{mix} to identify phase separation and the compositions of any demixed phases. At a fixed pressure, these demixing temperatures were combined with the corresponding x_{He} of the demixed phase to map the T-x_{He} immiscibility diagram.

To respond to this, we have further discussed:

1) The direct comparison of our computed ΔG^{mix} with previous AIMD calculations, together with sensitivity tests of the fitted RK model order and the post-analysis procedures;
2) The effect of MLP errors in the computed immiscibility boundary;
3) The fully mixing assumption in AIMD simulations.

As a result, we found that the discrepancy in the predicted immiscibility boundaries originates from the large difference in the derived ΔG^{mix}. Moreover, the fully mixing assumption applied in prior AIMD simulations may not be strictly accurate across all thermodynamic conditions. A detailed discussion follows:

1) In Fig. S25, we compared ΔG^{mix} computed from AIMD (https://doi.org/10.18453/rosdok_id00002384) and from our MLP MD studies based on vdW-DF at 400 GPa and 1000 GPa over 3000-10000 K. Such a comparison suggests that the discrepancy in the inferred immiscibility boundary is due to the large difference in the underlying ΔG^{mix} calculations.

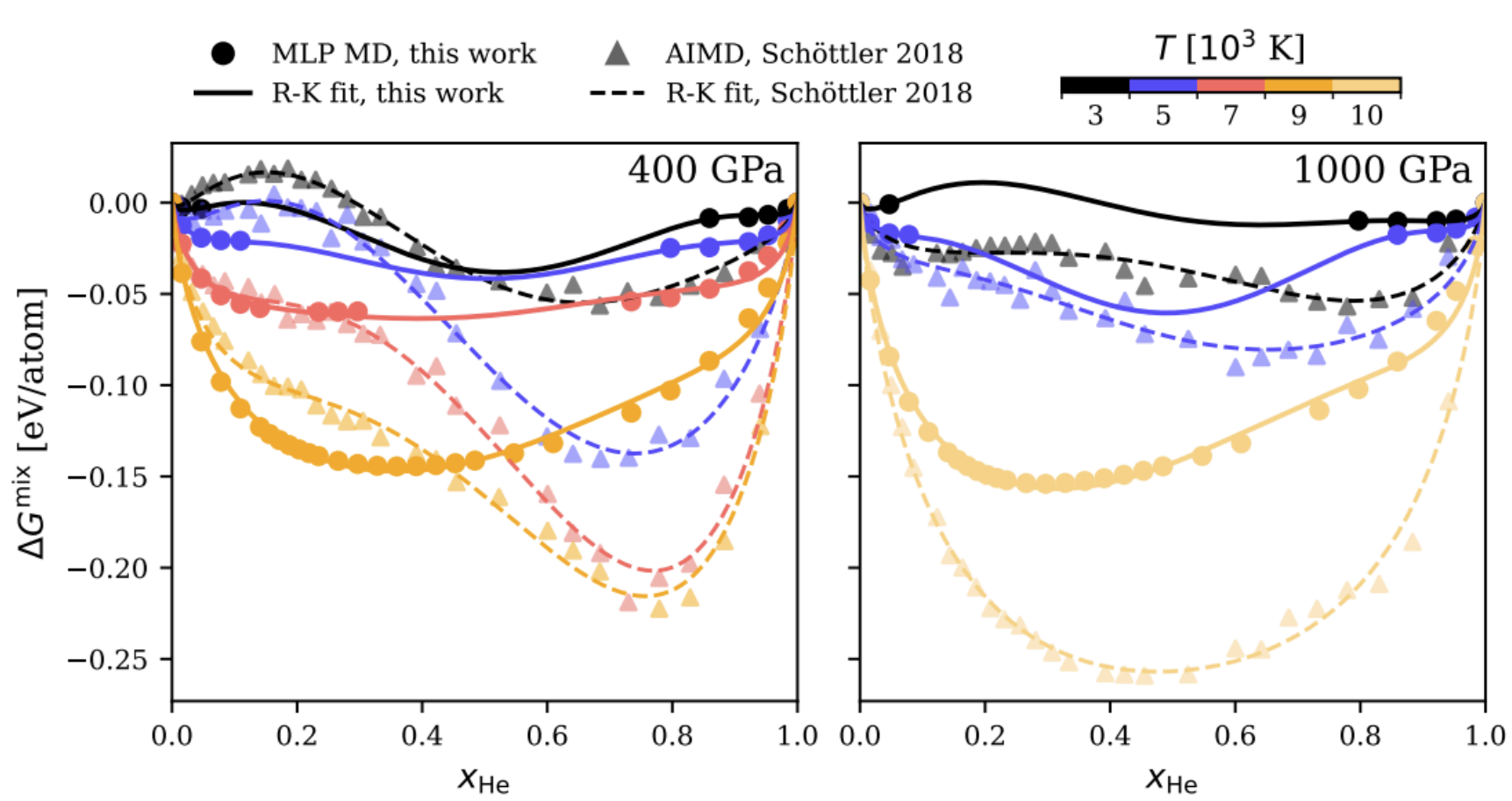

FIG. 25. Comparison of the Gibbs free energy of mixing (ΔG^{mix}) between the MLP MD and AIMD results [8] based on vdW-DF at 400 GPa (left) and 1000 GPa (right) over a range of temperatures. MLP MD results for ΔG^{mix} are reported only for single-phase regions. Note that the AIMD data of ΔG^{mix} at 400 GPa was derived by assuming the ideal entropy of mixing, whereas the data at 1000 GPa was analyzed by including nuclear quantum effects and non-ideal entropy contributions.

To further investigate the cause of the discrepancy and the resulting uncertainty, we further tested the immiscibility boundary derived from 3rd- and 5th-order R-K fits, see Fig. S24:

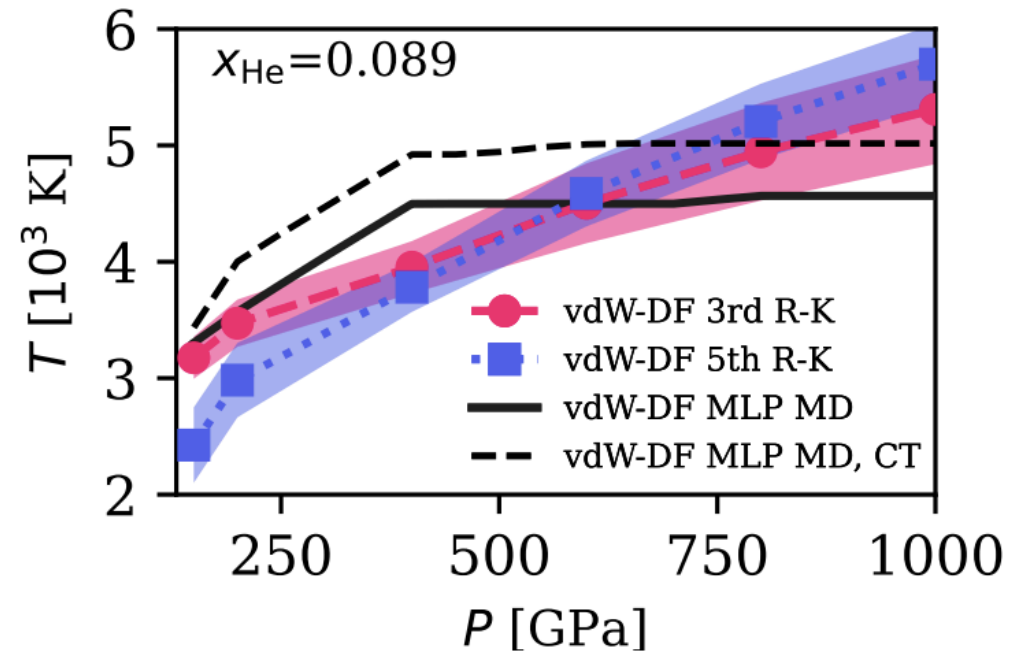

FIG. 24. Comparison of H/He immiscibility boundaries inferred from the third-order (dashed red) and fifth-order (dotted blue) Redlich–Kister fits to vdW-DF MLP MD data. The solid black line denotes the results from direct MLP MD simulations based on the criterion $\left(\partial^2 \Delta G^{\mathrm{mix}}/\partial x_{\mathrm{He}}^2\right)_{T,P} \leq 0$, while the black dashed line denotes the results based on the common-tangent construction.

We found that the 5th-order fit does not meaningfully improve the fit to ΔG^{mix} (see Fig. S23). This is also supported by the fact that the immiscibility boundary inferred from the 5th-order R-K fits (dotted blue line in Fig. S24) shows no better agreement with direct MD results (solid black line) than the 3rd-order model (dashed red line in Fig. S24).
In addition, we find that the immiscibility boundaries determined using the common-tangent construction (dashed black line in Fig. S24) adopted in previous AIMD studies agree well with those obtained from the criterion of $(\partial^2 G/\partial x^2)_{T,P}$ =0, within a difference of 450 K.
These findings demonstrate that varying the order of the R-K model or the post-analysis procedures changes the derived demixing temperature by no more than 1000 K.

2) As detailed in our response to the first comment, we further benchmarked our MLPs and found that the uncertainty of the MLP-derived demixing boundary is within 1000 K relative to DFT values. This accounts for part of the discrepancy between our work and prior work.

3) While it is difficult to ascertain the cause of the discrepancy without fully replicating the previous DFT work, we hypothesize that finite-size effects are the primary cause. In particular, Morales et al. (2013) and Schottler et al.(2018) assumed that the H/He mixtures of 64 atoms are fully mixed. In what follows, we determine how good this assumption is by performing MD simulations for system sizes ranging from N=64 to N=3456 under four P-T conditions:
    a) 150 GPa and 2000 K, molecular H, H/He demixing;
    b) 150 GPa and 3000 K, atomic H, H/He demixing;
    c) 150 GPa and 5000 K, atomic H, fully mixed;
    d) 800 GPa and 7000 K, atomic H, H/He demixing;

   From our MD snapshots, we noted that simulations with a small system size (N=64) do not ensure the fully mixed state. For instance, for a 40-ps MD run for x_{He}=0.30 at 150 GPa and 2000 K with a system size of 64 atoms, we observed phase separation even in small simulation cells:

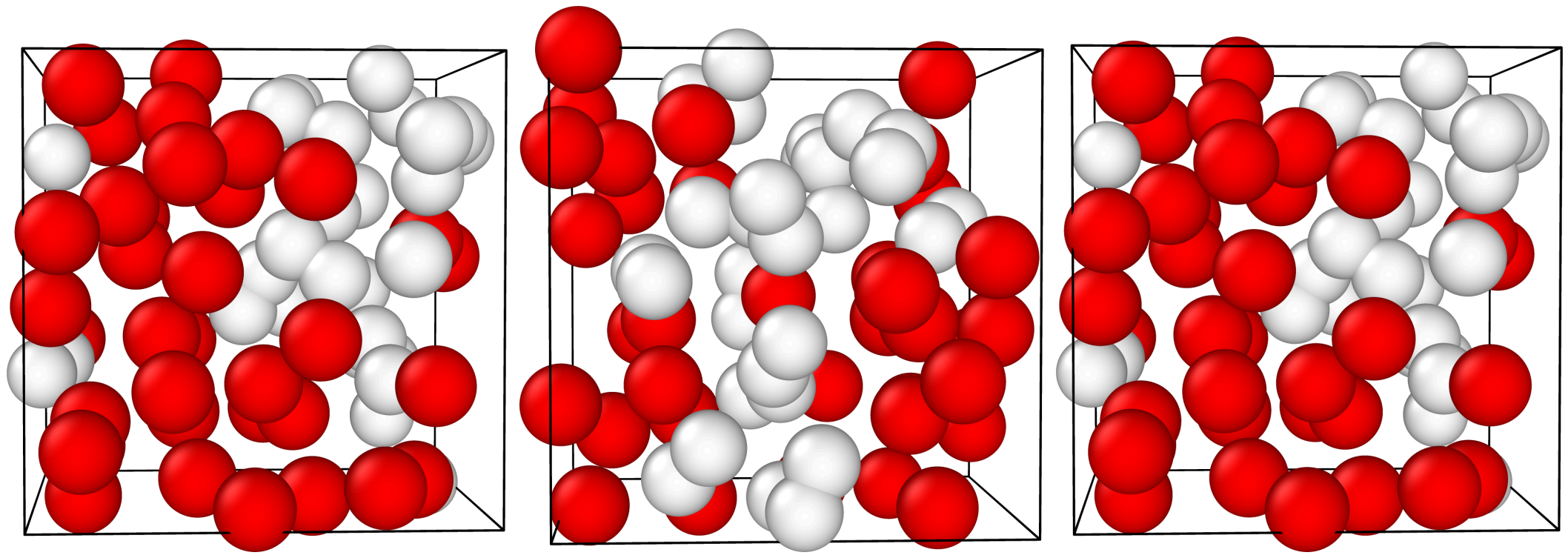

Figure:

We further calculated the radial distribution functions (RDFs) for system sizes ranging from N=64 to N=3456 at 4 thermodynamic conditions:

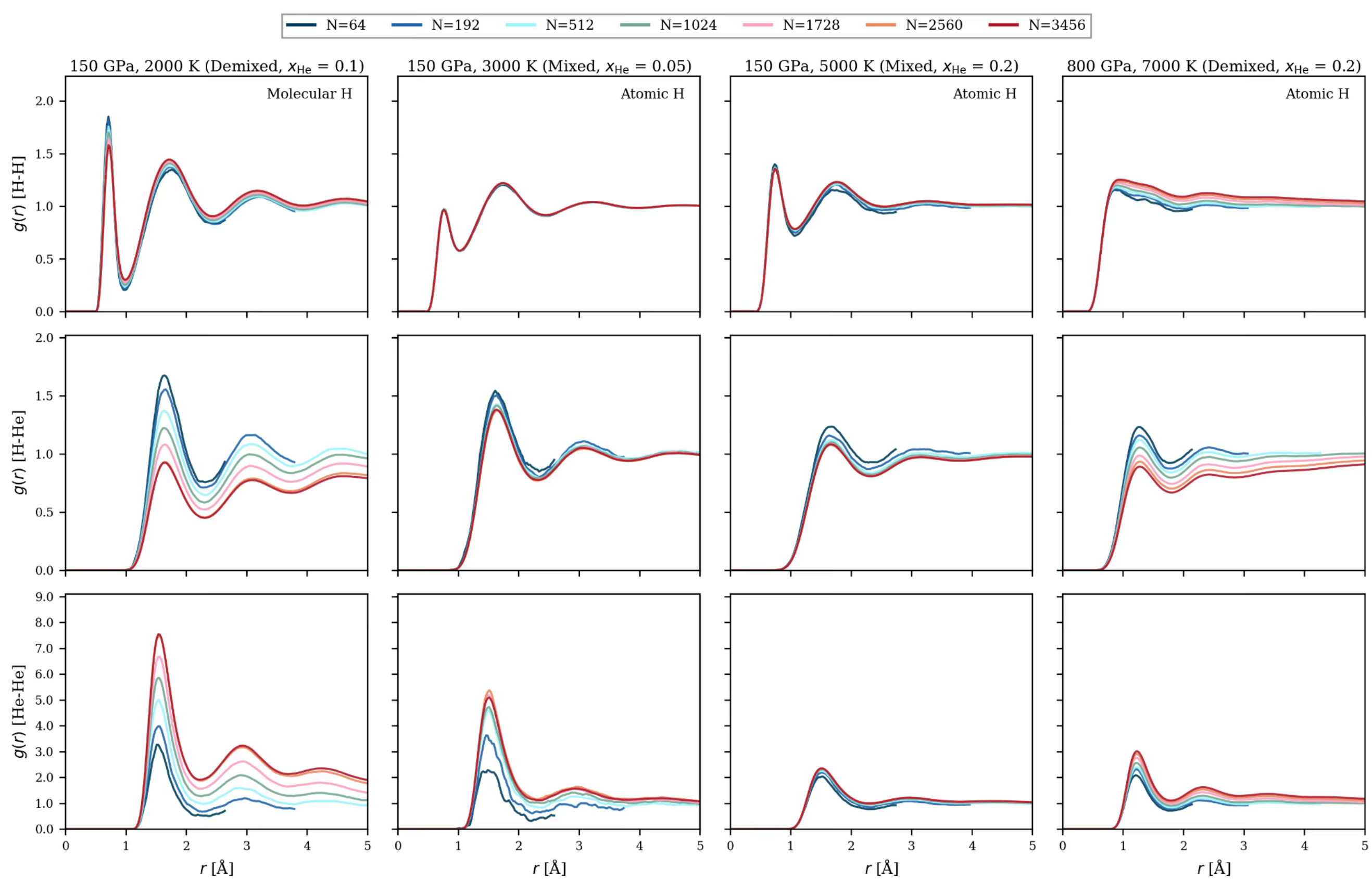

These results demonstrate that, even for small system sizes, the RDFs for N=64 and N=192 are inconsistent, which indicates the assumption of a fully mixed homogeneous system is not strictly valid. Furthermore, we found that, when phase separation occurs, larger system sizes are required to resolve concentration fluctuations at long distances. For example, for a H/He mixture with x_{He}=0.1 at 150 GPa and 2000 K, H/He demixing leads to gHHe<1 and gHeHe>1 at longer distances.

These findings indicate that the fully mixing assumption may be slightly violated even in small-scale simulations, and the calculated ΔG^{mix} may be noised by collective phase separation phenomena.

3. ML potentials appear to have been fitted to DFT calculations with small systems, while calculations of the chemical potentials with the ML potentials used large systems. While the latter addresses the issues of size effects in the calculation of the chemical potential, electronic size effects are typically very large in these conditions and can bias results significantly. This is particularly important in the H-He mixture, since the increase in He induces a metal-to-insulator transition as the He composition increases, which is susceptible to electronic size effects. This in my opinion is a serious flaw in the current work. The authors should address this directly.

Authors:
The referee raised concerns about how the system size of the training set might impact MLP accuracy, especially for capturing the metal-to-insulator transition in H/He mixtures.
To respond to such concerns, we have further discussed:

1) Training MLPs on DFT references based on small-sized systems to run large-scale MD simulations is a robust and standard practice;
2) Additional **benchmark tests of the MLP against DFT** using a large system size of **N=1024** atoms for thermodynamic conditions relevant to the atomic–molecular transition of hydrogen.

As a result, we found that the fitted MLP can reproduce DFT energies for large (N=1024) system sizes, as well as the thermodynamics of high-pressure H/He mixtures at the DFT level. We expand on these points in the detailed discussion that follows:

1) Our approach of training MLPs on DFT calculations based on small-sized cells to simulate larger systems aligns with established standard practices (e.g., https://pubs.acs.org/doi/full/10.1021/acs.chemrev.1c00107). This strategy is specifically designed to overcome the computational limitations of DFT. Moreover, previous MLP studies have successfully utilized small-sized DFT training sets to accurately capture complex physical phenomena in simulations involving thousands of atoms, including high-pressure hydrogen (https://www.nature.com/articles/s41586-020-2677-y, N=128 in the training set; https://journals.aps.org/pre/abstract/10.1103/PhysRevE.111.045307, N=200 in the training set) and H/He mixtures (https://www.nature.com/articles/s41467-024-52868-4, N≤28 in the training set).
2) To directly assess the electronic finite-size effect in our DFT reference data, as discussed above, we evaluated both vdW-DF DFT and N2P2 vdW-DF MLP energies on a dataset of 7000 snapshots at two system sizes, N=64 and N=1024. In particular, these evaluations cover thermodynamic conditions dominated by molecular hydrogen (150 GPa, 2000 K) as well as atomic hydrogen (150 GPa, 5000 K; 800 GPa, 7000 K).

   The comparison shown in the figure below indicates that the DFT energies obtained with N=64 and N=1024 are broadly consistent across all investigated thermodynamic conditions within the error bars. Moreover, the size-dependent DFT energy differences (the second row in the figure) show no clear systematic correlation with either the metal-to-insulator transition or the helium fraction. This verifies that the smaller-cell DFT data can reproduce the energies of high-pressure H/He mixture consistently with large-scale DFT calculations.

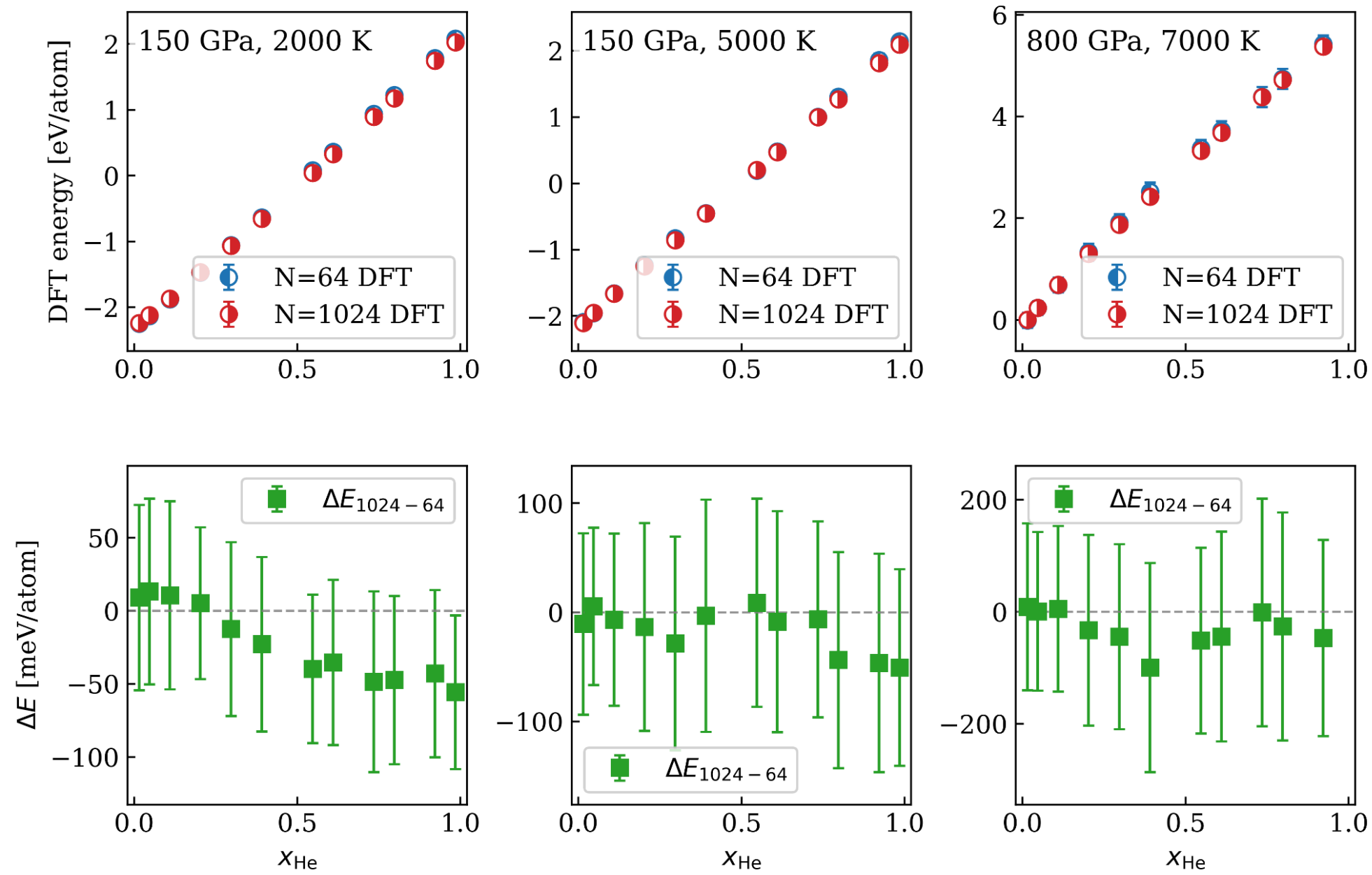

As discussed above for the first comment, we have shown that the fitted MLP can reproduce DFT energies for both small (N=64) and large (N=1024) system sizes:

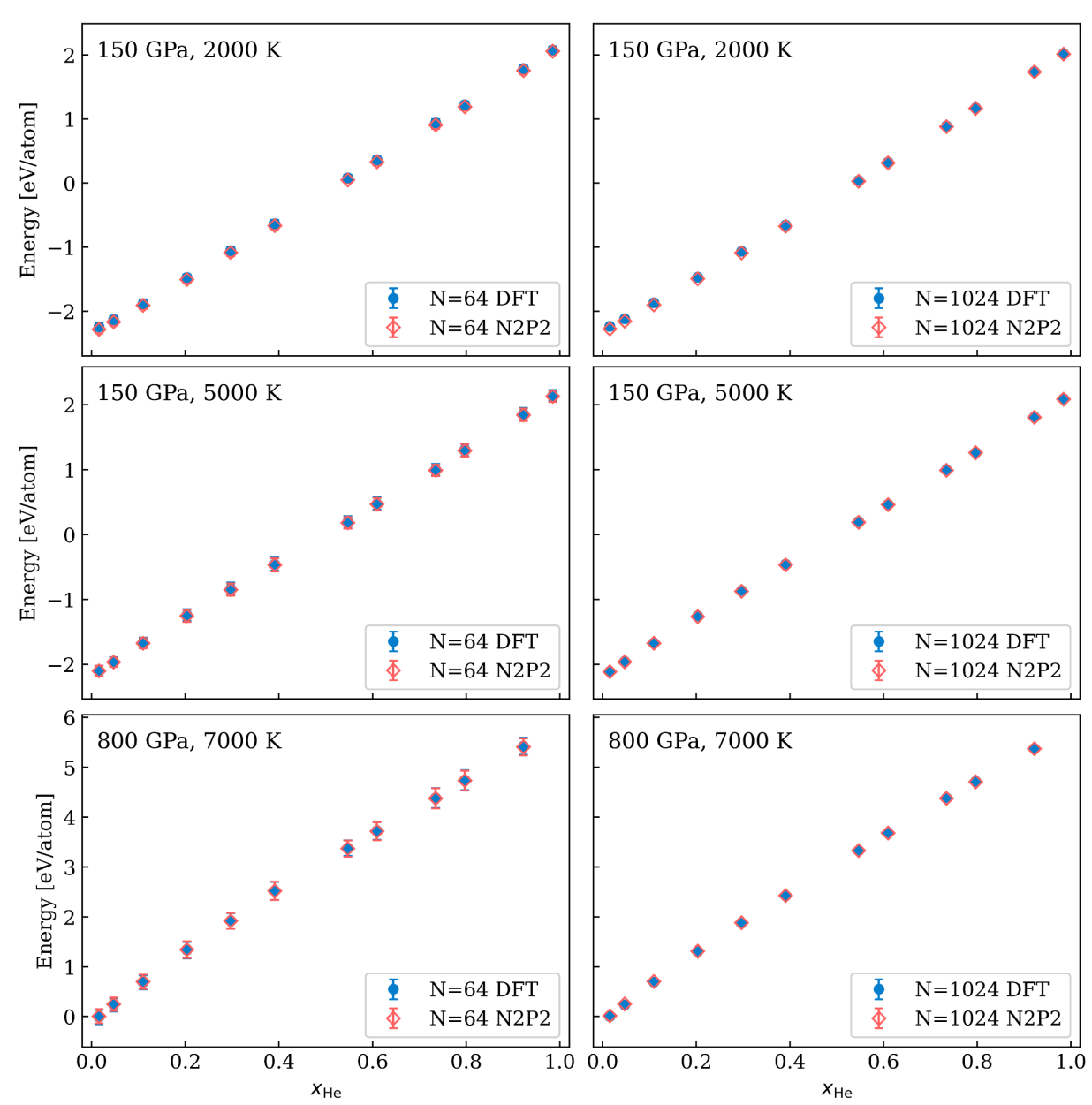

In summary, the predictions made by this article have wide implications in the understanding of the interior structure of hydrogen-rich planets and their approach uses state-of-the-art ML potentials and chemical potential calculations, but without direct evidence demonstrating the cause of the discrepancy with previous work it is difficult to assess the accuracy of the new predictions.

Authors:
We hope the response above provides additional validations of our approach, as well as explanations for the cause of the discrepancy with previous work.

Reviewer #2 (Remarks to the Author):

In this paper the authors calculate the miscibility of helium in hydrogen at very high pressures and temperatures. Before I read the paper, I believed that molecular helium is insoluble in metallic hydrogen, but otherwise they are soluble.

I dont understand helium rain (even though I've read the references). For a general reader, or even high pressure physics specialist, it would be good to have a clear explanation of what this is. Rain on earth is part of the water cycle, which requires precipitation (heavy droplets falling) and evaporation (water rising). If you really mean rain, please (find out) and explain the mechanism by which the helium rises. If you are simply talking about precipitation, use that correct term and explain that it isn't rain. Nobody talks about rain in vinaigrette...

Authors:
We thank the referee for highlighting the need to clarify the physical mechanism of "helium rain" for general audiences. To respond to this comment, we have further discussed:

1) The clarifications of the terminology;
2) The context for the helium rain hypothesis in planetary sciences and astrophysics;
3) The revision of our manuscript in order to clarify the term “helium rain”.

We elaborated on these points in the detailed discussion below:

1) We here used the term “helium rain” in the conventional astrophysical sense (e.g., the review article by Helled et al. (2021); https://www.nature.com/articles/s42254-020-0223-3), which refers specifically to helium phase separation followed by gravitational sedimentation. As the referee correctly pointed out, this is not a cyclic, meteorological process analogous to Earth’s hydrological cycle. Instead, this hypothesis specifically describes a He precipitation and downward settling phenomenon driven by density contrasts within the planetary interior.
2) While "helium precipitation" is indeed an accurate physical descriptor of the phase separation, "helium rain" remains the widely established and standard nomenclature for this planetary mechanism across hundreds of publications in high-pressure physics and planetary sciences. For example, the query of “helium rain” yields 123 results in the Web of Science Core Collection (https://www.webofscience.com/wos/woscc/summary/70a2a592-c8b3-46f2-8336-c2aed0877c6c-01a890a22f/91c6ddb9-0ec3-4018-961e-c3d878ddc81e-01a890a221/relevance/1) and approximately 640 results in Google Scholar (https://scholar.google.com/scholar?hl=en&as_sdt=0%2C5&q=%22helium+rain%22&btnG=), which demonstrates the widespread use of this terminology in the literature.
3) We have now revised the introduction to explicitly clarify this terminology: “In the deep interiors of these planets at hundreds of gigapascals and thousands of Kelvins, **helium is hypothesized to become immiscible in hydrogen and to precipitate as helium-rich droplets that sink toward the planetary core under gravity. This phase separation and subsequent gravitational settling of helium-rich components are commonly referred to as "helium rain"**”.

There are a number of theoretical approximations involved which I will review first.

The Gibbs Free energy diffrence is taken from the S0 method laid out in Prof Cheng's paper "Computing chemical potentials of solutions from structure factors". This claims to extract the gradient of the chemical potential directly from the partial Structure Factors. Although it is not widely used, and it

seems extraordinary to me that it works, it does seem to be robust. Moreover, precise accuracy is not essential for the conclusions of the paper. Given the central role of S(q) in this, it would be good to see the S(q) plotted as well as the g(r) so the reader can see the convergence. Otherwise, I'm happy with S0, although this is a "gut-feeling" and I will be interested in what other reviewers say.

Authors:
We appreciate the referee’s suggestion to examine the convergence of both g(r) and S(k), which provides an important additional validation of our calculations. For such a purpose, we performed a systematic size-scaling test using the vdW-DF N2P2 MLP by computing both RDFs and structure factors S(k) for a wide range of system sizes under four representative thermodynamic conditions.

We therefore concluded that the results reported in the main text, which are based on simulations with N = 3456 for S(k) values, are fully converged with respect to system size. A detailed discussion is provided below:

- We have performed a systematic size-scaling analysis using the vdW-DF N2P2 MLP. In particular, we calculated the radial distribution functions (RDFs) and structure factors (S(k)) for system sizes ranging from N=64 to N=3456 under four thermodynamic conditions:
  a) 150 GPa and 2000 K, molecular H, phase separation at x_{He}=0.1;
  b) 150 GPa and 3000 K, atomic H, fully mixed at x_{He}=0.05;
  c) 150 GPa and 5000 K, atomic H, fully mixed at x_{He}=0.2;
  d) 800 GPa and 7000 K, atomic H, phase separation at x_{He}=0.2;

  Based on the computed RDFs and S(k) data shown below, we found that both quantities are generally converged for N>=512 in fully mixed systems (middle two columns). When phase separation occurs, larger system sizes are required to resolve the long-range concentration fluctuations. For example, for the H/He mixture with x_{He} = 0.1 at 150 GPa and 2000 K, demixing leads to $g(r)_{H\text{-}He}$ < 1 and $g(r)_{He\text{-}He}$>1 at long distances. These findings demonstrate the need for a larger simulation cell to accurately capture the corresponding RDFs and S(k).

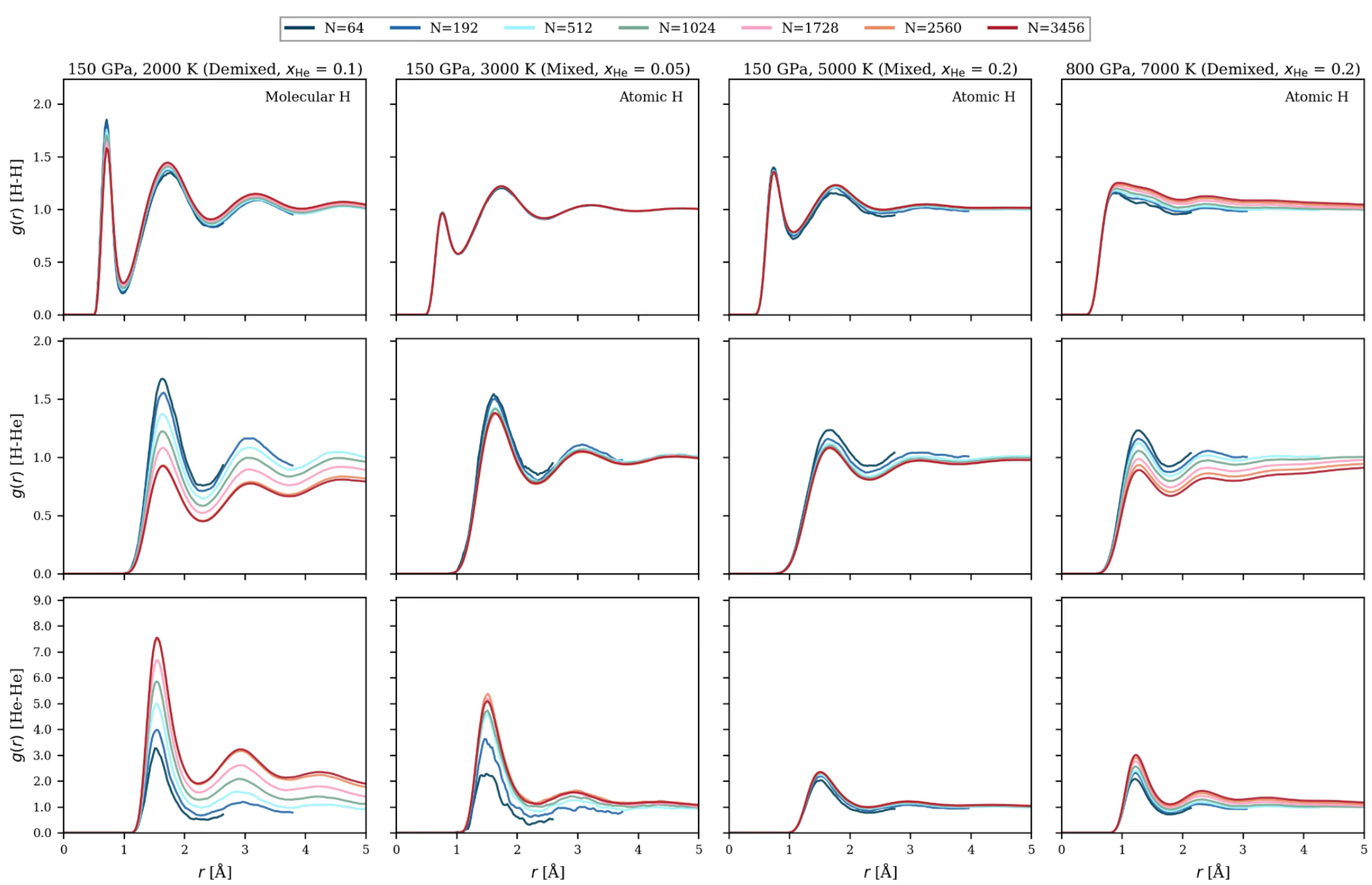

Figure: System-size dependence of the partial radial distribution functions (H-H, H-He, and He-He) from N=64 to N=3456. Each column corresponds to a distinct thermodynamic condition. When phase separation occurs, larger system sizes are required to resolve the long-range concentration fluctuations (e.g., $g(r)_{H\text{-}He} < 1$ and $g(r)_{He\text{-}He} > 1$ at larger r).

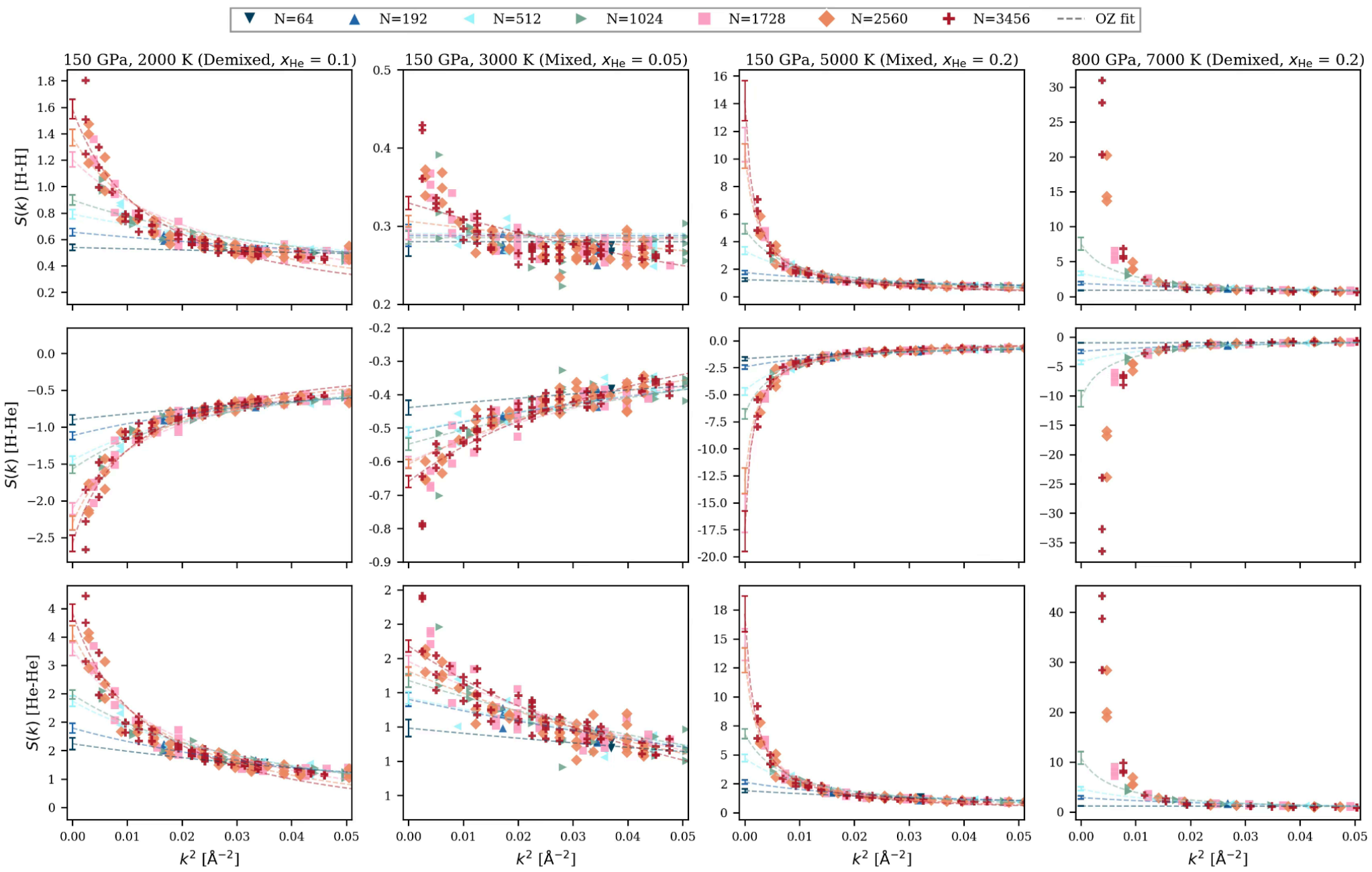

Figure: System-size dependence of the structure factors S(k) for H-H, H-He, and He-He pairs from N = 64 to N = 3456. Each column corresponds to a distinct thermodynamic condition. Dashed lines indicate Ornstein-Zernike (OZ) fits, while error bars at $k^2 = 0$ denote the extrapolated S0 limits.

- Based on the tests above, we have accordingly added the convergence analysis for S(k) and g(r) to the SI section. Moreover, we concluded that the results from the S0 method reported in the main text, which are based on simulations with N = 3456, are fully converged with respect to system size.

Second, a number of DFT calculations were carried out. Admirably, the author used three XC functionals, and Fig S12 shows this is a huge source of uncertainty, moving the atomisation temperature by 1000K between models. I think its safe to assign this to the XC functional rather than the MLIP fit - PBE is fairly well known to stabilise weakly bound molecular hydrogen, often with bonds >1A. We're not shown the hydrogen PBE g(r), and I wonder if this atomisation temperature is very sensitive to the choice of cutoff in Eq S1. Notably, this method and choice would claim that a fluid with 1A covalently bonded hydrogens was atomic. In fig 2, again we see at lower P the PBE calculations give much less solubility (which I assume links to metallization).

Authors:
The referee noted that the hydrogen atomization temperature varies by up to 1000 K across XC functionals and asked whether this uncertainty is primarily due to the XC functional itself or could instead be affected by how atomization is defined in Eq. S1, particularly by the choice of cutoff distance. To address this, we have now:

1) presented the PBE hydrogen RDFs at the relevant thermodynamic conditions, analogous to Fig. S19 (previously Fig. S12);

2) explicitly tested the sensitivity of the atomic-molecular transition temperature to three cutoff distances (1.05 Å, 1.10 Å, and 1.15 Å)

We concluded that the 1.1 Å atomization criterion for hydrogen is suitable for both PBE and vdW-DF. Moreover, the transition temperature depends only weakly on the cutoff choice, with variations below 10%, indicating that the much larger shift of about 1000 K mainly originates from the DFT functional rather than from the atomization criterion or the MLP fit. The details for the analyses are discussed in the following:

1) Analogous to Fig. S19, we plot here the H-H RDFs as a function of temperature at 100 GPa, computed using the PBE N2P2 MLP. These results also show that the 1.1 A criterion for identifying covalently bonded hydrogen remains appropriate for PBE.

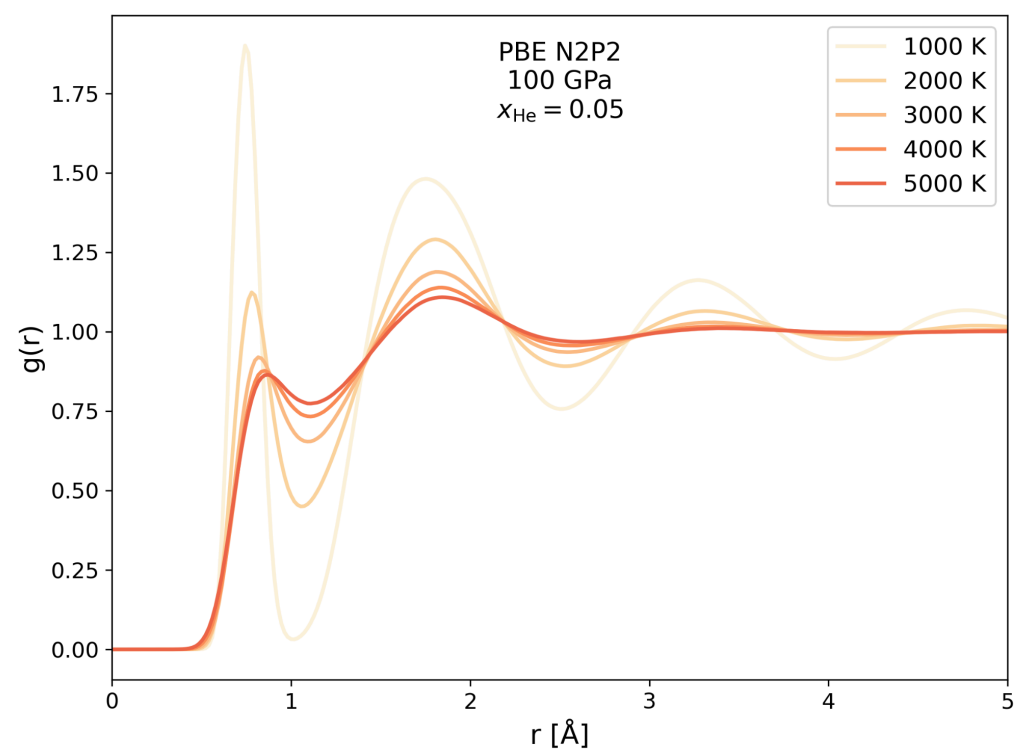

2) We examined the sensitivity of the atomic–molecular transition temperature of hydrogen to the cutoff distance (r_2) used in Eq. S1. In particular, we tested three different criteria (1.05 Å, 1.10 Å, and 1.15 Å) based on vdW-DF and the resulting phase boundaries of hydrogen atomization in a H/He mixture of x_{He} = 0.05 are presented in the following plot:

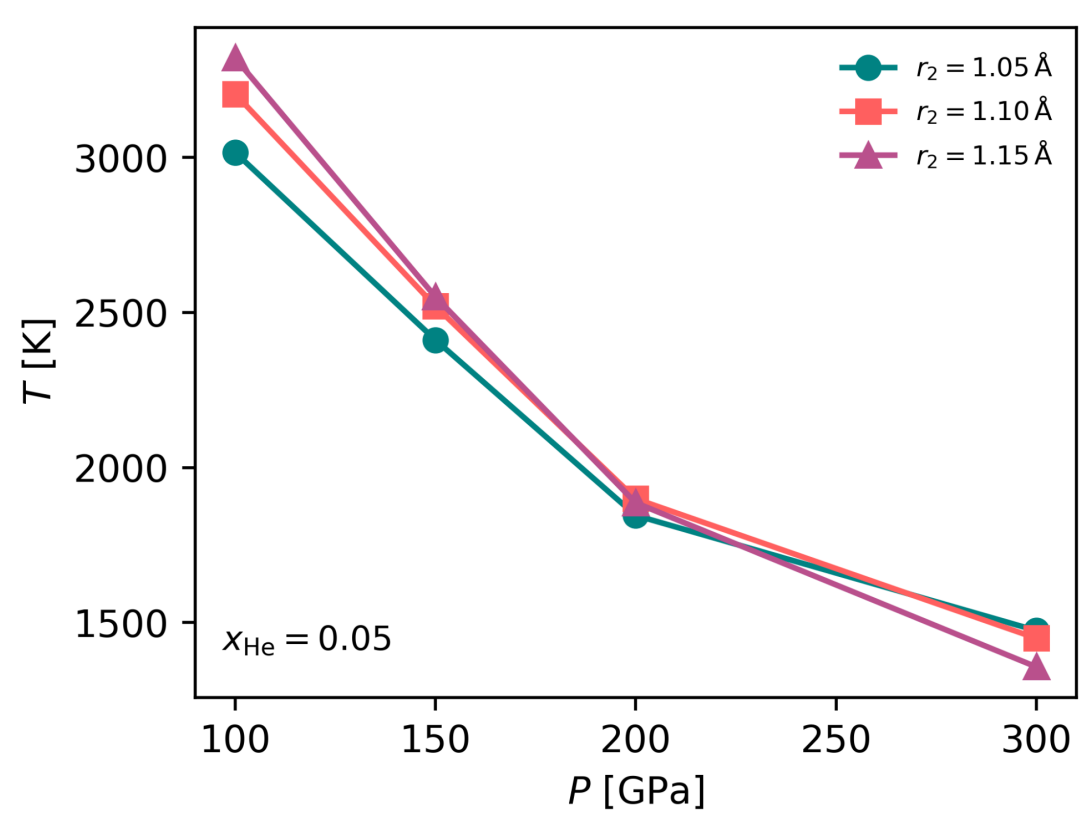

The resulting variation in the transition temperature is less than 10%. This indicates that the atomisation temperature is not very sensitive to the specific choice of cutoff (r_2 =1.10 Å is used in the main). To be consistent with the previous study on pure hydrogen (Cheng et al., 2020), we reported the atomic–molecular transition boundary with a cutoff r_2=1.10 Å in the main.

3) We have added this discussion to the Supporting Information, and the corresponding data have also been uploaded to GitHub.

Third, the DFT data is approximated by machine-learned potentials. Again, the authors use several independent methods trained on exactly the relevant conditions for this work. The MLIPs are therefore interpolating and they should work well. That they give consistent results is encouraging.

So I'm happy that the methods are robust form extending Born-oppenheimer MD through MLIPs to calculate mixing free energy. This is the basis of an excellent paper in a specialist journal. However, Nature Communications is aimed a a general audience and, specifically, requires correct and novel physics as well as accurate methodology

Now we see for Fig 2g that the planetary-relevant (and presumably all) immiscibility boundaries are much lower in temperature than previous thought, either in experiment or straight DFT calculations. This is a strong claim, and a central result certainly novel enough for general audience. But, as a referee, I want to be confident it is correct.

This is especially relevant because the senior author has had this type of problem before, where MLP was used to increase the simulation size beyond what DFT could do, but once the DFT capabilities caught up, there was no sign of the finite size effects that the MLP claimed.
https://www.nature.com/articles/s41586-021-04078-x

Authors:
The referee mentioned our previous Nature 2020 paper on the atomic-molecular phase transition in high-pressure hydrogen (https://www.nature.com/articles/s41586-020-2677-y). The main conclusion of the paper is that there is no first-order transition observed in the liquid phase, and that the critical point is right on the melting line. We also pointed out that some first-order transitions (as evidenced by jumps in density isobars) previously observed in first-principles MD simulations can indeed be attributed to solid-liquid transitions, rather than liquid-liquid transitions.

Based on DFT simulations using large system sizes, Karasiev et al. later show that: 1) at close to the melting line and P≥250 GPa, there is a jump in hydrogen density, and 2) at higher temperatures and lower pressures, the DFT density isobars are sharper compared to the MLP results.

Although this previous work is not directly relevant to this H/He paper, particularly because the temperatures relevant for the current paper are much higher than the H atomic-molecular phase transition temperatures, we would like to take the opportunity to point out that **several recent studies based on MLPs confirmed the conclusions from our 2020 paper**:

https://journals.aps.org/prb/abstract/10.1103/rbsg-r7hx: Small system size in simulations can lead to solid formation at 1200K, 180 GPa, with first-order transition signatures. This is a condition that is commonly associated with liquid-liquid transitions.

https://journals.aps.org/pre/abstract/10.1103/PhysRevE.111.045307: Istas et al. 2025 used NequIP MLP to investigate the liquid-liquid phase transition in hydrogen. They concluded that “We locate the critical point of this transition, the liquid-liquid phase transition, at 1200–1300 K and 155–160 GPa, a temperature lower than most previous estimates and close to the melting transition”.
Although their location of the critical point is not exactly the same as ours, the qualitative conclusion is the same.

https://journals.aps.org/prb/abstract/10.1103/pbrk-3zgd: Tenti et al. 2025 used the MACE architecture for the MLIP. Their conclusion is “Classical and path-integral molecular dynamics driven by this MLIP show

that the LLT is always supercritical above the melting temperature. The position of the corresponding Widom line agrees with previous *ab initio* PBE calculations, which in contrast predicted a first-order LLT. According to our calculations, the crossover line becomes a first-order transition only inside the molecular crystal region. These results call for a reconsideration of the LLT picture previously drawn."

In summary, in our opinion, the controversy regarding the atomic-molecular phase transition in high-pressure hydrogen is not closed yet. MLP will certainly shed more light on this matter in the future, as well as other important problems in high-pressure physics.

Another highly problematic is the lack of any quantum treatment of the protons. The underlying physics of the H-He incompatibility is the metallization of hydrogen which. An extensive body of experimental and theoretical work with diamond anvils and with path integral MD shows there are strong isotope effects in the metallization transition in liquid hydrogen (surprisingly, this work is not referenced).

I believe that if all the calculations here were repeated with deuterium instead of hydrogen, the phase boundaries would be identical (if not, where in the theory does a difference arise?). So the metal/insulator phase boundary is calculated for classical protons which is of theoretical interest only. Again, the authors have experience of full PIMD calculation enabled by MLPs (e.g. https://www.pnas.org/doi/10.1073/pnas.1815117116) where they "reveal the crucial role of nuclear quantum fluctuations in modulating the thermodynamic stabilities of different phases of water"

Authors:
We thank the reviewer for pointing out the relevant work on isotope effects on high-pressure H/He mixtures. We agree that nuclear quantum effects (NQEs) can play a role in high-pressure H/He systems, including both the hydrogen metallization and demixing boundary. To address this, we performed additional ring-polymer path-integral molecular dynamics (PIMD) simulations using our vdW-DF N2P2 MLP to explicitly quantify the impact of NQEs on:

1) the hydrogen atomic-molecular transition
2) the H/He demixing free energy and hence the immiscibility boundary

We find that inclusion of NQE for high-pressure H/He mixtures lowers the hydrogen atomic-molecular transition temperature by up to about 450 K, but has only a minor effect on enhancing H/He immiscibility. As a result, NQEs do not alter the main conclusions of the present work.

Details of PIMD simulations are discussed below:

1) To address NQEs in H/He mixtures, we performed PIMD simulations via the i-PI interface coupled with LAMMPS, using the vdW-DF N2P2 MLP. In particular, we employed eight beads over the investigated temperature range in PIMD simulations, based on convergence benchmarks in prior work (e.g., Hinz et al., 2020, and Van De Bund et al., 2021). Consistent with the previous PIMD protocol for high-pressure hydrogen, each PIMD simulation begins with a 10-ps equilibration in the NPT ensemble, followed by an 80-ps production run in the NVT ensemble. During the NPT equilibration stage, pressure is controlled using the isotropic stochastic barostat, with a relaxation time of 500 fs. Temperature is controlled using the stochastic path integral Langevin equation thermostat (PILE_L) with a relaxation time of 50 fs. A system size of N=2560 and a timestep of 0.2 fs were selected to ensure numerical convergence of MD simulations and reduce the computational cost.

2) **NQEs on the atomic–molecular transition temperature of hydrogen.** For an H/He mixture with x_{He} = 0.05, inclusion of NQEs lowers the atomic–molecular transition temperature of hydrogen by up to 450 K, as shown in the following table and figure:

TABLE V. The atomic–molecular transition temperature of hydrogen T_{AM} in a H/He mixture of x_{He} = 0.05 from MD and PIMD at 150 and 300 GPa, using the vdW-DF N2P2 MLP.

Method	T_{AM} at 150 GPa	T_{AM} at 300 GPa
MD	2520 K	1449 K
PIMD	2190 K	993 K

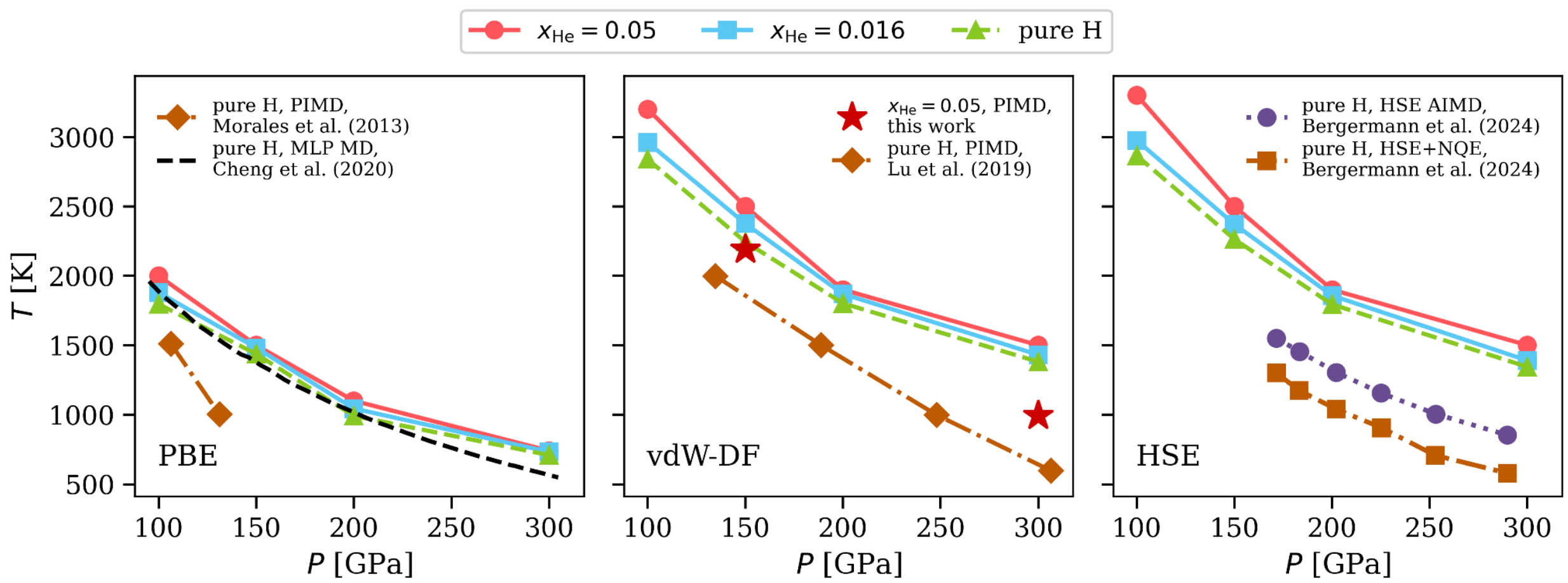

Figure: Comparison of the atomic-molecular transition temperatures of hydrogen for H/He mixtures with x_{He} = 0.05 and x_{He} = 0.016, and for pure hydrogen, based on PBE (left), vdW-DF (middle), and HSE (right). Red stars represent the transition temperatures with x_{He} = 0.05 from our PIMD simulations based on the vdW-DF N2P2 MLP at 150 and 300 GPa. The dashed black line denotes the transition boundary for pure hydrogen reported in the previous PBE MLP study (Cheng et al., 2020). The purple dotted line indicates the transition boundary for pure hydrogen in an earlier AIMD study (Bergermann et al., 2024). Brown dot-dashed lines correspond to phase boundaries of pure hydrogen with NQEs reported by previous DFT-based studies (PBE: Morales et al., 2013; vdW-DF: Lu et al., 2019; HSE: Bergermann et al., 2024).

Such a decrease in the atomic-molecular transition temperatures of hydrogen mostly originates from the zero-point energy (ZPE) in molecular hydrogen, which weakens the covalent bond and promotes molecular dissociation. This finding is consistent with previous DFT-based (Morales et al., 2013; Lu et al., 2019; Hinz et al., 2020; Van De Bund et al., 2021; and Bergermann et al., 2024) and MLP-based studies (Tenti et al., 2025) of dense hydrogen that explicitly account for NQEs.

3) To assess **the effect of NQEs on H/He demixing**, we conducted both classical MD and PIMD simulations under three thermodynamic conditions relevant to planetary conditions: 1) 150 GPa and 2000 K, corresponding to molecular hydrogen; 2) 150 GPa and 3000 K, corresponding to atomic hydrogen; and 3) 400 GPa and 2000 K, corresponding to atomic hydrogen. At each P-T condition, we varied x_{He} from 0.016 to 0.98 across at least 24 compositions.

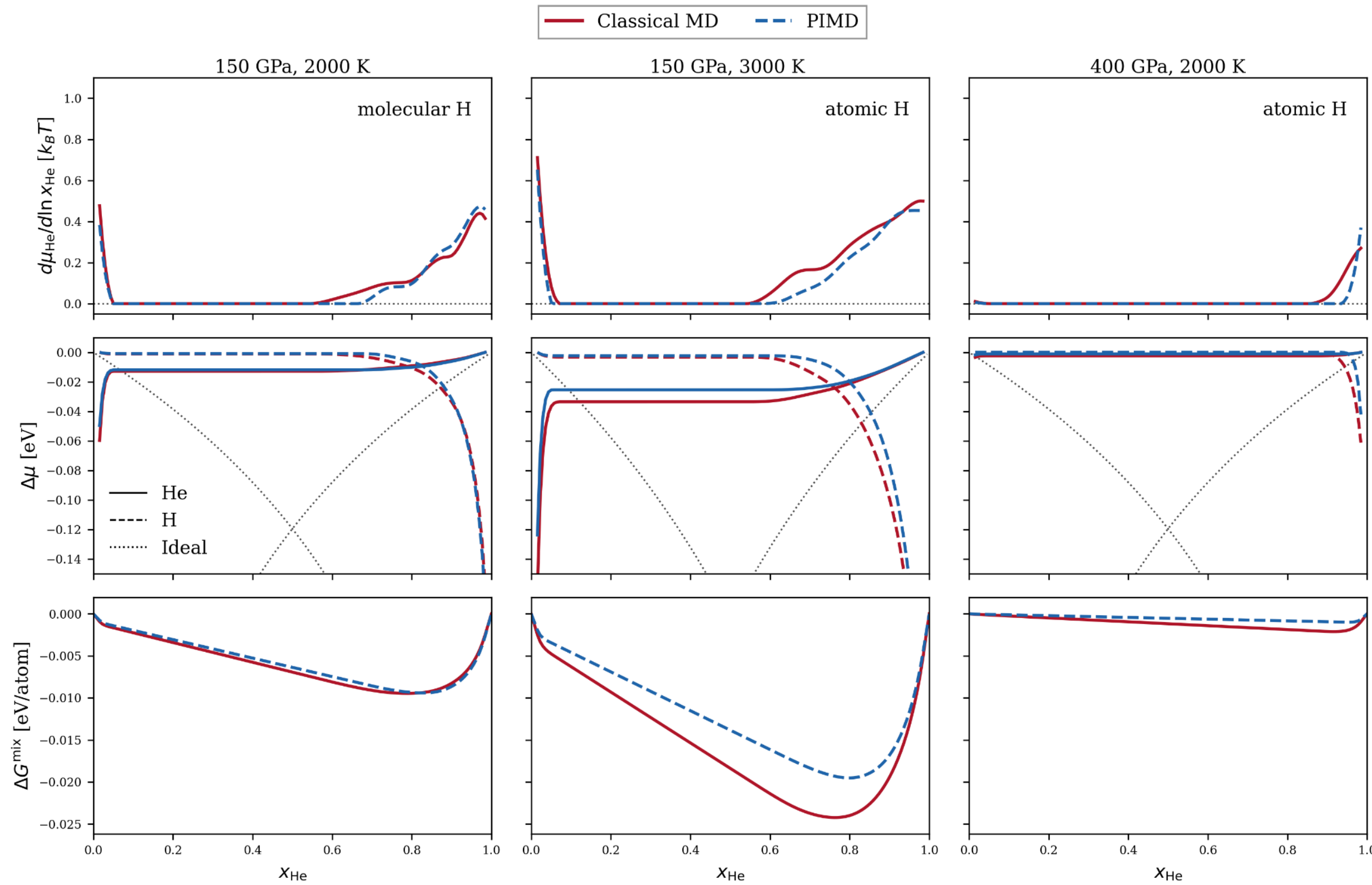

Figure: Thermodynamic behaviors of H/He mixtures predicted by the vdW-DF N2P2 MLP with classical MD (red) and PIMD simulations (blue). Each column represents a distinct thermodynamic condition: 1) 150 GPa and 2000 K (molecular H), 2) 150 GPa and 3000 K (atomic H), and 3) 400 GPa and 2000 K (atomic H).

As shown above, inclusion of NQEs (blue dashed lines) systematically increases ΔG^{mix} and thus enhances the H/He immiscibility. This aligns with previous DFT findings that NQEs remain positive at lower pressures (https://doi.org/10.18453/rosdok_id00002384). The estimated maximum NQE of 6 meV/atom at 150 GPa and 3000 K agrees with the 10 meV/atom upper bound previously reported at 100 GPa and 3000 K. As a result, inclusion of NQEs has only a minor effect on enhancing H/He immiscibility.

To sum up, we performed PIMD simulations to explicitly include NQEs in high-pressure H/He mixtures and found that they shift the hydrogen atomic-molecular transition to lower temperatures and enhance H/He demixing. We have revised the manuscript accordingly and added the relevant prior references.

So, while I accept this is careful and extensive technical work, I'm not convinced by the headline conclusion and I dont recommend publication in a general interest journal like Nature Comms.

Reviewer #2 (Remarks on code availability):

No code was given in the submission.
I am familiar with, and regularly use, the community codes used, VASP, LAMMPS ASE

Authors:
We have uploaded all data necessary to reproduce our results, including the training set, the trained MLP model, and the input files used for training, to the following GitHub repository: https://github.com/ChengUCB/HighPressure_HHe.

Reviewer #3 (Remarks to the Author):

The manuscript by Wang, Hamel and Cheng is a very thorough calculation of the immiscibility boundary in a hydrogen-helium mixture. They use DFT calculations, machine learning models and extensive molecular dynamics to make the best estimate to date of the phase diagram. They then discuss the implications for the gas giant planets, Jupiter and Saturn. As their extensive references document, this scientific question has a history extending more than 50 years. However, modern computational technique are reaching the point where definitive answers are within reach. I think the present manuscript is likely to be the best study though I have several reservations that I discuss below. What makes it stand out is that they have employed three different DFT functionals and three different machine learning models. This enables one to get some idea of how sensitive the results are to the underlying assumptions. Their phase diagram (fig. 2) predicts a lower phase separation temperature than experiment and earlier works, but still of crucial importance in modeling Jupiter and Saturn.

I think this work shows definite progress in predicting this phase diagram. There are several major uncertainties however, that suggest to me that the story is not quite finished.

Authors:
We thank the referee for recognizing the implications of our study on planetary models. We have extensively revised the manuscript to further quantify/eliminate the source of uncertainty that the referee pointed out.

-are the DFT functionals and calculations accurate enough for hydrogen and helium in this regime? I am particularly concerned with the dispersion interaction, the finite size effects thermal electron energies in the hydrogen DFT calculation and differences caused by the different energy scales of hydrogen and helium. say from the use of pseudopotentials at high pressure.

Authors:
The referee asked whether our DFT reference calculations are sufficiently accurate for high-pressure H/He mixtures, particularly with respect to the choice of XC functional, dispersion interactions, thermal electronic effects, pseudopotentials, and finite-size effects.
To respond to these concerns, we discuss the following aspects:

1) The accuracy of the selected DFT functionals in this work;
2) The treatment of dispersion interactions in the DFT functionals;
3) The incorporation of thermal electron energies in the DFT calculations and therefore in the fitted MLPs;
4) Possible errors associated with pseudopotentials and the corresponding planewave cutoff at high pressures;

5) Finite size effects in DFT calculations, which we have directly examined based on additional **benchmark tests of DFT calculations** using a large system size of **N=1024** atoms for at least 11 helium compositions across three P-T conditions;
6) The comparison of our derived atomic-molecular transition boundary of hydrogen in H/He mixtures with prior QMC-based references.

We find that DFT calculations align well with existing QMC benchmarks for high-pressure H/He mixtures, although some functional-dependent uncertainties remain. One of the main motivations of this work is to address this uncertainty associated with DFT functional choice by systematically investigating the immiscibility boundaries using three different functionals.

We discuss these points in detail below:

1) The reliability of DFT for dense H/He mixtures depends mainly on the approximations used for the exchange-correlation (XC) functional and the resulting electron density. To assess the possible errors in DFT carefully, we employed **three widely used functionals that reflect different physical approximations: the semilocal PBE functional, the nonlocal dispersion-corrected vdW-DF functional, and the hybrid exact-exchange-based HSE functional. This allows us to evaluate the robustness of our results across different DFT functionals.**

   In particular, PBE is widely used and computationally efficient, but QMC benchmarks have shown that, although it gives a reasonable equation of state, it does not reproduce the H/He mixing enthalpy with sufficient accuracy (Clay et al., 2016; DOI: 10.1103/PhysRevB.93.035121). In contrast, vdW-DF includes nonlocal correlation effects absent in PBE and gives better agreement with QMC for H/He mixing enthalpy (Clay et al., 2016) and hydrogen metallization (Cozza et al., 2026; DOI: https://doi.org/10.1103/yrk6-ryps), although it can overestimate pressures relative to QMC, consistent with our Fig. 2(c) in the main text. The expensive HSE generally provides more accurate energies and forces for high-pressure hydrogen and H/He mixtures because partial exact HF exchange improves the description of hydrogen dissociation and metallization.

[REDACTED]

Figure: QMC benchmark results for mixing enthalpy (left panel), pressure calculations (middle panel), energy calculations (right panel) for different XC functionals at different densities over all helium concentrations, which were taken from Clay et al. (2016) (DOI: 10.1103/PhysRevB.93.035121).

   To clarify this point in the revised manuscript, we have added the following sentence to directly address DFT errors: "**DFT relies on approximations for the exchange-correlation (XC) functionals and electron densities that can cause both functional-driven and density-driven errors.**"

2) Regarding dispersion interactions, this is one of the main reasons we included vdW-DF for this work. Since this local XC functional is enhanced with nonlocal correlation effects, it provides a

more appropriate description of dispersion-related contributions that are not well captured by PBE. The comparison among PBE, vdW-DF, and HSE, therefore, also serves as a practical assessment of the sensitivity of our results to the treatment of dispersion.

3) Thermal electronic effects were directly included in our DFT calculations and are naturally embedded in the fitted MLPs. Specifically, we set the electronic Fermi smearing temperature equal to the ionic temperature in the DFT calculations. In this way, the potential energy surfaces used for training consistently include thermal electron contributions. We have also successfully employed this methodology in our previous work on superionic water (https://www.nature.com/articles/s41567-021-01334-9).
4) As for pseudopotentials and basis-set convergence at high pressure, we agree that this is an important issue, especially for hydrogen. To ensure no pseudopotential overlap at high pressures, we employed the 'hard' version of the hydrogen PAW potential (PAW_PBE H_h 06Feb2004) and the helium PAW potential (PAW_PBE He 05Jan2001) from the VASP library. To fully converge the basis set with these potentials, we used a large planewave energy cutoff of 1200 eV. Furthermore, the Brillouin zone was sampled with a dense k-point grid resolution of at least 0.02 $Å^{-1}$ for the PBE and vdW-DF calculations, and 0.03 $Å^{-1}$ for the HSE calculations. These settings were chosen specifically to minimize errors arising from the different energy scales of hydrogen and helium and from the use of pseudopotentials in the high-pressure regime.
5) To directly assess the finite-size effect in our DFT reference data, we evaluated both vdW-DF DFT and N2P2 vdW-DF MLP energies on a dataset of 7000 snapshots at two system sizes, N=64 and N=1024. In particular, these evaluations cover thermodynamic conditions dominated by molecular hydrogen (150 GPa, 2000 K) as well as atomic hydrogen (150 GPa, 5000 K; 800 GPa, 7000 K). The comparison shown in the figure below indicates that the DFT energies obtained with N=64 and N=1024 are broadly consistent across all investigated thermodynamic conditions within the error bars. Moreover, the size-dependent DFT energy differences (the second row in the figure) show no clear systematic correlation with either the atomic-molecular transition or the helium fraction. This indicates that the smaller-cell DFT data can reproduce the energies of high-pressure H/He mixtures consistently with large-scale DFT calculations.

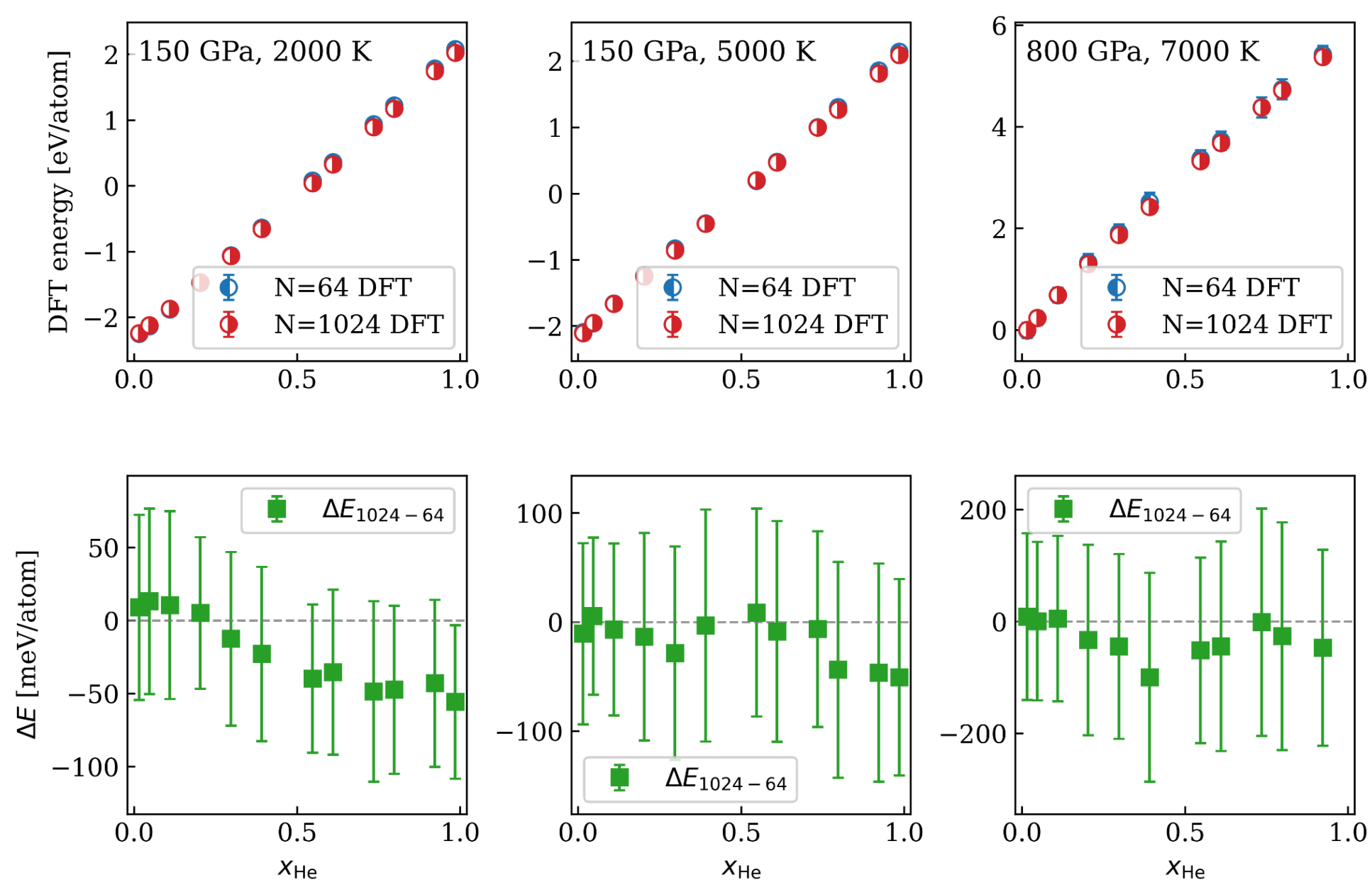

6) We further compared the vdW-DF-MLP-derived atomic-molecular phase transition of hydrogen at xHe=0.05 with available QMC results (Mazzola et al., 2018; DOI:

10.1103/PhysRevLett.120.025701) in the SI, as shown below. The right panel shows that the vdW-DF DFT that we used yields similar transition temperatures to the QMC reference.

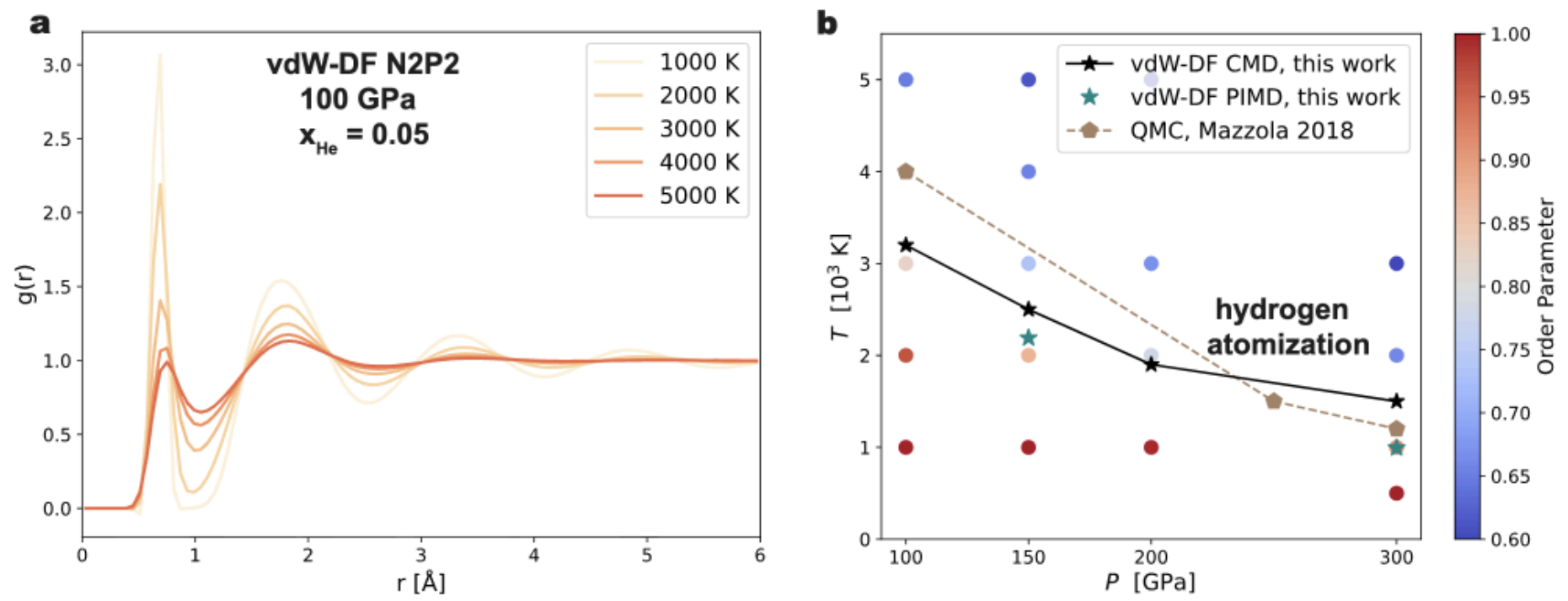

FIG. 18. The atomic–molecular transition of hydrogen in a H/He mixture of $x_{\mathrm{He}} = 0.05$. (a) Temperature dependence of the H-H radial distribution functions (gHH) at 100 GPa based on the vdW-DF N2P2 MLP. The first gHH peak diminishes with increasing temperature. (b) Order parameter $s(r)$ as a function of temperature and pressure. The black curve denotes the phase boundary of hydrogen atomization in a H/He mixture of $x_{\mathrm{He}} = 0.05$, compared with previous quantum Monte Carlo results [17] (brown dashed line) for $x_{\mathrm{He}} = 0.085$.

-are the machine learning models adequate and indeed even necessary? In the introduction they say "DFT studies are constrained to system sizes of several hundred atoms...finite size effects can be significant for phase transition.." Although such a statement is the accepted wisdom for some phase transitions, I question whether size effects are important here. It would seem to me that if one does careful free energy calculations of homogenous helium and hydrogen phases and then carefully studies the changes when a few helium atoms are introduced into the hydrogen and vice versa, one should be able to get an accurate idea of at least the low and high concentration sides of the phase separation. using hundreds of atoms, thus avoiding the necessity of the machine learning approximation. Such a check would be more convincing. The pair correlation functions shown in the SI are informative but what is needed are the actual energetic and pressure comparison between the ML models and the DFT calculations.

Authors:

First, the referee asked about the importance of the finite size effects in phase transitions. Second, the referee also asked for direct energetic and pressure comparisons between the MLPs and their DFT references.
We first address the question about finite-size effects and whether the MLP is necessary:

1) We agreed that free-energy calculations in small DFT cells can be informative, but only in the limit that He is dilute and the interactions between He atoms are less important. However, the helium molar fraction relevant to planetary conditions is typically around 10% in Gas Giants and can be

even beyond 50% in Saturn (e.g., Howard et al., 2024, https://doi.org/10.1051/0004-6361/202450629; Sur et al., 2025, https://doi.org/10.3847/1538-4357/ae16a3), so it cannot be treated as a low-concentration case. Composition-dependent non-ideal mixing effects must therefore be taken into account

2) Near H/He demixing, collective concentration fluctuations cannot be neglected and fully captured by small-sized simulations. To further demonstrate the finite-size effect for H/He demixing, we have performed a systematic size-scaling analysis using the vdW-DF N2P2 MLP. We calculated the radial distribution functions (RDFs) for system sizes ranging from N=64 to N=3456 under four thermodynamic conditions:
    a) 150 GPa and 2000 K, molecular H, H/He demixing;
    b) 150 GPa and 3000 K, atomic H, H/He demixing;
    c) 150 GPa and 5000 K, atomic H, fully mixed;
    d) 800 GPa and 7000 K, atomic H, H/He demixing;

   As discussed in our response to the second comment of Reviewer #1, the MD snapshots show that H/He demixing already occurs for x_{He}=0.30 at 150 GPa and 2000 K in a 64-atom cell. Our simulation results (see the figure below) also demonstrate that RDFs for N=64 and N=192 are inconsistent, which indicates the assumption of a fully mixed homogeneous system is not strictly valid for small-scale simulations. Furthermore, when phase separation occurs, larger system sizes are required to resolve concentration fluctuations at long distances. For example, for a H/He mixture with x_{He}=0.1 at 150 GPa and 2000 K, H/He demixing leads to gHHe<1 and gHeHe>1 at longer distances.

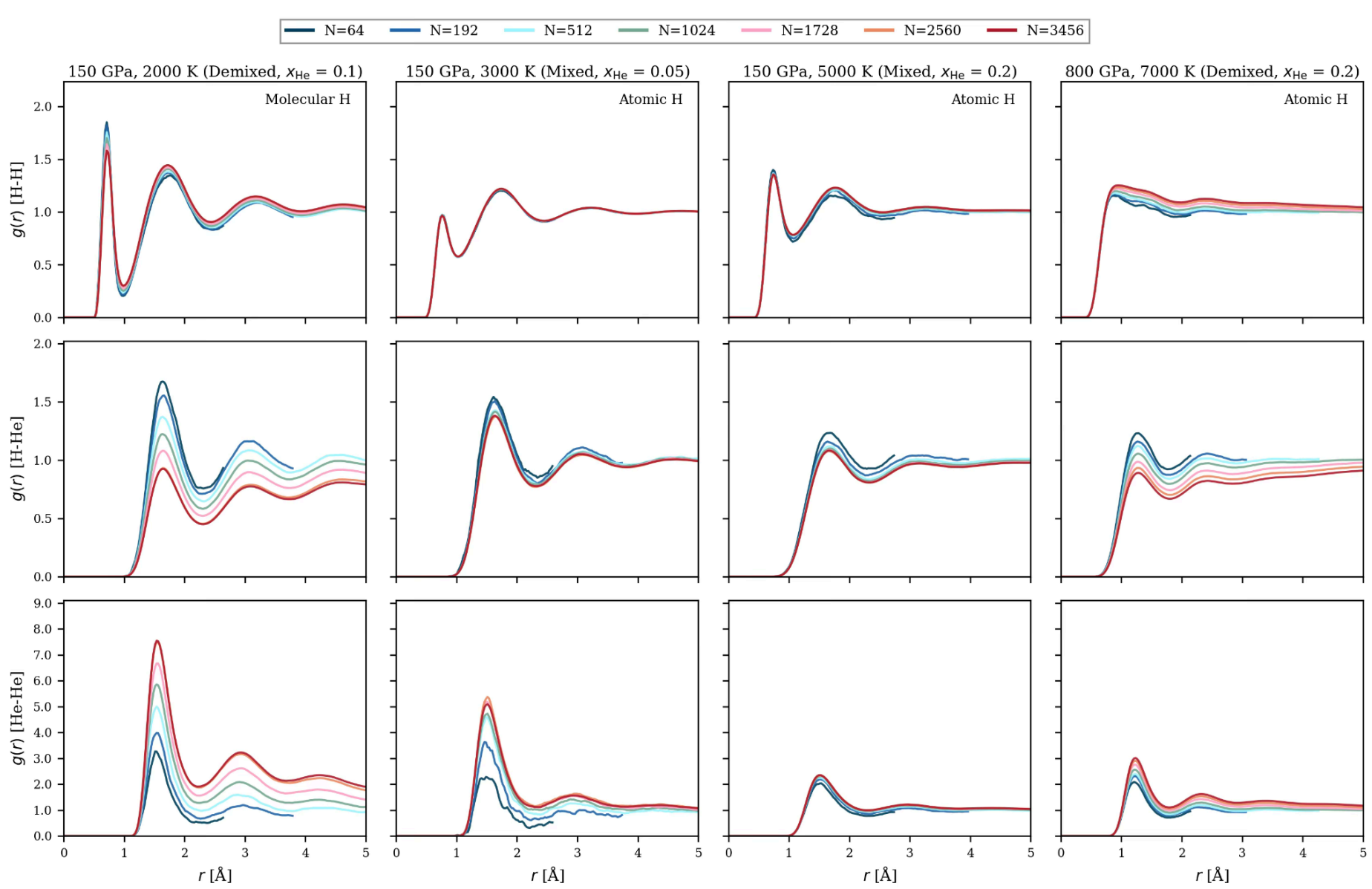

   Moreover, based on the S0 method and the resulting free-energy calculations, we find that the mixing free energy requires larger system sizes (N≥1024) to achieve convergence. These results highlight why MLPs are essential in the present work: they enable the large-scale MD simulations needed to study H/He demixing with converged simulations.

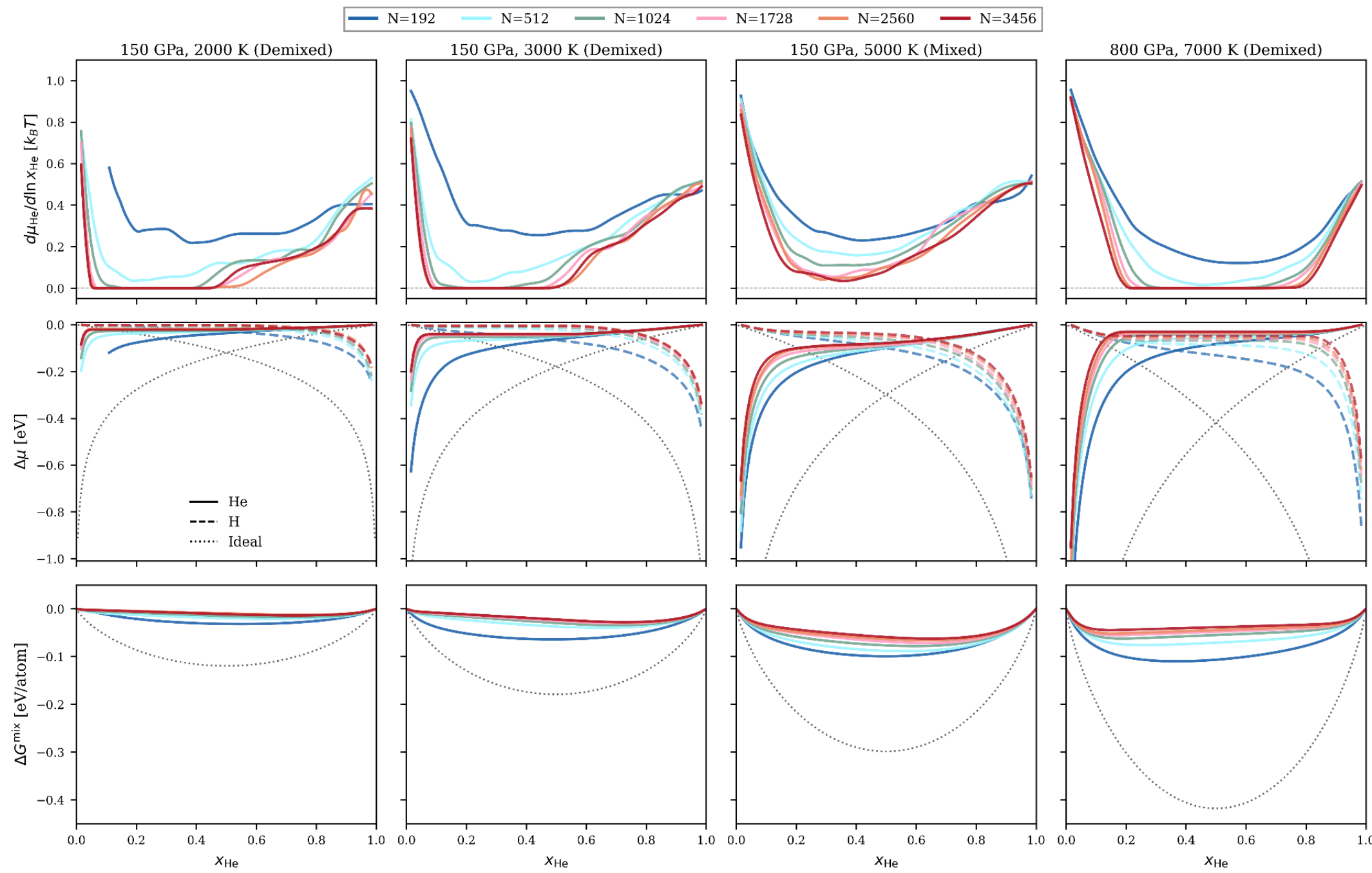

We then address the second question about direct energetic and pressure comparisons between the MLPs and DFT:

1) As for the pressure comparison between MLP and DFT results, in Fig. S8 we compared the density as a function of pressure for seven H/He mixtures (xHe = 0.125, 0.25, 0.375, 0.5, 0.625, 0.75, and 0.875) at five temperatures (T = 2000, 3000, 4000, 6000, and 10000 K), and found a good agreement between the MLP and DFT calculations.

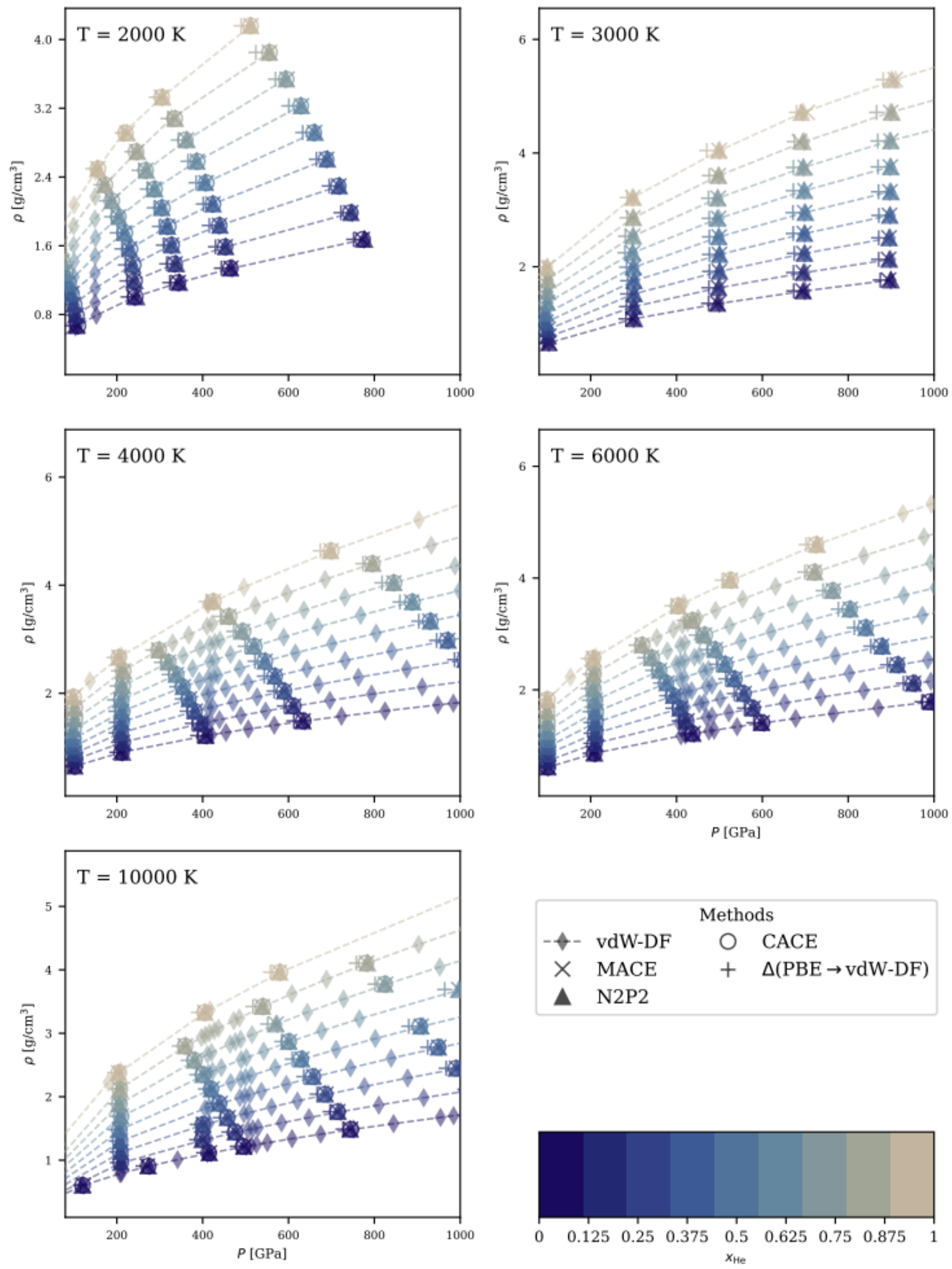

FIG. 8. Density (ρ) as a function of pressure (P) for seven H/He mixtures (x_{He} = 0.125, 0.25, 0.375, 0.5, 0.625, 0.75, and 0.875) at five temperatures (T= 2000, 3000, 4000, 6000, and 10000 K), predicted by vdW-DF DFT (dashed lines) and four vdW-DF MLPs (markers). The results are from NVT MD simulations of a system with 64 electrons (32-64 atoms).

2) To demonstrate that the MLP can accurately reproduce DFT energies and pressures, we evaluated both vdW-DF DFT and N2P2 vdW-DF MLP energies and pressures on a dataset of 7000 snapshots. For each system size (N=64 and N=1024), these configurations cover 12 helium compositions (x_{He} = 0.016, 0.047, 0.11, 0.20, 0.30, 0.39, 0.55, 0.61, 0.73, 0.80, 0.92, and 0.98) for 2000 K and 5000 K at 150 GPa, and 11 compositions (x_{He}= 0.016-0.92) at 800 GPa and 7000 K. Note that the reported pressure excludes the kinetic contribution.
As shown in the figures below, **the DFT and MLP energies are in close agreement for both system sizes** across the investigated P-T-x_{He} space, with R^2=0.9999. The MLP pressures differ from the DFT references by up to 2.5 GPa only. This discrepancy does not affect the predicted immiscibility temperatures because the pressure grid was sampled at intervals of at least 50 GPa.

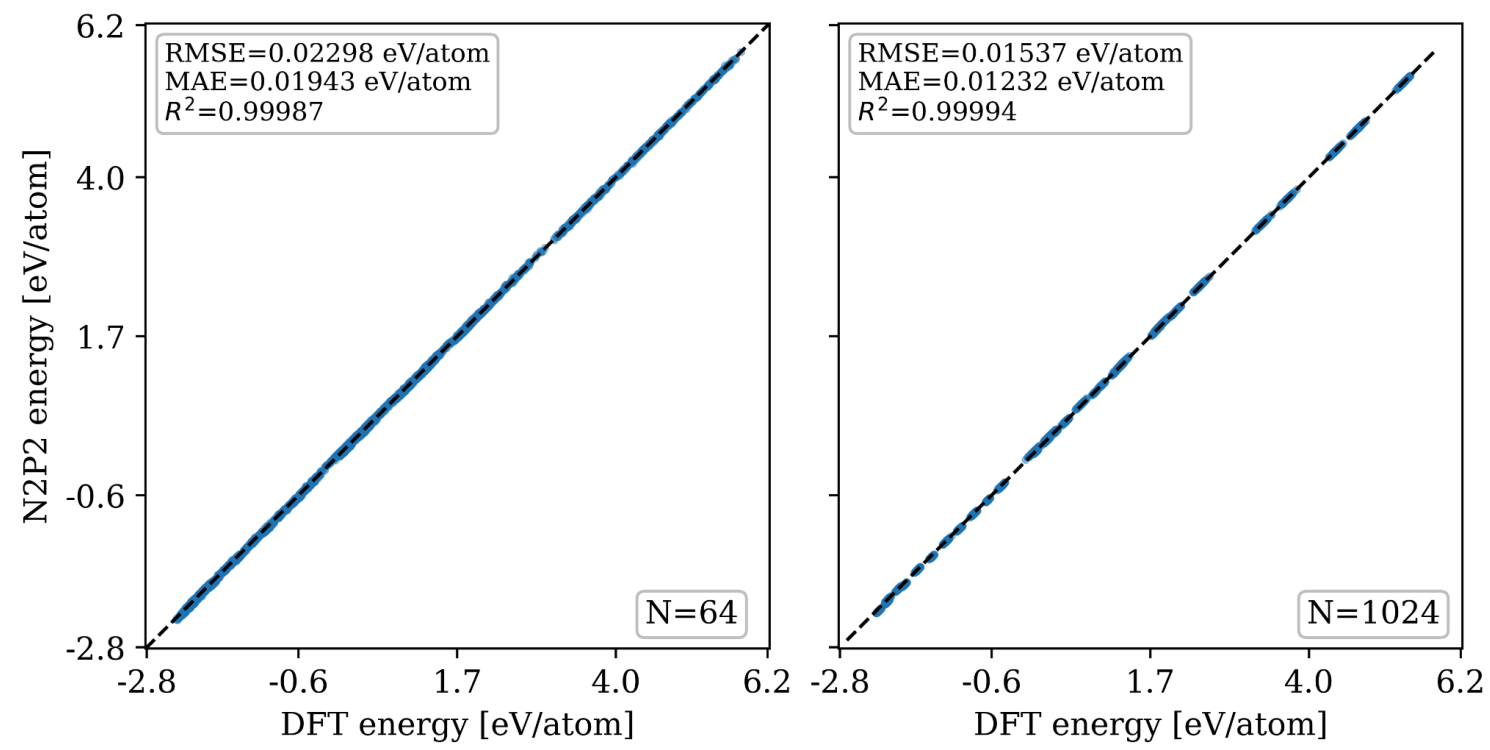

Figure: Parity comparison of per-atom energies predicted by the N2P2 MLP against vdW-DF DFT reference energies for H/He mixtures at 150 GPa and 2000 K, 150 GPa and 5000 K, and 800 GPa and 7000 K. The left and right panels show the aggregated results for N=64 and N=1024, respectively.

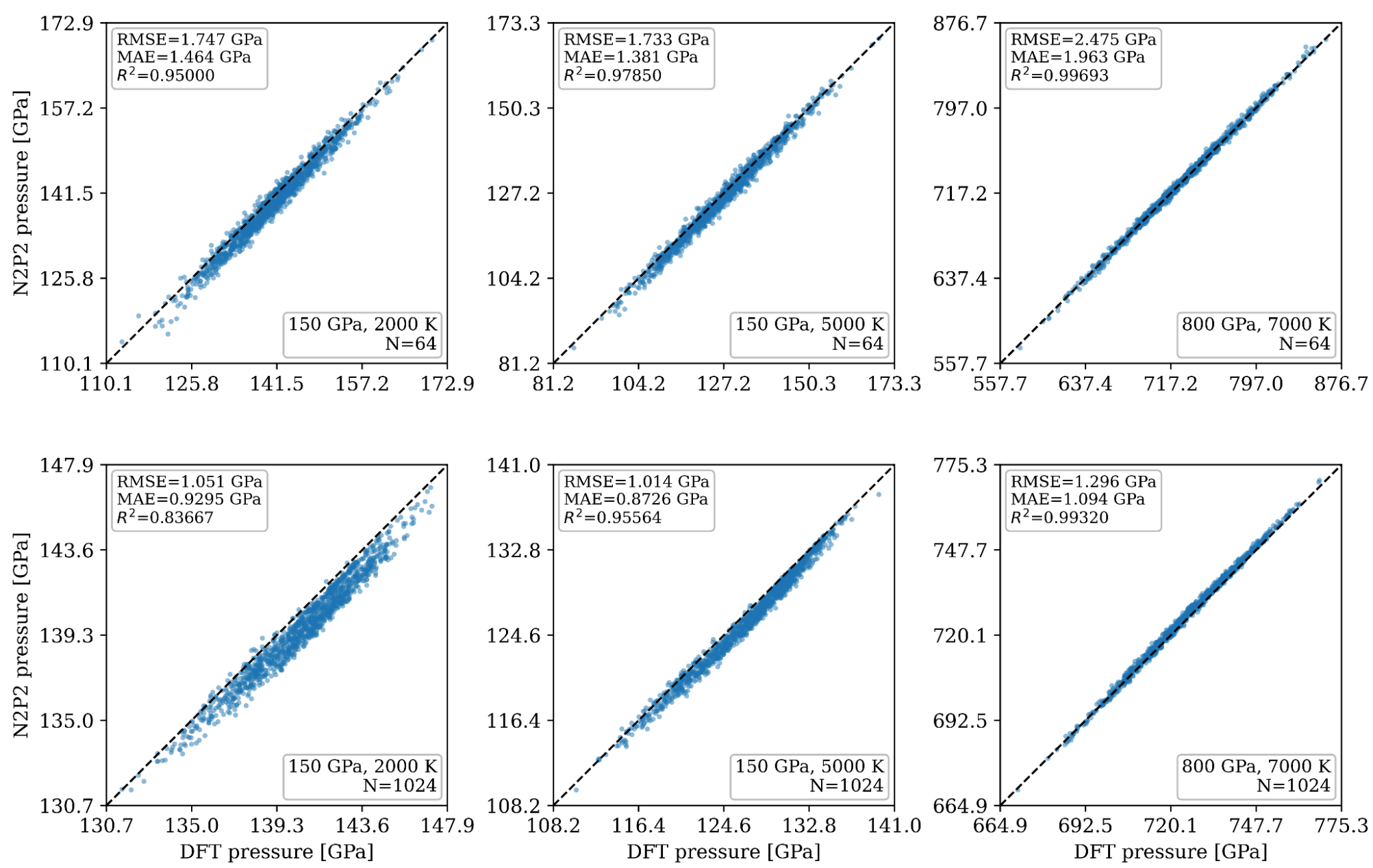

We also found that more advanced MLP architectures, such as MACE, can reduce the pressure discrepancy substantially. As shown in the figure below, the MACE MLP lowers the pressure error to about 1 GPa. Moreover, as discussed in Fig. 1 of the main text, the chemical potentials and mixing free energies computed with MACE remain consistent with those obtained from the N2P2 MLP. This further verified the thermodynamic results obtained from the N2P2 MLP models.

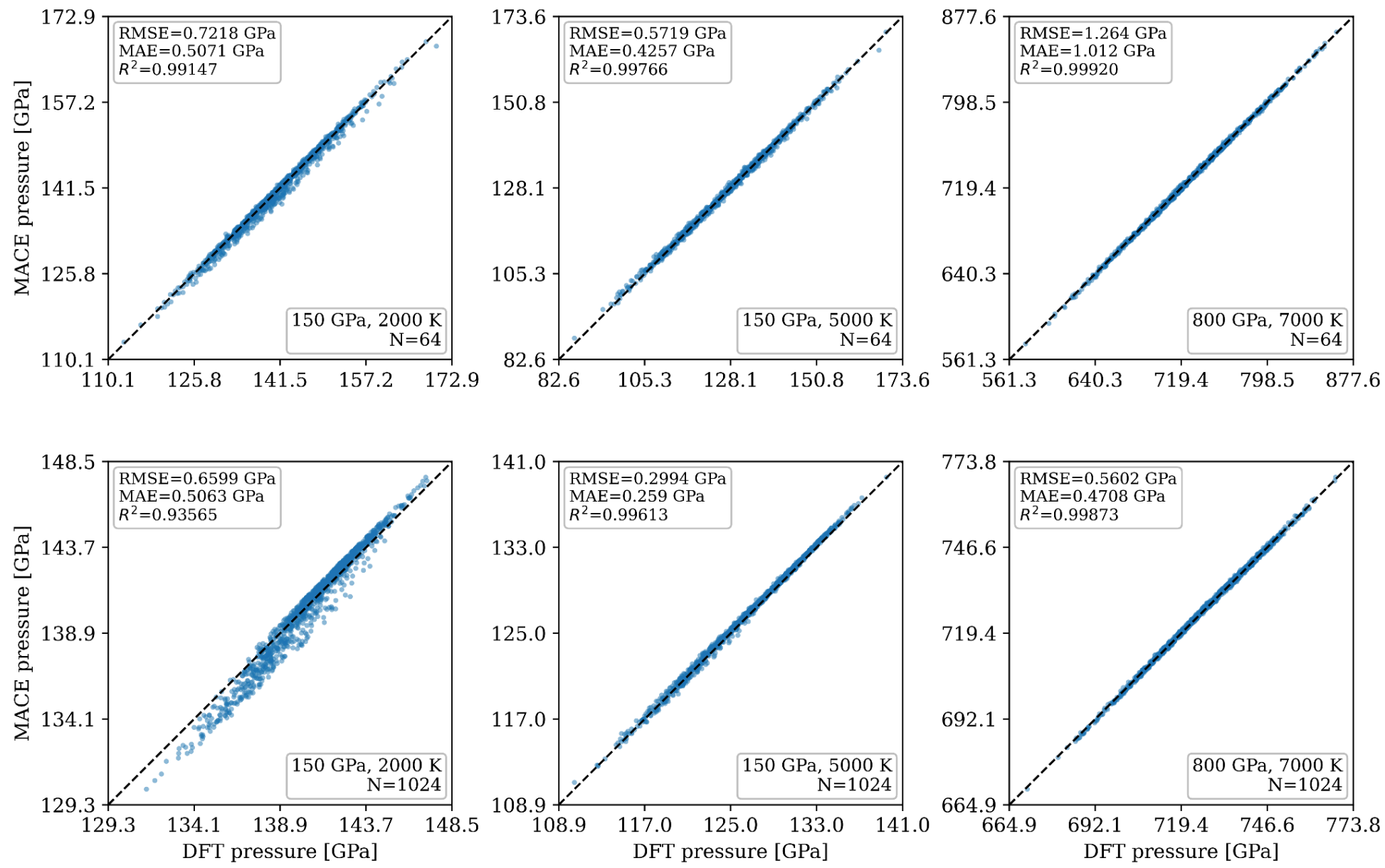

I find the discussion of how the machine learning potential was trained and tested (see supplementary material) inadequate. How do we relate the RMS errors of the energy (is that per atom?) and force, to the errors in the phase diagram? I understand that these measures are standard in the field, however what should be important is whether a given model is biased toward pure hydrogen, helium, or a mixture and whether there is any pressure dependance in the bias.

Authors:
The referee asked how the reported energy and force errors of the MLP propagate into uncertainty in the phase diagram, and in particular, whether the MLP models are biased toward the mixture compositions or show any pressure-dependent bias. To address this, we have now:

1) clarified that the reported energy error is the per-atom RMSE;
2) performed additional benchmark of the MLP against DFT for 7000 snapshots covering at least 11 helium compositions at three P-T conditions;
3) quantified the effect of the MLP error on the predicted miscibility boundary by promoting the mixing free energy ΔG^{mix} to the DFT level, using the free energy perturbation (FEP) method based on the MLP free energies.

As a result, we confirm that the MLP accurately reproduces the DFT energies without a composition-dependent bias, and that the MLP-derived phase boundaries remain consistent with the DFT reference within an uncertainty of about 1000 K. We elaborate on these details in the following:

1) First, we have corrected Table S1 in the SI to make clear that the RMS energy errors are reported in units of per-atom energy:

TABLE I. Training and testing errors (RMSE) for different MLP architectures based on the vdW-DF, PBE, and HSE functionals.

Functional	ML package	Training Error (RMSE)		Testing Error (RMSE)	
		Energy [meV/atom]	Force [meV/Å]	Energy [meV/atom]	Force [meV/Å]
vdW-DF	N2P2	32.49	445.12	33.38	454.58
	MACE	13.10	187.30	12.50	192.00
	CACE	8.30	273.88	7.25	272.75
	Δ(PBE→vdW-DF)	1.16	39.77	1.18	40.54

2) To demonstrate that the MLP accurately reproduces DFT energies without bias toward specific mixture compositions or pressure regimes, we further evaluated both vdW-DF DFT and N2P2 vdW-DF MLP energies on a dataset of 7000 snapshots. For each system size (N=64 and N=1024), these configurations cover 12 helium compositions (x_{He} = 0.016, 0.047, 0.11, 0.20, 0.30, 0.39, 0.55, 0.61, 0.73, 0.80, 0.92, and 0.98) for 2000 K and 5000 K at 150 GPa, and 11 compositions (x_{He} = 0.016-0.92) at 800 GPa and 7000 K.

We further verified that there is no systematic bias in the accuracy of the MLP related to helium composition or pressure by analyzing the composition-dependent energy differences between the DFT and N2P2 MLP calculations at different pressures, as shown below:

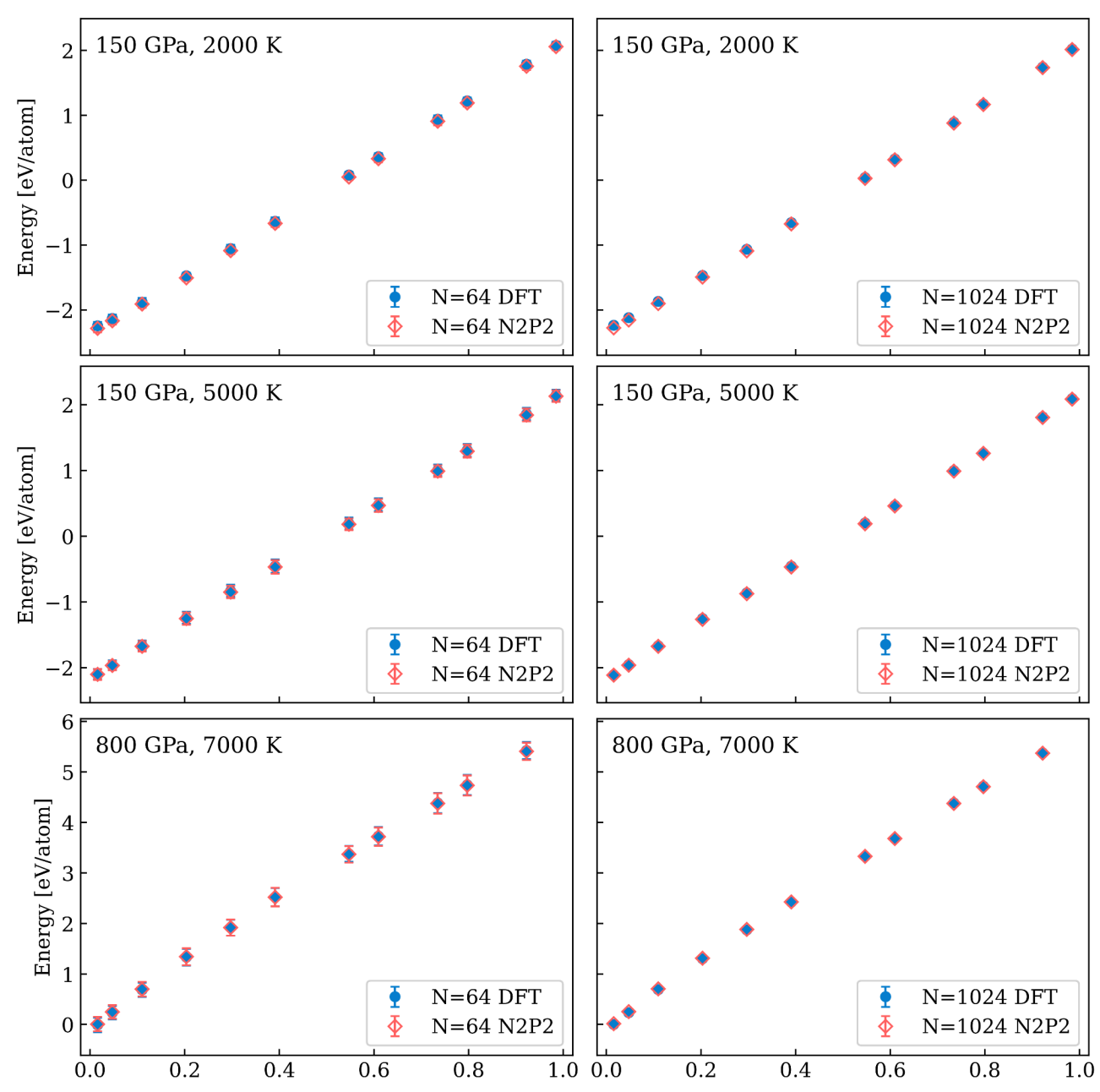

Figure: Per-atom energies derived from vdW-DF DFT and N2P2 MLP as a function of helium fraction, x_{He}, for H/He mixtures at three thermodynamic conditions: 150 GPa and 2000 K, 150 GPa and 5000 K, and 800 GPa and 7000 K. The left and right columns show results for N=64 and N=1024, respectively. For each system size, DFT energies are shown in blue and MLP energies in red. Error bars indicate the standard error of the mean over the 100 sampled configurations.

Moreover, the close agreement between MLP and DFT energies provides the basis for applying an FEP correction from the MLP to the DFT level.

3) We agreed with the referee that standard fitting metrics alone are not sufficient to judge the reliability of the phase diagram. To quantify the **effect of the MLP error on the computed immiscibility temperatures**, we further corrected the MLP-derived Gibbs free energy of mixing ΔG^{mix} to the DFT level using free-energy perturbation (FEP) for the large system with N=1024 based on vdW-DF.

   In detail, for each P-T-x_{He} condition with N=1024, we used 100 configurations and computed the energy difference $\Delta U = U_{DFT} - U_{MLP}$. More specifically, U_{DFT} is the DFT energy for a configuration, and U_{MLP} is the corresponding MLP energy evaluated on the same configuration. The free-energy difference for the system at the DFT and the MLP level Δg can then be estimated as:

$$N\Delta g = -k_{\mathrm{B}}T\ln\left\langle \exp\left(-\frac{\Delta U}{k_{\mathrm{B}}T}\right)\right\rangle_{\mathrm{MLP}}$$

   Using such free energy correction terms, we can compute the mixing Gibbs free energies ($\Delta G^{mix}(x_{He}) = x_{He}\,\mu_{He} + (1-x_{He})\,\mu_H + \Delta g(x_{He})$) for systems at fixed P, T. As shown in the following plot, the MLP mixing free energies show overall good agreement with the DFT values at all three conditions (150 GPa, 2000 K; 150 GPa, 5000 K; 800 GPa, 7000 K) that cover atomic and molecular H, mixing and demixing conditions. The largest absolute correction to ΔG^{mix} is observed at the low temperature condition (150 GPa, 2000 K), and the biggest error (at x_{He}=0.54) is still within 10 meV/atom. At the two higher temperatures that are more relevant for the immiscibility boundary, the MLP mixing free energies are essentially on top of DFT values. Even if we take the most pessimistic view and assume that the ΔG^{mix} is on the order of 10 meV/atom, this discrepancy would propagate to the second-order derivative of ΔG^{mix} with respect to x_{He}, $(\partial^2 G/\partial x^2)_{T,P}$, and can potentially shift the helium composition of the demixed phases by less than 0.05. Such a shift may be even smaller when statistical uncertainties are taken into account (see error bars in the figure below). Consequently, the DFT-level correction translates to less than 1000 K adjustment in the derived phase boundaries, based on our phase diagrams (Fig. 2(d-f)) reported in the main text. Corrections of this magnitude are not expected to significantly shift the predicted demixing boundary.

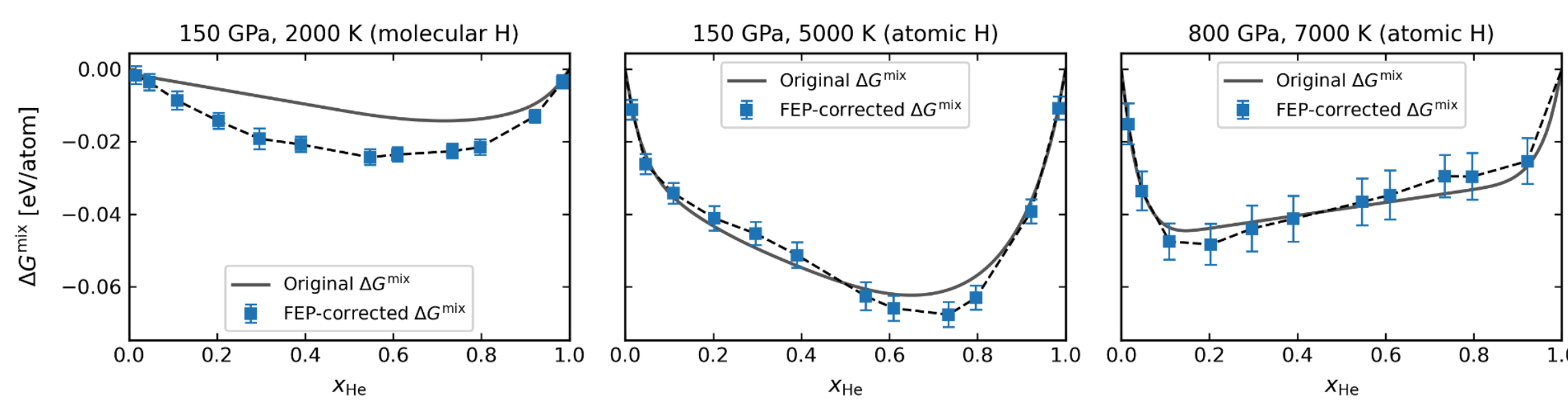

   Figure: Per-atom Gibbs free energy of mixing, ΔG^{mix}, as a function of x_{He} for H/He mixtures for three thermodynamic conditions based on vdW-DF: 150 GPa and 2000 K, 150 GPa and 5000 K, and 800 GPa and 7000 K. Gray solid lines show the original MLP results for the N=3456 system, while blue squares denote the FEP-corrected ΔG^{mix}.

I am also concerned that the "configurations spanned a broad range of PT conditions from 1-1200GPa.." Is this a good idea? Would't it be more accurate to have the training set be in the region of density relevant to the planetary adiabat, say 400GPa-600GPa and 3000K to 6000K. Was the composition of the

training set controlled to represent the 3 phases (pure H, pure He and mixed) equally to avoid biasing the results? The training sets should be made available, otherwise the models and the results are not reproducible.

Authors:
The referee asked whether it is better to train the MLP on a broad P-T range rather than focusing only on the region most relevant to the planetary conditions, whether the training set could be biased by uneven representation of pure and mixed phases, and whether the training data are publicly available for reproducibility. To address these points, we have now:

1) discussed recent developments in MLP research showing that training on broader, more diverse datasets improves robustness and interpolation within specific target domains;
2) trained a specific model only for 400-600 GPa and 3000-6000 K, and compared its accuracy with that of the model trained on the full dataset;
3) clarified the public availability of the training set.

Our main conclusion is that training on the broader dataset does not reduce accuracy in the target planetary regime.

Indeed, the latest insight from the field of MLP research is that it is better to train on a big dataset including diverse systems and conditions. For example, the UMA model (https://arxiv.org/abs/2506.23971) is trained on hundreds of millions of molecular, material, and interfacial configurations, and can be used to model a wide range of systems with elements across the periodic table. We have also trained a MACELES-OFF model (https://pubs.acs.org/doi/abs/10.1021/acs.jctc.5c01400) on ~1M small molecular systems, and the MLP is generally applicable for organic systems.

The H/He MLP is not at the same scale as the “foundation” models mentioned above, but nevertheless benefits from training simultaneously on configurations from all thermodynamic conditions and compositions together. To show this, we also trained an MACE model only on configurations from 400 GPa-600 GPa and 3000 K to 6000 K, and compared the test errors of the model on such configurations to the original model trained on all configurations. The table below shows that training on the whole set improves the MLP’s accuracy on the configurations within a P-T region.

Table: RMSE errors on configurations from 400GPa-600GPa and 3000K to 6000K.

models	RMSE E / meV / atom	RMSE F / meV / Å	RMSE Stress (Virials) / meV / Å^3
MACE (whole set)	7.8	214.4	17.1
MACE (400GPa-600GPa and 3000K to 6000K)	8.0	233.0	22.7

Indeed, the training set is fully publicly available in the SI repository:
https://github.com/ChengUCB/HighPressure_HHe
In the folder https://github.com/ChengUCB/HighPressure_HHe/tree/main/DFT_calculations

Reviewer #3 (Remarks on code availability):

The training set needs to be made available.

We have uploaded all data necessary to reproduce our results, including the training set, the trained MLP model, and the input files used for training, to the following GitHub repository: https://github.com/ChengUCB/HighPressure_HHe.

Reviewer #4 (Remarks to the Author):

The paper predicts the conditions of H-He immiscibility as they apply to our understanding of the evolution of giant planet interiors. The authors find that immiscibility and Helium rainout are likely to be occurring in Saturn today, whereas He rainout may not be occurring in Jupiter. These are important results that will serve as a baseline in future efforts to understand the thermal evolution of these bodies, their recently discovered compositional inhomogeneity, and the generation of their magnetic fields.

There have been many previous predictions of H-He immiscibility based on ab initio molecular dynamics simulations and the present results place the critical temperature at the low temperature end of these previous efforts. It should be emphasized that predicting immiscibility in this system is computationally challenging and previous efforts may have been biased by small system sizes or limitations in the methodology, as the present authors discuss in some detail. As compared with previous work, I appreciated in this paper the use of relatively large systems, the application of the S0 method, which allows for very dense sampling of P-T-x space, the careful contstruction of MLP and comparison of multiple XC functionals.

Authors:
We thank the referee for examining our work and for the appreciation of our methodology. We have extensively revised the manuscript and addressed the comments.

I recommend publication after the following comments are addressed.

1) I would appreciate further discussion of the MLP construction, particularly addressing the following points: a) Errors in energy and force are probably acceptable, but not the smallest that have been published. For example, Deng et al. (PRB, 2023) in a three-component system over a comparable PT range produce energy and force RMSE at the low end of what is reported here while also fitting the viral very well. b) Do the authors attempt to fit the viral tensor? If not why? If so, what are the results? c) fitting to energy/force/(viral) involves meta-parameters that provide relative weights to these misfits. How did the authors choose these weights?

Authors:
The referee asked for further verification of the MLP construction: whether the reported errors are sufficiently accurate compared with prior work, whether we attempted to fit the virial tensor, and how the relative weights of the energy, force, and virial loss terms were chosen. To address this, we have now:

1) compared our MLP errors with those in the literature in the context of the different thermodynamic ranges and system complexity,
2) examined explicitly whether including stress/virial data in the training improves the model
3) clarified how the fitting weights were selected in practice.

We confirmed that our MLPs are appropriate for the much broader and more challenging H/He dataset considered here, and including virial in the training does not substantially improve the fitted model accuracy or thermodynamic predictions. We discuss these points in detail below:

a) We suppose that the referee is referring to Deng, Jie, et al. "Melting of $MgSiO_3$ determined by machine learning potentials." *Physical Review B* 107.6 (2023): 064103. https://journals.aps.org/prb/abstract/10.1103/PhysRevB.107.064103 where the authors fitted a MLP using DeepMD for configurations of $MgSiO_3$ at 2000 to 8000 K and pressures from ~0 to 220 GPa. Their MLP RMSE errors for the energies, atomic forces, and stresses are 4.9 meV/atom, 0.24 eV/Å, and 0.37 GPa, respectively. Note that in about half of such a thermodynamic condition window, $MgSiO_3$ is a crystal.

   In comparison, our H/He training set spans the temperatures 500-16000 K and pressure 1-1200 GPa, so the energy variances here are larger compared to Deng et. al. Moreover, liquids are harder to describe compared to crystals, as the latter typically contain very similar atomic environments.

   TABLE I. Training and testing errors (RMSE) for different MLP architectures based on the vdW-DF, PBE, and HSE functionals.

Functional	ML package	Training Error (RMSE)		Testing Error (RMSE)	
		Energy [meV/atom]	Force [meV/Å]	Energy [meV/atom]	Force [meV/Å]
vdW-DF	N2P2	32.49	445.12	33.38	454.58
	MACE	13.10	187.30	12.50	192.00
	CACE	8.30	273.88	7.25	272.75
	Δ(PBE→vdW-DF)	1.16	39.77	1.18	40.54

   Our MLP errors depend on the underlying architectures used. CACE and MACE have smaller errors compared to N2P2, although, as we extensively benchmarked and compared in the paper, all three MLPs produce consistent thermodynamic behaviors of H/He mixtures.

   MACE and CACE are considered to be amongst the most accurate architectures nowadays. N2P2 and DeepMD are considered to be cheap but have lower accuracy, as both omit many-body interactions beyond three-body terms. Here is one benchmark from https://www.nature.com/articles/s41524-024-01332-4 on the system of water, while there are many other benchmarks that reached similar conclusions.

   TABLE I. Root mean square errors (RMSE) for energy (E) per water molecule (in meV/H_2O) and force (in meV/Å) on a liquid water dataset from Ref. [37]. The cutoffs r_{cut} of the atomic environment and the numbers of message passing layers are listed.

	RuNNer [37] 6.35 Å	N2P2 6.35 Å	DeepMD [38] 6 Å	EANN [39] 6.2 Å	linear ACE [40] 5.5 Å	REANN [34] 6.2 Å, $T=3$	NequIP [17] 4.5 Å, $T=3$	MACE [18] 6 Å	CACE 5.5 Å, $T=0$	CACE 5.5 Å, $T=1$
E	7.0	5.8	6.3	6.3	5.196	2.4	2.8	1.9	3.49	1.77
F	120	108	92	129	99	53.2	45	36.2	79	47

b) We typically do not fit to virial/stress tensors when constructing MLPs for the following reasons: 1) The virials are completely determined by atomic forces. If the forces are used in the fit, in principle, fitting to virials does not provide additional information; 2) In DFT calculations, one often needs higher plane wave cutoffs to converge stress, compared to energy and forces, due to Pulay stress. In some DFT training sets, the stress tensors are not sufficiently converged; 3) We have observed that fitting to virial/stress tensors is not necessary and only yields very modest improvement in virial/stress predictions.

To expand on point 3, we have fitted an MACE and a CACE MLP using stress tensors. In the table below, we compare the stress error from these models to our original ones that were only fitted to energies and forces:

models	RMSE E / meV / atom	RMSE F / meV / Å	RMSE Stress (Virials) / meV / $Å^3$
MACE (E+F)	12.5	192.0	27.3
MACE (E+F+S)	12.9	195.6	24.7
CACE (E+F)	7.25	272.8	32.0
CACE (E+F+S)	7.97	305.3	32.7

From the comparison above, one can see that adding stress/virial to the training has an insignificant effect.

c) Indeed, fitting to energy/force/virials simultaneously involves selecting the weights of different loss terms. In practice, many MLIP packages have recommended defaults that usually yield near-optimal outcomes. For example, both MACE and CACE use two-stage training: the first stage has high force weight (1000) and low energy weight (1); the second stage has high energy weight (1000) and lower force weight (100). For N2P2, this is less established, but there are nonetheless typically used values. For N2P2, we tested a few combinations used on training accuracy and selected the optimal one.

2) S0 method. I would appreciate confirmation of the convergence of this method in k-space for this system with a plot similar to that of Fig. 2 of Chen (JCP, 2022).

Authors:
The referee asked for a convergence test to validate our S0 method for the high-pressure H/He mixtures. To address such a concern, we have performed a systematic size-scaling analysis using the vdW-DF N2P2 MLP.
As a result, we conclude that the mixing free energies and the resulting immiscibility boundary reported in the main text, which are computed based on N=3456 simulations, are fully converged with respect to system size. A detailed discussion follows:

We have performed a systematic size-scaling analysis using the vdW-DF N2P2 MLP. In particular, we calculated the radial distribution functions (RDFs) and structure factors (S(k)) for system sizes ranging from N=64 to N=3456 under four thermodynamic conditions:

1) 150 GPa and 2000 K, molecular H, phase separation at x_{He}=0.1;
2) 150 GPa and 3000 K, atomic H, fully mixed at x_{He}=0.05;
3) 150 GPa and 5000 K, atomic H, fully mixed at x_{He}=0.2;
4) 800 GPa and 7000 K, atomic H, phase separation at x_{He}=0.2;

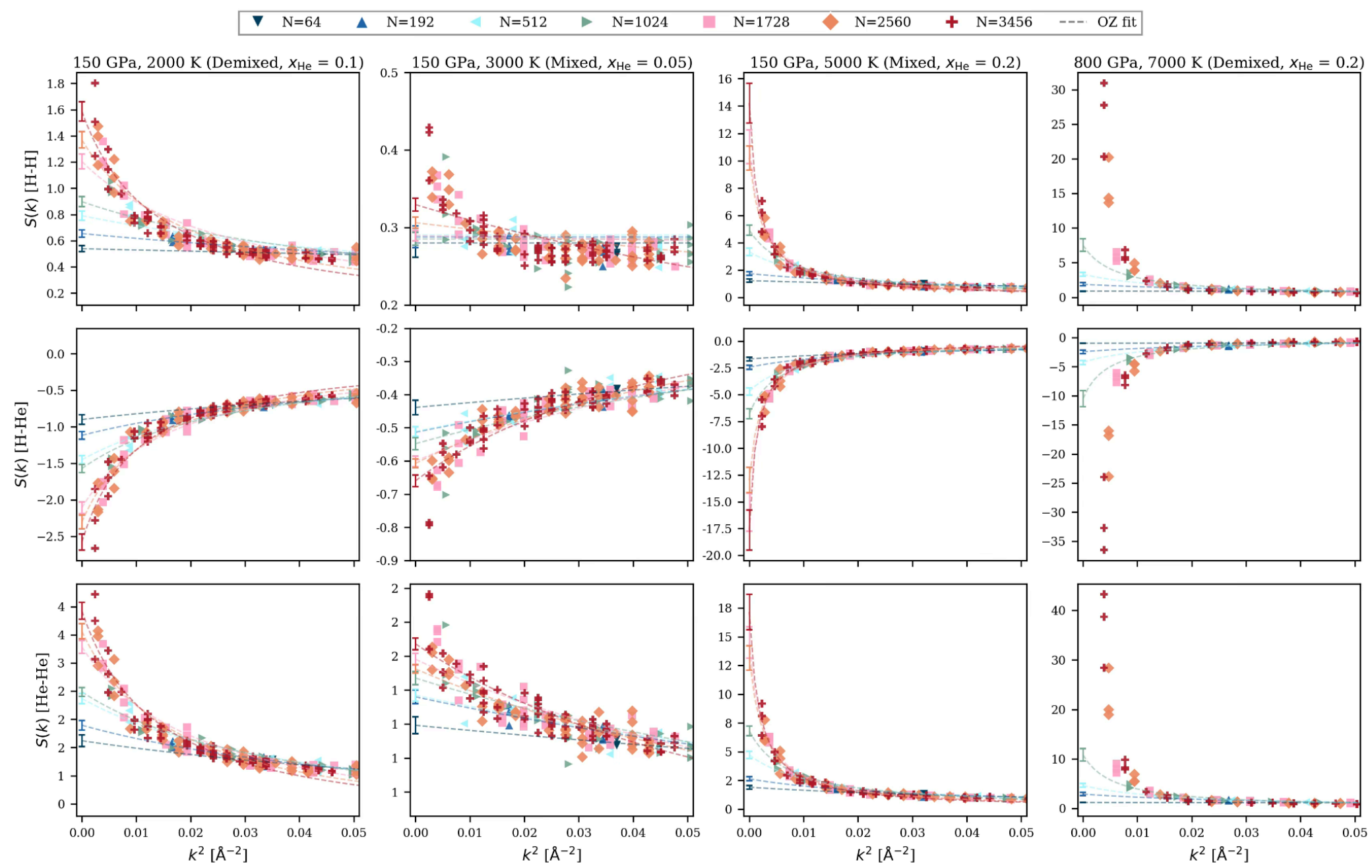

Figure: System-size dependence of the structure factors S(k) for H-H, H-He, and He-He pairs from N = 64 to N = 3456. Each column corresponds to a distinct thermodynamic condition. Dashed lines indicate Ornstein-Zernike (OZ) fits, while error bars at k^2 = 0 denote the extrapolated S0 limits.

The figure above demonstrates that the computed S(k) values and the corresponding S(0) fits are fully converged for a system size of N=3456. Furthermore, applying this S(0) method to our free-energy calculations confirms that the resulting mixing free energies are also fully converged at this system size.

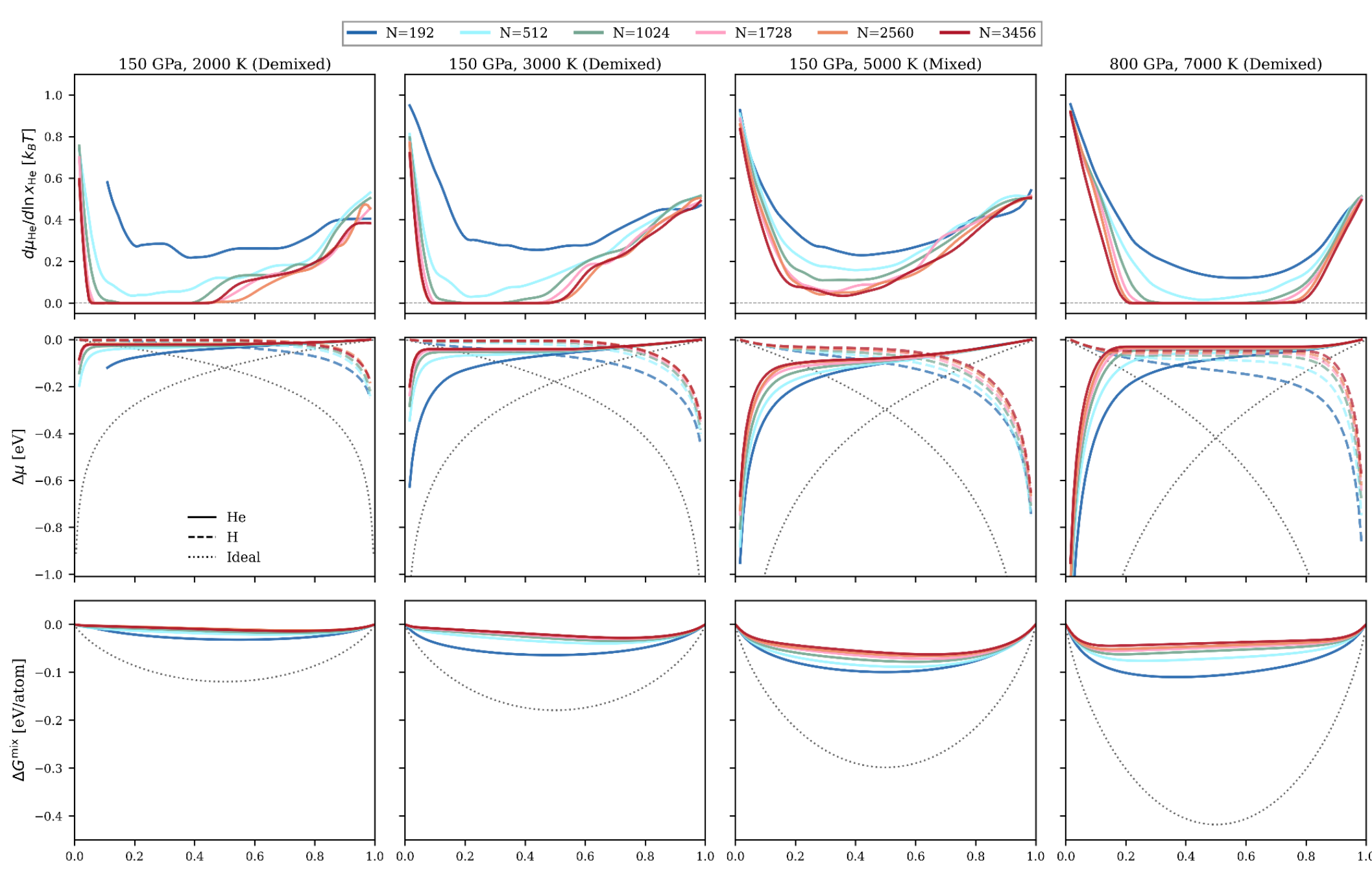

3) S0 method. The authors recognize the issue of two-phase separation as it occurs during simulations more completely than do many previous studies. The authors argue that in their method, immiscible states are manifested by d \mu / dn = 0. I would appreciate a demonstration or argument confirming this result starting from their Eq. 1. It is not immediately obvious to me from the right hand side of this equation that a phase separated system should yield zero.

Authors:
The referee asked for an explicit demonstration of why the condition dμ/dn = 0 marks the onset of phase separation in our manuscript. To address this, we now make explicit the thermodynamic connection between the dμ/dn = 0 and the curvature of the Gibbs free energy (G) with respect to composition. Our main point is that the condition of dμ/dn = 0 marks the point where the free-energy curvature vanishes. At this point, an infinitesimal composition fluctuation no longer costs free energy and the homogeneous state becomes unstable. Detailed discussion is provided below:

When evaluating the thermodynamic stability of a system at constant temperature (T) and pressure (P), we look at how the Gibbs free energy changes with respect to a single control variable, e.g., helium fraction (x) here. Considering a small fluctuation in x, i.e., x + δx, we can expand G in a Taylor series:

$$G(x+\delta x) = G(x) + (\partial G/\partial x)_{T,P}\,\delta x + 1/2\,(\partial^2 G/\partial x^2)_{T,P}\,(\delta x)^2 + O((\delta x)^3)$$

At equilibrium, $(\partial G/\partial x)_{T,P} = 0$ as the free energy is minimized at a fixed T-P condition. Therefore, the leading contribution to G(x+δx) from a small fluctuation is:

$$\Delta G = 1/2\,(\partial^2 G/\partial x^2)_{T,P}\,(\delta x)^2$$

The sign of ΔG is determined by the second derivative $(\partial^2 G/\partial x^2)_{T,P}$ as $(\delta x)^2>0$:

- If $(\partial^2 G/\partial x^2)_{T,P} > 0$, ΔG is locally convex (curving upward). An infinitesimal fluctuation further increases G (ΔG>0) and the system returns to equilibrium (minimum G).

- If $(\partial^2 G/\partial x^2)_{T,P} < 0$, ΔG is locally concave (curving downward). An infinitesimal fluctuation further lowers G (ΔG<0). Hence, it indicates that G(x) is not a free-energy minimum, and the assumption of thermodynamic equilibrium is broken. The system will spontaneously evolve, e.g., toward demixing.

Therefore, $(d\mu/dn)_{T,P} = 0$ or equivalently $(\partial^2 G/\partial x^2)_{T,P} = 0$ is the criterion for phase separation.

4) N-dependence. I may have missed this but the authors should discuss the dependence of their results on the system size.

Authors:
The referee asked about the dependence of our results on the system size. To address this, we now examine the size dependence of our results from three perspectives:

1) the finite-size effect in the DFT reference data;
2) the size dependence of the MLP predictions relative to DFT;
3) the convergence of the free-energy calculations with respect to the simulation cell size;

Our main conclusion is that the free energies and phase boundaries reported in the main text, which are based on simulations up to N = 3456, are fully converged with respect to system size. Detailed discussion follows:

1) To directly assess the electronic finite-size effect in our DFT reference data, we evaluated both vdW-DF DFT and N2P2 MLP energies on a dataset of 7000 snapshots at two system sizes, N=64 and N=1024. For each system size (N=64 and N=1024), these configurations cover 12 helium compositions (x_{He} = 0.016, 0.047, 0.11, 0.20, 0.30, 0.39, 0.55, 0.61, 0.73, 0.80, 0.92, and 0.98) for 2000 K and 5000 K at 150 GPa, and 11 compositions (x_{He} = 0.016-0.92) at 800 GPa and 7000 K. As a result, the comparison shows that the DFT energies obtained with N=64 and N=1024 are broadly consistent within the uncertainty across all investigated thermodynamic conditions.

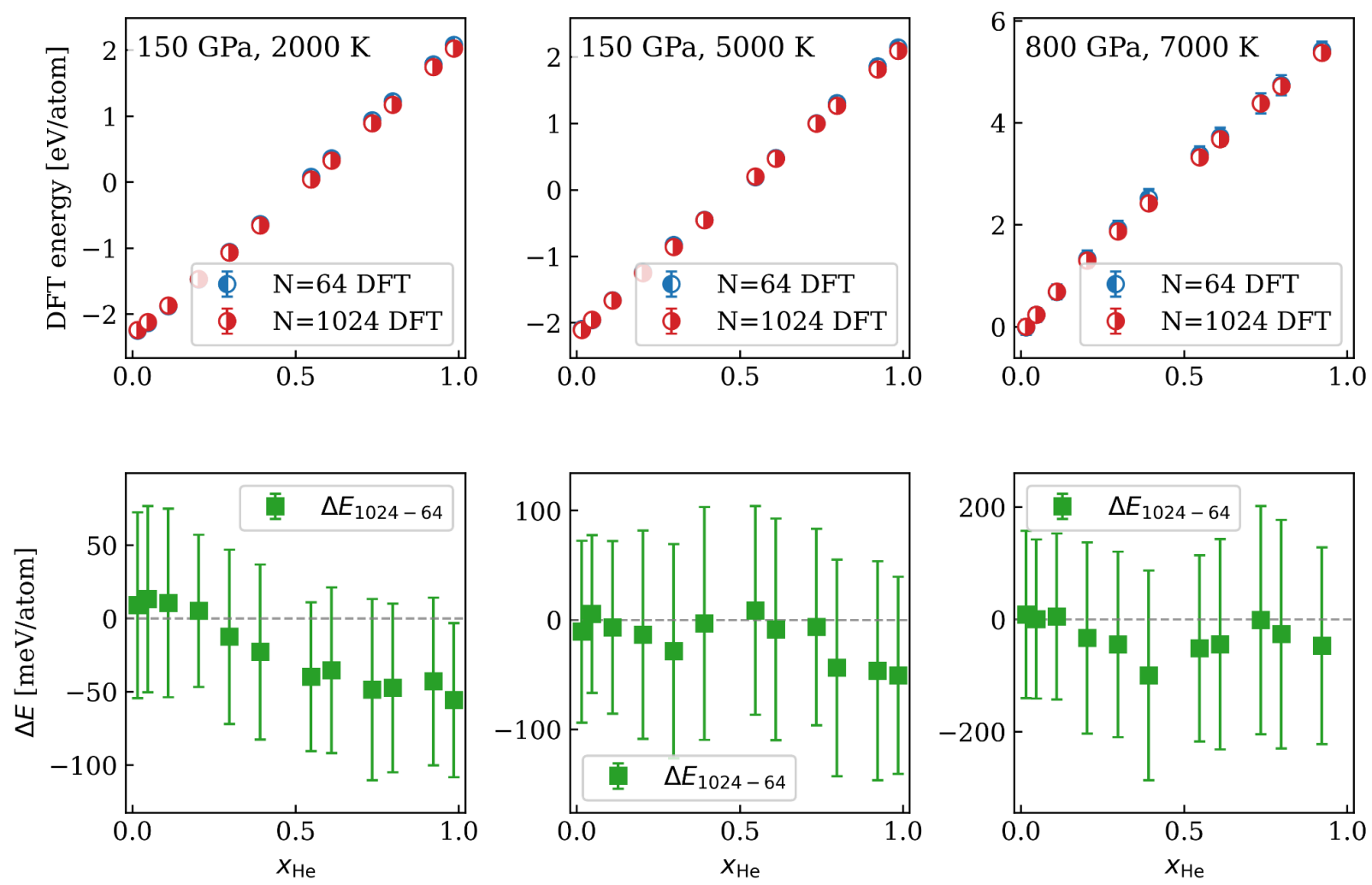

2) Building on the first point, we further verified that the N2P2 MLP accurately reproduces the vdW-DF DFT energies for both system sizes, N=64 and N=1024. This shows that the MLP does not introduce an additional size-dependent bias relative to the underlying DFT reference.

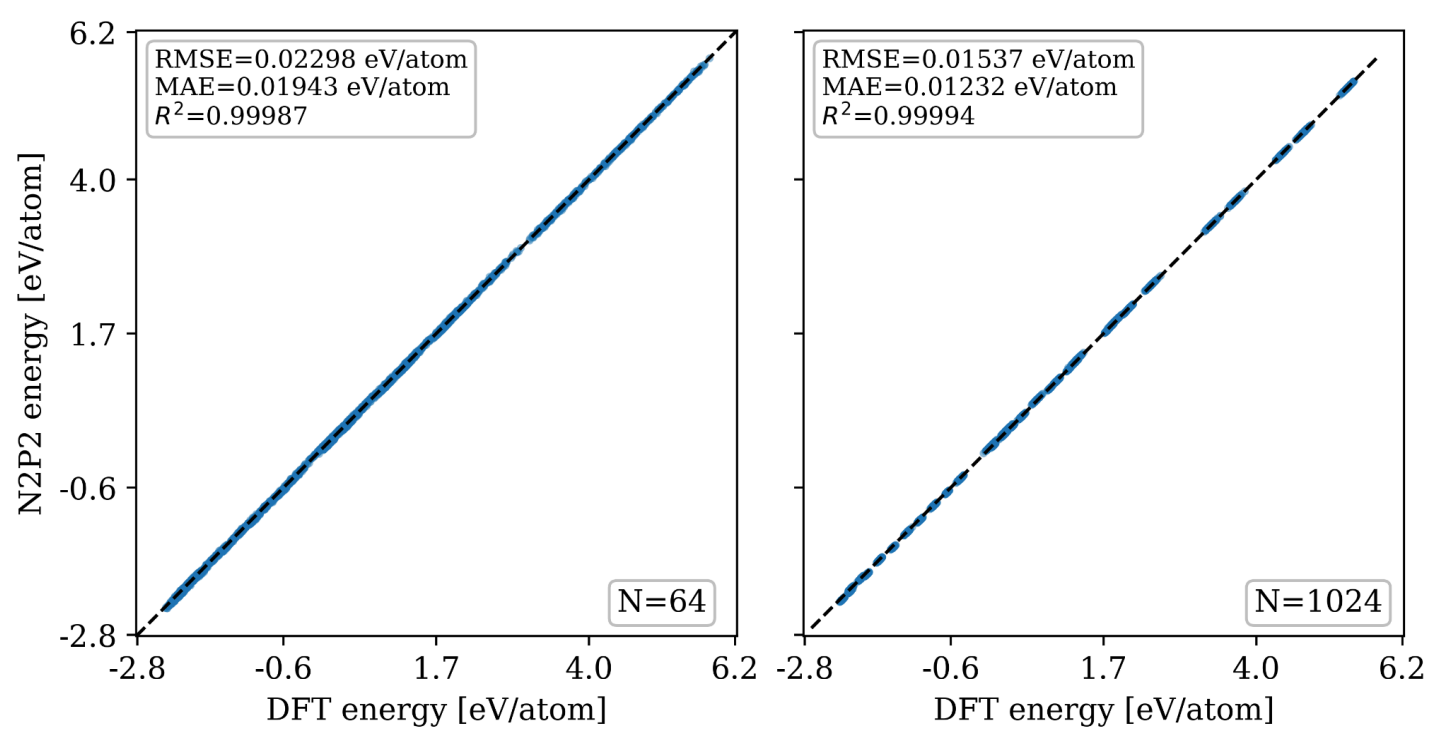

Figure: Parity comparison of per-atom energies predicted by the N2P2 MLP against vdW-DF DFT reference energies for H/He mixtures at 150 GPa and 2000 K, 150 GPa and 5000 K, and 800 GPa and 7000 K. The left and right panels show the aggregated results for N=64 and N=1024, respectively.

3) As the free-energy calculation is ultimately based on the S0 method, we carried out a systematic size-scaling analysis using the vdW-DF N2P2 MLP for system sizes ranging from N=64 to N=3456 under four representative P-T conditions:

a) 150 GPa and 2000 K, molecular H, H/He demixing;
b) 150 GPa and 3000 K, atomic H, H/He demixing;
c) 150 GPa and 5000 K, atomic H, fully mixed;
d) 800 GPa and 7000 K, atomic H, H/He demixing;

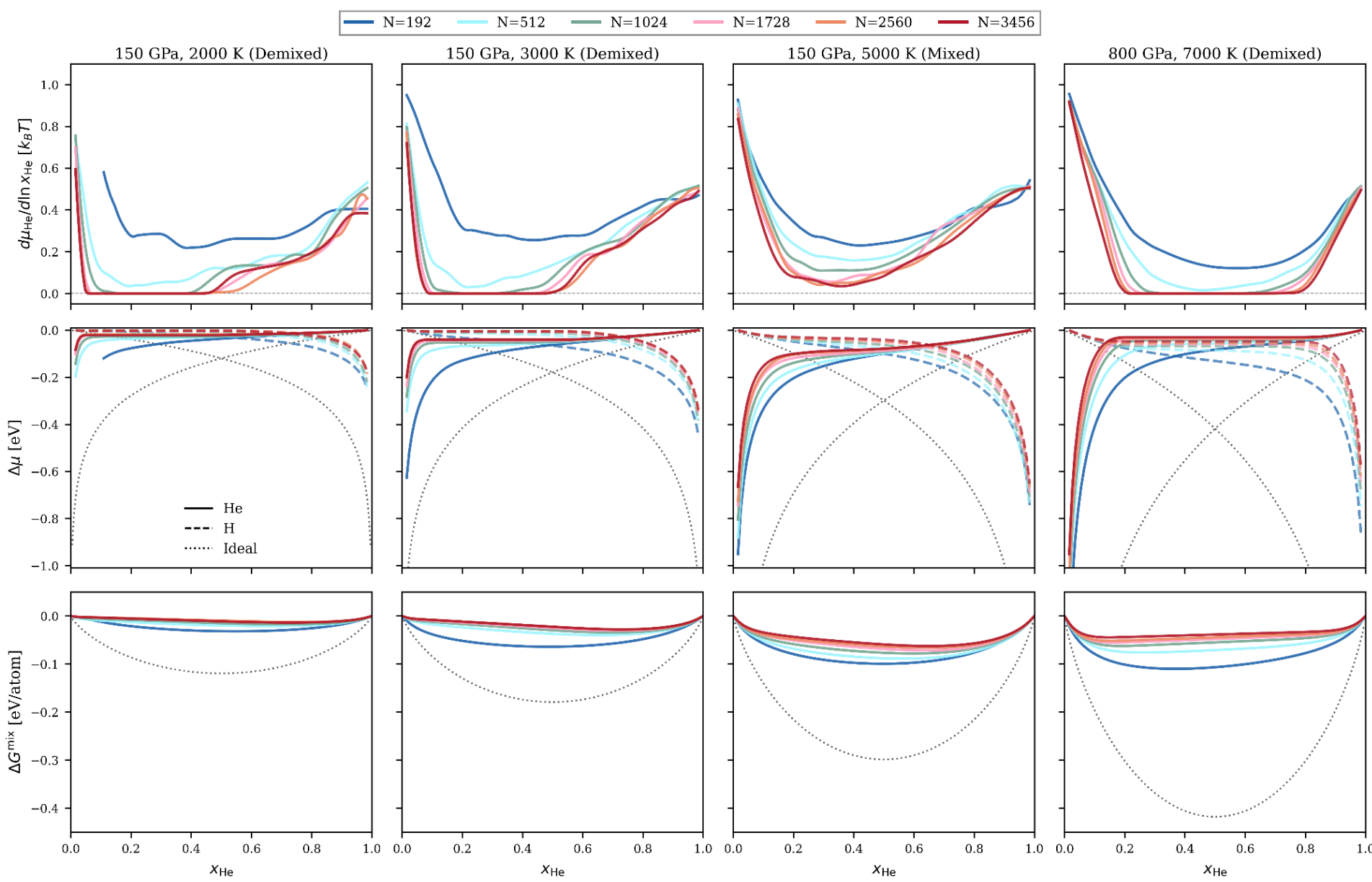

As shown in the figure above, we found that the mixing free energy requires larger system sizes (N >= 1024) to reach convergence.
As a result, we further confirm that the free-energy calculations and the resulting phase diagram reported in the main text, which are based on N=3456 simulations, are fully converged with respect to system size.

5) ENCUT. The authors use ENCUT=1200 eV, which is very large, but their PAW potential is also extremely hard. They must demonstrate convergence of their DFT results with respect to ENCUT.

Authors:
The referee asked whether the DFT results are fully converged with respect to the plane-wave cutoff, especially because the PAW potentials used here are very hard and the chosen ENCUT=1200 eV is unusually large. To address this, we performed a cutoff-convergence test over the range of 700-1300 eV and checked the convergence of energy, forces, and pressure. We conclude that an ENCUT of 1200 eV is sufficient to converge all quantities relevant to this study.

A detailed discussion follows:

1) **Energy calculations.** The results shown below indicate that the energy becomes essentially converged at a large cutoff of 1200 eV, since it changes by only 0.235 meV/atom (0.015%) from 1100 to 1200 eV and by only 0.099 meV/atom (0.007%) from 1200 to 1300 eV.

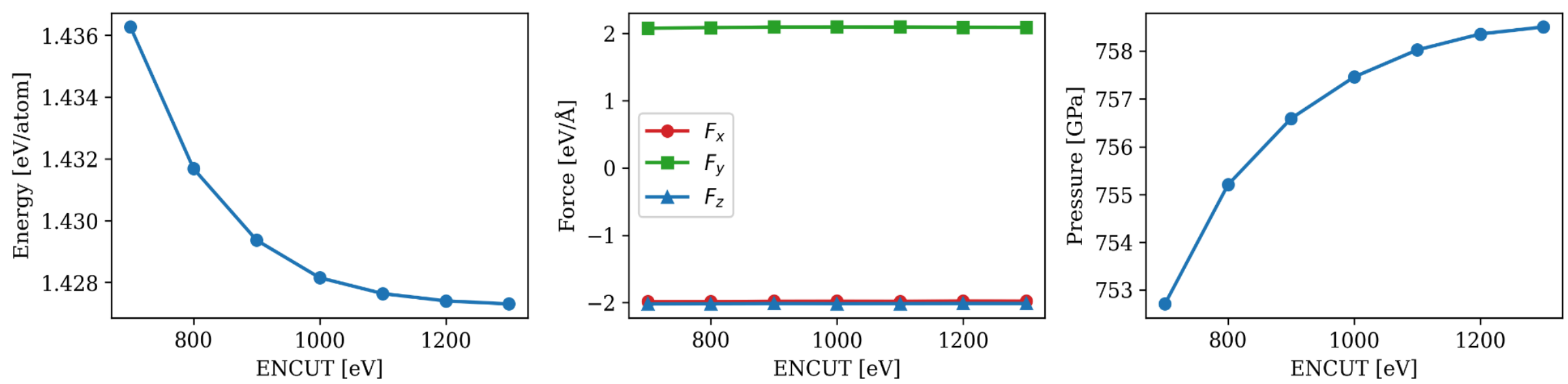

2) **Force calculations.** The atomic forces change marginally over the tested range: from 1100 to 1200 eV, the relative changes in the three force components are all below 0.13%, while from 1200 to 1300 eV they are at most 0.06%.
3) **Pressure calculations.** Although the pressure still shows a small monotonic increase with increasing cutoffs, its change is already reduced to 0.147 GPa (0.019%) between 1200 and 1300 eV, which does not affect the conclusions of this work.

Therefore, ENCUT = 1200 eV was adopted in all calculations to ensure reliable convergence. We have added the corresponding convergence-test results and discussion to the revised manuscript.

6) Fig. 2. Their critical curves for PBE and vdW-DF display prominent jogs near 600 GPa. The authors should comment on the possible origin of these features.

Authors:
The referee asked about the origin of the prominent jogs in the PBE and vdW-DF critical curves (black solid line) near 600 GPa in Fig. 2. To address this, we here discuss the finite resolution of our sampled P-T grid and the resulting uncertainty in the inferred immiscibility boundary. Our main conclusion is that these jogs do not indicate a physical anomaly, but instead arise from interpolation between discrete simulation points on the discrete P-T grid.

In detail, the immiscibility boundary was determined from simulations performed at 7 pressures (100, 150, 200, 400, 600, 800, 1000 GPa) and 12 temperatures from 1000 K to 12000 K in 1000 K increments. The subsequent linear interpolation between neighboring points can naturally produce apparent kinks or plateaus of a similar scale. For such a reason, these jogs reflect small variations in the H/He immiscibility boundary that cannot be fully resolved with the present grid spacing.

Taking the PBE results as an example, simulations at 600 GPa revealed H/He immiscibility at 8000 K, but not at 9000 K. Consequently, we determined the upper bound of the immiscibility boundary (black dashed line) at 600 GPa as 8000 K. As the same pattern was found at 800 GPa, we likewise assigned the upper bound there as 8000 K. Physically, the true immiscibility temperature at 800 GPa is likely slightly higher than at 600 GPa. Although the immiscibility temperature likely increases slightly from 600 GPa to 800 GPa, this subtle change is below our 1000 K resolution limit, which appears as a jog in the plotted curve.

7) Fig. 2. The authors should say something more about the serious disagreement between their results and the experimental results of Brygoo et al.

Authors:

The referee asked to further discuss the disagreement between our predicted H/He immiscibility boundary and the experimental result of Brygoo et al. (2021) at 150 GPa. To address this, we suggest that the difference may reflect the fact that the experiment probes a more complex nonequilibrium response than the equilibrium immiscibility boundary considered in our work.

Moreover, high-pressure experiments on H/He mixtures remain extremely challenging, yet only one experimental study based on laser-shock compression is currently available for H/He demixing in this regime. Given this limited experimental data, it is not easy to justify the origin of the discrepancy between theory and experiment.

While our calculations are targeted to determine the equilibrium thermodynamics of H/He demixing, the kinetic effects for H/He demixing are not constrained based on this work. However, under dynamic shock conditions in experiments, the observed mixing/demixing response may also depend on kinetic effects as well, including the timescales and length scale of hydrogen dissociation and species redistribution, all of which are closely coupled in this regime. A better understanding of these kinetic effects may help bridge the current gap between theoretical predictions and experimental observations.

8) RK. The authors do a sensible job of fitting their results to RK. These fits provide an additional opportunity to compare with previous results and I would appreciate the authors exploring this. T- and P-derivatives of Eq. 3 yield the entropy and volume of mixing (and the excess quantities). The former could be compared to the ideal mixing result, while the latter could be compared to widely used tabulated eos of H, He and H-He mixtures (Chabrier et al.), where volume mixing across the H-He join is known to be significantly non-ideal.

Authors:
We thank the referee for pointing out that the fitted R-K model provides an additional check to compare our results with previous work through the entropy and volume of mixing. To address this, we have now

1) used the temperature and pressure derivatives of the fitted R-K model to extract the excess entropy ΔS^{ex}_{RK} and excess volume of mixing V^{mix}_{RK}, respectively;
2) compared ΔS^{ex}_{RK} with the ideal-mixing model and V^{mix}_{RK} with the tabulated H/He equations of state mentioned by the referee.

These analyses further confirmed that the fitted R-K model reveals strongly non-ideal mixing in high-pressure H/He mixtures, particularly at lower temperatures. Moreover, the R-K-model-derived densities are consistent with tabulated EOS data, although with DFT-functional-dependent differences. Details are provided below:

1) **Excess entropy ΔS^{ex}_{RK}**

Within the framework of the R-K model, the mixing entropy can be taken from the temperature derivative as $\Delta S^{mix} = -(\partial \Delta G^{mix}/\partial T)_P = \Delta S^{id} + \Delta S^{ex}$. The excess entropy of mixing ΔS^{ex}_{RK} in the R-K model is then given by:

$$\Delta S^{ex}_{RK} = - x_{He} x_H \sum (\partial \omega_i/\partial T)(1 - 2x_{He})^{i-1}$$

The following figure presents the derived ΔS^{ex}_{RK} for x_{He}=0.09 as a function of temperature based on R-K models for three DFT functionals, with ideal entropy of mixing $\Delta S^{id} = -k_B (x_H \ln x_H + x_{He} \ln x_{He})$ (black dashed line) included for comparison:

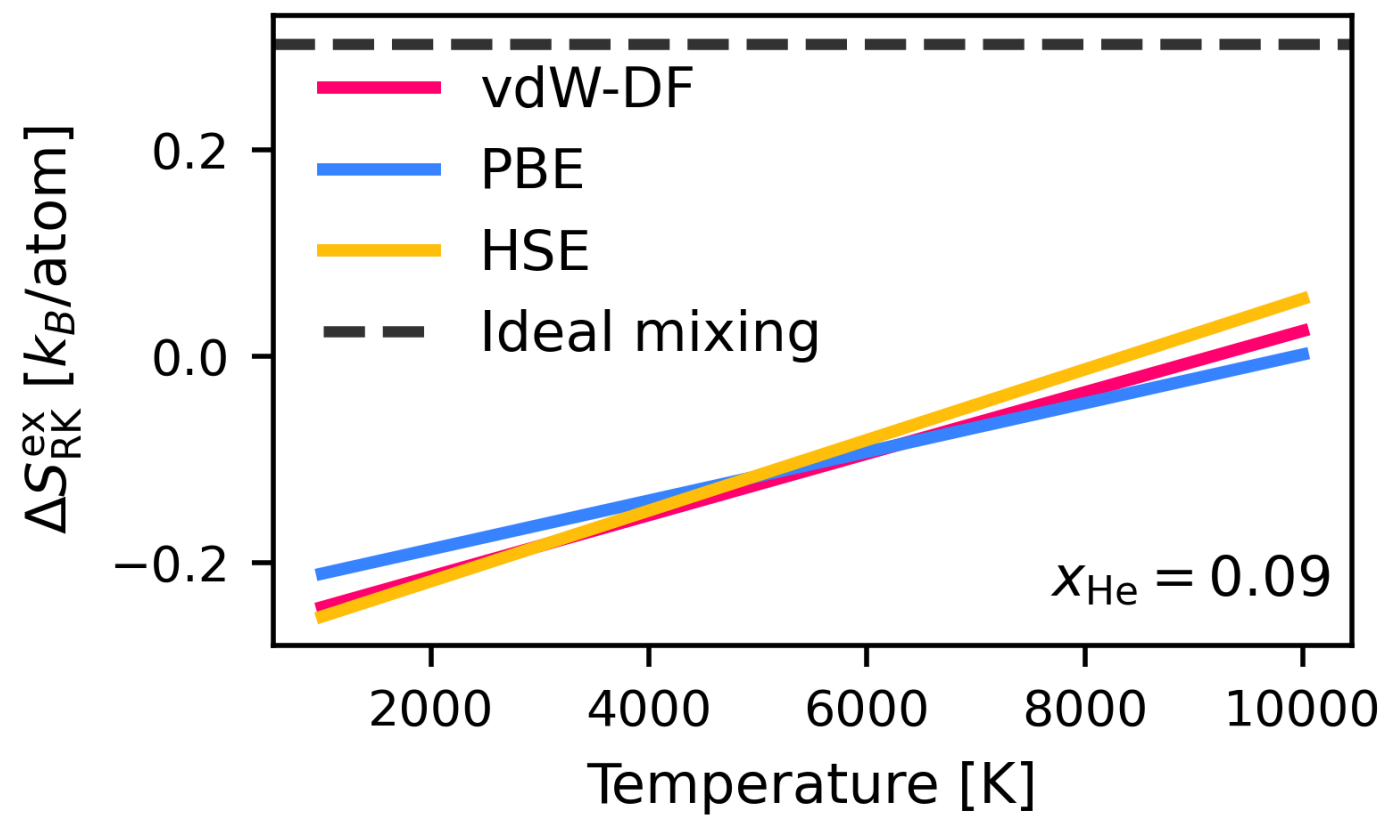

As a result, the ΔS^{ex}_{RK} values indicate a strong non-ideal mixing behavior in H/He mixtures, particularly at lower temperatures.

2) Excess volume of mixing V^{mix}_{RK}.

Likewise, the excess volume of mixing V^{ex} is obtained from the pressure derivative of ΔG^{mix} as $V^{ex} = -(\partial \Delta G^{mix}/\partial P)_T$, which takes the form:

$$V^{ex}_{RK} = -x_{He}x_H \sum(\partial \omega_i/\partial P)(1-2x_{He})^{i-1}$$

The total volume of the mixture is then expressed as the sum of the ideal components and the excess volume:

$$V^{mix}_{RK} = x_H V_{pure\ H} + x_{He} V_{pure\ He} + V^{ex}_{RK}$$

where $V_{pure\ H}$ and $V_{pure\ He}$ are obtained from MLP MD simulations of the pure phases. The resulting density as a function of pressure for $x_{He} = 0.09$ is shown below for the three DFT functionals:

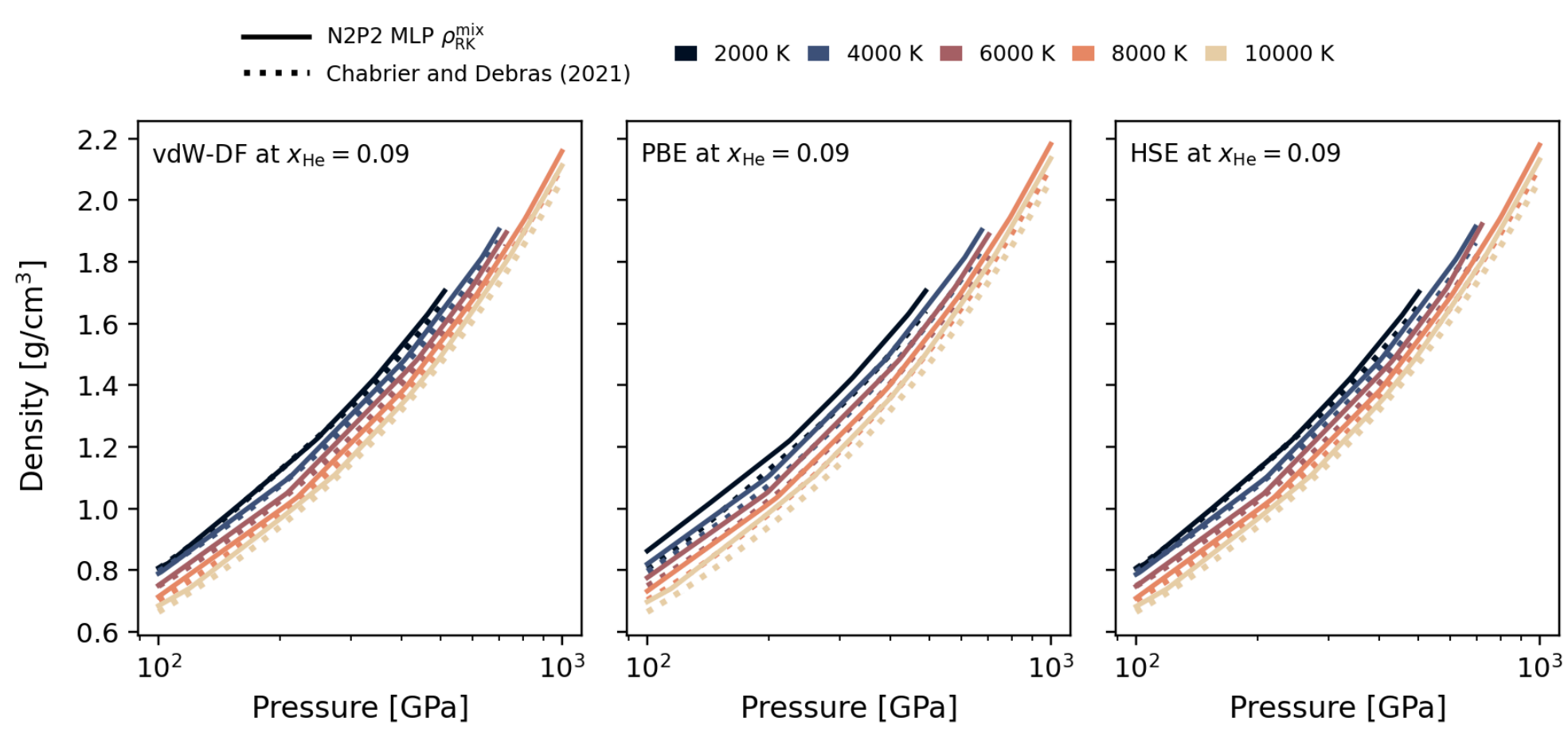

For comparison, we also include the widely used tabulated EOS values for H/He mixtures from Chabrier and Debras (2021, DOI: 10.3847/1538-4357/abfc48) with H/He interactions taken into account. Relative to these tabulated EOS data (black dotted line), the differences in our derived densities are up to 3.2% for vdW-DF, up to 7.2% for PBE, and up to 4.3% for HSE, respectively.

Overall, this comparison shows that the R-K analysis is physically consistent with the expected non-ideal mixing behavior of high-pressure H/He mixtures and provides a useful connection between our thermodynamic results and previous EoS models.

Reviewer #5 (Remarks to the Author):

I co-reviewed this manuscript with one of the reviewers who provided the listed reports. This is part of the Nature Communications initiative to facilitate training in peer review and to provide appropriate recognition for Early Career Researchers who co-review manuscripts.

This manuscript presents a first-principles study of hydrogen–helium immiscibility under planetary interior conditions. Using large-scale molecular dynamics driven by machine-learning potentials trained on the PBE, vdW-DF, and HSE functionals together with the S_0 chemical-potential method, the authors compute composition-dependent free energies and map the H/He immiscibility boundary over a wide pressure–temperature range. A central result is that the predicted demixing temperatures are ~2000 K lower than those from earlier ab initio studies using small simulation cells, which is attributed mainly to finite-size effects and enforced homogeneity. By comparison with planetary interior models, the authors conclude that helium rain is likely in Saturn but unlikely in Jupiter at protosolar helium abundance. The study is technically solid and has significant implications for planetary physics.

Authors:
We thank the reviewer for this positive and thoughtful assessment of our work. We appreciate the recognition of both the technical aspects of the study and its implications for planetary physics, particularly the importance of finite-size effects and large-scale simulations in determining the H/He immiscibility boundary.

Regarding this investigation, I have the following questions:
1. The MLPs are trained on configurations obtained from DFT cells and then applied in large-scale MLP-driven MD simulations. Could the authors comment on whether both the size of the training DFT cells and the system size used in the MLP MD simulations are sufficient to fully capture the H/He mixing free energy and immiscibility boundary—particularly near the molecular–atomic hydrogen transition—and how sensitive the results are to the chosen system size?

Authors:
The referee asked about the dependence of our results on the system size. To address this, we now examine this (particularly near the atomic-molecular transition of hydrogen) from three perspectives:

1) the finite-size effect in the DFT reference data;
2) the size dependence of the MLP predictions relative to DFT;
3) the convergence of the free-energy calculations with respect to the simulation cell size;

Our main conclusion is that the free energies and phase boundaries reported in the main text, which are based on simulations up to N=3456, are fully converged with respect to system size.
Detailed discussion follows:

1) To directly assess the electronic finite-size effect in our DFT reference data, we evaluated both vdW-DF DFT and N2P2 MLP energies on a dataset of 7000 snapshots at two system sizes, N=64 and N=1024. For each system size (N=64 and N=1024), these configurations cover 12 helium compositions (x_{He} = 0.016, 0.047, 0.11, 0.20, 0.30, 0.39, 0.55, 0.61, 0.73, 0.80, 0.92, and 0.98) for 2000 K and 5000 K at 150 GPa, and 11 compositions (x_{He} = 0.016-0.92) at 800 GPa and 7000 K. The condition of 150 GPa and 2000 K represents H/He mixtures in the molecular-hydrogen regime, whereas the latter two correspond to mixtures dominated by atomic hydrogen. We then

compared the averaged per-atom DFT energies obtained from the two cell sizes, and the results are shown in the following figure:

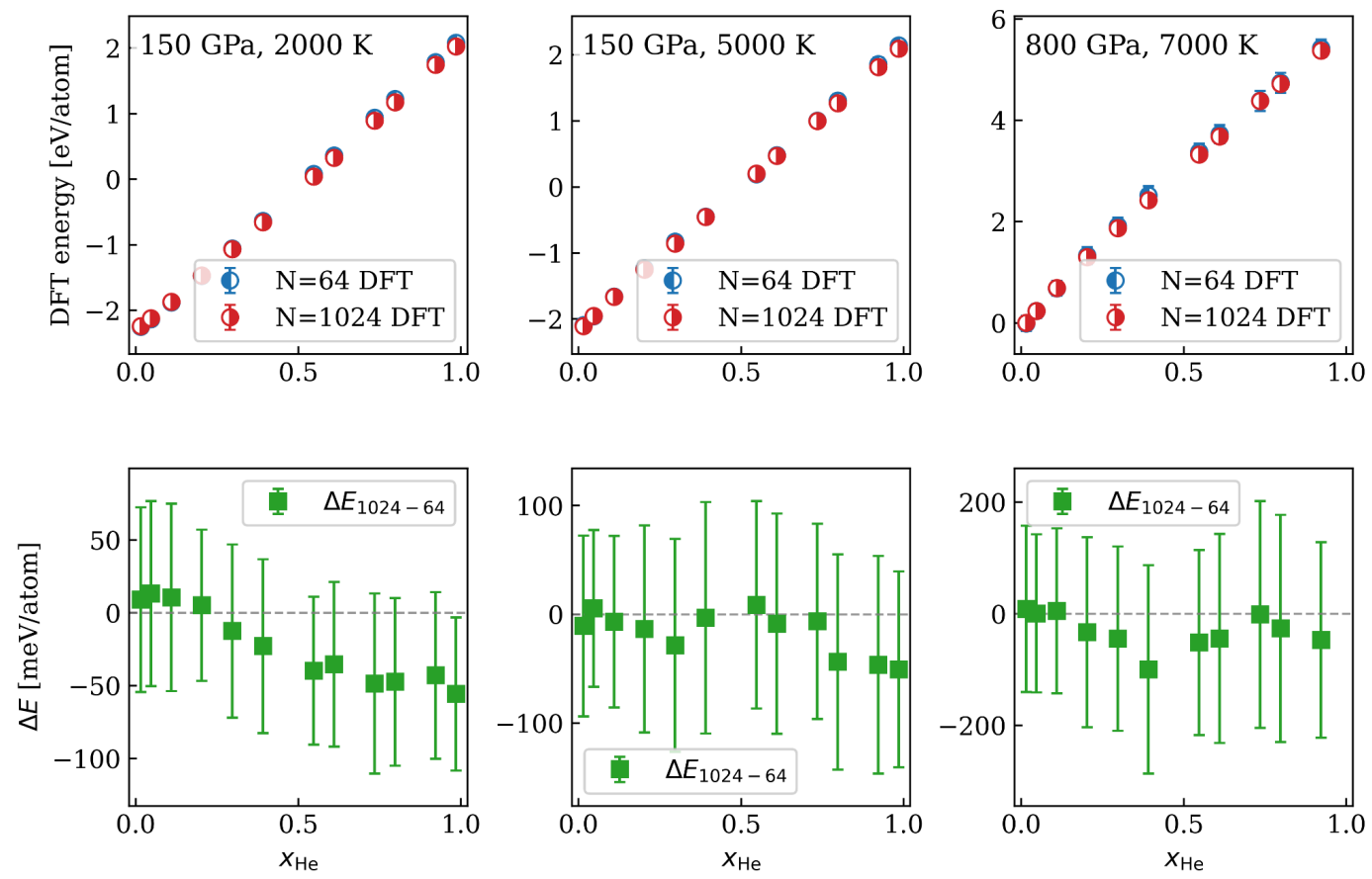

As a result, the comparison shows that the DFT energies obtained with N=64 and N=1024 are broadly consistent within the uncertainty across all investigated thermodynamic conditions. Moreover, **the size-dependent DFT energy differences show no clear systematic correlation with either the atomic-molecular phase transition or the demixing behavior.**

2) **System-size effects on MLP energy predictions.** Building on the first point, we further verified that the N2P2 MLP accurately reproduces the vdW-DF DFT energies for both system sizes, N=64 and N=1024. This shows that the MLP does not introduce an additional size-dependent bias relative to the underlying DFT reference:

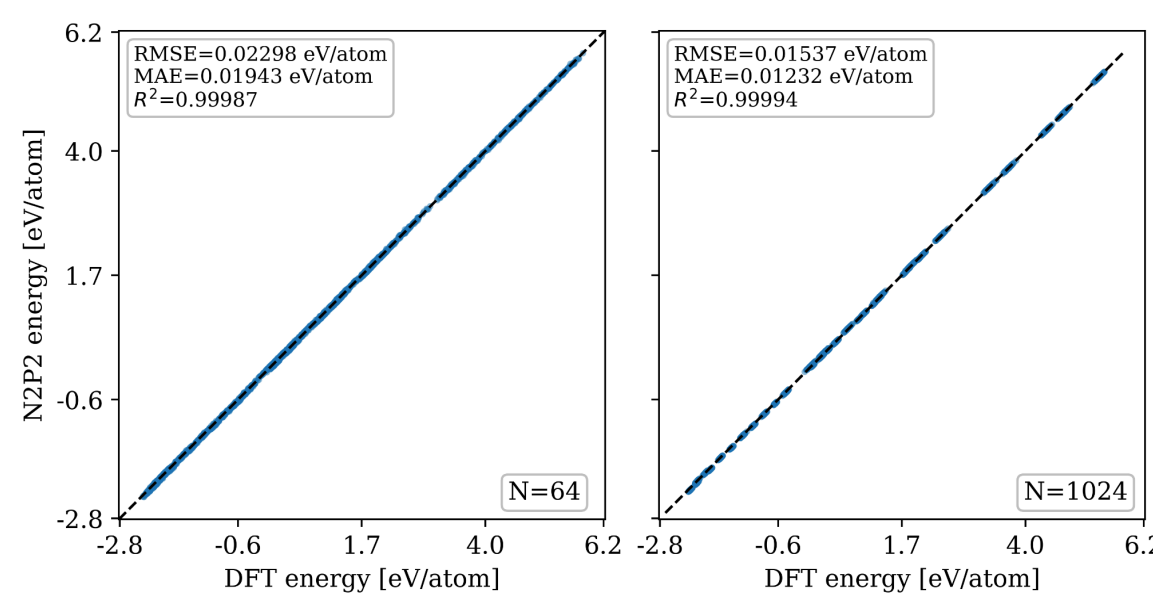

3) As the free-energy calculation is ultimately based on the S0 method, we carried out a systematic size-scaling analysis using the vdW-DF N2P2 MLP for system sizes ranging from N=64 to N=3456 under four representative P-T conditions:
   a) 150 GPa and 2000 K, molecular H, H/He demixing;
   b) 150 GPa and 3000 K, atomic H, H/He demixing;
   c) 150 GPa and 5000 K, atomic H, fully mixed;
   d) 800 GPa and 7000 K, atomic H, H/He demixing;

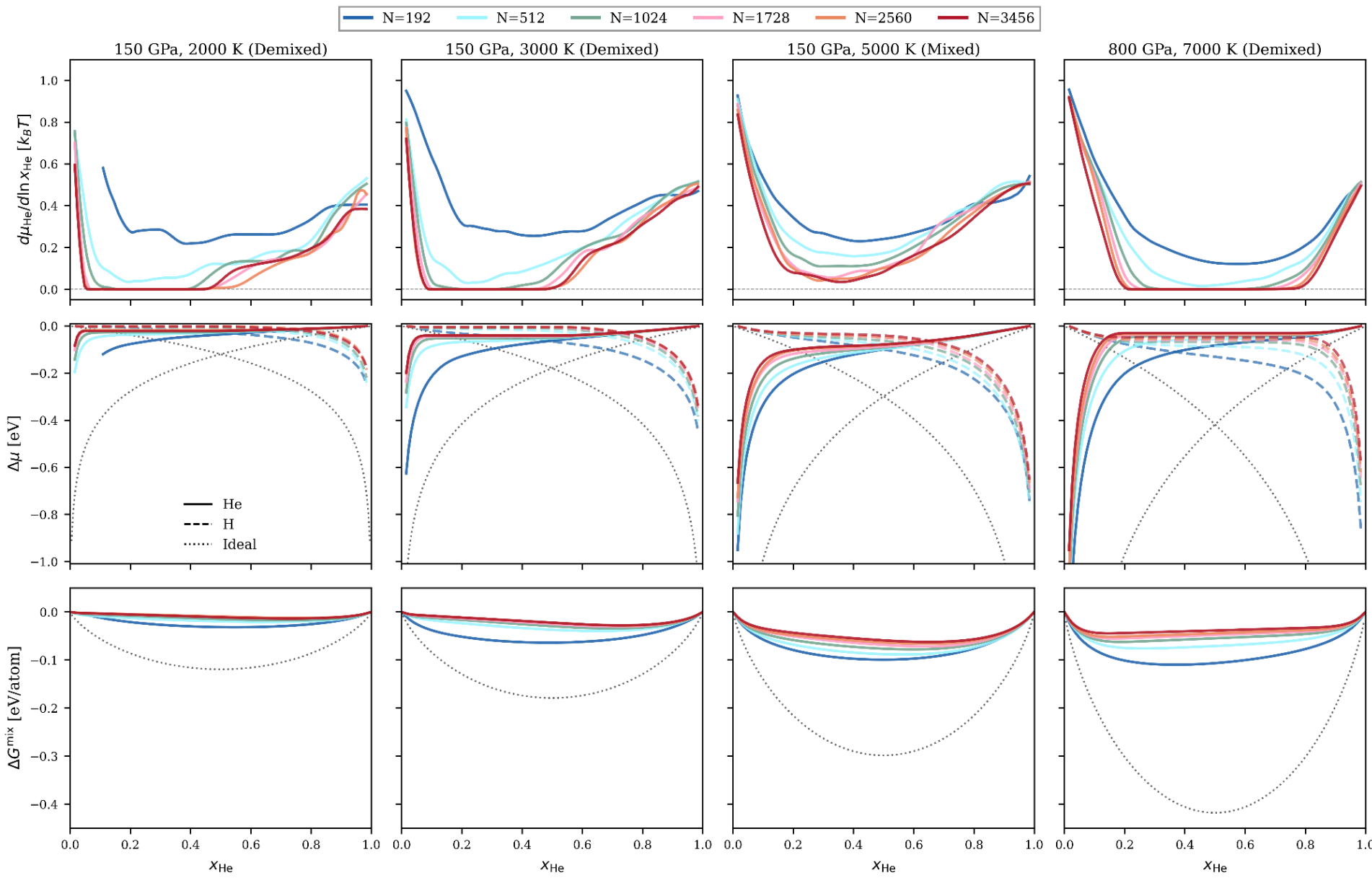

As shown in the figure above, we found that the mixing free energy requires larger system sizes (N >= 1024) to reach convergence. We therefore confirm that the free-energy calculations and resulting phase diagram reported in the main text, which are based on N=3456 simulations, are fully converged with respect to system size.

2. The results rely on DFT reference data generated with the PBE, vdW-DF, and HSE functionals under extreme pressure–temperature conditions. Could the authors comment on the expected reliability of these exchange–correlation functionals in this regime, and on whether possible functional deficiencies could systematically affect the predicted H/He mixing free energies and immiscibility boundary?

Authors:
The referee asked whether the selected PBE, vdW-DF, and HSE functionals are reliable under extreme pressure-temperature conditions, and the effect of possible functional deficiencies on the predicted immiscibility boundary. To address this, in what follows, we discuss:

1) The performance of the three DFT functionals in reference to available QMC benchmark studies, particularly for the equation of state and mixing enthalpy.
2) The differences among the DFT functionals in the estimation of band gaps of high-pressure hydrogen, and their effect on the computation of the immiscibility boundary.

We find that DFT calculations align well with existing QMC benchmarks for high-pressure H/He mixtures, although some functional-dependent uncertainties remain. However, the choice of functional introduces a systematic uncertainty in the predicted immiscibility boundary at lower pressures, since the different functionals yield different band-gap predictions for hydrogen. At higher pressures, we do not find clear evidence for a comparable systematic bias. A detailed discussion is provided below.

1) Here, we further discuss the selected functionals that reflect different physical approximations: the semilocal PBE functional, the nonlocal dispersion-corrected vdW-DF functional, and the hybrid exact-exchange-based HSE functional, based on available QMC benchmark tests that included explicit description of electron correlations for high-pressure hydrogen (Cozza et al.,

2026; DOI: https://doi.org/10.1103/yrk6-ryps) and H/He mixtures (Clay et al., 2016; DOI: 10.1103/PhysRevB.93.035121):

a) Semilocal PBE is widely used and computationally efficient, but the previous QMC benchmark study (Clay et al., 2016) has shown that it does not reproduce the H/He mixing enthalpy with sufficient accuracy, even though it gives a reasonable equation of state, as shown in the figure below.
b) The local vdW-DF functional is nonlocal dispersion corrected and incorporates correlation effects that are absent in PBE and has been shown to improve the description of hydrogen metallization and to yield better agreement with QMC for H/He mixing enthalpy (Clay et al., 2016). However, vdW-DF is not necessarily superior in general. vdW-DF can also overestimate pressures relative to QMC more than PBE (Clay et al., 2016), which has been shown in Fig. 2(c) of the main text.
c) The nonlocal HSE provides a complementary perspective because combining the PBE exchange energy with the exact HF exchange energy can improve the description of electronic structure changes in dense hydrogen, especially near metallization (Cozza et al., 2026), even if its performance for H-He mixing thermodynamics has been much less extensively benchmarked than that of PBE and vdW-DF due to the higher computational cost (Clay et al., 2016).

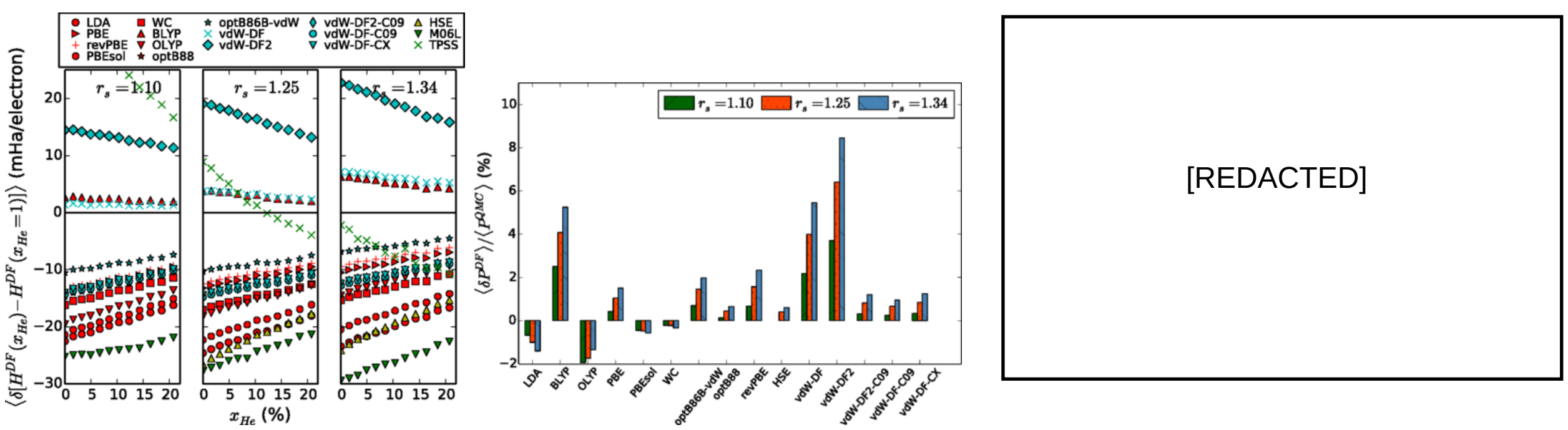

Figure: QMC benchmark results for mixing enthalpy (left panel), pressure calculations (middle panel), energy calculations (right panel) for different XC functionals at different densities over all helium concentrations, which were taken from Clay et al. (2016) (DOI: 10.1103/PhysRevB.93.035121).

Based on the QMC benchmarks, we conclude that all of the selected XC functionals are capable of capturing the essential thermodynamics of high-pressure H/He mixtures, although with different levels of accuracy for specific properties.

2) As discussed in the main text, we hypothesize that the underestimation of the hydrogen band gap by PBE can shift the predicted immiscibility boundary toward lower P-T conditions. For example, Fig. 2(g) in the main shows that PBE predicts H/He demixing at 100 GPa, whereas vdW-DF and HSE do not, consistent with the discussion that hydrogen atomization can promote H/He demixing. At higher pressures, however, the immiscibility boundaries predicted by the three functionals are broadly consistent, differing by <500 K. We further verified such consistency by fitting the RK thermodynamic model for each functional, and the resulting immiscibility boundaries also show good agreement; see Fig. 3(c).

Therefore, our results indicate that possible functional deficiencies can systematically affect the predicted H/He immiscibility boundary at lower pressures, while this effect becomes less pronounced at higher pressures.

3. The simulations are based on molecular dynamics using machine-learning potentials trained on finite-temperature DFT data. Could the authors comment on the possible role of nuclear quantum effects, in particular zero-point energy, on the H/He mixing free energy and immiscibility boundary, especially near the molecular–atomic hydrogen transition?

Authors:
We thank the reviewer for pointing out the relevant work on isotope effects on high-pressure H/He mixtures. We agree that nuclear quantum effects (NQEs) can play a role in high-pressure H/He systems, including both the hydrogen metallization and demixing boundary. To address this, we performed additional ring-polymer path-integral molecular dynamics (PIMD) simulations using our vdW-DF N2P2 MLP to explicitly quantify the impact of NQEs on:

1) the hydrogen atomic-molecular transition
2) the H/He demixing free energy and hence the immiscibility boundary

We find that inclusion of NQE for high-pressure H/He mixtures lowers the hydrogen atomic-molecular transition temperature by up to about 450 K, but has only a minor effect on enhancing H/He immiscibility. As a result, NQEs do not alter the main conclusions of the present work.

Details of PIMD simulations are discussed below:

1) To address NQEs in H/He mixtures, we performed PIMD simulations via the i-PI interface coupled with LAMMPS, using the vdW-DF N2P2 MLP. In particular, we employed eight beads over the investigated temperature range in PIMD simulations, based on convergence benchmarks in prior work (e.g., Hinz et al., 2020, and Van De Bund et al., 2021). Consistent with the previous PIMD protocol for high-pressure hydrogen, each PIMD simulation begins with a 10-ps equilibration in the NPT ensemble, followed by an 80-ps production run in the NVT ensemble. During the NPT equilibration stage, pressure is controlled using the isotropic stochastic barostat, with a relaxation time of 500 fs. Temperature is controlled using the stochastic path integral Langevin equation thermostat (PILE_L) with a relaxation time of 50 fs. A system size of N=2560 and a timestep of 0.2 fs were selected to ensure numerical convergence of MD simulations and reduce the computational cost.
2) **Atomic–molecular transition temperature of hydrogen.** For an H/He mixture with x_{He} = 0.05, inclusion of NQEs lowers the atomic–molecular transition temperature of hydrogen by up to 450 K, as shown in the following table and figure:

TABLE V. The atomic–molecular transition temperature of hydrogen T_{AM} in a H/He mixture of $x_{He} = 0.05$ from MD and PIMD at 150 and 300 GPa, using the vdW-DF N2P2 MLP.

Method	T_{AM} at 150 GPa	T_{AM} at 300 GPa
MD	2520 K	1449 K
PIMD	2190 K	993 K

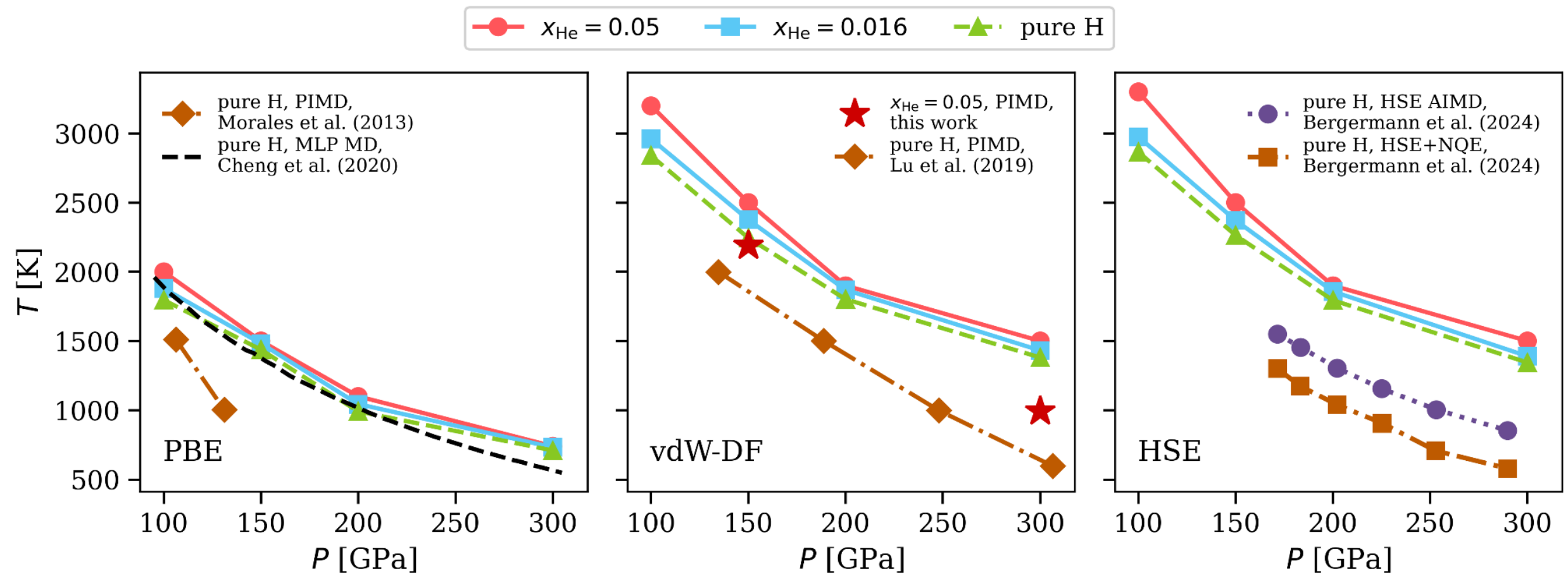

Figure: Comparison of the atomic-molecular transition temperatures of hydrogen for H/He mixtures with x_{He} = 0.05 and x_{He} = 0.016, and for pure hydrogen, based on PBE (left), vdW-DF (middle), and HSE (right). Red stars represent the transition temperatures with x_{He} = 0.05 from our PIMD simulations based on the vdW-DF N2P2 MLP at 150 and 300 GPa. The dashed black line denotes the transition boundary for pure hydrogen reported in the previous PBE MLP study (Cheng et al., 2020). The purple dotted line indicates the transition boundary for pure hydrogen in an earlier AIMD study (Bergermann et al., 2024). Brown dot-dashed lines correspond to phase boundaries of pure hydrogen with NQEs reported by previous DFT-based studies (PBE: Morales et al., 2013; vdW-DF: Lu et al., 2019; HSE: Bergermann et al., 2024).

Such a decrease in the atomic-molecular transition temperatures of hydrogen mostly originates from the zero-point energy (ZPE) in molecular hydrogen, which weakens the covalent bond and promotes molecular dissociation. This finding is consistent with previous DFT-based (Morales et al., 2013; Lu et al., 2019; Hinz et al., 2020; Van De Bund et al., 2021; and Bergermann et al., 2024) and MLP-based studies (Tenti et al., 2025) of dense hydrogen that explicitly account for NQEs.

3) To assess **the effect of NQEs on H/He demixing**, we conducted both classical MD and PIMD simulations under three thermodynamic conditions relevant to planetary conditions: 1) 150 GPa and 2000 K, corresponding to molecular hydrogen; 2) 150 GPa and 3000 K, corresponding to atomic hydrogen; and 3) 400 GPa and 2000 K, corresponding to atomic hydrogen. At each P-T condition, we varied x_{He} from 0.016 to 0.98 across at least 24 compositions.

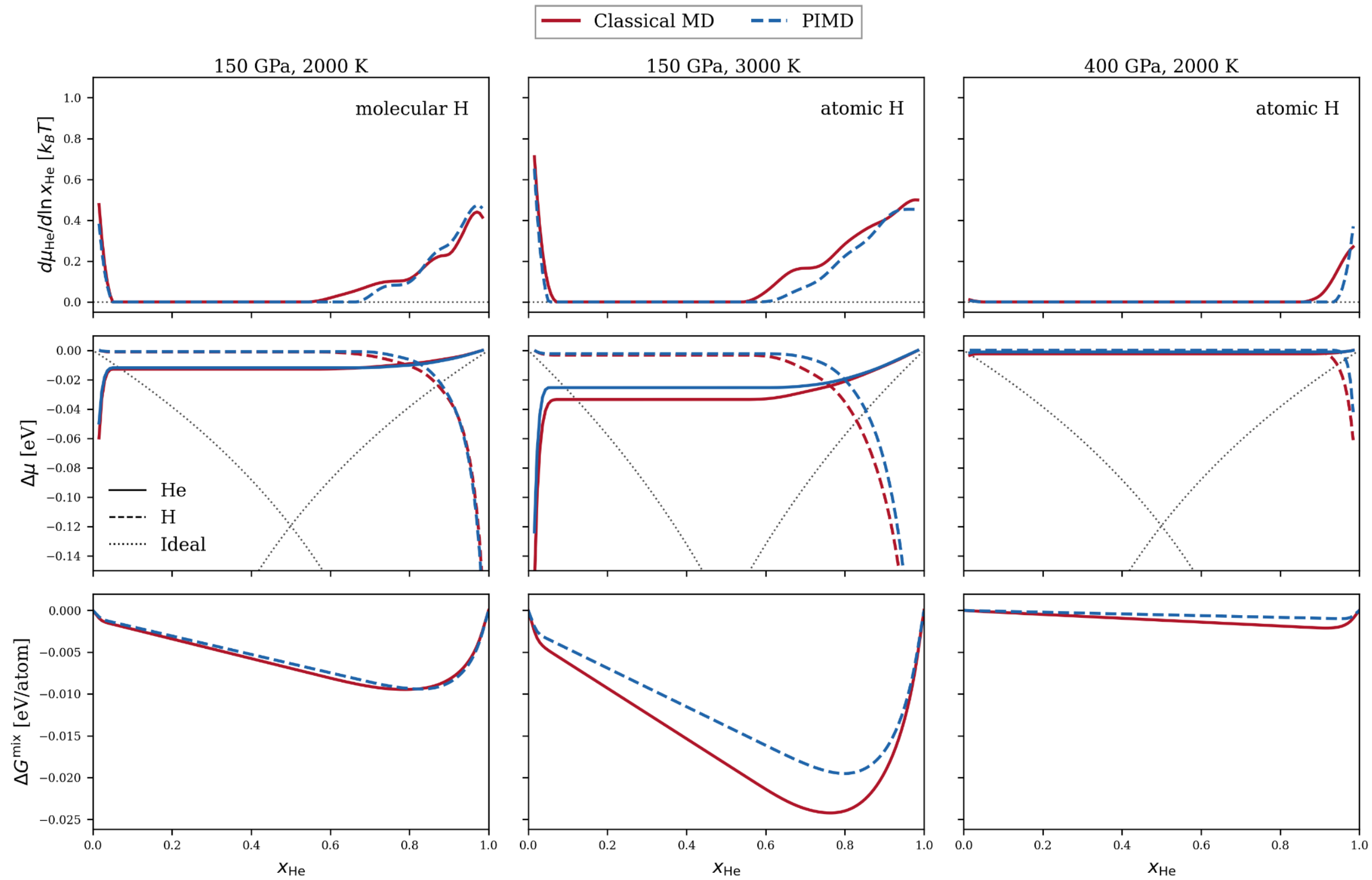

Figure: Thermodynamic behaviors of H/He mixtures predicted by the vdW-DF N2P2 MLP with classical MD (red) and PIMD simulations (blue). Each column represents a distinct thermodynamic condition: 1) 150 GPa and 2000 K (molecular H), 2) 150 GPa and 3000 K (atomic H), and 3) 400 GPa and 2000 K (atomic H).

As shown above, inclusion of NQEs (blue dashed lines) systematically increases ΔG^{mix} and thus enhances the H/He immiscibility. This aligns with previous DFT findings that NQEs remain positive at lower pressures (https://doi.org/10.18453/rosdok_id00002384). The estimated maximum NQE of 6 meV/atom at 150 GPa and 3000 K agrees with the 10 meV/atom upper bound previously reported at 100 GPa and 3000 K. As a result, inclusion of NQEs has only a minor effect on enhancing H/He immiscibility.

To sum up, we performed PIMD simulations to explicitly include NQEs in high-pressure H/He mixtures and found that they shift the hydrogen atomic-molecular transition to lower temperatures and enhance H/He demixing. We have revised the manuscript accordingly and added the relevant prior references.

4. If helium rain occurs, the local helium concentration xHe will evolve over time as helium precipitates and redistributes. Could the authors comment on how a decreasing local xHe would affect the miscibility behavior and immiscibility boundary, and whether their thermodynamic results can be used to infer the qualitative time evolution of helium rain in planetary interiors?

Authors:
The referee asked how a decrease in the local helium fraction x_{He}, as helium precipitates and redistributes, would affect the miscibility behavior, and whether our thermodynamic results can be used to infer the qualitative time evolution of helium rain in planetary interiors. To address these concerns, we have now discussed:

1) The thermodynamic dependence of the H/He immiscibility boundary;
2) The consequences and implications for H/He demixing and redistribution of helium during planetary evolution.

Our main conclusion is that a decreasing local x_{He} generally makes further H/He demixing less favorable at the same P-T condition, while the resulting helium enrichment in the deeper planetary interior can further promote continued demixing there. Moreover, the lower H/He immiscibility boundary derived from this study suggests that helium rain initiates later in the evolution of Jupiter and Saturn than previously estimated. However, a full description of the time evolution of helium rain in planetary interiors requires a coupled planetary-evolution model.
Detailed discussion follows:

1) Since the H/He immiscibility boundary shifts to lower temperatures as x_{He} decreases (Fig. 2 in the main text), a decreasing local helium fraction makes further H/He demixing less likely. In other words, as helium rains out and the overlying region becomes He-poor, further demixing there becomes progressively less favorable. Conversely, the deeper planetary interior into which helium settles becomes He-enriched, which can make phase separation more favorable at depth.
2) This suggests a self-consistent picture in which helium rainout progressively depletes helium from shallower layers while enriching deeper layers. Consequently, a compositional gradient in Gas Giants could be established over time. In this sense, our thermodynamic results provide a useful baseline for how helium redistribution may alter the location and extent of the immiscibility region during planetary evolution.
   Moreover, the lower H/He immiscibility boundary derived in this study suggests that helium rain begins later in Jupiter and Saturn than predicted by previous estimates. As these planets continue to evolve and cool, larger regions of their interiors are expected to cross the predicted H/He immiscibility boundary, thereby allowing helium rain to persist. However, once helium rain begins, the planetary interior can no longer be described by thermodynamics alone or treated as a homogeneous adiabatic system. Composition redistribution, mass transport, and the release of gravitational and thermal energy all modify the thermal structure (e.g., P-T conditions) and may further affect the kinetics of helium rain.
   Therefore, while our results constrain the equilibrium tendency for demixing as a function of P, T, and x_{He}, quantitative predictions for the time evolution of helium rain in planets require a coupled planetary-evolution model that includes transport and energy-balance effects.

We thank the referees for their further comments on the revised manuscript. Reviewer #1 recommends publication, while Reviewers #2 and #4 ask for additional clarifications and analyses. We address each comment below. Reviewer #2's remaining points helped us sharpen the physical interpretation of our results, in particular the relationship between hydrogen atomization, nuclear quantum effects, and H/He miscibility, which we now discuss more explicitly in the manuscript. All changes to the manuscript and Supplementary Information are highlighted in blue.

Reviewer #1 (Remarks to the Author):

Regarding the replies from the authors to the reviewer comments, I believe the authors did a good job attempting to address the concerns. The authors clearly went to great lengths to provide numerical evidence and tests for most of the reviewers concerns. I think the manuscript is clear, provides a comprehensive study of H/He miscibility using ML potentials and provides an accurate answer to the miscibility question in terms of the generated potentials. For these reasons, I think the paper should be published.

I must say, on the other hand, that the question of whether the ML potentials faithfully represent the associated DFT-based phase diagram for the mixture is still an open question. The free energy integration presented in the latest version of the paper does suggest that this is indeed the case, but in my opinion it is still something that we must assume/accept. Finally, the paper does not address the limitations of DFT and the associated Born-Oppenheimer approximations inherent in the calculations, so questions still remain unfortunately.

Authors:
We thank the referee for the positive assessment.

We also thank the referee for raising two important caveats that we now address directly in the manuscript:

- MLP accuracy compared to DFT reference. We agree that the accuracy of our study hinges on the MLP errors with respect to the DFT ground truth. We hope that the free-energy perturbation (FEP) analysis performed in the previous round provides a quantitative, rather than assumed, answer to this question. Specifically, by recomputing the vdW-DF DFT energies for 3,500 uncorrelated N = 1024 configurations at each P-T-x_He condition and reweighting the MLP-derived ΔG^{mix} to the DFT level, we found the FEP correction to be below 10 meV/atom at the most demanding condition (150 GPa, 2000 K) and essentially negligible at the higher temperatures most relevant to demixing (Fig. 1g, k). Propagating the maximum error would shift the predicted H/He immiscibility boundary by less than 1000 K, **which we have now stated explicitly in the Discussion**. While we agree that this does not amount to a proof, it does provide a bounded, quantitative uncertainty estimate for the MLP.

- DFT and Born-Oppenheimer limitations. We agree that our calculations establish the H/He immiscibility boundary within the Born-Oppenheimer DFT framework represented by the fitted MLPs, rather than an exact quantum solution. We have therefore revised the Discussion to state more explicitly that the remaining uncertainty is dominated by the residual MLP errors, underlying DFT approximation, and the Born-Oppenheimer treatment.

  We have **added a short paragraph at the beginning of the Discussion** that acknowledges (i) the residual XC-functional sensitivity (<500 K spread across PBE, vdW-DF, and HSE), (ii) the absence of explicit electronic correlation beyond what is captured by the chosen functionals, with reference to available QMC benchmarks for H/He mixtures (Clay et al., 2016) and pure hydrogen (Cozza et al., 2026), (iii) the use of Born–Oppenheimer MD with finite-temperature Mermin–Kohn–Sham occupations, which captures thermal electronic effects but neglects nonadiabatic coupling, (iv) the main MD results were performed under a classical MD framework without explicit nuclear quantum effects (NQEs), although our PIMD simulations suggest that NQEs are not important for the H/He immiscibility boundary.

"The immiscibility boundaries reported here should be interpreted within the accuracy of the underlying Born-Oppenheimer DFT framework, MLP-driven MD, and the numerical free-energy calculations. One systematic uncertainty arises from the exchange-correlation functional (PBE, vdW-DF, and HSE predict immiscibility boundaries that differ by less than 500 K at the protosolar helium abundance). Meanwhile, the MLP-to-DFT free-energy perturbation tests indicate the MLP error is not important: MLP and underlying DFT predict statistically indistinguishable mixing free energies at temperatures relevant to the immiscibility boundary, and MLP errors would lead to a shift of less than 1000 K in the worst-case scenario analysis (see the Supplementary Information). The PIMD simulations indicate that NQEs would only slightly affect the immiscibility boundaries by less than 200 K. Thus, although the absolute demixing temperatures remain condition-dependent and uncertain at the level of several hundred kelvin, the lowering of the H/He immiscibility boundary relative to previous small-cell AIMD estimates is robust across the three exchange-correlation functionals tested here."

Reviewer #2 (Remarks to the Author):

I would like to thank the authors for their thorough response and testing.

The paper now makes a number of conclusions which I find strange. That's fine of course, the whole point of research is to reveal new physics, but they are somewhat hidden in the manuscript which risks being misleading.

Authors:
We thank the referee for the constructive comments. We have extensively revised the manuscript again in response to the comments. We hope the conclusions are clear now, with uncertainties explicitly stated.

Firstly, although the methodology is impressive, there are very large sources of error. It is quite hard to quantify these for a general reader, because they depend on P,T and concentration. But it seems to me that the uncertainty is roughly

500K (10%) cutoff dependence in g(r).
1000K from choice of XC functional
400K in atomisation from NQE
100K (10meV) from error in MLP training
+ some unquantified error from the S(0) method.

So essentially the uncertainty in the calculation is close to the reported effects. Uncertainty in conclusions needs to be stated up front in the abstract, not buried in SM.
Authors:
We thank the referee for compiling this useful breakdown of the dominant error sources. We agree that the uncertainty should be stated more clearly, and we have therefore revised the **Abstract** and **Discussion**. At the same time, we would like to clarify that **the listed contributions do not all enter the H/He immiscibility boundary, and therefore should not be added as independent errors on the demixing temperature.** As we detail below, the uncertainties that directly affect the H/He immiscibility boundary are the XC functional choice, the MLP training error, and the NQEs on the immiscibility boundary. Such errors are smaller than the reported discrepancy with previous AIMD results.

500K (10%) cutoff dependence in g(r): The g(r) does not enter our calculation or analysis for determining the chemical potentials of H and He in the mixture, so it does not affect our computed H-He miscibility phase boundary.
We assume that the referee is referring to the analysis on how the cutoff changes the determination of the atomic-molecular transition temperature provided in our last response letter:

> We examined the sensitivity of the atomic–molecular transition temperature of hydrogen to the cutoff distance (r_2) used in Eq. S1. In particular, we tested three different criteria (1.05 Å, 1.10 Å, and 1.15 Å) based on vdW-DF and the resulting phase boundaries of hydrogen atomization in a H/He mixture of x_{He} = 0.05 are presented in the following plot:

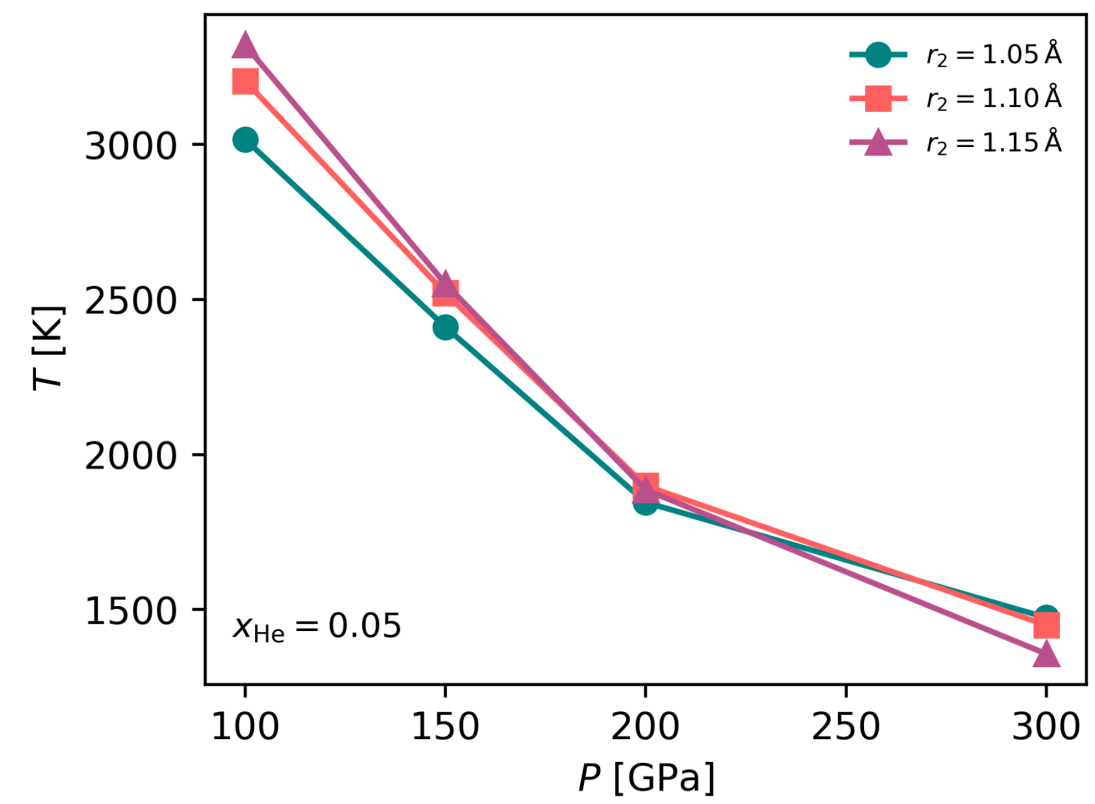

The resulting variation in the transition temperature is less than 10%. This indicates that the atomisation temperature is not very sensitive to the specific choice of cutoff (r_2=1.10 Å is used in the main). To be consistent with the previous study on pure hydrogen (Cheng et al., 2020), we reported the atomic–molecular transition boundary with a cutoff r_2=1.10 Å in the main.

The cutoff dependence discussed only refers to the structural criterion used to locate the hydrogen atomic-molecular transition, not to the calculation of the H/He chemical potentials or the immiscibility boundary. The H-H g(r) cutoff is used only to interpret whether hydrogen is predominantly molecular or atomic, and to draw the dotted atomic-molecular transition lines. **Thus, the estimated ~300 K variation in the H atomization line does *not* introduce uncertainty in the H/He immiscibility boundary.**

1000K from choice of XC functional: We agree that the exchange-correlation functional is one of the largest remaining sources of uncertainty. This is precisely why we used three different functionals, PBE, vdW-DF, and HSE, which represent different approximations to exchange and correlation. For the H/He immiscibility boundary at the protosolar helium abundance, the predicted boundaries from the three functionals **differ by less than about 500 K** over much of the planetary pressure range, with larger ambiguity mainly near the low-pressure region where hydrogen atomization also changes rapidly. We have revised the manuscript to state more clearly that the DFT functional choice remains the dominant systematic uncertainty.

400K in atomisation from NQE: The ~450 K shift caused by nuclear quantum effects **applies to the hydrogen atomic-molecular transition temperature, not directly to the H/He immiscibility boundary**. Our PIMD calculations show that NQEs lower the hydrogen atomization temperature, as expected from zero-point weakening of molecular H-H bonds. When we compute the H/He mixing free energy directly with PIMD, the effect is much smaller: the maximum correction we observe is about 6 meV/atom under the tested conditions, and it only modestly enhances immiscibility. We have clarified this point in the Discussion.

100K (10meV) from error in MLP training: We agree that ordinary MLP fitting errors alone are not sufficient to assess uncertainty in a phase boundary. For this reason, we went beyond RMSE metrics and promoted the MLP-derived ΔG^{mix} to the DFT level using free-energy perturbation. These FEP tests show no systematic composition-dependent bias in the MLP free

energies. The largest correction to ΔG^{mix} is within about 10 meV/atom at the lowest-temperature condition tested, while at temperatures relevant to the immiscibility boundary, the MLP and the DFT mixing free energies are statistically indistinguishable.
We performed a worst-case scenario analysis based on the maximum error at the low temperature, we estimate that this MLP-to-DFT correction would shift the inferred boundary by less than 1000 K in this case, although we expect the typical error is much less than that due to the much smaller difference between the MLP and the DFT mixing free energies at relevant temperatures. We have kept this as a conservative upper bound rather than assigning a universal 100 K value.

Some unquantified error from the S0 method: We agree that the practical numerical uncertainty associated with the S0 procedure should be discussed clearly. The S0 method itself is not an empirical approximation; it follows from fluctuation theory and relates chemical-potential derivatives to the partial structure factors extrapolated to the k->0 limit. The possible numerical errors arise from finite simulation size, sampling, and extrapolation of S(k) to k=0. We have directly tested these sources by computing S(k) and ΔG^{mix} for a range of system sizes from N=64 to N=3456. These tests show that the long-wavelength structure factors and mixing free energies are converged for the system sizes used in the main calculations. Therefore, while the S0-related statistical uncertainty is not a single simple temperature number, we find **no evidence that it is comparable to the main physical shifts discussed in the paper.**
It is worth pointing out that the S0 method has been applied to a number of problems, including the solubility of molecular crystals (Reinhardt et al., 2023; Herboth et al., 2025), determining phase equilibria in mixtures (Cheng et al., 2023; Trinkle, 2025), computing adsorption isotherms for confined fluids (Schmid and Cheng, 2023), and the formation of azeotropes in binary mixtures (Wang and Cheng, 2024), with proven high accuracy.

Overall, we agree with the referee that the uncertainties come from different sources and need to be explicitly stated. However, the individual uncertainty estimates listed above affect different parts of the analysis: some concern the hydrogen atomization diagnostic, some concern the DFT reference, and some concern the MLP or S0 numerical implementation. They should therefore not be summed as a single uncertainty in the H/He immiscibility temperature.

Now in the Abstract section, we have updated the following sentence:
“Across the pressure range of 100–1000 GPa, the demixing temperatures are typically ~2000 K lower than previous *ab initio* simulations using small system sizes, and this discrepancy is larger than the estimated error from the chosen density functional, machine learning potential, neglect of nuclear quantum effects, and statistical uncertainties.”

In the Discussion section, we also add the following statement to explicitly discuss the combined errors:
“The immiscibility boundaries reported here should be interpreted within the accuracy of the underlying Born-Oppenheimer DFT framework, MLP-driven MD, and the numerical free-energy calculations. One systematic uncertainty arises from the exchange-correlation functional (PBE, vdW-DF, and HSE predict immiscibility boundaries that differ by less than 500 K at the

protosolar helium abundance). Meanwhile, the MLP-to-DFT free-energy perturbation tests indicate the MLP error is not important: MLP and underlying DFT predict statistically indistinguishable mixing free energies at temperatures relevant to the immiscibility boundary, and MLP errors would lead to a shift of less than 1000 K in the worst-case scenario analysis (see the Supplementary Information). The PIMD simulations indicate that NQEs would only slightly affect the immiscibility boundaries by less than 200 K. Thus, although the absolute demixing temperatures remain condition-dependent and uncertain at the level of several hundred kelvin, the lowering of the H/He immiscibility boundary relative to previous small-cell AIMD estimates is robust across the three exchange-correlation functionals tested here."

A new conclusion is that the solubility lines is unaffected by NQE, even though NQE moves the metallization/atomization temperature by ~ 500K. Scientifically, this is far more interesting than a calculated phase boundary with large errors. Is the enthalpy of mixing somehow unrelated to metallization? It seems like new chemistry - at least to me. Please explain?

Authors:
We thank the referee for highlighting this point. It is indeed an interesting physical observation. The explanation is as follows:

As the referee pointed out, NQEs have a relatively large effect on the atomic-molecular transition of hydrogen, and they reduce the H atomic-molecular transition temperature by ~ 500K. The NQE here is mainly due to the zero-point energy (ZPE) of the H-H bond, and the amount of ZPE in the system is mainly determined by the population of molecular versus atomic hydrogen, as discussed in van de Bund et al., 2021 (10.1103/PhysRevLett.126.225701). Across the transition, the ratio between molecular and atomic hydrogen differs, and thus the amount of ZPE changes drastically.

In contrast, the H/He demixing has a smaller and indirect effect on the molecular/atomic hydrogen ratio: when a He-rich phase precipitates, leaving behind a solution with a lower He fraction, this influences the molecular/atomic hydrogen ratio in both the He-rich and the He-poor phases, as a higher He concentration tends to slightly stabilize molecular hydrogen (see Fig. 2(d-f)). However, this is a secondary effect, differing from the direct and large molecular/atomic hydrogen ratio change across the atomic-molecular transition line of hydrogen.

Another reason for the smaller NQE in the H/He demixing is that the associated temperature range is 2000-6000 K, which is higher than the temperature range of 1000-3000 K for the atomic-molecular transition. The higher the temperature, the less important the NQEs (van de Bund et al., 2021; 10.1103/PhysRevLett.126.225701).

In Fig S9 it is shown that in the "mixed" atomic region 3000K/150GPa there is a big peak and the H2 distance, and at 5000K it is even bigger (for higher He%). Does it mean that the extra helium is somehow significantly driving H2 formation? What is the chemistry behind that? I presume it can be seen in the DFT calculation.

Authors:
The referee correctly identifies a real effect: adding helium does promote molecular hydrogen formation. This is the same phenomenon underlying the upward shift of the hydrogen atomization temperature with helium fraction reported in the main text (Fig. 2d–f), consistent with previous QMC (Mazzola et al., 2018; 10.1103/PhysRevLett.120.025701) and DFT (Vorberger et al., 2007, 10.1103/PhysRevB.75.024206; Preising and Redmer, 2026, 10.1063/5.0312028) studies.

The physical origin can indeed be understood from the electronic structure in DFT calculations. In pure hydrogen, increasing pressure enhances intermolecular electronic overlap between neighboring H2 molecules. Due to Pauli exclusion, electrons are driven out of localized bonding states into more delocalized states, i.e., molecular dissociation.
With helium added, the stronger nuclear charge of He (Z=2) tightly localizes electrons around He and suppresses such intermolecular electronic overlap between hydrogen. As a result, neighboring H2 remains more electronically isolated and stable.

In the revised manuscript, we have explained this mechanism: “This is because the higher nuclear charge of He tightly localizes electrons and reduces the intermolecular electronic overlap between neighboring hydrogen molecules (Vorberger et al., 2007; Mazzola et al., 2018).”

I have now lost track of the big picture here with regard to enthalpy of solution: is it the case that enthalpy of mixing is positive in atomic and metallic hydrogen, so the solubility in the deep planet is driven by temperature rather than pressure. As I understand Fig 2d-f, the solubility boundary is where the red line crosses the black one: i.e. there are two solubility boundaries. Firstly, the enthalpy of mixing He in metallic H drives phase separation, then the high temperature overcomes this deeper in the planet. This would mean they calculated two physical miscibility boundaries in the planets from just one in the PT phase diagram. Is that correct? It seems like something that could be made clearer.

Authors:
We thank the referee for pointing out that the physical picture was not sufficiently clear.

The short answer is that there is **one H/He immiscibility surface** in the three-dimensional P-T-x_{He} space. A planetary interior profile is a one-dimensional path through this space. Depending on the shape of the planetary P-T profile and the local helium abundance, that path may intersect the same immiscibility surface zero, one, or two times. Thus, two crossings along a planetary profile do not imply two distinct phase diagrams or two different physical boundaries;

they are simply the entry and exit points of a finite immiscibility region along the planet's interior profile.

We have revised the Results to clarify the relation between the thermodynamic driving force for H/He demixing, the hydrogen atomic/metallic regime, and the way a planetary thermal profile intersects the immiscibility surface:

“The black curves in Fig. 2(d-f) represent the upper boundary of the immiscibility region in the P-T projection, while the planetary profiles indicate possible paths through this projected boundary. A crossing of a planetary profile with the boundary marks where the local thermodynamic state enters or exits the two-phase region for the relevant helium concentration.”

The story about helium rain remains confusing. In the introduction the authors state:

"Precipitate as helium-rich droplets that sink toward the planetary core under gravity. This phase separation and subsequent gravitational settling of helium rich components are commonly referred to as “helium rain”

Which is how I understood it from detailed reading of Helled etc., but not how almost every general reader understands it. As I said before, its deeply misleading to call this one-off process "rain", but that's perhaps not the author's fault. I suspect if they state clearly that their model predicts at most a one-off precipitation rather than a rain cycle they would increase their paper impact significantly. (Even a one-off event depends rather sensitively on the details of the planet formation.

Authors:
We thank the referee for the point related to the terminology regarding “helium rain”. We have added the following clarification to the introduction:
"We use 'helium rain' in the conventional astrophysical sense, to denote precipitation and gravitational settling of helium-rich droplets, not a cyclic meteorological process.”

This makes clear from the outset what physical process the paper actually constrains.

But now in Fig 1b they show the "rain" between the molecular and metallic regions. In general I think the planetary "core" means the heavy elements below the metallic hydrogen layer, not the metallic hydrogen layer itself. Certainly this is what Helled etc. mean when they talk about "fuzzy core" of Jupiter. So it seems Fig 1b is at variance with the introduction about where the rain occurs (this may just be terminology).

Authors:
We thank the referee for pointing out this possible ambiguity regarding the term “core”. We agree that, in planetary interior models, the term “core” usually refers to the heavy-element-rich central region. Also, we suppose the referee was referring to the schematic in Fig. 2b instead of Fig. 1b (pressure-density relation for H/He mixtures).

In our manuscript and Fig. 2b, the helium-rain region is intended to represent the H/He immiscibility region in the H/He envelope. This helium-rain region may lie near or overlap the molecular-to-metallic transition of hydrogen, but it is distinct from the heavy-element core. Thus, the schematic does **not** imply that the rain region is part of the heavy-element core, nor that the metallic hydrogen layer is being identified as the core.

Moreover, due to the finite solubility of helium in molecular hydrogen, helium rain is not necessarily confined to a metallic-hydrogen layer in planets, consistent with recent Jupiter interior models inferred from Juno's gravity field data (e.g.,
Militzer and Hubbard, 2024; 10.1016/j.icarus.2024.115955; see the figure below).

[REDACTED]

Figure: Planetary models for Jupiter, taken from Fig. 1 of Militzer and Hubbard (2024; 10.1016/j.icarus.2024.115955).

To avoid any confusion and additional complexity of the term "core", we have revised the terminology in the manuscript by replacing "core" with "interior", and we have updated Fig. 2(b) and its caption to clarify that the helium-rain region refers to H/He immiscibility in the H/He envelope of gas giants.

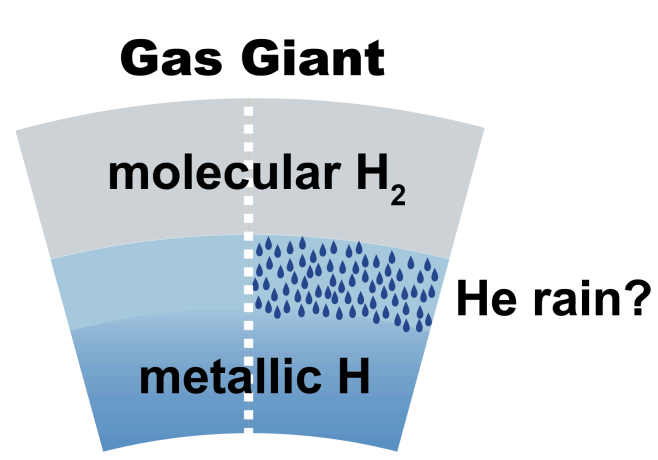

Figure: Updated Fig. 2b in the manuscript.

So my basic summary of the paper is that...

With the best available calculation methods it is still very difficult to get sufficient accuracy to say anything definitive about solubility in Jupiter and Saturn. However within the uncertainty of teh mode is it possible that there are zero, one or two miscibility boundaries in Jupiter, and either one of two in Saturn. The model has some very strange chemistry and unexplained wherein He in solution disrupts metallisation, forming more hydrogen molecules.

Authors:
We thank the referee for this summary, which helped us identify where the manuscript needed a clearer explanation. We agree that the H/He solubility boundary remains challenging to determine with absolute certainty, and that the implications for Jupiter and Saturn depend on both the calculated immiscibility boundary and the adopted planetary model. We have therefore revised the Discussion to state the uncertainty and conditional nature of the planetary conclusions more explicitly.

At the same time, we would like to clarify a few points. First, our calculation does not imply multiple distinct physical miscibility boundaries. As explained in the answer above, there is a single immiscibility surface in the P-T-x_He space. Second, as discussed above, the combined uncertainty in our computed miscibility boundaries is on the order of hundreds of kelvins, and does not qualitatively affect our main conclusions. Third, the apparent “strange chemistry” that helium slightly stabilizes molecular hydrogen relative to atomic/metallic hydrogen under some conditions has also been reported in previous QMC (Mazzola et al., 2018; 10.1103/PhysRevLett.120.025701) and AIMD (Vorberger et al., 2007, 10.1103/PhysRevB.75.024206; Preising and Redmer, 2026, 10.1063/5.0312028) studies. This is because the stronger nuclear charge of He enhances electron localization and suppresses intermolecular electronic overlap of molecular hydrogen, which stabilizes H-H bonds and shifts hydrogen dissociation to higher temperatures.

Reviewer #4 (Remarks to the Author):

The authors have addressed my comments very thoroughly. My remaining comments are relatively minor. I will continue to use the same numbers for the various points:

1. MLP training. I am still unclear on the MLP construction, particularly the use of the viral or lack thereof. I appreciate the authors' having done the extra work to train an MLP including the virial. The problem I still have though is that the RMS error in the viral is quite large: 4 GPa, as compared with 0.4 GPa in Deng et al. (2023) PRB.

Authors:
We thank the reviewer for pointing out the larger RMSE in our stress tensor compared to Deng et al. (2023) PRB https://journals.aps.org/prb/abstract/10.1103/PhysRevB.107.064103 where the authors fitted a MLP using DeepMD for configurations of $MgSiO_3$ at 2000 to 8000 K and pressures from ~0 to 220 GPa. Their MLP RMSE errors for the energies, atomic forces, and stresses are 4.9 meV/atom, 0.24 eV/Å, and 0.37 GPa, respectively.
The reviewer correctly pointed out that our training errors for the H/He system are significantly larger. The specific RMSE values depend on the specific setup and which architecture (N2P2, MACE, CACE) is used, and the representative values are:

TABLE II. Comparison of testing errors for MLPs trained with and without stress tensors.

Model	RMSE E [meV/atom]	RMSE F [meV/Å]	RMSE Stress [meV/Å^3]
MACE (E+F)	12.5	192.0	27.3
MACE (E+F+S)	12.9	195.6	24.7
CACE (E+F)	7.25	272.8	32.0
CACE (E+F+S)	7.97	305.3	32.7

Our H/He training set spans the temperatures 500-16000 K and pressure 1-1200 GPa, so the energy variances here are larger compared to Deng et al. Moreover, liquids are harder to describe compared to crystals, as the latter typically contain very similar atomic environments.

Our reasoning on why our errors are larger compared to Deng et al. (2023) PRB is simply that the underlying training set is different, and the direct comparison is not particularly meaningful.

To prove this point, we take the training set from Deng et al. (2023) PRB. The whole set has 39,909 frames. Our experience is that DeepMD typically requires more configurations to train compared to the more modern equivariant MLPs, so here we randomly selected 4,000 configurations for the training set, about 10% of the original set. We then train on the energies and forces of this 4000-configuration training set using MACE, with a hyperparameter setup that is similar to our H/He MACE model.

For full reproducibility, the training inputs/test inputs/trained potentials are provided in: https://www.dropbox.com/scl/fo/c2oqgeu336lhm50r2i1vk/APXtSpKtFo-Ximl1SgPhI1A?rlkey=l3tpqwmalrk0skqjt62or6gw1&dl=0

The test errors are:

	RMSE E [meV/atom]	RMSE F [meV/Å]	RMSE Stress/Virials [meV/Å^3]
train_4000	1.5	46.8	2.1
**Whole set**	**2.5**	**106.5**	**2.7**

Even though we only trained on 10% of the data, the MACE energy and force RMSE errors on the whole set are much smaller compared to the DeepMD results from Deng et al. (2023) PRB. The MACE stress RMSE (~0.4 GPa) is similar to DeepMD, even though we did not train on stress and the training set is smaller (10% of the original).

The smaller error from the MACE fit compared to the original DeepMD MLP is consistent with the current understanding in the MLP field: equivariant MLPs like MACE and CACE are considered to be amongst the most accurate architectures nowadays. N2P2 and DeepMD are considered to be cheap but have lower accuracy, as both omit many-body interactions beyond three-body terms.

To summarize, the training errors of MLPs are dependent on the specific data set and the system. The Deng et al. (2023) PRB set can produce MLPs with smaller errors, compared to our H/He set.

3. Equation 1. Thanks for the derivation, which makes sense. My question however was a different one. My question was about the right-hand side of the equation. Is there a simple argument one can make that would help me understand why the RHS must become very small in an inhomogeneous system? i.e. something having to do with the values of S_ab(k->0) in an inhomogeneous as opposed to a homogeneous system. I am not questioning the basic result, and indeed I can confirm myself using numerical values of S_ab(k->0) that you have now provided that the RHS is indeed very small for the demixed cases. I am looking for a simple qualitative argument. Hope this makes sense.

Authors:

We thank the reviewer for agreeing with our derivation and for the interest in a more intuitive interpretation of the right-hand side (RHS) of Eq. 1 in the manuscript, which is

$$\left(\frac{\partial \mu_{\mathrm{He}}}{\partial \ln x_{\mathrm{He}}}\right)_{T,P} = \frac{k_{\mathrm{B}}T}{x_{\mathrm{H}}S^{0}_{\mathrm{He-He}} + x_{\mathrm{He}}S^{0}_{\mathrm{H-H}} - 2\sqrt{x_{\mathrm{H}}x_{\mathrm{He}}}S^{0}_{\mathrm{H-He}}}.$$

To resolve such a concern, we first provide a qualitative explanation for why the RHS is 0 when demixing occurs. We then present the mathematical derivation to support the argument.

The denominator of the RHS of Eq. 1, $x_{\mathrm{H}}S^{0}_{\mathrm{He-He}} + x_{\mathrm{He}}S^{0}_{\mathrm{H-H}} - 2\sqrt{x_{\mathrm{H}}x_{\mathrm{He}}}S^{0}_{\mathrm{H-He}}$, measures **the magnitude of compositional fluctuations at large spatial scales** (i.e., k→0).
In a homogeneous system, large-scale compositional fluctuations are modest because no macroscopic H-rich/He-rich domains develop.
On the other hand, in an inhomogeneous system, H-rich and He-rich domains form. The concentration varies strongly across the system, and compositional fluctuations become very large. Consequently, the denominator becomes large, and the RHS approaches 0.

To prove that the RHS of Eq. 1 approaches 0 during demixing, we consider a binary A-B system, and let N_A and N_B be particle numbers, $N = N_A + N_B$, $x_A = N_A/N$, $x_B = N_B/N$. In reciprocal k-space, the density fluctuation $\delta\rho_\alpha(\mathbf{k})$ for species α is defined as:

$$\delta\rho_\alpha(\mathbf{k}) = \sum_{i\in\alpha} e^{-i\mathbf{k}\cdot\mathbf{r}_i} - \left\langle \sum_{i\in\alpha} e^{-i\mathbf{k}\cdot\mathbf{r}_i} \right\rangle, \quad \alpha = A, B.$$

Also, Hermitian symmetry in k-space gives $\delta\rho_\alpha(-\mathbf{k}) = \delta\rho_\alpha(\mathbf{k})^*$, so the partial structure factors are:

$$S_{\alpha\beta}(\mathbf{k}) = \frac{\langle \delta\rho_\alpha(\mathbf{k})\, \delta\rho_\beta(-\mathbf{k})\rangle}{\sqrt{N_\alpha N_\beta}},$$

and more explicitly, we can write down the structure factors as:

$$S_{AA}(\mathbf{k}) = \frac{\langle \delta\rho_A\, \delta\rho_A^*\rangle}{N_A}, \quad S_{BB}(\mathbf{k}) = \frac{\langle \delta\rho_B\, \delta\rho_B^*\rangle}{N_B}, \quad S_{AB}(\mathbf{k}) = \frac{\langle \delta\rho_A\, \delta\rho_B^*\rangle}{\sqrt{N_A N_B}}.$$

The composition imbalance $Y(\mathbf{k})$ in the system is defined as:

$$Y(\mathbf{k}) = x_B\, \delta\rho_A(\mathbf{k}) - x_A\, \delta\rho_B(\mathbf{k}),$$

with $|Y|^2 = YY^* = (x_B\, \delta\rho_A - x_A\, \delta\rho_B)(x_B\, \delta\rho_A^* - x_A\, \delta\rho_B^*)$.

Moreover, the variance of the composition imbalance $\langle|Y|^2\rangle$ takes the form:

$$\langle |Y|^2\rangle = x_B^2 \langle \delta\rho_A\, \delta\rho_A^*\rangle + x_A^2 \langle \delta\rho_B\, \delta\rho_B^*\rangle - 2x_A x_B \langle \delta\rho_A\, \delta\rho_B^*\rangle.$$

Substituting the structure factors into the above formula gives:

$$\langle |Y|^2\rangle = N x_A x_B \big(x_B S_{AA} + x_A S_{BB} - 2\sqrt{x_A x_B}\, S_{AB}\big),$$

which can be connected to the denominator of the RHS of Eq. 1.

Next, when setting **k**→0 and imposing particle-number conservation $\delta N_A + \delta N_B = 0$, the density fluctuations reduce to $\delta\rho_\alpha(0) = \delta N_\alpha$, so:

$$Y(\mathbf{k}\to\mathbf{0}) = x_B\,\delta N_A - x_A\,\delta N_B = (x_A + x_B)\,\delta N_A = \delta N_A = -\delta N_B,$$

Correspondingly, we can write down: $\langle|Y(\mathbf{k}\to\mathbf{0})|^2\rangle = \mathrm{Var}(N_A) = N^2\,\mathrm{Var}(x_A).$

Demixing leads to the coexistence of two phases. In other words, the atomic fraction of A, x_A, follows a bimodal distribution peaked in these two domains:

$$x_A = \begin{cases} x_A^\alpha & \text{prob. } p \\ x_A^\beta & \text{prob. } 1-p \end{cases}$$

The variance of x_A is expressed as:

$$\mathrm{Var}(x_A) = p(1-p)\Big(x_A^\alpha - x_A^\beta\Big)^2 = O(1), \quad (0 < p < 1).$$

Hence, the magnitude of $\langle|Y(\mathbf{k}\to 0)|^2\rangle$ is scaled as:

$$\langle|Y(\mathbf{k}\to\mathbf{0})|^2\rangle = N^2 p(1-p)(x_A^\alpha - x_A^\beta)^2 = O(N^2)$$

In the end, during demixing, the connection of $\langle|Y(\mathbf{k}\to 0)|^2\rangle$ to Eq. 1 follows:

$$\left(\frac{\partial\mu_{\mathrm{He}}}{\partial\ln x_{\mathrm{He}}}\right)_{T,P} = k_BT\,\frac{N x_{\mathrm{H}} x_{\mathrm{He}}}{\langle|Y(\mathbf{k}\to\mathbf{0})|^2\rangle} = \frac{k_BT\,x_{\mathrm{H}}x_{\mathrm{He}}}{N\,p(1-p)(x_{\mathrm{He}}^\alpha - x_{\mathrm{He}}^\beta)^2} \xrightarrow{N\to\infty} 0,$$

where the numerator is O(1) while the denominator grows as O(*N*). This demonstrates that, for an inhomogeneous system with the two-phase region, the RHS of Eq. 1 is zero.

6. Jog. I accept that the jog is an artifact of interpolation. Is it not possible to perform the interpolation in a slightly different way that makes the jog disappear?

Authors:
We thank the referee for the suggestion to reduce the interpolation artifacts in the critical curves (black lines) reported in Figs. 2(d, e, f).
To address this issue, we have updated the boundary-construction procedure by replacing local interpolation with a smoothing process constrained by a global fit to the data. As shown in the figures below, the updated critical curves remove the visible jog while preserving the physically relevant features.
Correspondingly, we have updated Figs. 2(d–f) in the manuscript.

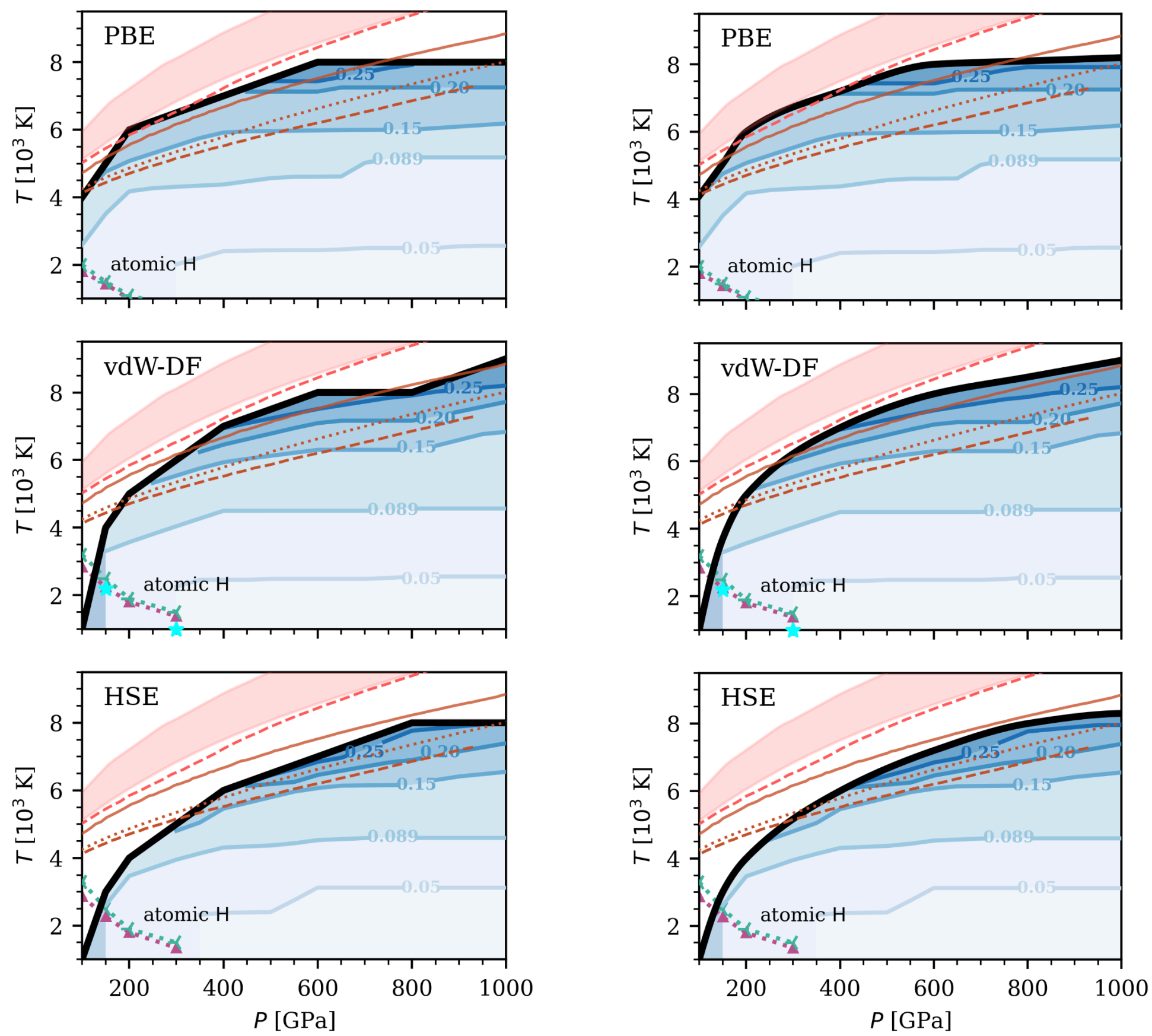

Figure: Comparison of the critical curves (black solid lines) before (left) and after (right) smoothing.

8. RK. I appreciate the density vs. pressure plots now provided. I think it would be even more useful to compare the EXCESS volume of the RK against Chabrier and Debras.

Authors:
We thank the referee for suggesting further analyses on the excess volume under the framework of the RK model.
To resolve this concern, we now computed the **excess volume** from the RK models and compared it directly with the tabulated H/He EOS of Chabrier and Debras (2021; DOI: 10.3847/1538-4357/abfc48) for all three DFT functionals (vdW-DF, PBE, HSE).

In detail, the excess volume of mixing V^{ex}_{RK} is obtained from the pressure derivative of ΔG^{mix} as $V^{ex} = -(\partial \Delta G^{mix}/\partial P)_T$, which takes the form:

$$V^{ex}_{RK} = - x_{He} x_H \sum (\partial \omega_i/\partial P)(1 - 2x_{He})^{i-1}$$

Because our ω_i fits are polynomial in T and P without T-P coupling (c + a1*T + a2*T^2 + b1*P + b2*P^2), there is no temperature dependence of V^{ex}_{RK}. The resulting V^{ex}_{RK} as a function of pressure for x_{He} = 0.09 is shown below for the three DFT functionals:

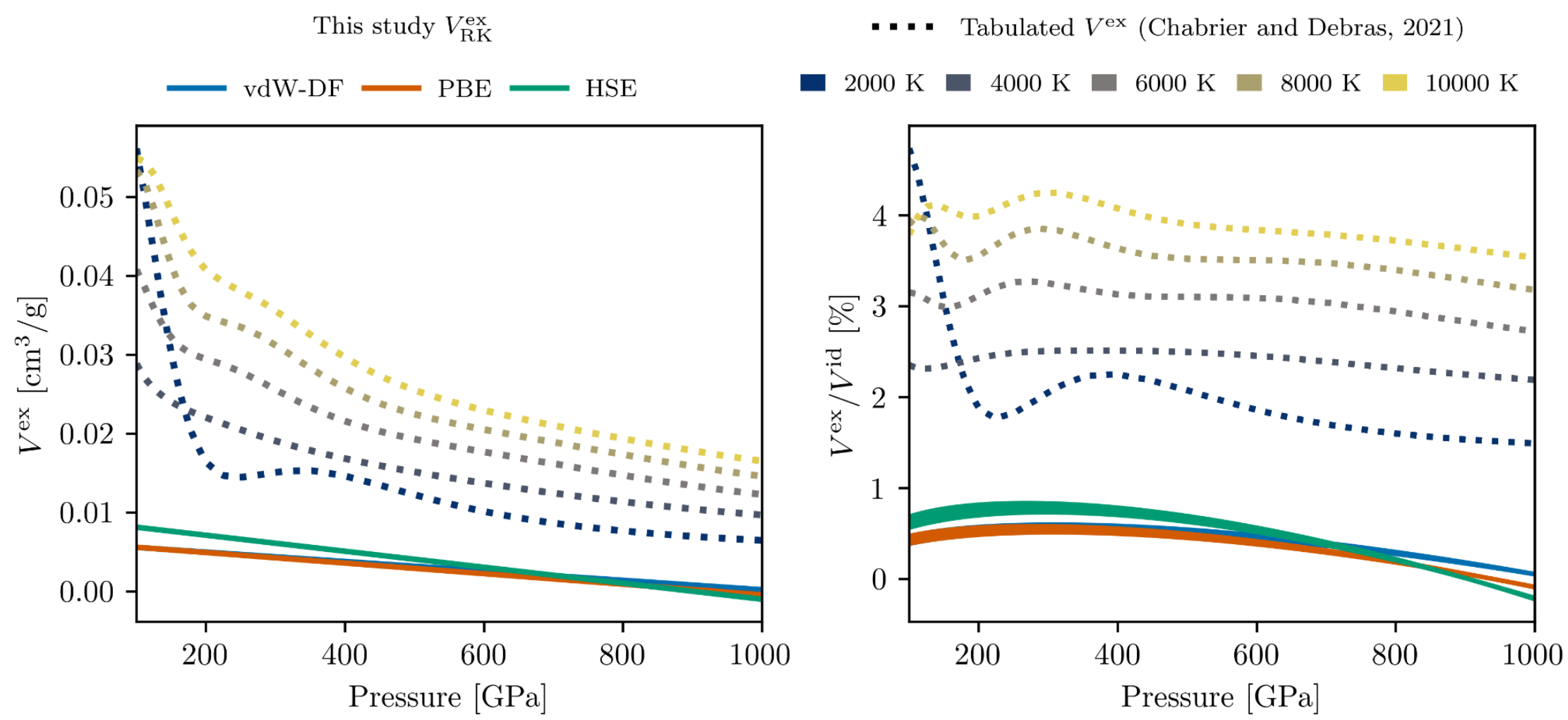

Figure: Excess volume V^{ex} derived from our RK models (solid lines) and previous studies of Chabrier and Debras (2021) (dotted lines) across 100-1000 GPa.
Left panel: the absolute V^{ex} value as a function of pressure for three DFT functionals (vdW-DF, PBE, and HSE).
Right panel: the percentage ratio of V^{ex} to the ideal-mixing volume V^{id} from the additive volume law. The width of the colored bands represents the variations of V^{id} across temperatures.

In the figure shown above, we also included the tabulated V^{ex} values from Chabrier and Debras (2021, DOI: 10.3847/1538-4357/abfc48) as colored dotted lines at different temperatures.

As a result, **the V^{ex}_{RK} terms are relatively small** (within 1% of the ideal-mixing volume V^{id}) and show weak XC-functional dependence. These findings are consistent with prior work by Chabrier and Debras (2021, DOI: 10.3847/1538-4357/abfc48), which also reports that V^{ex} remains **relatively modest** (up to about **4%**) and less sensitive to temperature (<3% variation at fixed pressures).

Reviewer #5 (Remarks to the Author):

I co-reviewed this manuscript with one of the reviewers who provided the listed reports. This is part of the Nature Communications initiative to facilitate training in peer review and to provide appropriate recognition for Early Career Researchers who co-review manuscripts.

Authors:
We thank the referee for participating in the co-review process and for their useful comments.

We thank the referees for their further comments on the revised manuscript. Reviewers #4 and #5 now recommend publication, and Reviewer #1 had already recommended publication in the previous round of review. Reviewer #2 asks for additional clarifications regarding the fidelity of the fitted MLPs and the physical miscibility boundaries. We address each comment below. We also ensure that the ability of the MLP to represent the DFT phase diagram is clearly discussed throughout the manuscript. All changes to the manuscript and Supplementary Information are highlighted in blue.

Reviewer #2 (Remarks to the Author):

I thank the authors for the careful reply.

Authors:
We thank the referee for examining both our technical aspects and the big picture claims.

Most of the review stage concentrated on the technical aspects of the paper, I would like to move now to the bigger picture:

Like referee 1, I take on trust "that the question of whether the ML potentials faithfully represent the DFT". The authors have worked hard to address it with respect to the phase boundary and the FEP and say essentially that it is not proved, but what more could they do? I sympathise, and note a simple resolution:

Authors:
We thank the referee for recognizing the effort we made in benchmarking and validating the MLPs. We now ensure that the ability of the MLP to represent the DFT phase diagram is clearly discussed throughout the manuscript, by adding sentences and phrases to the introduction, results, and the discussion sections.

The big difference between DFT and MLIP-predicted demixing temperature is now highlighted in the abstract - this seems like the main new finding of the paper. The difference is attributable to finite size effects, this being the only place that MLIPs have an advantage over DFT. In the SM, they run the MLIP on these too-small system and show that they can accurately reproduce the finite size errors. I dont fully understand why the S(0) in fig S10 is clearly unconverged, but the dG in fig S12 are converged. Can they say more about this?

Authors:
The referee is correct that the S(0) values in Fig.S10 in the SI (reproduced below) are not converged at small system sizes (≤1024 atoms), and only become converged when the system size reaches 1728 atoms and above.

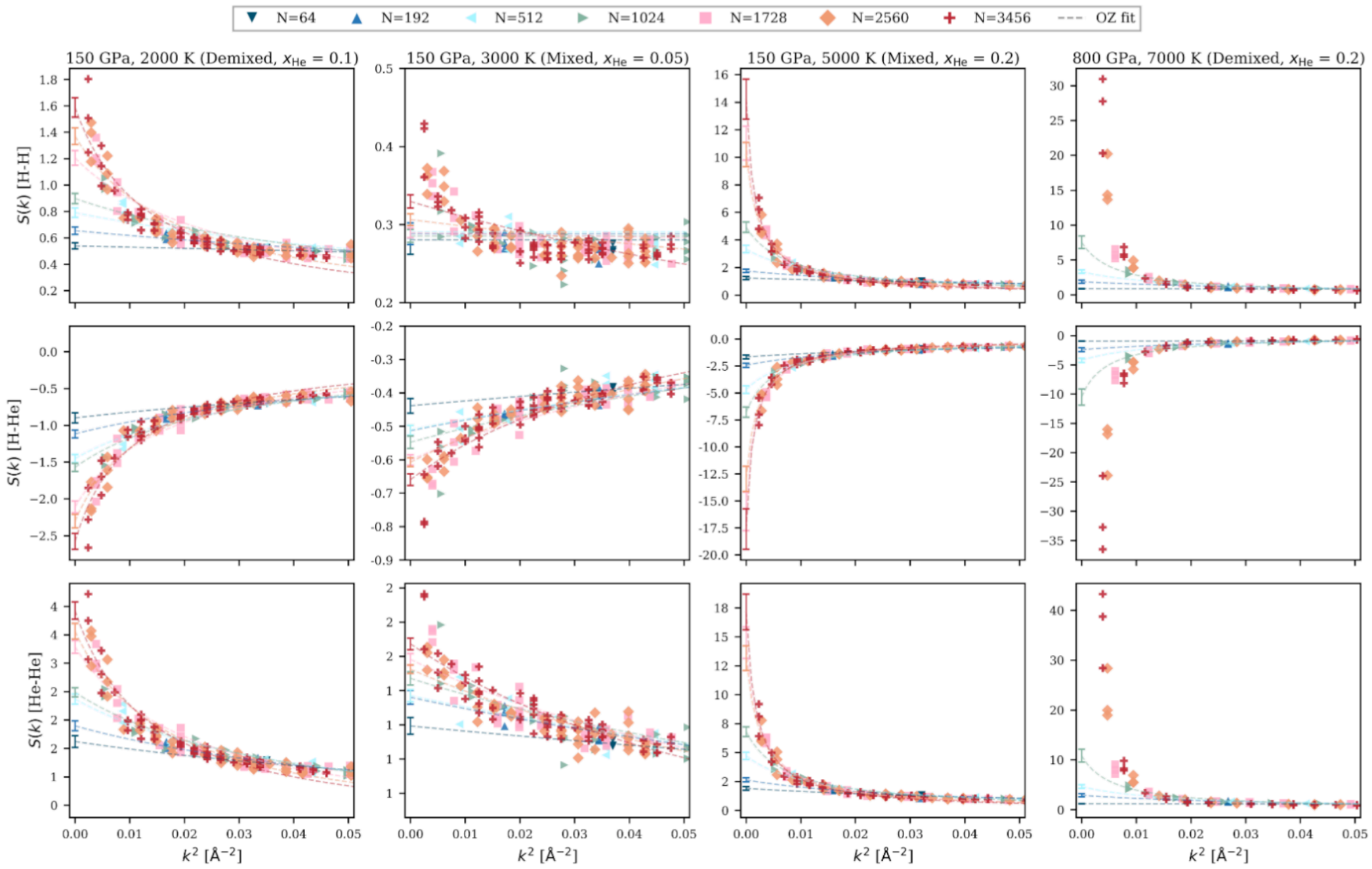

FIG. 10. System-size dependence of the structure factors $S(k)$ for H-H, H-He, and He-He pairs from $N = 64$ to $N = 3456$. Each column corresponds to a distinct P-T-x_{He} condition. Dashed lines indicate Ornstein–Zernike (OZ) fits, while error bars at $k^2 = 0$ denote the extrapolated S_0 limits.

Likewise, Fig.S12 in the SI are not converged at small system sizes (≤1024 atoms), and only becomes converged when the system size reaches 1728 atoms and above: the chemical potential gradient (upper panel), chemical potentials (middle panel), and the mixing free energy (ΔG^{mix}) from the MD using 1728 atoms (pink), 2526 atoms (orange), and 3456 atoms (red) are consistent, while the simulations based on smaller system sizes (192 atoms in dark blue; 512 atoms in light blue, 1024 atoms in green) show large deviations.

We have remade Fig.S12 to make the ΔG^{mix} more clear (reproduced below). Previously, the y-axis range for that plot was too large, and now we have adjusted this to improve the visibility of the ΔG^{mix} results.

In addition, we expanded the discussion in the SI:
“Fig.S10 shows the structure factor S(k) and also the extrapolation to the S(0) values using the Ornstein–Zernike (OZ) fits. The fitted S(0) values show finite size effects: they are not converged at small system sizes (≤1024 atoms), and only become converged when the system size reaches 1728 atoms and above.

Moreover, using the S0 method based on the S(0) values to determine the free energies of mixing, we find that the calculated ΔG^{mix} curves are also prone to finite-size effects: they require larger system sizes (N≥1728) to achieve convergence; see Fig.S12. Notably, simulations with N≤512 (dark blue lines) may fail to capture phase separation as the computed ΔG^{mix} remains artificially convex due to suppressed composition fluctuations. ”

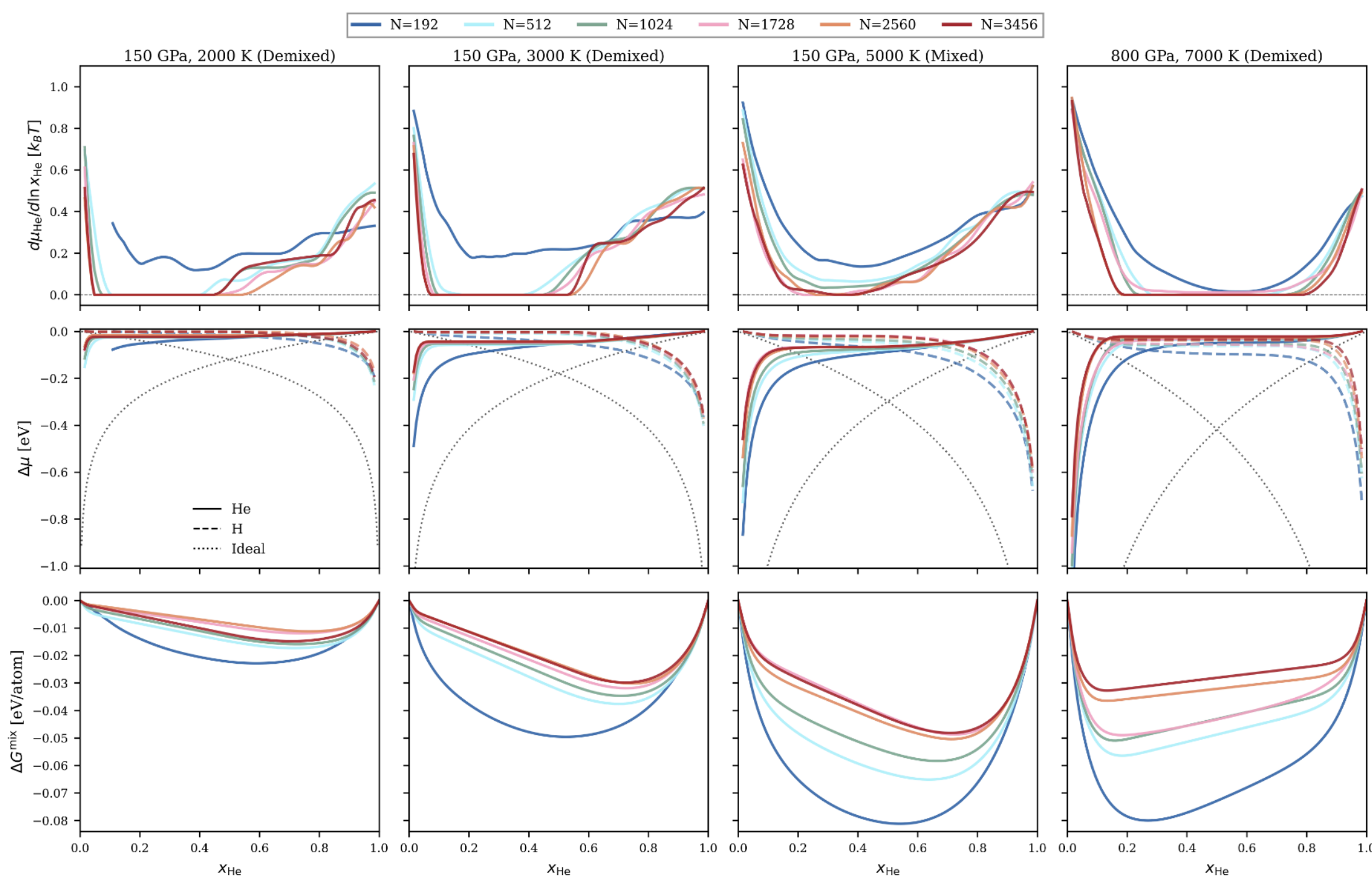

FIG. 12. System-size dependence of three thermodynamic properties from $N = 64$ to 3456: the derivative of the helium chemical potential $(\partial\mu_{He}/\partial \ln x_{He})_{T,P}$, chemical potentials ($\Delta\mu$) of He (solid lines) and H (dashed lines), and per-atom mixing free energy (ΔG^{mix}). Each column corresponds to a distinct P-T condition. Dotted gray lines indicate ideal solution behaviors.

I asked before:

"This would mean they calculated two physical miscibility boundaries in the planets from just one in the PT phase diagram."

I think they misunderstood the question. They state that there is only one immiscibility surface in the three-dimensional P-T-xHe space (I agree). But what I meant was that there are miscibility boundaries in the planets: i.e. as one descends into the planet, there are two crossings of this surface (miscible -> immiscible -> miscible). This is strongly implied by Fig 2b. But if I understand the figure correctly, it should mean that the brown and red lines in figure 2d-f cross

the black line twice? So the two-boundary picture in 2b applies only to PBE data and a couple of th e thermal profiles. So 2b shows an unusual scenario for their calculations, applying only to the least reliable of the functionals - is that correct? Also, in figs 2d-f it seems that the planetary thermal profiles don't cross into the molecular region for the chosen P range. That's not wrong, but creates a little confusion comparing to 2b

I'm fairly sure this is more a matter of clarification than criticism.

Authors:
We thank the referee for clarifying this question.

We have now realized that the original Fig. 2b could be misleading. We have modified this figure (reproduced below) by extending the rains towards the core.
In addition, we modified the caption of Fig. 2b to highlight that it serves as a schematic of different possible scenarios:
"(b) A schematic of the possible scenarios of the H/He immiscibility and helium rain in the H/He envelope of gas giants."

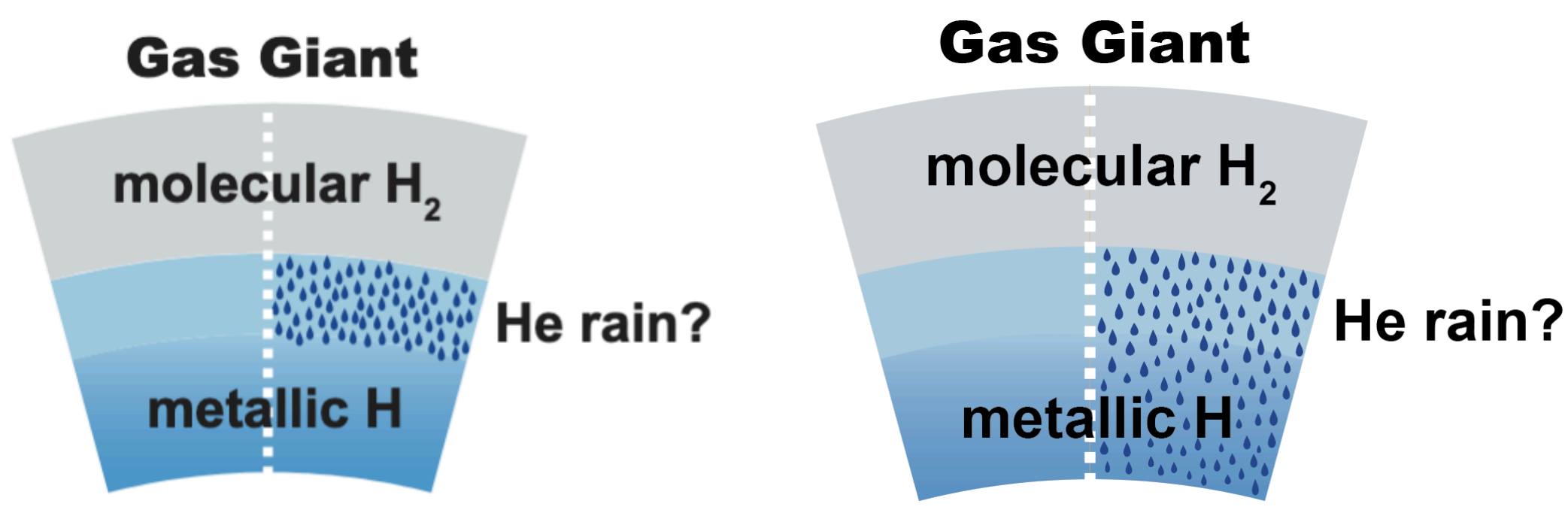

Figure: Previous (left panel) versus revised (right panel) versions of Fig. 2b in the manuscript.

As the referee pointed out, if the planetary thermal profile crosses the thermodynamic phase boundary once (as is the case for most of the conditions we studied), there would be one miscibility boundary in the planet (miscible -> immiscible). If the planetary thermal profile crosses the thermodynamic phase boundary twice (as can happen in the high He fraction region on the PBE phase diagram), there would be two miscibility boundaries in the planet (miscible -> immiscible -> miscible). However, the two-boundary scenario is less likely than the one-boundary case, as only one DFT functional (PBE) can produce this and the required He fraction ($x_{He} \geq 0.15$) is higher than protosolar abundance ($x_{He} = 0.089$).

Also in figure 2, they show the black line crossing the atomic-molecular boundary. This implies that helium is immiscible in fluid molecular hydrogen. It that really what the model shows?

Authors:

Indeed, the phase diagrams in Figs. 2(d-f) suggest that helium can be immiscible in molecular hydrogen, at conditions below both the atomic-molecular hydrogen boundary and the H-He immiscibility boundary. We have added a sentence to the main text to highlight this:
“Within the H-He immiscibility region, atomic hydrogen dominates, but there is a narrow range of thermodynamic conditions ($T \leqq 2000$ K and $P \leqq 200$ GPa) where molecular hydrogen is stable.”

Reviewer #2 (Remarks on code availability):

I have not tried to run the code. Given the number of review requests I get and the timescales for reviewing 60 pages of MS and SM, this is not practical without funding.

Reviewer #4 (Remarks to the Author):

I appreciate the authors thoughtful responses to my concerns. I am happy to now recommend publication.

Authors:
We thank the referee for the positive assessment.

Reviewer #5 (Remarks to the Author):

I co-reviewed this manuscript with one of the reviewers who provided the listed reports. This is part of the Nature Communications initiative to facilitate training in peer review and to provide appropriate recognition for Early Career Researchers who co-review manuscripts.

Authors:
We thank the referee for examining our work carefully.

This manuscript presents a first-principles study of hydrogen–helium immiscibility under planetary interior conditions. Using large-scale molecular dynamics driven by machine-learning potentials trained on the PBE, vdW-DF, and HSE functionals together with the S_0 chemical-potential method, the authors compute composition-dependent free energies and map the H/He immiscibility boundary over a wide pressure–temperature range. A central result is that the predicted demixing temperatures are ~2000 K lower than those from earlier ab initio studies using small simulation cells, which is attributed mainly to finite-size effects and enforced homogeneity. By comparison with planetary interior models, the authors conclude that helium rain is likely in Saturn but unlikely in Jupiter at protosolar helium abundance. The study is technically solid and has significant implications for planetary physics.

Regarding this investigation, I have the following questions:

1. The MLPs are trained on configurations obtained from DFT cells and then applied in large-scale MLP-driven MD simulations. Could the authors comment on whether both the size of the training DFT cells and the system size used in the MLP MD simulations are sufficient to fully capture the H/He mixing free energy and immiscibility boundary—particularly near the molecular–atomic hydrogen transition—and how sensitive the results are to the chosen system size?
2. The results rely on DFT reference data generated with the PBE, vdW-DF, and HSE functionals under extreme pressure–temperature conditions. Could the authors comment on the expected reliability of these exchange–correlation functionals in this regime, and on whether possible functional deficiencies could systematically affect the predicted H/He mixing free energies and immiscibility boundary?
3. The simulations are based on molecular dynamics using machine-learning potentials trained on finite-temperature DFT data. Could the authors comment on the possible role of nuclear quantum effects, in particular zero-point energy, on the H/He mixing free energy and immiscibility boundary, especially near the molecular–atomic hydrogen transition?
4. If helium rain occurs, the local helium concentration x_{He} will evolve over time as helium precipitates and redistributes. Could the authors comment on how a decreasing local x_{He} would affect the miscibility behavior and immiscibility boundary, and whether their thermodynamic results can be used to infer the qualitative time evolution of helium rain in planetary interiors?
